# Identification of a gene for an ancient cytokine, interleukin 15-like, in mammals; interleukins 2 and 15 co-evolved with this third family member, all sharing binding motifs for IL-15Rα

**DOI:** 10.1007/s00251-013-0747-0

**Published:** 2013-11-26

**Authors:** Johannes M. Dijkstra, Fumio Takizawa, Uwe Fischer, Maik Friedrich, Veronica Soto-Lampe, Christophe Lefèvre, Matthias Lenk, Axel Karger, Taei Matsui, Keiichiro Hashimoto

**Affiliations:** 1 Institute for Comprehensive Medical Science, Fujita Health University, Dengakugakubo 1-98, Toyoake, Aichi 470-1192 Japan; 2 Institute of Infectology, Friedrich-Loeffler-Institute, Boddenblick 5A, 17498 Insel Riems, Germany; 3 Institute of Clinical Immunology, Max-Bürger Research Centre, Johannisallee 30, 04103 Leipzig, Germany; 4 Centre for Biotechnology and Interdisciplinary Sciences, Deakin University, Pigdons Road, Geelong, Victoria 3217 Australia; 5 Institute of Molecular Biology, Friedrich-Loeffler-Institute, Boddenblick 5A, 17498 Insel Riems, Germany

**Keywords:** Cytokine, Evolution, Interleukins 2, 15 and 15-like, Receptor

## Abstract

Interleukins 2 and 15 (IL-2 and IL-15) are highly differentiated but related cytokines with overlapping, yet also distinct functions, and established benefits for medical drug use. The present study identified a gene for an ancient third IL-2/15 family member in reptiles and mammals, *interleukin 15*-*like* (*IL*-*15L*), which hitherto was only reported in fish. *IL*-*15L* genes with intact open reading frames (ORFs) and evidence of transcription, and a recent past of purifying selection, were found for cattle, horse, sheep, pig and rabbit. In human and mouse the *IL*-*15L* ORF is incapacitated. Although deduced IL-15L proteins share only ~21 % overall amino acid identity with IL-15, they share many of the IL-15 residues important for binding to receptor chain IL-15Rα, and recombinant bovine IL-15L was shown to interact with IL-15Rα indeed. Comparison of sequence motifs indicates that capacity for binding IL-15Rα is an ancestral characteristic of the IL-2/15/15L family, in accordance with a recent study which showed that in fish both IL-2 and IL-15 can bind IL-15Rα. Evidence reveals that the species lineage leading to mammals started out with three similar cytokines IL-2, IL-15 and IL-15L, and that later in evolution (1) IL-2 and IL-2Rα receptor chain acquired a new and specific binding mode and (2) IL-15L was lost in several but not all groups of mammals. The present study forms an important step forward in understanding this potent family of cytokines, and may help to improve future strategies for their application in veterinarian and human medicine.

**Electronic supplementary material:**

The online version of this article (doi:10.1007/s00251-013-0747-0) contains supplementary material, which is available to authorized users.

## Introduction

A group of related short-chain helical cytokines IL-2, IL-4, IL-7, IL-9, IL-15 and IL-21 bind receptors that have an IL-2Rγ chain (also known as "common cytokine-receptor γ-chain" or "γ_c_") and play important roles in the immune system (Leonard 2008). Overall sequence similarity levels classify IL-2, IL-15 and IL-21 as a distinct subfamily (Parrish-Novak et al. 2002; Kono et al. 2008). Functional similarities indicate a close phylogenetic relationship between IL-2 and IL-15, because their respective receptor complexes IL-2Rα·IL-2Rβ·IL-2Rγ (Grabstein et al. 1994; Giri et al. 1994, 1995; Ring et al. 2012) and IL-15Rα·IL-2Rβ·IL-2Rγ (Grabstein et al. 1994; Giri et al. 1994, 1995; Ring et al. 2012) are unique by including IL-2Rβ chain and a chain of the IL-2Rα/15Rα family. The closely related IL-2Rα and IL-15Rα are encoded by tandemly duplicated genes (Anderson et al. 1995), are not related to other known cytokine receptor chains, and bind cytokines by their "sushi" domains (aliases "complement control protein" or "short consensus repeat" domains). The IL-2Rα and IL-15Rα chains confer cytokine specificity, and affinities that are much higher than those of the IL-2Rβ·IL-2Rγ receptor alone (Sugamura et al. 1996; Giri et al. 1995; Waldmann and Tagaya 1999). The binding affinity of IL-15 for IL-15Rα is exceptionally high (*K*_d_ = ~50 pM; e.g., Mortier et al. 2006), and IL-15 predominantly functions with co-expressed IL-15Rα in either membrane-bound or released form as a stable heterodimer that can stimulate other cells which express IL-2Rβ·IL-2Rγ (Dubois et al. 2002; Sandau et al. 2004; Mortier et al. 2008; Bergamaschi et al. 2008, 2012). This mode of presentation is called "trans-presentation", indicating that IL-15Rα is not expressed by the same cell as IL-2Rβ·IL-2Rγ. The binding affinity of IL-2 for IL-2Rα is much lower (*K*_d_ = ~20 nM; e.g., Myszka et al. 1996), and IL-2Rα tends to function within co-expressed IL-2Rα·IL-2Rβ · IL-2Rγ complexes that have the three receptor chains all inserted in the same membrane (*cis*-presentation) and can bind free secreted IL-2. Signaling through IL-2 and IL-15 receptors is mediated intracellularly by the cytoplasmic tails of IL-2Rβ and IL-2Rγ.

IL-2 protein is predominantly expressed by activated T cells (Taniguchi et al. 1983; Malek 2008), whereas dendritic cells and monocytes are important for expression of IL-15 protein (Waldmann 2006). In vitro assays show substantial overlap in IL-2 and IL-15 functions involving survival, proliferation and differentiation of various B, T and natural killer (NK) cell populations (Taniguchi et al. 1983; Grabstein et al. 1994; Waldmann 2006; Malek 2008; Ring et al. 2012). Pronounced differences between IL-2 and IL-15 functions, however, are apparent when comparing genetically engineered mice. IL-15-deficient mice show marked defects in the production/maintenance of NK cells, natural killer T (NKT) cells, intestinal intraepithelial lymphocytes, and CD8 memory cells (Kennedy et al. 2000; Waldmann 2006). In contrast, IL-2-deficient mice show lymphoproliferative and autoimmune disorder, caused by a defect in the production of CD4^+^CD25^+^Foxp3^+^ T regulatory (Treg) cells (Sadlack et al. 1995; Almeida et al. 2002; Malek 2008). The sensitivity of Treg cells to IL-2 correlates with their constitutive expression of high levels of IL-2Rα (alias CD25; Fontenot et al. 2005; Malek 2008).

Recombinant IL-2 has been established as an anti-cancer drug (Waldmann 2006), but because of the dual function of both enhancing and downregulating immune responses, modified IL-2 with less specificity for Treg has been developed (Levin et al. 2012; Liao et al. 2013). IL-15 lacks this duality and may be a more promising anti-cancer agent than IL-2, especially if its stability and potency are enhanced by recombinant combination with IL-15Rα (Mortier et al. 2006; Vincent et al. 2013). Blockage of IL-2Rα or IL-15Rα function is medically used, or investigated for that purpose, to halt lymphoma progression or inflammation (Waldmann 2006; Wang et al. 2010).

The sequences of short-chain type I helical cytokines are very poorly conserved, even among orthologues (Huising et al. 2006), which is exemplified by the initial inability to properly distinguish between *IL*-*2* and *IL*-*15* identity of chicken *IL*-*2* (Sundick and GillDixon 1997; Choi et al. 1999). More recently, however, the availability of whole genome sequences allowed reliable identification of *IL*-*2* and *IL*-*15* in various tetrapod species and teleost fishes because of gene synteny arguments (Kaiser and Mariani 1999; Bird et al. 2005; Bei et al. 2006; Fang et al. 2006; Gunimaladevi et al. 2007; Wang et al. 2007; Ohtani et al. 2008).

In teleost fish, a gene for an additional IL-2/15 family member was found which was designated IL-15-like (IL-15L; Bei et al. 2006; Gunimaladevi et al. 2007), alias IL-15x (Fang et al. 2006). The function of fish *IL*-*15L* was not determined. The present study is the first to identify *IL*-*15L* genes and transcripts in mammals, to carefully analyze deduced IL-15L molecular features, and to describe interaction of recombinant IL-15L with IL-15Rα. It also comprises the first thorough analysis of IL-2 versus IL-15 sequence evolution.

## Results and discussion

### Identification of IL-15L in genome sequences of reptiles and mammals

**Fig. 1:**
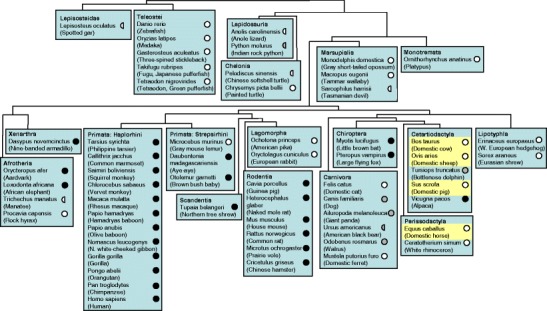
Phylogenetic distribution of *IL*-*15L. White circles* represent consensus intact *IL*-*15L* ORFs, *gray circles* represent non-typical but possibly intact *IL*-*15L* ORFs, and *black circles* represent incapacitated *IL*-*15L* ORFs; *half circles* refer to incomplete sequence information (for details, see Fig. S2). Mammals important for agriculture are highlighted in yellow. Branch knots within mammals are based on Meredith et al. (2011)

Probably because of its pseudogene nature in human and mouse, *IL*-*15L* has not been reported outside fish. However, after scrutinizing available genome sequence databases for vertebrate species, we here present *IL*-*15L* gene in reptiles and mammals, which as in fish maps between the genes *PLEKHG2* and *SUPT5H* (Fig. S1 *A*). In tetrapod *IL*-*15L* the family consensus intron between exons 3 and 4 was lost, without hampering the coding capacity, and the resulting larger exon is referred to in this article as "exon 3/4" (Fig. S1 *B*). In birds or amphibians *IL*-*15L* could not be found, despite extensive searches, and the gene may have been lost in these animal classes. The cladogram in Fig. 1 shows the distribution among species of *IL*-*15L*, and distinguishes between consensus intact open reading frames (ORFs) (white circles), non-typical but possibly intact ORFs (gray circles), and incapacitated ORFs (black circles); half circles refer to incomplete sequence information (for details, see Fig. S2). Intact *IL*-*15L* may be common in reptiles and non-eutherian mammals (monotremes plus marsupials), while in many eutherian mammals the ORF was incapacitated (Fig. 1 and Fig. S2). In eutherian mammals intact *IL*-*15L* ORF could be found in rock hyrax, gray mouse lemur, rabbit, pika, cat, ferret, horse, rhinoceros, cattle, sheep, pig, hedgehog, and shrew (Fig. 1 and Fig. S2), which interestingly include the four most important agricultural mammals (highlighted in yellow in Fig. 1). Database sequences may contain errors, and at the individual species level the detected ORF incapacitation motifs may not always represent the biological situation. However, by comparison of related species, such as for example among primates, some of the incapacitation motifs could be confirmed in independent databases (Fig. S2). In the human genome, large parts of incapacitated *IL*-*15L* remain, while in mouse only minor remnants are found (Fig. S2). Despite some modifications, the *IL*-*2*, *IL*-*15*, *IL*-*15L* and *IL*-*21* loci are relatively well conserved throughout classes of jawed vertebrates (Fig. S1 *A*), which may be rather common among genes of cytokines involved in T cell differentiation (Secombes et al. 2011). The relatively well conserved nature of *IL*-*2*/*15*/*15L*/*21* family loci contrasts the evolution pattern of other families of secreted mediators of the immune system such as chemokines and type I interferons, which experienced more extensive gene expansions, contractions and translocations (Nomiyama et al. 2008; Xu et al. 2013).

Independent *IL*-*15L* ORF incapacitations in different lineages of eutherian mammals (Fig. 1 and Fig. S2) suggest that after the separation from marsupials the IL-15L protein considerably lost in importance early in the eutherian mammal clade. However, lack of importance in one animal does not necessarily conclude the same for others. The eutherian mammal superorder in which IL-15L may have retained a relatively high importance, as indicated by the distribution of intact *IL*-*15L* ORFs, is Laurasiatheria, the superorder which includes Carnivora, Chiroptera, Cetartiodactyla, Perissodactyla, and Lipotyphla (Fig. 1). In some Cetartiodactyla and Perissodactyla like cattle, sheep, horse and rhinoceros, the potential importance at the protein level, which may have been retained, regained or newly acquired, is underlined by modifications around the start codon which are predicted to ensure a higher efficiency of translation (Fig. S2).

### Analysis of IL-15L transcripts in mammals

**Fig. 2:**
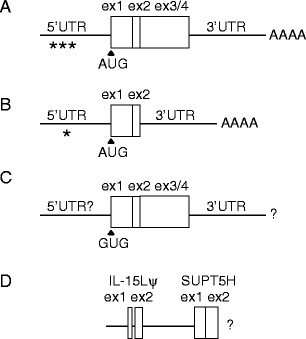
Schematic view of different types of mammalian *IL*-*15L* transcripts. **a** Transcript with consensus *IL*-*15L* ORF and additional out of frame AUGs in the 5′UTR (*asterisks*). Full-length transcripts including poly-A tail (AAAA) were determined for cattle and rabbit (Fig. S3). **b** In rabbits, however, most transcripts lack the exon3/4 sequence (ex3/4) and are unlikely to encode IL-15L protein (Figs. S3 *C*–*E* and S5*B*, middle picture). **c** In dog and bear, the consensus *IL*-*15L* AUG start codon has been replaced by GUG, and the region 5′ thereof is unlikely to encode the N-terminus of a functional cytokine (Figs. S2 and S4*A*–*C*). It was not determined whether these transcripts have a poly-A tail. **d** For human a hybrid *IL*-*15Lψ*-*SUPT5H* cDNA sequence was reported (GenBank DC400386), which is unlikely to encode modified IL-15L protein (Fig. S4 *D* and *E*). We did not investigate the 5′ and 3′ ends of this type of transcript

Evidence for IL-15L coding transcripts in cattle, horse, pig and sheep, as well as in rabbit, could be obtained (Figs. S3 and S5). The major types of transcripts that we found are schematically summarized in Fig. 2, with Fig. 2a showing a typical transcript that encodes intact IL-15L. Bovine and rabbit *IL*-*15L* transcripts were investigated extensively, including 5′RACE and 3′RACE analyses (Fig. S3 *A* and *C*). Bovine and rabbit *IL*-*15L* transcripts could be amplified from all investigated tissues and from various fibroblastoid and epithelioid permanent cell lines, and the results suggest low but ubiquitous expression (Fig. S5). A higher expression of *IL*-*15L* transcripts in organs of the immune system such as thymus, lymph nodes, or spleen, was not observed (Fig. S5), similar to previous findings in fish (Bei et al. 2006; Gunimaladevi et al. 2007). That mammalian *IL*-*15L* expression is generally low was not only indicated by the large number of PCR cycles necessary for detection (Fig. S5; and by our initial difficulties to establish positive RT-PCR conditions), but also by the near absence of *IL*-*15L* sequences in EST databases (see below).

In the 5′UTR of *IL*-*15L* transcripts additional out-of-frame AUG codons can be found (Fig. 2, asterisks), namely, three or six in cattle (Fig. S3 *A* and *B*) and one in rabbit (Fig. S3 *C* and *D*). Such upstream AUGs tend to interfere negatively with the efficiency of translation and are abundant in *IL*-*15* where they are believed to be important in translational control (Waldmann and Tagaya 1999).

Not all mammalian *IL*-*15L* transcripts have apparent protein coding capacity. In rabbit, most transcripts do not contain exon3/4 sequence, but are organized as schematically shown in Fig. 2b and are not expected to encode functional protein (Fig. S3 *C*–*E*). And in some species, like dogs and bears, the *IL*-*15L* consensus start codon has been replaced by GUG, which is schematically shown in Fig. 2c (details for these carnivores are shown in Fig. S4 *A*–*C*). Furthermore, as depicted in Fig. 2d, a human cDNA sequence reported in the EST database (GenBank DC400386) contains both an *IL*-*15Lψ* part and a part of the downstream *SUPT5H* gene (details in Fig. S4 *D* and *E*). This human *IL*-*15Lψ*-*SUPT5H* sequence and an American black bear EST sequence (GenBank GW294330; Fig. S4 *B*), which both presumably do not encode (modified) IL-15L protein (Fig. S4 *B* and *E*), are the only two mammalian *IL*-*15L* sequences in the NCBI EST database with evidence of transcription (intron sequences were spliced out). Transcripts that may not encode protein are not unique to mammalian *IL*-*15L*, since splice variants with no apparent protein coding function were also described for teleost fish *IL*-*15L* (Gunimaladevi et al. 2007) and mammalian *IL*-*15* is well known for its ubiquitous transcripts compared to a much more restricted protein distribution (Waldmann and Tagaya 1999).

Somewhat reminiscent of the *IL*-*15L* story is that of *interleukin 26* (*IL*-*26*). Although *IL*-*26* intact gene and IL-26 protein function were described for humans (Donnelly et al. 2010), *IL*-*26* ORF incapacitations were observed in several independent mammalian lineages, and multiple *IL*-*26* transcripts with unknown function were found in species with incapacitated ORF (Shakhsi-Niaei et al. 2013).

### Purifying selection on coding capacity of intact mammalian IL-15L genes

**Fig. 3:**
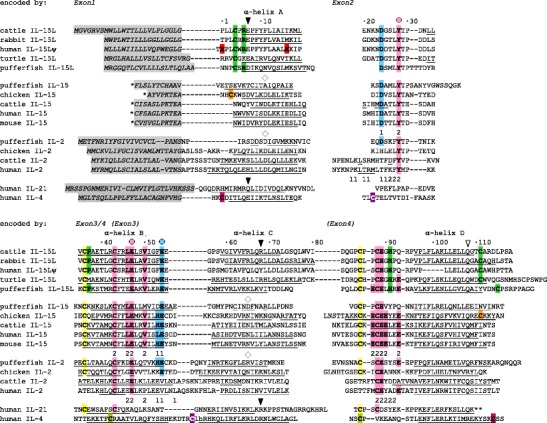
Alignment of deduced IL-15L, IL-15, IL-2, IL-21 and IL-4 amino acid sequences. *, for IL-15 only leader peptide fragments encoded by family consensus exon1 are shown. **, the sequence encoded by the last coding exon of *IL*-*21* is not shown. Small font in the IL-4 sequence relates to a fragment deleted for lay-out reasons (see Fig. S6 *A*). *Gaps*, open spaces relate to exon borders, whereas *hyphens* connect residues encoded by the same exon. *X shaded red*, frame-shift. Known or predicted leader peptide and α-helix sequences are shaded *gray* and *underlined*, respectively (based on IPD accessions 2PSM, 2Z3R, 1Z92, 1ITL and 3TGX for murine IL-15 and human IL-15, IL-2, IL-4 and IL-21, respectively). Similar colored cysteines refer to known or expected disulfide bridges. Several residues, including a cysteine pair, are rather specific for IL-15L and are shaded green. Murine IL-15 and human IL-2 residues with "1" or "2" indications below contribute to patches 1 and 2 of their interface with IL-15Rα and IL-2Rα, respectively. The patch 1 and 2 contributing residues of mammalian IL-15 are shaded *blue* and *pink*, respectively, as are matching identical residues in the other compared cytokines; *circles* above the alignment indicate the mammalian IL-15 residues which are key for IL-15Rα binding (Olsen et al. 2007). The *diamonds* indicate positions important for binding of IL-2 and IL-15 to IL-2Rβ chain, and the *open inverted triangle* indicates the glutamine important for interaction with IL-2Rγ chain (Wang et al. 2005; Ring et al. 2012). The *solid inverted triangles* indicate positions important for binding of IL-4 and IL-21 to IL-4Rα and IL-21Rα, respectively (Hage et al. 1999; Bondensgaard et al. 2007; Hamming et al. 2012). For database accessions, see Figs. S2 and S6 *A*

Comparison between the intact *IL*-*15L* ORFs found in various mammalian species indicates high rates of synonymous versus non-synonymous nucleotide substitutions with an average *d*_s_/*d*_n_ value of 5.3 (Table S2 *A*). This reveals purifying selection after these animals separated in time, and suggests protein function in the extant species. Similar analyses for *IL*-*15* (Table S2 *B*) and *IL*-*2* (Table S2 *C*) in the nearly same sets of mammalian species calculate d_s_/d_n_ values of only 3.8 and 2.8, respectively, which indicates that sequence conservation pressure on IL-15L is relatively high compared to its family members. At the protein level, the absolute degrees of conservation appear similar within IL-15 and IL-15L evolution, which in mammals are clearly higher than found for IL-2 (compare *d*_n_ values in Table S2, but also see Fig. 3 and Fig. S6 *A* and *C*).

### Analysis of deduced amino acid sequences; IL-15L is similar to IL-2 and IL-15

Figure 3 compares amino acid sequences deduced from partially reconstituted human *IL*-*15L* pseudogene (human IL-15Lψ) and from intact *IL*-*15L* of cattle, rabbit, painted turtle, and green pufferfish, with several IL-2 and IL-15 sequences and human IL-21 and IL-4. Figure S6 *A* shows a more extensive sequence comparison including additional IL-15L, IL-15, IL-2, IL-21 and IL-4 sequences, and also neoteleost fish-specific IL-2-like (IL-2L). Such comparison of many highly diverged but related sequences is helpful to deduce ancestral motifs and to understand their gradual divergence in some phylogenetic lineages. Our alignment is different from previous studies by inclusion of IL-15L and non-mammalian IL-2 sequences (if, e.g., compared with Olsen et al. 2007), or by proper alignment of binding motifs for IL-2Rα and IL-15Rα (if, e.g., compared with Bei et al. 2006 or Gunimaladevi et al. 2007).

Similar to other short-chain helical cytokines (Rozwarski et al. 1994), computer software predicts that IL-15L molecules have a leader peptide (Fig. 3, gray shading) and four α-helices A, B, C and D (Fig. 3, underlined). In Fig. 3, residues which are rather specific for IL-15L are shaded green, and known or expected disulfide bridges are indicated by identical color shading of contributing cysteines. A deduced ancestral motif that can be found in many IL-2, IL-15, IL-15L and IL-21 molecules, while distinguishing them from other short-chain helical cytokines, consists of two cysteine pairs (Fig. 3, yellow and pink C's), an LXTP motif (residues 27–30), F44, D/E89 and Q108 (Fig. 3 and Fig. S6 *A*; residue numbering throughout this article agrees with numbers above these alignments). The IL-15L molecules have an additional cysteine pair (Fig. 3, green C's) which may connect the N- and C-terminal regions by disulfide bridge as found in human IL-4 (magenta C's); a similar bridge may also be present in avian IL-15 (orange C's). The residues Y28, E47 and D/E54 are well conserved between IL-2, IL-15 and IL-15L, but are absent in most IL-21, and these are exactly the key residues for binding of IL-15 to IL-15Rα (Olsen et al. 2007; see below). This agrees with IL-21 not binding a sushi-domain containing receptor (Leonard 2008). It is notable that only after the ancestors of mammals and birds separated, IL-2 in the mammalian lineage differentiated from family consensus by losing a cysteine pair (Fig. 3, yellow C's), by getting a longer exon2, and by losing the above-mentioned D/E54 residue (Fig. 3 and Fig. S6 *A*).

### Phylogeny within the IL-2/15/15L family; IL-2 and IL-15 may be each other's closest relatives

The members of the short chain helix cytokine family rapidly diverged, and even cysteine bridges and helix lengths are not well conserved (Rozwarski et al. 1994). The ancientness of the phylogenetic issues in question, the evolutionary rapidness of the changes, the plasticity of the sequences and molecule structures, and also the shortness of this family of molecules, make it difficult for specialized software programs to resolve their phylogenetic relationships (Fig. S6 *B*; e.g., Gunimaladevi et al. 2007; Kono et al. 2008), because of the following reasons: (1) "chance occurrence" — rather than homogenous gradual diversification — played a relatively large role in how much the molecules diverged from the original; (2) various amino acid positions may be "saturated" from calculation perspective (Van de Peer et al. 2002); (3) alignments remain questionable. Therefore, for the distinction of IL-2/15/15L/21 and IL-2/15/15L as true phylogenetic (sub-)families, we believe that better evidence is provided by the distribution of "hallmark" motifs such as discussed in the above paragraph. Furthermore, percentages of identical amino acids also support the existence of the IL-2/15/15L family as a true phylogenetic group (Fig. S6 *C*). The similarity comparisons in Fig. S6 *C* underline that mammalian IL-2 differentiated to a relatively high degree from family consensus, an observation made as well for IL-2 in turtles (Fig. S6 *C*) which similar to mammalian IL-2 did not retain all family consensus cysteine pairs (Fig. S6 *A*).

Our alignments of the highly differentiated α-helices A and C (Fig. 3 and Fig. S6 *A*) remain discussable between different cytokines, but in some instances also between sequences of the same cytokine in different species. Nevertheless, for speculation on phylogeny within the IL-2/15/15L family it may be useful to look at the D11 + N65 motif which in both mammalian IL-2 and IL-15 is important for binding IL-2Rβ (e.g., Wang et al. 2005; Ring et al. 2012), and which is not found in IL-15L (diamonds in Fig. 3 and Fig. S6 *A*). IL-15L may share an evolutionary older motif for type I receptor binding, namely, E/Q7 + R68, with IL-21 and IL-4 (Hage et al. 1999; Bondensgaard et al. 2007; Hamming et al. 2012; inverted solid triangles in Fig. 3 and Fig. S6 *A*). The Q107 residue important for binding IL-2Rγ found in IL-2, IL-15 and IL-21 (Wang et al. 2005; Ring et al. 2012; Hamming et al. 2012) is highly conserved in IL-15L (inverted open triangle in Fig. 3 and Fig. S6 *A*), and we speculate that IL-15L can bind one of the type I receptors IL-2Rβ·IL-2Rγ or IL-21Rα·IL-2Rγ in a similar manner as by which IL-4 and IL-21 bind their respective type I receptors, which roughly includes one turn helical shifts of the cytokine helices A and C over the receptor binding site if compared with IL-2 and IL-15 (Bondensgaard et al. 2007; Hamming et al. 2012). If the mode by which IL-2 and IL-15 bind their type I receptor is unique and different from IL-15L indeed, that would suggest that IL-2 and IL-15 are each other's closest relatives. Future structural analyses of the type I receptor binding modes of IL-2/15/15L family members other than mammalian IL-2 and IL-15 should be helpful to clarify this matter.

### Conservation of binding motifs for IL-15Rα in the IL-2/15/15L family; recombinant IL-15L interacts with IL-15Rα

Olsen et al. (2007) distinguished the two regions of the mammalian IL-15·IL-15Rα interface as "patch 1" and "patch 2", and in Fig. 3 the IL-15 residues contributing to these interactions are indicated with "1" and "2" below and the residues are shaded blue and pink, respectively. Identical residues in the other aligned sequences are shaded equally, and the most important key residues for IL-15Rα interaction (Olsen et al. 2007) are indicated by circles above the alignment. Although the IL-15·IL-15Rα interface patches 1 and 2 spatially roughly correspond with the two regions of the mammalian IL-2·IL-2Rα interface (Fig. 3, contributing IL-2 residues are indicated with "1" and "2" below), only the patch 2 residues in mammalian IL-2 resemble those of IL-15 (Rickert et al. 2005; Wang et al. 2005; Stauber et al. 2006; Chirifu et al. 2007; Olsen et al. 2007; Ring et al. 2012). Whereas the dominant patch 1 interactions are charged in IL-15·IL-15Rα, they are hydrophobic in mammalian IL-2·IL-2Rα, with a prominent role for IL-2 residue F25 (Rickert et al. 2005; Olsen et al. 2007). Figure 3 shows that only in mammalian IL-2 the patch 1 residues markedly differ from IL-2/15/15L family consensus, and indeed it was found that in teleost fish both IL-2 and IL-15 can bind IL-15Rα (Wen et al. 2011).

**Fig. 4:**
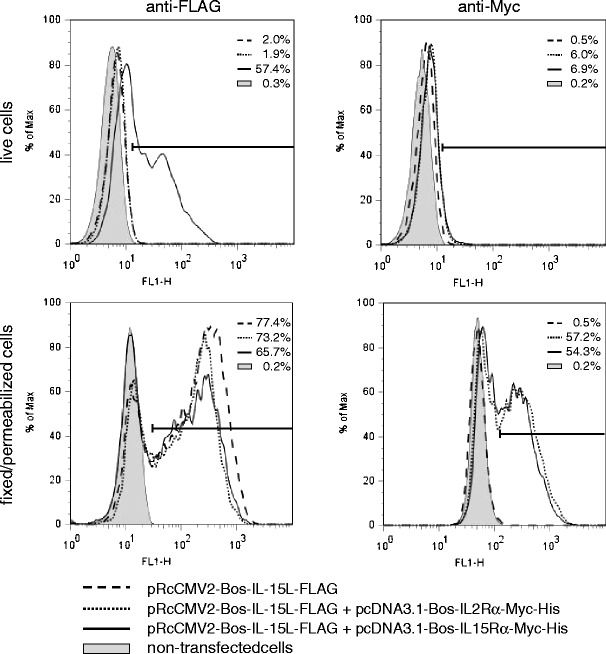
Surface presentation of IL-15L by IL-15Rα. The expression of bovine IL-15L-FLAG, bovine IL-2Rα-Myc-His and bovine IL-15Rα-Myc-His in transfected HEK-293 cells was assayed by flow cytometry using anti-FLAG (*left*) and anti-Myc (*right*) mAbs. Surface binding was monitored using live cells (*above*) and transfection efficiencies were monitored using fixed/permeabilized cells (*below*). Only in the presence of IL-15Rα receptor chain, IL-15L-FLAG could be observed at the cell surface by using anti-FLAG (*upper left histogram*). Because the Myc-tags of IL-2Rα and IL-15Rα were connected to the intracellular cytoplasmic tails, anti-Myc fluorescence could only be observed in fixed/permeabilized cells. Percentages refer to the cells within the fluorescence range indicated by horizontal bar. The data shown are of a single experiment, representative of three independent experiments

As expected from the sequence alignment (Fig. 3 and Fig. S6 *A*), IL-15L could be shown to interact with IL-15Rα in experiments using mammalian cells transfected with DNA plasmid expression vectors (Fig. S7 *A*). In particular, recombinant bovine IL-15L was found on the surface of transfected cells only in the presence of recombinant bovine IL-15Rα and not in the presence of recombinant bovine IL-2Rα or absence of recombinant receptor (Fig. 4, upper left histograms), while the IL-15L molecules could be found intracellularly in all three experiments (Fig. 4, lower left histograms). Furthermore, recombinant co-expression with soluble bovine IL-15Rα resulted in detectable IL-15L in the cell supernatant, whereas recombinant expression of IL-15L alone did not (Fig. S7 *B*). These experiments (Fig. 4 and Fig. S7) suggest that bovine IL-15L depends on interaction with IL-15Rα for efficient transport to and/or stability in the extracellular space, reminiscent of previous findings for IL-15 (Bergamaschi et al. 2008, 2012).

### Remarkable conservation throughout jawed vertebrates of the IL-15Rα motif for cytokine binding

**Fig. 5:**
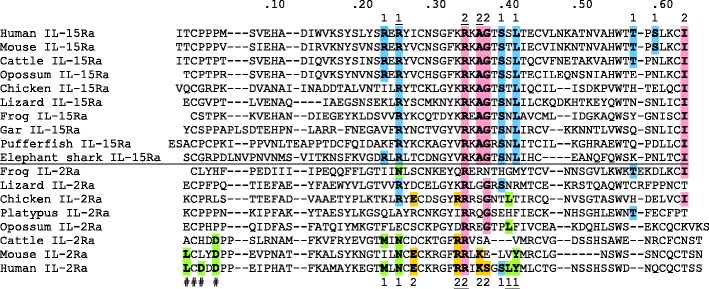
Alignment of major parts of the sushi-domain of IL-15Rα and the first sushi-domain of IL-2Rα in representative species. All depicted fragments are encoded by a single exon. The numbers 1 and 2 above and below the alignment indicate the human IL-15Rα and IL-2Rα residues, which interact with IL-15 and IL-2 in patches 1 and 2, respectively, with the important key residues being underlined (Rickert et al. 2005; Olsen et al. 2007). To highlight the evolutionary conservation of the IL-15Rα residues contributing to patches 1 and 2, these residues are shaded *blue* and *pink* throughout the alignment, including in IL-2Rα. Other human IL-2Rα residues contributing to binding patches 1 and 2, or binding to IL-2 residues contributing to those patches, are shaded *green* (except the sushi-domain specific cysteine) and *orange*, as are identical matching residues in the other aligned sequences. In human IL-2Rα, the first and second sushi-domains engage in a beta strand exchange event in which the first 18 residues depicted here are swapped with a stretch of domain 2; this 1–18 region is thus not functionally comparable with the IL-15Rα fragments to which it is aligned here. The IL-2Rα residues indicated by *pound symbols* (#) interact with IL-2 residues that contribute to patch 1 (Rickert et al. 2005). For GenBank accession numbers, see Fig. S1 *F*

Olsen et al. (2007) concluded that important IL-15Rα residues for binding IL-15 were well conserved between birds and mammals. Fang et al. (2007) already reported *IL*-*15Rα* in teleost fish, and in the present study our database searches identified *IL*-*15Rα* sequences in gar and elephant shark, which are primitive bony and cartilaginous fishes, respectively. Sequence comparisons show that also in IL-15Rα of fishes the motif for IL-15 binding is very well conserved (Fig. 5). In Fig. 5, the single-exon encoded major part of the sushi domain of IL-15Rα is aligned with corresponding sequences of the first sushi domain of IL-2Rα in representative animals; these sequence fragments contain most of the cytokine binding residues. To our knowledge, Fig. 5 constitutes the first analysis of the evolution of the cytokine binding motif of IL-2Rα. In fish no *IL*-*2R*α sequences could be found (see also Wen et al. 2011), and it is unclear whether in bony fish *IL*-*2R*α gene was lost or that *IL*-*2R*α was only established in the tetrapod line (Fig. S1 *C*–*F*).

In Fig. 5, the human IL-15Rα residues for binding IL-15 are colored blue and pink for patches 1 and 2, respectively, as was done for identical matching residues of the other sequences in the alignment. The well conserved IL-15Rα motif for binding IL-15 is not known in other sushi-domain molecules (http://smart.embl.de) and hence at the level of cartilaginous fish already an IL-2/15/15L family member with high affinity for IL-15Rα may have established, although we have not found a matching cytokine gene yet in this group of species for which sequence databases are incomplete. In databases of animals more primitive than jawed vertebrates, we could not find genes for IL-2/15/15L family cytokines or for IL-2Rα/15Rα receptors. In comparison with IL-15Rα to IL-15 binding, the IL-2Rα residues for binding IL-2 are conserved rather poorly between species (Fig. 5), which agrees with the diversification of the IL-2 sequences (Fig. 3 and Fig. S6) and the relative weakness of the binding between IL-2Rα and IL-2. The fact that the binding motif for IL-15Rα is well conserved in IL-15L suggests that this cytokine binds IL-15Rα with high affinity, but that remains to be determined experimentally.

Conservation of residue E47 throughout the IL-2/15/15L cytokines (Fig. 3 and Fig. S6 *A*) and residue R35 throughout the IL-2Rα/15Rα molecules agrees with the findings that these residues interact with each other in both IL-2·IL-2Rα and IL-15·IL-15Rα complexes (Rickert et al. 2005; Olsen et al. 2007). Also notable is that IL-15 residue E54 interacts with IL-15Rα residue R26 (Chirifu et al. 2007; Olsen et al. 2007), and that Figs. 3 and 5 and Fig. S6 *A* and show that mammalian IL-2 and IL-2Rα lack those residues, but that in IL-2 and IL-2Rα of several more primitive species including chicken those residues can be found. Thus, it will be interesting to investigate when in evolution IL-2 and IL-15 acquired specificity for their individual receptor chains IL-2Rα and IL-15Rα. In Text S1.2 we speculate about that development, also in regard to *cis*- versus *trans*-presentation and the association of IL-2 with Treg function.

## Conclusion and future prospects

IL-2 and IL-15 are among the most potent and best studied cytokines, and it is surprising that with *IL*-*15L* a gene for an unknown mammalian family member could be detected. Although in many eutherian mammals the *IL*-*15L* ORF was incapacitated, in the most important agricultural mammals intact *IL*-*15L* was found, and rates of synonymous versus non-synonymous nucleotide exchanges do suggest preservation of protein function. Our continuing lines of research involve a search for endogenous IL-15L protein, and production of stable recombinant IL-15L protein, the latter which currently proves difficult (not shown). After IL-15L function will be known we can speculate about possible use in veterinary medicine. Hopefully and importantly, future understanding of IL-15L·IL-15Rα structure may inspire widening of the range of agonists and antagonists for regulating IL-15 pathways in human medicine (e.g., Bernard et al. 2004; Zhu et al. 2009). Clarification of the evolution of the IL-2/15/15L family may also help to understand medically relevant mechanisms deciding between immune tolerance, in which IL-2 plays an important role, and inflammation mediated by IL-15. In summary, the intriguing conclusion of our study is that the mammalian IL-2 and IL-15 pathways developed in the presence of another IL-15Rα binding molecule, namely IL-15L. And, furthermore, that motifs for cytokine with sushi-domain receptor chain interaction were very well conserved despite enormous diversification of the overall cytokine sequences, with the notable exception of the IL-2 system in the mammalian lineage which partially acquired a new and unique cytokine-to-receptor binding mode.

## Materials and methods

### General

Details and additional materials and methods are described in Text S1.1.

### Database searching and genetic software analysis

Sequence databases at the National Center for Biotechnology Information (NCBI; http://www.ncbi.nlm.nih.gov/), Emsembl (http://www.ensembl.org/index.html) and the Elephant Shark Genome Project (http://esharkgenome.imcb.a-star.edu.sg/) were screened for *IL*-*2*, *IL*-*15*, *IL*-*15L*, *IL*-*2Rα* and *IL*-*15Rα*. Leader peptides were predicted by SignalP software (http://www.cbs.dtu.dk/services/SignalP/) and alpha-helices were predicted by Phyre software (http://www.sbg.bio.ic.ac.uk/~phyre/).

### Analysis by fluorescence-activated cell sorting (FACS) of transfected cells

HEK293 cells were transfected with plasmids described in Fig. S6, and 2 days after transfection half of the cells were fixed with 4 % paraformaldehyde, followed by permeabilization with 0.01 % digitonin. Both unfixed and fixed/permeabilized cells were subsequently stained with the murine monoclonal antibodies "ANTI-FLAG® M2" (Sigma) or "MAb to C-myc" (Meridian Life Science), washed, and then incubated with Alexa Fluor® 488 F(ab″)2 fragment of goat anti-mouse IgG (H + L) (Fisher Scientific). After a final washing step, cells were resuspended with FACS buffer containing propidium iodide (PI) and analyzed with a BD FACSCalibur flow cytometer (Becton Dickinson). Non-fixed PI-negative cells were regarded as live cells.

## Electronic supplementary material

Below is the link to the electronic supplementary material.

Table S1 (PDF 16 kb)

Table S2 (PDF 152 kb)

Text S1 (PDF 324 kb)

Fig. S1 (PDF 320 kb)

Fig. S2 (PDF 325 kb)

Fig. S3 (PDF 305 kb)

Fig. S4 (PDF 292 kb)

Fig. S5 (PDF 1208 kb)

Fig. S6 (PDF 306 kb)

Fig. S7 (PDF 490 kb)
