# Supplementary material for: Identification of a gene for an ancient cytokine, interleukin 15-like, in mammals; interleukins 2 and 15 co-evolved with this third family member, all sharing binding motifs for IL-15Rα
Source: Immunogenetics. 2013 Nov 26;66(2):93–103. doi: 10.1007/s00251-013-0747-0 (PMC3894449; doi:10.1007/s00251-013-0747-0)
Supplement: Supplementary file 1 — (PDF 16 kb) [file 251_2013_747_MOESM1_ESM.pdf]

Supplementary Table 1 (Table S1). Primer sequences

Name	Sequence
Cow-IL-15L-5'UTR-F	5'-CCCAGGCTGGAACCTGAGAA
Cow-IL-15L-5'UTR-3R-F	5'-CTTGTTTCCAGGAACCCAGGCTGGAACCTGAGAA
Cow-IL-15L-ex1-F1	5'-TCCATGTGGCTTCTCTGGACC
Cow-IL-15L-ex1-F2	5'-TTCCTCATTGCCATCACGAAGATG
Cow-IL-15L-ex1-R	5'-CAGCATCTTCGTGATGGCAATGA
Cow-IL-15L-ex3-R1	5'-GGAGAGTCTCAGCAGGACACA
Cow-IL-15L-ex3-R2	5'-ACCCGATCACAGACAACTCCAG
Cow-IL-15L-3'UTR-R1	5'-TACCAGGGAGACTTCCTCTGAAG
Cow-IL-15L-3'UTR-5R-R	5'-AGCTGGCCTCTTTGGGAGTACCAGGGAGAC
Cow-IL-15L-3'UTR-R2	5'-AAGTTCTCCAAGCAGTGATTATTTGTAGC
Cow-IL-15L-3'UTR-R3	5'-GTATTATTCCATTCATAGGAGATTCTAGAGCAG
Cow-IL-15L-Hind3start-F	5'-TACCAAGCTTACCATGTGGCTTCTCTGGACCAC
Cow-IL-15L-stopXbaI-R	5'-GTAGTCTAGATTATGCTGAGGGCAGGTCCC
Cow-IL-15L-FlagXbaI-R	5'-GTAGTCTAGATTACTTATCGTCGTCATCCTTGTAATCTGCTGAGGGCAGGTCCC
Cow-IL-15Rα-start-F	5'-CGATGTCCGGGCGGCT
Cow-IL-15Rα-stop-R	5'-CTTCCCTAGGTCCTCTCTGACTG
Cow-IL-15Rα-Hind3start-F	5'-TAGAAAGCTTACCATGTCCGGGCGGCTCCG
Cow-IL-15Rα-endXhoI-R	5'-TAGACTCGAGGTCCTCTCTGACTGGGGAAGAG
Cow-IL-15Rα-solXhoI-R	5'-TAGACTCGAGCTGGGATTGTTCTGATATGCACC
Cow-IL-2Rα-5'UTR-F	5'-CGGCGTTCTGCAGAGCAGCACT
Cow-IL-2Rα-3'UTR-R	5'-GATTGCGGTCCATGACATCTGTG
Cow-IL-2Rα-Hind3start-F	5'-TAGAAAGCTTACCATGGAGCCCAGCTTGCTGATG
Cow-IL-2Rα-endXhoI-R	5'-TAGACTCGAGATTGTCCTTCTGTTCTTCTTCCATTTCC
Rab-IL-15L-5'UTR-F	5'-ACCAGAGAAAGCCTCGGAGTG
Rab-IL-15L-5'UTR-3R-F	5'-ACCAGAGAAAGCCTCGGAGTGGGCAAGGT
Rab-IL-15L-ex1-F	5'-CTTCCTTGTGGCCATCATGAAGATAC
Rab-IL-15L-ex3-F	5'-CTTCAGGCTGGAGCTGGCT
Rab-IL-15L-ex3-R1	5'-CCTCAAACCCAATCACAGCCA
Rab-IL-15L-ex3-R2	5'-CATCCAGCAGGCGCTGTAG
Rab-IL-15L-ex3-5R-R	5'-ATCCAGCAGGCGCTGTAGCCGGAACAC
Rab-IL-15L-3'UTR-R1	5'-CTAGCCTGGGAGTCTCCGTC
Rab-IL-15L-3'UTR-R2	5'-ATCCGCACGGACAGACAGC
Gen-IL-15L-ex1-F1	5'-ATGTGGCCTCTCTGGACCAT
Gen-IL-15L-ex1-F2	5'-TTGCCATCATGAAGATGCTGGG
Gen-IL-15L-ex3-R1	5'-GCACAAGCCCCCTGTAATAACTC
Gen-IL-15L-ex3-R2	5'-TCCTTCACAGGGTGGACAAGG
Gen-IL-15L-3'UTR-R	5'-GAGTCTGTGTCCTYBVAGGC
Sheep-IL-15L-ex3-R	5'-CAACGGAGAGTCTCAGCAGGA
Horse-IL-15L-ex1-F	5'-CATTGTCCTGCTGATGCGGC
Dog-IL-15L-5'UTR-F	5'-CCAGAGAAAGCCTGGGTGGT
Dog-IL-15L-3'UTR-R	5'-AGCCTCTCCAGCAGTTCCAG
Human-IL-15L ex1-F	5'-TCTGAAGATACCAGCGAGTGG
Human-IL-15L ex2-F	5'-GACACCCTCTACACCCCAGA
Human-SUPT5H-R	5'-AAAGTTGCTGTCCTCGCTGT
GAPDH-F	5'-ACCATCTTCCAGGAGCGAGATC
GAPDH-R	5'-GTCTTCTGGGTGGCAGTGATG
UPM (Clontech)	5'-CTAATACGACTCACTATAGGGCAAGCAGTGGTATCAACGCAGAGT
NUP (Clontech)	5'-AAGCAGTGGTATCAACGCAGAGT
