# Supplementary material for: Identification of a gene for an ancient cytokine, interleukin 15-like, in mammals; interleukins 2 and 15 co-evolved with this third family member, all sharing binding motifs for IL-15Rα
Source: Immunogenetics. 2013 Nov 26;66(2):93–103. doi: 10.1007/s00251-013-0747-0 (PMC3894449; doi:10.1007/s00251-013-0747-0)
Supplement: Supplementary file 2 — (PDF 152 kb) [file 251_2013_747_MOESM2_ESM.pdf]

Supplementary Table 2 (Table S2).

Synonymous and nonsynonymous substitution rates between intact *IL-15L* ORFs (Table S2*A*), *IL-15* (Table S2*B*) and *IL-2* coding sequences (Table S2*C*) in the nearly same sets of eutherian mammals, dependent on whether (almost) complete *IL-15* or *IL-2* could be found for the respective species. Calculations were performed by SNAP software (**http://www.hiv.lanl.gov/content/sequence/SNAP/SNAP.html**). Frameshifts found in the coding sequences of *IL-15* and *IL-2* of Procavia (hyrax), and in *IL-15* of Microcebus (lemur), were interpreted as most likely due to sequencing errors and the alignments were corrected. For Sorex (shrew) and Ochotona (pika) *IL-15*, we could not detect the exon with start codon. For details of the analyzed sequences see Text S1.3.

Summaries of the comparisons shown on the following pages are as follows:

Supplementary Table 2*A* (Table S2*A*). Pages 2-3.
Synonymous and nonsynonymous substitution rates between intact *IL-15L* ORFs in 13 eutherian mammals: *Bos Taurus* (cattle), *Ovis aries* (domestic sheep), *Sus scrofa* (domestic pig), *Equus caballus* (horse), *Ceratotherium simum* (white rhinoceros), *Felis catus* (domestic cat), *Mustela putorius furo* (domestic ferret), *Erinaceus europaeus* (West-European hedgehog), *Sorex araneus* (Eurasian shrew), *Microcebus murinus* (gray mouse lemur), *Oryctolagus cuniculus* (European rabbit), *Ochotona princeps* (American pika), and *Procavia capensis* (rock hyrax).
Averages of all pairwise comparisons: ds = 0.4979, dn = 0.1000, **ds/dn = 5.2829**, ps/pn = 4.1092

Supplementary Table 2*B* (Table S2*B*). Pages 4-5.
Synonymous and nonsynonymous substitution rates between *IL-15* coding sequences in 12 eutherian mammals: *Bos Taurus* (cattle), *Ovis aries* (domestic sheep), *Sus scrofa* (domestic pig), *Equus caballus* (horse), *Ceratotherium simum* (white rhinoceros), *Felis catus* (domestic cat), *Mustela putorius furo* (domestic ferret), *Sorex araneus* (Eurasian shrew), *Microcebus murinus* (gray mouse lemur), *Oryctolagus cuniculus* (European rabbit), *Ochotona princeps* (American pika), and *Procavia capensis* (rock hyrax).
Averages of all pairwise comparisons: ds = 0.4481, dn = 0.1314, **ds/dn = 3.8075**, ps/pn = 3.0838

Supplementary Table 2*C* (Table S2*C*). Pages 6-7.
Synonymous and nonsynonymous substitution rates between *IL-2* coding sequences in 12 eutherian mammals: *Bos Taurus* (cattle), *Ovis aries* (domestic sheep), *Sus scrofa* (domestic pig), *Equus caballus* (horse), *Ceratotherium simum* (white rhinoceros), *Felis catus* (domestic cat), *Mustela putorius furo* (domestic ferret), *Erinaceus europaeus* (West-European hedgehog), *Sorex araneus* (Eurasian shrew), *Oryctolagus cuniculus* (European rabbit), *Ochotona princeps* (American pika), and *Procavia capensis* (rock hyrax).
Averages of all pairwise comparisons: ds = 0.6226, dn = 0.2083, **ds/dn = 2.8011**, ps/pn = 2.0441

Compare			Sequences_names	Sd	Sn	S	N	ps	pn	ds	dn	ds/dn	ps/pn
0	1	Bos_(cattle)_IL-15L	Ovis_(sheep)_IL-15L	7.0000	7.0000	106.6667	301.3333	0.0656	0.0232	0.0687	0.0236	2.9103	2.8250
0	2	Bos_(cattle)_IL-15L	Sus_(pig)_IL-15L	26.0000	20.0000	106.8333	301.1667	0.2434	0.0664	0.2942	0.0695	4.2313	3.6647
0	3	Bos_(cattle)_IL-15L	Equus_(horse)_IL-15L	30.0000	24.0000	107.0000	301.0000	0.2804	0.0797	0.3511	0.0843	4.1650	3.5164
0	4	Bos_(cattle)_IL-15L	Ceratotherium_(rhinoceros)_IL-15L	22.5000	26.5000	107.5000	300.5000	0.2093	0.0882	0.2454	0.0938	2.6158	2.3734
0	5	Bos_(cattle)_IL-15L	Felis_(cat)_IL-15L	32.0000	23.0000	106.0000	302.0000	0.3019	0.0762	0.3863	0.0803	4.8098	3.9639
0	6	Bos_(cattle)_IL-15L	Mustela_(ferret)_IL-15L	29.0000	23.0000	107.6667	300.3333	0.2693	0.0766	0.3337	0.0808	4.1310	3.5172
0	7	Bos_(cattle)_IL-15L	Erinaceus_(hedgehog)_IL-15L	39.6667	42.3333	104.0000	304.0000	0.3814	0.1393	0.5328	0.1540	3.4587	2.7389
0	8	Bos_(cattle)_IL-15L	Sorex_(shrew)_IL-15L	37.5000	38.5000	104.1667	303.8333	0.3600	0.1267	0.4904	0.1388	3.5334	2.8410
0	9	Bos_(cattle)_IL-15L	Microcebus_(lemur)_IL-15L	36.5000	30.5000	105.3333	302.6667	0.3465	0.1008	0.4650	0.1082	4.2966	3.4387
0	10	Bos_(cattle)_IL-15L	Oryctolagus_(rabbit)_IL-15L	40.5000	31.5000	106.5000	301.5000	0.3803	0.1045	0.5305	0.1125	4.7151	3.6398
0	11	Bos_(cattle)_IL-15L	Ochotona_(pika)_IL-15L	45.3333	42.6667	104.8333	303.1667	0.4324	0.1407	0.6445	0.1559	4.1352	3.0726
0	12	Bos_(cattle)_IL-15L	Procavia_(hyrax)_IL-15L	46.0000	39.0000	104.3333	303.6667	0.4409	0.1284	0.6648	0.1409	4.7192	3.4329
1	2	Ovis_(sheep)_IL-15L	Sus_(pig)_IL-15L	21.0000	19.0000	107.5000	300.5000	0.1953	0.0632	0.2263	0.0661	3.4260	3.0896
1	3	Ovis_(sheep)_IL-15L	Equus_(horse)_IL-15L	27.0000	24.0000	111.1667	311.8333	0.2429	0.0770	0.2935	0.0812	3.6142	3.1557
1	4	Ovis_(sheep)_IL-15L	Ceratotherium_(rhinoceros)_IL-15L	21.5000	24.5000	111.6667	311.3333	0.1925	0.0787	0.2225	0.0831	2.6764	2.4467
1	5	Ovis_(sheep)_IL-15L	Felis_(cat)_IL-15L	30.5000	19.5000	106.6667	301.3333	0.2859	0.0647	0.3600	0.0677	5.3201	4.4186
1	6	Ovis_(sheep)_IL-15L	Mustela_(ferret)_IL-15L	28.0000	20.0000	108.3333	299.6667	0.2585	0.0667	0.3169	0.0699	4.5337	3.8726
1	7	Ovis_(sheep)_IL-15L	Erinaceus_(hedgehog)_IL-15L	39.1667	40.8333	104.6667	303.3333	0.3742	0.1346	0.5183	0.1484	3.4931	2.7798
1	8	Ovis_(sheep)_IL-15L	Sorex_(shrew)_IL-15L	34.0000	36.0000	104.8333	303.1667	0.3243	0.1187	0.4248	0.1293	3.2860	2.7312
1	9	Ovis_(sheep)_IL-15L	Microcebus_(lemur)_IL-15L	31.0000	27.0000	106.0000	302.0000	0.2925	0.0894	0.3706	0.0952	3.8934	3.2711
1	10	Ovis_(sheep)_IL-15L	Oryctolagus_(rabbit)_IL-15L	41.0000	26.0000	107.1667	300.8333	0.3826	0.0864	0.5352	0.0918	5.8282	4.4267
1	11	Ovis_(sheep)_IL-15L	Ochotona_(pika)_IL-15L	46.8333	38.1667	105.5000	302.5000	0.4439	0.1262	0.6722	0.1381	4.8656	3.5184
1	12	Ovis_(sheep)_IL-15L	Procavia_(hyrax)_IL-15L	45.0000	38.0000	105.0000	303.0000	0.4286	0.1254	0.6355	0.1372	4.6305	3.4173
2	3	Sus_(pig)_IL-15L	Equus_(horse)_IL-15L	24.5000	13.5000	107.8333	300.1667	0.2272	0.0450	0.2707	0.0464	5.8357	5.0517
2	4	Sus_(pig)_IL-15L	Ceratotherium_(rhinoceros)_IL-15L	18.1667	14.8333	108.3333	299.6667	0.1677	0.0495	0.1898	0.0512	3.7065	3.3878
2	5	Sus_(pig)_IL-15L	Felis_(cat)_IL-15L	33.5000	11.5000	106.8333	301.1667	0.3136	0.0382	0.4061	0.0392	10.3617	8.2120
2	6	Sus_(pig)_IL-15L	Mustela_(ferret)_IL-15L	28.0000	13.0000	108.5000	299.5000	0.2581	0.0434	0.3163	0.0447	7.0740	5.9454
2	7	Sus_(pig)_IL-15L	Erinaceus_(hedgehog)_IL-15L	39.8333	35.1667	104.8333	303.1667	0.3800	0.1160	0.5299	0.1260	4.2047	3.2757
2	8	Sus_(pig)_IL-15L	Sorex_(shrew)_IL-15L	38.6667	34.3333	105.0000	303.0000	0.3683	0.1133	0.5065	0.1228	4.1230	3.2499
2	9	Sus_(pig)_IL-15L	Microcebus_(lemur)_IL-15L	32.0000	18.0000	106.1667	301.8333	0.3014	0.0596	0.3855	0.0621	6.2034	5.0542
2	10	Sus_(pig)_IL-15L	Oryctolagus_(rabbit)_IL-15L	39.0000	20.0000	107.3333	300.6667	0.3634	0.0665	0.4969	0.0697	7.1340	5.4624
2	11	Sus_(pig)_IL-15L	Ochotona_(pika)_IL-15L	48.3333	30.6667	105.6667	302.3333	0.4574	0.1014	0.7060	0.1090	6.4780	4.5095
2	12	Sus_(pig)_IL-15L	Procavia_(hyrax)_IL-15L	42.3333	29.6667	105.1667	302.8333	0.4025	0.0980	0.5771	0.1050	5.4969	4.1090
3	4	Equus_(horse)_IL-15L	Ceratotherium_(rhinoceros)_IL-15L	10.0000	11.0000	112.1667	310.8333	0.0892	0.0354	0.0949	0.0363	2.6182	2.5192
3	5	Equus_(horse)_IL-15L	Felis_(cat)_IL-15L	27.5000	13.5000	107.0000	301.0000	0.2570	0.0449	0.3147	0.0462	6.8044	5.7304
3	6	Equus_(horse)_IL-15L	Mustela_(ferret)_IL-15L	22.5000	14.5000	108.6667	299.3333	0.2071	0.0484	0.2423	0.0501	4.8386	4.2744
3	7	Equus_(horse)_IL-15L	Erinaceus_(hedgehog)_IL-15L	38.1667	34.8333	105.0000	303.0000	0.3635	0.1150	0.4972	0.1248	3.9842	3.1619
3	8	Equus_(horse)_IL-15L	Sorex_(shrew)_IL-15L	39.0000	30.0000	105.1667	302.8333	0.3708	0.0991	0.5116	0.1062	4.8151	3.7434
3	9	Equus_(horse)_IL-15L	Microcebus_(lemur)_IL-15L	32.0000	20.0000	106.3333	301.6667	0.3009	0.0663	0.3847	0.0694	5.5420	4.5392
3	10	Equus_(horse)_IL-15L	Oryctolagus_(rabbit)_IL-15L	37.0000	22.0000	107.5000	300.5000	0.3442	0.0732	0.4606	0.0770	5.9795	4.7013
3	11	Equus_(horse)_IL-15L	Ochotona_(pika)_IL-15L	49.8333	33.1667	105.8333	302.1667	0.4709	0.1098	0.7413	0.1187	6.2463	4.2899
3	12	Equus_(horse)_IL-15L	Procavia_(hyrax)_IL-15L	42.5000	34.5000	105.3333	302.6667	0.4035	0.1140	0.5791	0.1236	4.6838	3.5397
4	5	Ceratotherium_(rhinoceros)_IL-15L	Felis_(cat)_IL-15L	25.5000	15.5000	107.5000	300.5000	0.2372	0.0516	0.2852	0.0534	5.3359	4.5988
4	6	Ceratotherium_(rhinoceros)_IL-15L	Mustela_(ferret)_IL-15L	18.0000	17.0000	109.1667	298.8333	0.1649	0.0569	0.1862	0.0592	3.1473	2.8984
4	7	Ceratotherium_(rhinoceros)_IL-15L	Erinaceus_(hedgehog)_IL-15L	33.1667	32.8333	105.5000	302.5000	0.3144	0.1085	0.4075	0.1172	3.4754	2.8964
4	8	Ceratotherium_(rhinoceros)_IL-15L	Sorex_(shrew)_IL-15L	39.0000	33.0000	105.6667	302.3333	0.3691	0.1092	0.5081	0.1180	4.3076	3.3814
4	9	Ceratotherium_(rhinoceros)_IL-15L	Microcebus_(lemur)_IL-15L	25.0000	20.0000	106.8333	301.1667	0.2340	0.0664	0.2805	0.0695	4.0338	3.5238
4	10	Ceratotherium_(rhinoceros)_IL-15L	Oryctolagus_(rabbit)_IL-15L	30.5000	23.5000	108.0000	300.0000	0.2824	0.0783	0.3544	0.0827	4.2831	3.6052
4	11	Ceratotherium_(rhinoceros)_IL-15L	Ochotona_(pika)_IL-15L	42.8333	34.1667	106.3333	301.6667	0.4028	0.1133	0.5777	0.1228	4.7048	3.5566
4	12	Ceratotherium_(rhinoceros)_IL-15L	Procavia_(hyrax)_IL-15L	37.6667	36.3333	105.8333	302.1667	0.3559	0.1202	0.4826	0.1311	3.6825	2.9599
5	6	Felis_(cat)_IL-15L	Mustela_(ferret)_IL-15L	22.0000	4.0000	107.6667	300.3333	0.2043	0.0133	0.2386	0.0134	17.7516	15.3421
5	7	Felis_(cat)_IL-15L	Erinaceus_(hedgehog)_IL-15L	53.1667	30.8333	104.0000	304.0000	0.5112	0.1014	0.8584	0.1090	7.8772	5.0403
5	8	Felis_(cat)_IL-15L	Sorex_(shrew)_IL-15L	47.0000	32.0000	104.1667	303.8333	0.4512	0.1053	0.6902	0.1135	6.0818	4.2841
5	9	Felis_(cat)_IL-15L	Microcebus_(lemur)_IL-15L	37.5000	18.5000	105.3333	302.6667	0.3560	0.0611	0.4828	0.0638	7.5726	5.8245
5	10	Felis_(cat)_IL-15L	Oryctolagus_(rabbit)_IL-15L	43.5000	18.5000	106.5000	301.5000	0.4085	0.0614	0.5899	0.0640	9.2155	6.6566
5	11	Felis_(cat)_IL-15L	Ochotona_(pika)_IL-15L	50.3333	30.6667	104.8333	303.1667	0.4801	0.1012	0.7666	0.1087	7.0550	4.7465
5	12	Felis_(cat)_IL-15L	Procavia_(hyrax)_IL-15L	43.5000	32.5000	104.3333	303.6667	0.4169	0.1070	0.6088	0.1155	5.2721	3.8957
6	7	Mustela_(ferret)_IL-15L	Erinaceus_(hedgehog)_IL-15L	45.6667	31.3333	105.6667	302.3333	0.4322	0.1036	0.6439	0.1115	5.7733	4.1700
6	8	Mustela_(ferret)_IL-15L	Sorex_(shrew)_IL-15L	43.5000	30.5000	105.8333	302.1667	0.4110	0.1009	0.5956	0.1084	5.4941	4.0721
6	9	Mustela_(ferret)_IL-15L	Microcebus_(lemur)_IL-15L	37.5000	19.5000	107.0000	301.0000	0.3505	0.0648	0.4723	0.0678	6.9713	5.4098
6	10	Mustela_(ferret)_IL-15L	Oryctolagus_(rabbit)_IL-15L	44.0000	19.0000	108.1667	299.8333	0.4068	0.0634	0.5863	0.0662	8.8552	6.4193
6	11	Mustela_(ferret)_IL-15L	Ochotona_(pika)_IL-15L	53.5000	29.5000	106.5000	301.5000	0.5023	0.0978	0.8310	0.1048	7.9265	5.1342
6	12	Mustela_(ferret)_IL-15L	Procavia_(hyrax)_IL-15L	42.0000	33.0000	106.0000	302.0000	0.3962	0.1093	0.5636	0.1181	4.7719	3.6261

7	8	Erinaceus_(hedgehog)_IL-15L	Sorex_(shrew)_IL-15L	50.1667	40.8333	102.1667	305.8333	0.4910	0.1335	0.7975	0.1470	5.4242	3.6777
7	9	Erinaceus_(hedgehog)_IL-15L	Microcebus_(lemur)_IL-15L	41.6667	39.3333	103.3333	304.6667	0.4032	0.1291	0.5785	0.1417	4.0835	3.1233
7	10	Erinaceus_(hedgehog)_IL-15L	Oryctolagus_(rabbit)_IL-15L	41.6667	39.3333	104.5000	303.5000	0.3987	0.1296	0.5689	0.1423	3.9983	3.0766
7	11	Erinaceus_(hedgehog)_IL-15L	Ochotona_(pika)_IL-15L	53.6667	48.3333	102.8333	305.1667	0.5219	0.1584	0.8927	0.1779	5.0174	3.2950
7	12	Erinaceus_(hedgehog)_IL-15L	Procavia_(hyrax)_IL-15L	53.1667	48.8333	102.3333	305.6667	0.5195	0.1598	0.8850	0.1797	4.9261	3.2520
8	9	Sorex_(shrew)_IL-15L	Microcebus_(lemur)_IL-15L	47.3333	35.6667	103.5000	304.5000	0.4573	0.1171	0.7058	0.1274	5.5415	3.9044
8	10	Sorex_(shrew)_IL-15L	Oryctolagus_(rabbit)_IL-15L	50.0000	36.0000	104.6667	303.3333	0.4777	0.1187	0.7599	0.1292	5.8817	4.0251
8	11	Sorex_(shrew)_IL-15L	Ochotona_(pika)_IL-15L	50.1667	44.8333	103.0000	305.0000	0.4871	0.1470	0.7861	0.1636	4.8047	3.3134
8	12	Sorex_(shrew)_IL-15L	Procavia_(hyrax)_IL-15L	52.6667	50.3333	102.5000	305.5000	0.5138	0.1648	0.8666	0.1860	4.6583	3.1187
9	10	Microcebus_(lemur)_IL-15L	Oryctolagus_(rabbit)_IL-15L	26.0000	15.0000	105.8333	302.1667	0.2457	0.0496	0.2976	0.0514	5.7949	4.9489
9	11	Microcebus_(lemur)_IL-15L	Ochotona_(pika)_IL-15L	38.0000	25.0000	104.1667	303.8333	0.3648	0.0823	0.4997	0.0872	5.7338	4.4335
9	12	Microcebus_(lemur)_IL-15L	Procavia_(hyrax)_IL-15L	36.6667	37.3333	103.6667	304.3333	0.3537	0.1227	0.4784	0.1340	3.5716	2.8833
10	11	Oryctolagus_(rabbit)_IL-15L	Ochotona_(pika)_IL-15L	33.5000	13.5000	105.3333	302.6667	0.3180	0.0446	0.4138	0.0460	8.9986	7.1303
10	12	Oryctolagus_(rabbit)_IL-15L	Procavia_(hyrax)_IL-15L	48.3333	33.6667	104.8333	303.1667	0.4610	0.1111	0.7154	0.1202	5.9522	4.1517
11	12	Ochotona_(pika)_IL-15L	Procavia_(hyrax)_IL-15L	50.1667	45.8333	103.1667	304.8333	0.4863	0.1504	0.7839	0.1678	4.6713	3.2341

Compare		Sequences_names		Sd	Sn	S	N	ps	pn	ds	dn	ds/dn	ps/pn
0	1	Bos_(cattle)_IL-15	Ovis_(sheep)_IL-15	5.0000	9.0000	96.5000	389.5000	0.0518	0.0231	0.0537	0.0235	2.2876	2.2424
0	2	Bos_(cattle)_IL-15	Sus_(pig)_IL-15	18.5000	30.5000	95.8333	390.1667	0.1930	0.0782	0.2232	0.0826	2.7036	2.4695
0	3	Bos_(cattle)_IL-15	Equus_(horse)_IL-15	15.5000	32.5000	96.1667	389.8333	0.1612	0.0834	0.1815	0.0884	2.0533	1.9333
0	4	Bos_(cattle)_IL-15	Ceratotherium_(rhinoceros)_IL-15	19.1667	31.8333	96.6667	389.3333	0.1983	0.0818	0.2303	0.0866	2.6598	2.4250
0	5	Bos_(cattle)_IL-15	Felis_(cat)_IL-15	22.8333	38.1667	96.6667	389.3333	0.2362	0.0980	0.2837	0.1051	2.7003	2.4095
0	6	Bos_(cattle)_IL-15	Mustela_(ferret)_IL-15	25.5000	52.5000	96.0000	390.0000	0.2656	0.1346	0.3279	0.1484	2.2101	1.9732
0	7	Bos_(cattle)_IL-15	Sorex_(shrew)_IL-15	39.5000	86.5000	94.5000	379.5000	0.4180	0.2279	0.6112	0.2717	2.2494	1.8338
0	8	Bos_(cattle)_IL-15	Microcebus_(lemur)_IL-15	20.8333	32.1667	96.5000	386.5000	0.2159	0.0832	0.2546	0.0882	2.8861	2.5940
0	9	Bos_(cattle)_IL-15	Oryctolagus_(rabbit)_IL-15	29.6667	39.3333	98.3333	387.6667	0.3017	0.1015	0.3859	0.1090	3.5404	2.9735
0	10	Bos_(cattle)_IL-15	Ochotona_(pika)_IL-15	32.1667	45.8333	95.3333	378.6667	0.3374	0.1210	0.4482	0.1320	3.3955	2.7876
0	11	Bos_(cattle)_IL-15	Procavia_(hyrax)_IL-15	35.6667	42.3333	96.6667	389.3333	0.3690	0.1087	0.5079	0.1175	4.3235	3.3933
1	2	Ovis_(sheep)_IL-15	Sus_(pig)_IL-15	21.5000	35.5000	96.6667	389.3333	0.2224	0.0912	0.2638	0.0972	2.7137	2.4392
1	3	Ovis_(sheep)_IL-15	Equus_(horse)_IL-15	20.0000	37.0000	97.0000	389.0000	0.2062	0.0951	0.2411	0.1017	2.3704	2.1677
1	4	Ovis_(sheep)_IL-15	Ceratotherium_(rhinoceros)_IL-15	23.1667	36.8333	97.5000	388.5000	0.2376	0.0948	0.2857	0.1014	2.8190	2.5062
1	5	Ovis_(sheep)_IL-15	Felis_(cat)_IL-15	26.8333	44.1667	97.5000	388.5000	0.2752	0.1137	0.3429	0.1233	2.7814	2.4208
1	6	Ovis_(sheep)_IL-15	Mustela_(ferret)_IL-15	26.5000	58.5000	96.8333	389.1667	0.2737	0.1503	0.3405	0.1678	2.0295	1.8205
1	7	Ovis_(sheep)_IL-15	Sorex_(shrew)_IL-15	42.1667	89.8333	95.3333	378.6667	0.4423	0.2372	0.6682	0.2852	2.3431	1.8644
1	8	Ovis_(sheep)_IL-15	Microcebus_(lemur)_IL-15	25.3333	41.6667	97.3333	385.6667	0.2603	0.1080	0.3197	0.1167	2.7402	2.4091
1	9	Ovis_(sheep)_IL-15	Oryctolagus_(rabbit)_IL-15	34.1667	43.8333	99.1667	386.8333	0.3445	0.1133	0.4613	0.1228	3.7550	3.0406
1	10	Ovis_(sheep)_IL-15	Ochotona_(pika)_IL-15	34.6667	50.3333	96.1667	377.8333	0.3605	0.1332	0.4914	0.1467	3.3503	2.7060
1	11	Ovis_(sheep)_IL-15	Procavia_(hyrax)_IL-15	40.1667	47.8333	97.5000	388.5000	0.4120	0.1231	0.5977	0.1345	4.4441	3.3460
2	3	Sus_(pig)_IL-15	Equus_(horse)_IL-15	17.0000	25.0000	96.3333	389.6667	0.1765	0.0642	0.2012	0.0671	2.9999	2.7506
2	4	Sus_(pig)_IL-15	Ceratotherium_(rhinoceros)_IL-15	18.6667	26.3333	96.8333	389.1667	0.1928	0.0677	0.2228	0.0709	3.1421	2.8489
2	5	Sus_(pig)_IL-15	Felis_(cat)_IL-15	25.5000	42.5000	96.8333	389.1667	0.2633	0.1092	0.3244	0.1180	2.7484	2.4114
2	6	Sus_(pig)_IL-15	Mustela_(ferret)_IL-15	30.0000	57.0000	96.1667	389.8333	0.3120	0.1462	0.4033	0.1626	2.4798	2.1335
2	7	Sus_(pig)_IL-15	Sorex_(shrew)_IL-15	40.6667	84.3333	94.6667	379.3333	0.4296	0.2223	0.6378	0.2637	2.4189	1.9322
2	8	Sus_(pig)_IL-15	Microcebus_(lemur)_IL-15	28.6667	32.3333	96.6667	386.3333	0.2966	0.0837	0.3774	0.0887	4.2527	3.5433
2	9	Sus_(pig)_IL-15	Oryctolagus_(rabbit)_IL-15	34.1667	30.8333	98.5000	387.5000	0.3469	0.0796	0.4656	0.0841	5.5354	4.3593
2	10	Sus_(pig)_IL-15	Ochotona_(pika)_IL-15	38.1667	38.8333	95.5000	378.5000	0.3997	0.1026	0.5709	0.1103	5.1741	3.8953
2	11	Sus_(pig)_IL-15	Procavia_(hyrax)_IL-15	36.6667	43.3333	96.8333	389.1667	0.3787	0.1113	0.5272	0.1205	4.3739	3.4006
3	4	Equus_(horse)_IL-15	Ceratotherium_(rhinoceros)_IL-15	11.0000	15.0000	97.1667	388.8333	0.1132	0.0386	0.1227	0.0396	3.0987	2.9346
3	5	Equus_(horse)_IL-15	Felis_(cat)_IL-15	22.0000	29.0000	97.1667	388.8333	0.2264	0.0746	0.2695	0.0786	3.4311	3.0358
3	6	Equus_(horse)_IL-15	Mustela_(ferret)_IL-15	25.3333	50.6667	96.5000	389.5000	0.2625	0.1301	0.3231	0.1429	2.2617	2.0181
3	7	Equus_(horse)_IL-15	Sorex_(shrew)_IL-15	42.8333	75.1667	95.0000	379.0000	0.4509	0.1983	0.6894	0.2303	2.9930	2.2734
3	8	Equus_(horse)_IL-15	Microcebus_(lemur)_IL-15	23.3333	20.6667	97.0000	386.0000	0.2405	0.0535	0.2901	0.0555	5.2217	4.4929
3	9	Equus_(horse)_IL-15	Oryctolagus_(rabbit)_IL-15	27.5000	22.5000	98.8333	387.1667	0.2782	0.0581	0.3477	0.0605	5.7483	4.7879
3	10	Equus_(horse)_IL-15	Ochotona_(pika)_IL-15	31.0000	30.0000	95.8333	378.1667	0.3235	0.0793	0.4233	0.0838	5.0486	4.0776
3	11	Equus_(horse)_IL-15	Procavia_(hyrax)_IL-15	33.8333	31.1667	97.1667	388.8333	0.3482	0.0802	0.4681	0.0848	5.5219	4.3441
4	5	Ceratotherium_(rhinoceros)_IL-15	Felis_(cat)_IL-15	22.5000	28.5000	97.6667	388.3333	0.2304	0.0734	0.2752	0.0772	3.5635	3.1390
4	6	Ceratotherium_(rhinoceros)_IL-15	Mustela_(ferret)_IL-15	27.1667	52.8333	97.0000	389.0000	0.2801	0.1358	0.3506	0.1498	2.3400	2.0621
4	7	Ceratotherium_(rhinoceros)_IL-15	Sorex_(shrew)_IL-15	43.3333	82.6667	95.3333	378.6667	0.4545	0.2183	0.6987	0.2580	2.7079	2.0821
4	8	Ceratotherium_(rhinoceros)_IL-15	Microcebus_(lemur)_IL-15	24.5000	24.5000	97.5000	385.5000	0.2513	0.0636	0.3060	0.0664	4.6082	3.9538
4	9	Ceratotherium_(rhinoceros)_IL-15	Oryctolagus_(rabbit)_IL-15	32.0000	17.0000	99.3333	386.6667	0.3221	0.0440	0.4210	0.0453	9.2916	7.3273
4	10	Ceratotherium_(rhinoceros)_IL-15	Ochotona_(pika)_IL-15	37.5000	23.5000	96.1667	377.8333	0.3899	0.0622	0.5504	0.0649	8.4766	6.2696
4	11	Ceratotherium_(rhinoceros)_IL-15	Procavia_(hyrax)_IL-15	32.0000	31.0000	97.6667	388.3333	0.3276	0.0798	0.4307	0.0844	5.1025	4.1044
5	6	Felis_(cat)_IL-15	Mustela_(ferret)_IL-15	29.0000	50.0000	97.0000	389.0000	0.2990	0.1285	0.3814	0.1410	2.7051	2.3260
5	7	Felis_(cat)_IL-15	Sorex_(shrew)_IL-15	49.1667	79.8333	95.5000	378.5000	0.5148	0.2109	0.8698	0.2477	3.5123	2.4409
5	8	Felis_(cat)_IL-15	Microcebus_(lemur)_IL-15	29.0000	29.0000	97.5000	385.5000	0.2974	0.0752	0.3789	0.0793	4.7792	3.9538
5	9	Felis_(cat)_IL-15	Oryctolagus_(rabbit)_IL-15	33.5000	38.5000	99.3333	386.6667	0.3372	0.0996	0.4479	0.1068	4.1929	3.3871
5	10	Felis_(cat)_IL-15	Ochotona_(pika)_IL-15	44.1667	42.8333	96.3333	377.6667	0.4585	0.1134	0.7087	0.1230	5.7635	4.0425
5	11	Felis_(cat)_IL-15	Procavia_(hyrax)_IL-15	37.0000	42.0000	97.6667	388.3333	0.3788	0.1082	0.5276	0.1168	4.5172	3.5028
6	7	Mustela_(ferret)_IL-15	Sorex_(shrew)_IL-15	41.8333	97.1667	94.8333	379.1667	0.4411	0.2563	0.6654	0.3136	2.1220	1.7214
6	8	Mustela_(ferret)_IL-15	Microcebus_(lemur)_IL-15	28.6667	53.3333	96.8333	386.1667	0.2960	0.1381	0.3766	0.1526	2.4669	2.1435
6	9	Mustela_(ferret)_IL-15	Oryctolagus_(rabbit)_IL-15	31.3333	56.6667	98.6667	387.3333	0.3176	0.1463	0.4130	0.1627	2.5376	2.1707
6	10	Mustela_(ferret)_IL-15	Ochotona_(pika)_IL-15	39.3333	63.6667	95.6667	378.3333	0.4111	0.1683	0.5959	0.1906	3.1269	2.4432

6	11	Mustela_(ferret)_IL-15	Procavia_(hyrax)_IL-15	32.6667	54.3333	97.0000	389.0000	0.3368	0.1397	0.4471	0.1546	2.8924	2.4111
7	8	Sorex_(shrew)_IL-15	Microcebus_(lemur)_IL-15	39.3333	80.6667	95.0000	376.0000	0.4140	0.2145	0.6023	0.2527	2.3834	1.9299
7	9	Sorex_(shrew)_IL-15	Oryctolagus_(rabbit)_IL-15	44.1667	84.8333	97.0000	377.0000	0.4553	0.2250	0.7007	0.2675	2.6189	2.0235
7	10	Sorex_(shrew)_IL-15	Ochotona_(pika)_IL-15	49.3333	85.6667	96.1667	377.8333	0.5130	0.2267	0.8640	0.2700	3.2002	2.2626
7	11	Sorex_(shrew)_IL-15	Procavia_(hyrax)_IL-15	54.1667	85.8333	95.5000	378.5000	0.5672	0.2268	1.0587	0.2700	3.9206	2.5011
8	9	Microcebus_(lemur)_IL-15	Oryctolagus_(rabbit)_IL-15	30.0000	30.0000	99.1667	383.8333	0.3025	0.0782	0.3873	0.0825	4.6927	3.8706
8	10	Microcebus_(lemur)_IL-15	Ochotona_(pika)_IL-15	33.0000	39.0000	96.0000	375.0000	0.3438	0.1040	0.4598	0.1120	4.1073	3.3053
8	11	Microcebus_(lemur)_IL-15	Procavia_(hyrax)_IL-15	36.5000	38.5000	97.5000	385.5000	0.3744	0.0999	0.5186	0.1072	4.8386	3.7485
9	10	Oryctolagus_(rabbit)_IL-15	Ochotona_(pika)_IL-15	30.0000	14.0000	97.8333	376.1667	0.3066	0.0372	0.3943	0.0382	10.3287	8.2392
9	11	Oryctolagus_(rabbit)_IL-15	Procavia_(hyrax)_IL-15	42.6667	35.3333	99.3333	386.6667	0.4295	0.0914	0.6377	0.0974	6.5444	4.7005
10	11	Ochotona_(pika)_IL-15	Procavia_(hyrax)_IL-15	51.1667	44.8333	96.3333	377.6667	0.5311	0.1187	0.9237	0.1292	7.1479	4.4742T

Compare			Sequences_names	Sd	Sn	S	N	ps	pn	ds	dn	ds/dn	ps/pn
0	1	Bos_(cattle)_IL-2	Ovis_(sheep)_IL-2	5.0000	6.0000	100.1667	361.8333	0.0499	0.0166	0.0517	0.0168	3.0805	3.0103
0	2	Bos_(cattle)_IL-2	Sus_(pig)_IL-2	17.6667	51.3333	98.3333	360.6667	0.1797	0.1423	0.2054	0.1578	1.3013	1.2623
0	3	Bos_(cattle)_IL-2	Equus_(horse)_IL-2	16.5000	60.5000	93.6667	350.3333	0.1762	0.1727	0.2008	0.1963	1.0230	1.0201
0	4	Bos_(cattle)_IL-2	Ceratotherium_(rhinoceros)_IL-2	20.1667	62.8333	95.0000	349.0000	0.2123	0.1800	0.2496	0.2059	1.2121	1.1791
0	5	Bos_(cattle)_IL-2	Felis_(cat)_IL-2	18.0000	61.0000	100.3333	358.6667	0.1794	0.1701	0.2050	0.1929	1.0631	1.0548
0	6	Bos_(cattle)_IL-2	Mustela_(ferret)_IL-2	24.0000	64.0000	96.0000	354.0000	0.2500	0.1808	0.3041	0.2069	1.4700	1.3828
0	7	Bos_(cattle)_IL-2	Erinaceus_(hedgehog)_IL-2	70.5000	100.5000	102.1667	335.8333	0.6900	0.2993	1.8949	0.3819	4.9621	2.3059
0	8	Bos_(cattle)_IL-2	Sorex_(shrew)_IL-2	52.8333	82.1667	99.5000	341.5000	0.5310	0.2406	0.9232	0.2901	3.1820	2.2069
0	9	Bos_(cattle)_IL-2	Oryctolagus_(rabbit)_IL-2	29.6667	74.3333	97.6667	349.3333	0.3038	0.2128	0.3894	0.2503	1.5560	1.4275
0	10	Bos_(cattle)_IL-2	Ochotona_(pika)_IL-2	44.3333	81.6667	101.8333	357.1667	0.4354	0.2287	0.6515	0.2727	2.3886	1.9040
0	11	Bos_(cattle)_IL-2	Procavia_(hyrax)_IL-2	29.3333	85.6667	96.0000	345.0000	0.3056	0.2483	0.3924	0.3016	1.3013	1.2305
1	2	Ovis_(sheep)_IL-2	Sus_(pig)_IL-2	16.1667	51.8333	97.8333	361.1667	0.1652	0.1435	0.1867	0.1593	1.1718	1.1514
1	3	Ovis_(sheep)_IL-2	Equus_(horse)_IL-2	15.5000	59.5000	93.1667	350.8333	0.1664	0.1696	0.1881	0.1923	0.9784	0.9810
1	4	Ovis_(sheep)_IL-2	Ceratotherium_(rhinoceros)_IL-2	17.1667	62.8333	94.5000	349.5000	0.1817	0.1798	0.2080	0.2055	1.0120	1.0104
1	5	Ovis_(sheep)_IL-2	Felis_(cat)_IL-2	19.0000	63.0000	99.8333	359.1667	0.1903	0.1754	0.2195	0.1998	1.0987	1.0850
1	6	Ovis_(sheep)_IL-2	Mustela_(ferret)_IL-2	23.0000	64.0000	95.5000	354.5000	0.2408	0.1805	0.2905	0.2065	1.4065	1.3340
1	7	Ovis_(sheep)_IL-2	Erinaceus_(hedgehog)_IL-2	68.5000	102.5000	101.6667	336.3333	0.6738	0.3048	1.7147	0.3911	4.3845	2.2108
1	8	Ovis_(sheep)_IL-2	Sorex_(shrew)_IL-2	51.8333	84.1667	99.0000	342.0000	0.5236	0.2461	0.8982	0.2983	3.0114	2.1275
1	9	Ovis_(sheep)_IL-2	Oryctolagus_(rabbit)_IL-2	32.0000	75.0000	97.1667	349.8333	0.3293	0.2144	0.4337	0.2525	1.7175	1.5361
1	10	Ovis_(sheep)_IL-2	Ochotona_(pika)_IL-2	42.3333	82.6667	101.3333	357.6667	0.4178	0.2311	0.6107	0.2763	2.2101	1.8075
1	11	Ovis_(sheep)_IL-2	Procavia_(hyrax)_IL-2	29.0000	86.0000	95.5000	345.5000	0.3037	0.2489	0.3893	0.3025	1.2869	1.2200
2	3	Sus_(pig)_IL-2	Equus_(horse)_IL-2	17.5000	47.5000	92.6667	351.3333	0.1888	0.1352	0.2176	0.1491	1.4594	1.3968
2	4	Sus_(pig)_IL-2	Ceratotherium_(rhinoceros)_IL-2	24.0000	43.0000	94.0000	350.0000	0.2553	0.1229	0.3121	0.1342	2.3262	2.0782
2	5	Sus_(pig)_IL-2	Felis_(cat)_IL-2	21.0000	47.0000	99.0000	360.0000	0.2121	0.1306	0.2493	0.1434	1.7382	1.6248
2	6	Sus_(pig)_IL-2	Mustela_(ferret)_IL-2	31.0000	38.0000	94.6667	355.3333	0.3275	0.1069	0.4304	0.1154	3.7299	3.0621
2	7	Sus_(pig)_IL-2	Erinaceus_(hedgehog)_IL-2	69.1667	89.8333	101.0000	337.0000	0.6848	0.2666	1.8322	0.3294	5.5626	2.5690
2	8	Sus_(pig)_IL-2	Sorex_(shrew)_IL-2	51.6667	64.3333	98.0000	343.0000	0.5272	0.1876	0.9104	0.2158	4.2178	2.8109
2	9	Sus_(pig)_IL-2	Oryctolagus_(rabbit)_IL-2	32.8333	49.1667	96.8333	350.1667	0.3391	0.1404	0.4512	0.1555	2.9025	2.4149
2	10	Sus_(pig)_IL-2	Ochotona_(pika)_IL-2	41.5000	53.5000	100.5000	358.5000	0.4129	0.1492	0.5998	0.1664	3.6049	2.7671
2	11	Sus_(pig)_IL-2	Procavia_(hyrax)_IL-2	28.8333	75.1667	94.3333	346.6667	0.3057	0.2168	0.3926	0.2559	1.5341	1.4097
3	4	Equus_(horse)_IL-2	Ceratotherium_(rhinoceros)_IL-2	12.6667	21.3333	92.3333	351.6667	0.1372	0.0607	0.1515	0.0633	2.3951	2.2614
3	5	Equus_(horse)_IL-2	Felis_(cat)_IL-2	12.5000	35.5000	94.6667	349.3333	0.1320	0.1016	0.1452	0.1092	1.3300	1.2993
3	6	Equus_(horse)_IL-2	Mustela_(ferret)_IL-2	20.5000	35.5000	90.3333	344.6667	0.2269	0.1030	0.2703	0.1108	2.4395	2.2033
3	7	Equus_(horse)_IL-2	Erinaceus_(hedgehog)_IL-2	67.6667	82.3333	97.0000	332.0000	0.6976	0.2480	1.9958	0.3011	6.6285	2.8130
3	8	Equus_(horse)_IL-2	Sorex_(shrew)_IL-2	41.0000	61.0000	93.0000	333.0000	0.4409	0.1832	0.6647	0.2100	3.1649	2.4067
3	9	Equus_(horse)_IL-2	Oryctolagus_(rabbit)_IL-2	21.0000	46.0000	92.0000	340.0000	0.2283	0.1353	0.2722	0.1492	1.8243	1.6871
3	10	Equus_(horse)_IL-2	Ochotona_(pika)_IL-2	31.8333	49.1667	96.5000	347.5000	0.3299	0.1415	0.4346	0.1568	2.7721	2.3315
3	11	Equus_(horse)_IL-2	Procavia_(hyrax)_IL-2	20.3333	72.6667	89.1667	336.8333	0.2280	0.2157	0.2719	0.2544	1.0687	1.0570
4	5	Ceratotherium_(rhinoceros)_IL-2	Felis_(cat)_IL-2	12.0000	27.0000	96.0000	348.0000	0.1250	0.0776	0.1367	0.0819	1.6696	1.6111
4	6	Ceratotherium_(rhinoceros)_IL-2	Mustela_(ferret)_IL-2	18.5000	26.5000	91.6667	343.3333	0.2018	0.0772	0.2351	0.0815	2.8864	2.6148
4	7	Ceratotherium_(rhinoceros)_IL-2	Erinaceus_(hedgehog)_IL-2	62.3333	77.6667	98.8333	330.1667	0.6307	0.2352	1.3788	0.2823	4.8845	2.6811
4	8	Ceratotherium_(rhinoceros)_IL-2	Sorex_(shrew)_IL-2	39.3333	57.6667	94.8333	331.1667	0.4148	0.1741	0.6039	0.1981	3.0479	2.3819
4	9	Ceratotherium_(rhinoceros)_IL-2	Oryctolagus_(rabbit)_IL-2	26.5000	39.5000	93.3333	338.6667	0.2839	0.1166	0.3568	0.1268	2.8146	2.4344
4	10	Ceratotherium_(rhinoceros)_IL-2	Ochotona_(pika)_IL-2	38.0000	42.0000	97.8333	346.1667	0.3884	0.1213	0.5472	0.1323	4.1344	3.2013
4	11	Ceratotherium_(rhinoceros)_IL-2	Procavia_(hyrax)_IL-2	22.3333	64.6667	90.5000	335.5000	0.2468	0.1927	0.2993	0.2228	1.3433	1.2803
5	6	Felis_(cat)_IL-2	Mustela_(ferret)_IL-2	15.5000	26.5000	96.6667	353.3333	0.1603	0.0750	0.1804	0.0790	2.2830	2.1379
5	7	Felis_(cat)_IL-2	Erinaceus_(hedgehog)_IL-2	69.0000	86.0000	103.5000	334.5000	0.6667	0.2571	1.6479	0.3148	5.2344	2.5930
5	8	Felis_(cat)_IL-2	Sorex_(shrew)_IL-2	46.5000	62.5000	100.5000	340.5000	0.4627	0.1836	0.7196	0.2105	3.4184	2.5207
5	9	Felis_(cat)_IL-2	Oryctolagus_(rabbit)_IL-2	23.3333	44.6667	98.6667	348.3333	0.2365	0.1282	0.2841	0.1406	2.0202	1.8442
5	10	Felis_(cat)_IL-2	Ochotona_(pika)_IL-2	35.5000	46.5000	102.5000	356.5000	0.3463	0.1304	0.4646	0.1433	3.2425	2.6553
5	11	Felis_(cat)_IL-2	Procavia_(hyrax)_IL-2	20.0000	68.0000	96.0000	345.0000	0.2083	0.1971	0.2441	0.2287	1.0673	1.0570
6	7	Mustela_(ferret)_IL-2	Erinaceus_(hedgehog)_IL-2	69.3333	82.6667	98.8333	330.1667	0.7015	0.2504	2.0542	0.3047	6.7423	2.8018
6	8	Mustela_(ferret)_IL-2	Sorex_(shrew)_IL-2	45.3333	48.6667	96.0000	336.0000	0.4722	0.1448	0.7449	0.1609	4.6288	3.2603
6	9	Mustela_(ferret)_IL-2	Oryctolagus_(rabbit)_IL-2	32.5000	44.5000	94.5000	343.5000	0.3439	0.1295	0.4601	0.1422	3.2354	2.6547
6	10	Mustela_(ferret)_IL-2	Ochotona_(pika)_IL-2	45.0000	47.0000	98.1667	351.8333	0.4584	0.1336	0.7085	0.1471	4.8161	3.4315
6	11	Mustela_(ferret)_IL-2	Procavia_(hyrax)_IL-2	28.3333	63.6667	91.5000	340.5000	0.3097	0.1870	0.3994	0.2151	1.8570	1.6561
7	8	Erinaceus_(hedgehog)_IL-2	Sorex_(shrew)_IL-2	55.0000	84.0000	104.5000	327.5000	0.5263	0.2565	0.9074	0.3139	2.8907	2.0520
7	9	Erinaceus_(hedgehog)_IL-2	Oryctolagus_(rabbit)_IL-2	69.1667	85.8333	100.6667	325.3333	0.6871	0.2638	1.8587	0.3251	5.7167	2.6043
7	10	Erinaceus_(hedgehog)_IL-2	Ochotona_(pika)_IL-2	81.0000	88.0000	104.8333	333.1667	0.7727	0.2641	NA	0.0000	NA	2.9253
7	11	Erinaceus_(hedgehog)_IL-2	Procavia_(hyrax)_IL-2	67.0000	98.0000	98.0000	322.0000	0.6837	0.3043	1.8191	0.3904	4.6596	2.2464
8	9	Sorex_(shrew)_IL-2	Oryctolagus_(rabbit)_IL-2	55.1667	62.8333	97.8333	331.1667	0.5639	0.1897	1.0453	0.2187	4.7785	2.9720

8	10	Sorex_(shrew)_IL-2	Ochotona_(pika)_IL-2	56.6667	66.3333	101.5000	339.5000	0.5583	0.1954	1.0231	0.2263	4.5199	2.8574
8	11	Sorex_(shrew)_IL-2	Procavia_(hyrax)_IL-2	51.6667	81.3333	95.3333	327.6667	0.5420	0.2482	0.9618	0.3014	3.1906	2.1834
9	10	Oryctolagus_(rabbit)_IL-2	Ochotona_(pika)_IL-2	28.5000	28.5000	100.0000	347.0000	0.2850	0.0821	0.3585	0.0870	4.1216	3.4700
9	11	Oryctolagus_(rabbit)_IL-2	Procavia_(hyrax)_IL-2	26.6667	64.3333	93.3333	335.6667	0.2857	0.1917	0.3597	0.2213	1.6251	1.4907
10	11	Ochotona_(pika)_IL-2	Procavia_(hyrax)_IL-2	33.3333	76.6667	97.3333	343.6667	0.3425	0.2231	0.4575	0.2648	1.7277	1.5351
