# Supplementary material for: Identification of a gene for an ancient cytokine, interleukin 15-like, in mammals; interleukins 2 and 15 co-evolved with this third family member, all sharing binding motifs for IL-15Rα
Source: Immunogenetics. 2013 Nov 26;66(2):93–103. doi: 10.1007/s00251-013-0747-0 (PMC3894449; doi:10.1007/s00251-013-0747-0)
Supplement: Supplementary file 3 — (PDF 324 kb) [file 251_2013_747_MOESM3_ESM.pdf]

Supplementary Text 1 (Text S1).
Materials and methods.
Discussion on IL-2/15/15L family evolution in regard to binding to the sushi domain containing receptor chains IL-15Rα and IL-2Rα.
Sequences used for Table S2.

Table of Contents:

**Text S1.1**	Materials and methods	Page 2
**Text S1.2**	A hypothesis on the evolution of binding by IL-2/15/15L family cytokines to IL-2Rα/15Rα family receptor chains	Page 9
**Text S1.3**	Sequences used for Table S2	Page 11

Text S1.1

Materials and Methods

Bovine and rabbit cell lines.

Permanent bovine and rabbit cell lines were provided by the Collection of Cell Lines in Veterinary Medicine (CCLV) cell bank at the Friedrich-Loeffler-Institute, Insel Riems, Germany. Cells were grown in commercially available cell culture media containing 10% FBS at 37°C according to recommendations by the CCLV. RNA was extracted from 10^6 cells of each cell line using "NucleoSpin® RNA II" kit (MACHEREY-NAGEL).

cDNA synthesis, PCR amplification, and sequence analysis.

"Ready-to-Use Total RNA" samples of cattle, dog, horse, pig, rabbit, and sheep, were purchased from Zyagen Laboratories, and cDNA was synthesized using equal amounts of total RNA and "Superscript First-Strand Synthesis System for RT-PCR" (Invitrogen). Then equal amounts of cDNA solution were used for PCR using "ExTaq polymerase" (Takara) and primers indicated in Table S1. Annealing temperatures and extension times varied between experiments; amplification schedules were : 94°C for 5 min, 23-40x (94°C for 30 sec, 62-67°C for 30 sec, 72°C for 30-120 sec), 72°C for 7 min. For the experiments shown in Fig. S5, cDNA constructed with random hexamer primers was used, but similar though weaker results were obtained when using cDNA constructed with oligo-dT primers. For 5'RACE and 3'RACE analysis cDNA samples were constructed from total RNA using the "SMARTER RACE cDNA amplification system" (Clontech); from these cDNA preparations *IL-15L* fragments were amplified by nested PCR reactions (Fig. S3) using "ExTaq polymerase", 40 cycles for each reaction, touch-down temperature schedules, and extension times of $\geq$ 1min. Expression analyses using total RNA from bovine and rabbit permanent cell lines were done using a "OneStep RT-PCR Kit" (Qiagen).

PCR fragments were eluted from gel, cloned into plasmid vector, and multiple clones from independent experiments (to exclude PCR errors) were sequenced by dideoxy chain termination method and using an automated sequencer. Sequences of mammalian *IL-15L* cDNA obtained in this study are available as GenBank accessions JX271582-to-6 and KC914889.

Construction of DNA plasmid vectors for eukaryotic expression of bovine IL-15L, IL-15Rα and IL-2Rα.

IL-15L, *IL-15Rα* and *IL-2Rα* sequences were amplified by PCR from bovine cDNA samples. *IL-15L* amplification is described in Fig. S3. For bovine *IL-15Rα* amplification, the primer-set Cow-IL-15Rα-start-F + Cow-IL-15Rα-stop-R (for primers see Table S1) was used. With these, but also with other primers, several IL-15Rα splicoforms were amplified, reminiscent of the situation in human [e.g. reference 1]. For further cloning a full-length IL-15Rα clone was used. The first 823 nt of the ORF in this IL-15Rα clone were 100% identical with GenBank report XM_002692067, while the last part of the ORF was identical with ESTs at GenBank EV661551 and DN640106. For bovine *IL-2Rα* amplification, the primer-set Cow-IL-2Rα-5'UTR-F + Cow-IL-2Rα-3'UTR-R was used. This only amplified a single PCR fragment with a sequence 100% identical to GenBank accession BC133546, representing full-length *IL-2Rα*. *IL-15L*, *IL-15Ra*, and *IL-2Ra* were cloned into expression vectors as indicated below, and relevant sequences are shown in Fig. S7*A*.

Construction of pRcCMV2-Bos-IL-15L. For expression of bovine IL-15L we chose to use the consensus ATG start codon shared with other mammalian *IL-15L*, rather than the 21 nt upstream ATG which is in better context for translation. SignalP software predicts cleavage of leader peptides of both potential proteins at identical sites (GLG-PL) or at one residue apart

(the longer form has similar chances of being cleaved at GLG-PL or GL-GPL), so probably our choice did not affect experimental results.

By PCR amplification using primer-set Cow-IL-15L-Hind3start-F + Cow-IL-15L-stopXbaI-R and a full-length *IL-15L* clone as template, *Hin*DIII and *Xba*I sites were added upstream and downstream of *IL-15L*, respectively, and an ACC motif was positioned in front of the ATG start codon for efficient translation. Then, after digestion with *Hin*DIII and *Xba*I, the gene was cloned into *Hin*DIII/*Xba*I digested eukaryotic expression vector pRc/CMV2 (Invitrogen) behind a CMV promoter.

Construction of pRcCMV2-Bos-IL-15L-FLAG. Similar to construction of *pRcCMV2-Bos-IL-15L*, except that the primer-set Cow-IL-15L-Hind3start-F + Cow-IL-15L-FlagXbaI-R was used which added a FLAG coding sequence to the 3' end of *IL-15L*.

Construction of pcDNA3.1-Bos-IL-15Rα-Myc-His. By PCR amplification using primer-set Cow-IL-15Rα-Hind3start-F + Cow-IL-15Rα-endXhoI-R and a full-length *IL-15Rα* clone as template, *Hin*DIII and *Xho*I sites were added upstream and downstream of *IL-15Rα*, respectively, and an ACC motif was positioned in front of the ATG start codon for efficient translation. Then, after digestion with *Hin*DIII and *Xho*I, the gene was cloned into *Hin*DIII/*Xho*I digested eukaryotic expression vector pcDNA™3.1/myc-His B (Invitrogen) behind a CMV promoter, which added Myc and 6xHis coding sequences to the 3' end of *IL-15Rα.*

Construction of pcDNA3.1-Bos-sIL-15Rα-Myc-His. Similar to construction of *pcDNA3.1-Bos-IL-15Rα-Myc-His*, except that primer-set Cow-IL-15Rα-Hind3start-F + Cow-IL-15Rα-solXhoI-R was used for PCR amplification.

Construction of pcDNA3.1-Bos-IL-2Rα-Myc-His. Similar to construction of *pcDNA3.1-Bos-IL-15Rα-Myc-His*, except that primer-set Cow-IL-2Rα-Hind3start-F + Cow-

IL-2Rα-endXhoI-R and a full-length *IL-2Rα* clone as template were used for PCR amplification.

Analysis by fluorescence-activated cell sorting (FACS) of transfected cells.

Monolayer HEK-293 cells obtained from the CCLV cell bank were cultured in 6-well plates at 37°C in a 2.5% CO2 atmosphere with CCLV medium No. 5 (MEM (H) + MEM (E) + NEA containing 10% FBS). Cells were transfected with 1 μg of each plasmid per well using "FuGENE HD Transfection Reagent" (Roche). After two days, cells were detached by 0.02% EDTA PBS^- and resuspended with FACS buffer (Medium No. 5 containing 0.05% sodium azide). Then half of the cells were fixed with 4% paraformaldehyde for 10 min, followed by permeabilization with 0.01% digitonin PBS^- for 5 min. Both unfixed and fixed/permeabilized cells were subsequently incubated on ice for 30 min with the murine monoclonal antibodies "ANTI-FLAG® M2" (SIGMA) or "MAb to C-myc" (Meridian Life Science), washed, and then incubated for 20 min with Alexa Fluor® 488 F(ab")2 fragment of goat anti-mouse IgG (H+L) (Fisher Scientific). After a final washing step, cells were resuspended with FACS buffer containing propidium iodide (PI) and analyzed with a BD FACSCalibur flow cytometer (Becton Dickinson). Non-fixed PI-negative cells were regarded as live cells.

Analysis of IL-15L and sIL-15Rα secretion.

Cells. FS293 (Freestyle™ 293-F) cells are HEK293 (human embryo kidney) cells that were selected by Invitrogen for optimized growth as single cell suspensions in Freestyle medium ("Freestyle™293 expression medium", Invitrogen). Freestyle medium is serum-free, which is an advantage for isolation and detection of secreted proteins.

Transfection. FS293 were cultured in Freestyle medium in erlenmeyers under constant shaking at 37 °C in 8% CO_2 atmosphere. Such cell cultures were mixed 1:4 with DMEM (Sigma)/10% FBS medium and seeded into 6-well plates, which were placed at 37 °C in 5%

CO2 atmosphere. The next day the cell monolayers were transfected using lipofectamine™2000 (Invitrogen) with 4 µg *pRcCMV2-Bos-IL-15L*, 4 µg *pRcCMV2-Bos-IL-15L-FLAG*, 2 µg *pRcCMV2-Bos-IL-15L* + 2 µg *pcDNA3.1-Bos-sIL-15Rα-Myc-His*, or 2 µg *pRcCMV2-Bos-IL-15L-FLAG* + 2 µg *pcDNA3.1-Bos-sIL-15Rα-Myc-His* expression vectors per well. Negative control cells were not transfected. After 5 hours the cells were briefly washed with Freestyle medium, and then the cells were incubated for 3 days with 4 ml Freestyle medium per well at 37 °C in 8% CO_2 atmosphere (without shaking). Under these conditions the cells remained as a somewhat loose monolayer.

Sample isolation. Three days after transfection the cells and supernatants were separated. Unless mentioned otherwise, the steps were performed on ice or at 4 °C.

(a) Isolation of cell samples. After removing the supernatants (see below), the cells remaining in the two wells were directly resuspended in 8 ml fresh Freestyle medium. Then 100 µl samples hereof were spun down for 1 min at 900 x g after which the supernatants were discarded and the cell pellets were lysed in 20 µl 1x “NuPAG®LDS Sample Buffer” (Invitrogen)/ 50 mM dithiothreitol (DTT) to be later used as “cell lysate” samples.

(b) Isolation of supernatant samples. The combined 8 ml cell supernatants of two equally treated wells were harvested and mixed with 5 ml PBS pH 7.4. Cells remaining in the supernatant were then removed by centrifugation for 5 min at 150 x g after which the supernatant was filtered through a 0.22 µm pore PVDF membrane (Syringe Driven Filter Unit, Millex-GV). Then the supernatants were concentrated to 300 µl by centrifugation at 5000 x g through an Amicon®Ultra-15 centrifugal filter with a 5kDa nominal molecular weight cutoff (Millipore). The retentate was used as “concentrated supernatant” sample. Except for enriched concentrations of >5 kDa proteins, this sample should also contain high levels of the detergent PLURONIC®F-68 which is contained at 0.1% as protective surfactant in the Freestyle medium (Invitrogen) and which has an average molecular weight of 8.4 kDa.

(c) Purification by nickel affinity chromatography. For isolation of polyHis-tagged sIL-15Rα molecules, 200 µl of concentrated supernatant sample was loaded on Ni-NTA spin column (Qiagen) which had been equilibrated with washing buffer (50 mM NaH_2PO_4/300 mM NaCl/20 mM imidazole, pH 8.0). After binding for 5 minutes at room temperature, the columns (with closed caps) were centrifuged for 5 min at 300 x g, followed by two washes with 500 µl washing buffer and centrifugation for 5 min at 300 x g, and finally eluted by incubation for 5 min at room temperature with 200 µl elution buffer (50 mM NaH_2PO_4/300 mM NaCl/250 mM imidazole, pH 8.0) and centrifugation for 5 min at 300 x g plus 2 min at 800 x g. The eluates were used as “concentrated supernatants after purification by Ni-column” samples.

SDS-PAGE. 14 µl volumes of the “concentrated supernatants” and “concentrated supernatants after purification by Ni-column” samples were mixed with 5 µl “4x NuPAGE®LDS Sample Buffer” (Invitrogen) and 1 µl 1 M DTT. Together with the 20 µl “cell lysates” samples they were heated for 5 min at 95 °C and then cooled on ice. Electrophoresis of the samples was performed using the NuPAGE®Bis-Tris system (Invitrogen) with the separating gels containing 12% poly-acrylamide and using MES-SDS as running buffer.

Establishment of rabbit antibodies against bovine IL-15L. Rabbit antibodies against bovine IL-15L were commercially produced by Medical & Biological Laboratories, Nagoya, Japan. In short, a polyclonal rabbit serum was established against IL-15L derived synthetic KLH-conjugated peptide CKMLENKNDGSLYTPDNL, by six times immunization of an SPF (specific pathogen free) Japanese White Rabbit. The serum was collected seven weeks after the first immunization. Affinity purification of the peptide-specific antibodies was done using a column with sepharose to which the peptides had been conjugated. The purified antibodies

were used as "anti-Bos-IL-15L". Experiments showed that the antibodies recognize denatured IL-15L, but not non-denatured IL-15L.

Western blot analysis. After electrophoretic separation by SDS-PAGE, the proteins were transferred to PVDF membranes (Immobilon-P) by using NuPaGe®Transfer Buffer (Invitrogen) and "XCell II™Blot Module" (Invitrogen). After transfer the membranes were blocked overnight in PBS/5% skim milk powder, followed by incubations with either (a) 1/200 rabbit anti IL-15L, (b) 1/200 murine "ANTI-FLAG® M2 Monoclonal Antibody" (Sigma), or (c) 1/500 murine monoclonal antibody "c-Myc (9E10): sc-40" (Santa Cruz Biotechnology) in PBS/5% skim milk powder, followed by washes with PBS/0.1% tween-20, followed by incubations with either (a) 1/1000 "HRP-Conjugated Stabilized Goat Anti-Rabbit" (Pierce) or (b) 1/1000 "ECL™ Peroxidase labeled anti-mouse antibody" (Sigma) in PBS/5% skim milk powder, followed by additional washes with PBS/0.1% tween-20. Visualization of bound antibody was done by chemiluminescence using "Pierce® Western Blotting Substrate Plus" kit (Pierce) and "ImageQuant™ LAS 4000 mini" equipment (GE Healthcare).

Reference in Text S1.1:

[1] Dubois S, et al. (1999) Natural splicing of exon 2 of human interleukin-15 receptor alpha-chain mRNA results in a shortened form with a distinct pattern of expression. *J Biol Chem* **274:** 26978-26984.

Text S1.2

A hypothesis on the evolution of binding by IL-2/15/15L family cytokines to IL-2Rα/15Rα family receptor chains.

Because IL-2Rα and IL-15Rα are encoded by tandemly duplicated genes [reference 1], we hypothesize that in their early evolution each of the cytokines IL-2, IL-15 and IL-15L could bind both receptor chains. In teleost fish IL-2Rα probably was lost (Fig. S1*C*-to-*F*), and in the mammalian line the IL-2·IL-2Rα interface differentiated from family consensus leading to different receptor specificities of IL-2 versus IL-15 plus IL-15L. Some mammals kept IL-15L, whereas in others it was lost.

A more detailed evolutionary hypothesis is difficult to make, but should probably consider the following observations and considerations: (i) IL-2 secreted by activated T cells stimulates those very cells by autocrine loop [2], which in a way is reminiscent of co-expression of complexes observed for IL-15 and IL-15Rα; (ii) Although IL-2 and IL-15 predominantly function with their sushi domain receptor chain provided "in cis" and "in trans", respectively, both cytokines can function with their receptor chain in either fashion [3, 4]; (iii) Also in bony fish, as found in mammals, the binding affinity of IL-2 for its sushi domain containing receptor chain (in bony fish that is IL-15Rα) is lower than found for IL-15 [5]; (iv) IL-2 and IL-15 are very potent cytokines leading to potential toxicity [6], and the high affinity interaction of an IL-2/15/15L ancestral molecule with an IL-2Rα/15Rα ancestral molecule probably was established to optimize control over cytokine localization.

Based on the above considerations, and on the distribution of the binding motif for IL-15Rα throughout the IL-2/15/15L family, we speculate that the IL-2/15/15L ancestral molecule bound the IL-2Rα/15Rα ancestral molecule with high affinity as does extant mammalian IL-15 and that the system could act both in cis and in trans. After IL-2, IL-15 and IL-15L separated in evolution, for IL-2 the in cis presentation mode may have been optimized

which necessitated that the free IL-2 molecule became more stable and that its high affinity for IL-15Rα decreased. A feedback system with Treg cells, expressing highest amounts of the sushi domain receptor chain for IL-2 (IL-15Rα at that time), may then have been established to protect from possible toxicity of IL-2-induced immune stimulation. After IL-2 was established in evolution, it may have acquired its own receptor IL-2Rα by modification of an *IL-15Rα* duplicate gene and by optimizing properties such as in cis presentation, relatively low affinity, and feedback regulation by Treg. Since bony fish probably lost IL-2Rα, we speculate that they may have returned to a here hypothesized ancient stage in which the division between properties of IL-2 and IL-15 were similar as in mammals, but with both cytokines binding to the same sushi domain containing receptor IL-15Rα. It will be very interesting to investigate, for example, whether bony fish IL-2 predominantly interacts with IL-15Rα in the in cis configuration while bony fish IL-15 may function with IL-15Rα provided in trans.

References in Text S1.2:

[1] Anderson D-M, et al. (1995) Functional characterization of the human interleukin-15 receptor alpha chain and close linkage of IL15RA and IL2RA genes. *J Biol Chem* 270(50):29862-29869.

[2] Busse D, et al. (2010) Competing feedback loops shape IL-2 signaling between helper and regulatory T lymphocytes in cellular microenvironments. *Proc Natl Acad Sci U S A* 107(7):3058-3063.

[3] Olsen S-K, et al. (2007) Crystal Structure of the interleukin-15.interleukin-15 receptor alpha complex: insights into trans and cis presentation. *J Biol Chem* 282(51):37191-37204.

[4] Wuest S-C, et al. (2011) A role for interleukin-2 trans-presentation in dendritic cell-mediated T cell activation in humans, as revealed by daclizumab therapy. *Nat Med* 17(5):604-609.

[5] Wen Y, Fang W, Xiang L-X, Pan R-L, Shao J-Z (2011) Identification of Treg-like cells in Tetraodon: insight into the origin of regulatory T subsets during early vertebrate evolution. *Cell Mol Life Sci* 68(15):2615-2626.

[6] Munger W, et al. (1995) Studies evaluating the antitumor activity and toxicity of interleukin-15, a new T cell growth factor: comparison with interleukin-2. *Cell Immunol* 165(2):289-293.

Text S1.3

Sequences used for d_s/d_n analysis in Table S2

The *IL-15L*, *IL-15* and *IL-2* sequences are listed below in Fasta format and for *IL-15* and *IL-2* the database accessions are provided. For database details on the *IL-15L* sequences see Fig. S2.

<u>The investigated *IL-15L* sequences:</u>

```
>Bos_(cattle)_IL-15L
ATGTGGCTTCTCTGGACCACCCTCCTGCTGGTGCTGCCCTTGGGAGGCCTAGGACCACTC
CTCTGCCCAAGGGAGCCTTTCTACTTCCTCATTGCCATCACGAAGATGCTGGAAAACAAA
AATGATGGCAGTCTGTACACCCCAGATAATCTATTGGTGTGTCCTGCTGAGACTCTCCGA
TGCTTCCGGCTGGAGTTGTCTGTGATCGGGTTTGAGGAGGGCCCATCCGTGGGGATCGTT
GTGTTCCGCCTACAGCGCCTACTGGATGCCCTGGGGTCCCAGCTGTGGGTGATTGATCAG
GGCCCTTGTCCACCCTGCGAAGGACACCCTCAGAGACCAGTCCCTCTTTTTCTGGCCAAA
CTCTTGGAGTTATTACAGGGGACTTGTGCTCGGGACCTGCCCTCAGCATAA
>Ovis_(sheep)_IL-15L
ATGTGGCTTCTCTGGACCACCCTCCTGCTGGTGCTGCCCTTGGGAGGCCTAGGACCACCC
CTCTGCCCGAGGGAGCCTTTCTACTTCCTCGTTGCCATCACGAAGATGCTGGAAAACAAA
AATGATGGCAGTCTCTACACCCCAGATAATCTATTGGTGTGTCCTGCTGAGACTCTCCGT
TGCTTCCGGCTGGAGCTGTCTGTGATTGGGTTTGAGGAGGGCCCATCCGTGGGGATTGTT
GTGTTCCGCCTACAGCGCCTACTGGATACCCTGGGGTCCCGGCTGTGGGTGACTGGTCAG
GGCCCTTGTCCACCCTGTGAAGGACACCCTCAGAGACCAGTCCCTCTTTTTCTGGCCAAA
CTCTTGGAGTTATTACAGGGGACTTGTGCTCAGGACCTGCCCTCAGCATAA
>Sus_(pig)_ IL-15L
ATGTGGCATCTCTGGACCATCCTCCTGCTGGTGCCACCCTTGGGGGGCCTAGGACCACCC
CTCTGCCCCCGGGAGCCCTTCTACTTCCTCATTGCCATCATGAAGATGCTGGGCAACAAA
AATGATGGCACCCTCTACACCCCAGATGATCTTTCGGTGTGTCCTGCCGAGACTCTAGGC
TGCTTCCGGCTGGAGCTGTCTGTGATTGGGTTCGAGGAGGGCCCATTGGTGGGGACTGCT
GTGTTCCGGCTGCAGCGCTTACTGGATGCCCTGGGGTCCCGGCTGTGGGTGACTGGCCAG
GGCCCTTGTCCACCCTGTGAAGGACACCCCCAGAGACCTGTCCCTCTCTTTCTGGCCAAA
CTCTTGGAGTTATTACAGGGGGCTTGTGCTCGGCACCTGGCCTCGGCTTGA
>Equus_(horse)_ IL-15L
ATATGGCCTCTCTGGACCATTGTCCTGCTGATGCGGCCTTTGGGGGGCCTAGGACCACCC
CTCTGCCCTCGGGAGCCTTTCTACTTCCTTCTTGCCATCATGAAGATGCTGGGAAACAAA
AATGATGGCACTCTCTACACCCCAGATGATTTCTCGGTGTGTCCTGCGGAGACGCTAGGC
TGCTTTCGGCTGGAGCTGTCTGTGATAGGGTTCGAAGAGGGCCCATCTGTGGAAATTGCT
GTGTTCCGACTACAGCGTCTGTTGGATGCCCTGGGGTCCCGGCTGTGGGGGACTGGCCCG
GGCCCTTGTCCACCCTGTGAAGGACATCCCCAGAGACCTGTCCCTCTCTTTCTGGCCAAA
CTCTTGGAGTTATTACAGGGGGCTTGTGCTCGGCACCTGCCCTCAGCATGA
>Ceratotherium_(rhinoceros)_IL-15L
ATGGGCAGGGTGTCCATATGGCCTCTCTGGACCATCCTCCTGCTGATGTGGCCTTTGGGG
GGCCTAGGACCACCCCTCTGCCCTCGGGAGCCTTTCTACTTCCTCGTTGCCCTCATGAAG
ATGCTGGGAAACAAAAATGATGGCACTCTCTACACCCCAGATGATTTCTCGGTGTGTCCT
GCGGAGACTCTAGGCTGCTTCCGGCTGGAGCTGTCTGTGATAGGGTTGGAGGAGGGCCCA
TCTGTGGGGACTGCTGTGTTCCGGCTACAGCGTCTGCTGGATGCCCTGGGGTCCCGGCTG
TGGGGGACTGGCCAGGGCCCTTGCCCACCCTGCGAAGGACATCCCCAGAGACCTGTCCCT
CTCTTTCTGGCCAAACTCTTGGAGTTATTACAGGGGGCTTGTGCTCGGCGCCTGCCCTCA
ACATGA
>Felix_(cat)_ IL-15L
ATGTGGCCTCTCTGGACCATCCTTCTGCTGGTACGGCCCTTGGGAGGCCTAGGATCACCC
CTCTGCCCTCGGGAGCCTTTCTACTTCCTTGTTGCCATCATGAAGATGCTGGGAAACAAA
AATGATGGCACTCTCTACACCCCTGATGATCTCTCGGTTTGTCCTGCTGAGACTCTGGGA
TGCTTCCGGCTGGAGCTTTCTGTGATCCAGTTCGAAGAGGGCCGATCCATGGGGATTGCT
```

GTGTTCCGGCTACAGCGTTTGCTGGATGCATTGGGGTCCCGGCTGTGGGTGACCGGCCAG
GGTCCTTGTCCACCCTGTGAAGGACATCCCCAGAGACCCGTGCCCCTCTTTCTGGCCAAG
CTCTTGGAGTTATTACAGGGGGCTTGTGCTAGGCACCTGCCCTCAGCATGA
>Mustela_(ferret)_ IL-15L
ATGTGGTCACTCTGGACCATCCTCCTGCTGGTACGACCCTTGGGAGGCCTAGGATCACCC
CTCTGTCCTCGGGAGCCTTTCTACTTCCTTGTTGCCATCATGAAGATGCTGGGAAACAAA
AATGATGGCACTCTCTACACACCAGATGACCTCTCGGTGTGTCCTGCTGAGACTCTAGGG
TGCTTCCGGCTGGAGCTGTCTGTGATCCAGTTCGAAGAGGGCCCATCCCTGGGGATTGCC
GTGTTCCGGCTACAGCGTCTGCTGGATGCACTGGGGTCCCGGCTGTGGGTGACTGGCCAG
GGCCCTTGTCCACCCTGCGAAGGACATCCCCAGAGACCTGTCCCTCTCTTTCTGGCCAAG
CTCTTGGAGTTATTACAGGGGGCATGTGCTCGGCACCGGCCCTCAGCATGA
>Erinaceus_(hedgehog)_IL-15L
ATGTGGGCTCTCTGGGCCATTCTCCTGCTAGTGTGGCCTTCAGGGGGCCTAGGATCCCCT
CTCTGCCCAAAGGAGCCTTTCTACTTCCTCGTGGCCCTCATGAAGATGCTGGGAAACAAA
AATTACGGCACTCTGTACACCCCAGATGACCTCTCGGTGTGTCCTGCAGAGACGCTGGGT
TGCTTCCAGCTGGAGCTGTCTGTAATTGTGTTTGAGGAGGACCCGACTGTGGTGATTGAT
GTGTTCCGGTTGCAACGCCTACTGGGTGCCCTGGGCTCTCGGCTGTGGGCCACTGACCAG
GGCCCTTGTCCACCCTGCGAAGGACATACCCAAAGGCCTGTCCATCACTTTCTGAGCAAA
CTCTTGGAGCTGTTACAGAGGGCTTGTATGCGGCACCTATTTTCACCATGA
>Sorex_(shrew)_IL-15L
ATGTGGGCTCTCTGGACCATCCTCCTGCTGGTGTGGCCCTTGAGGGTTCTCGGATCATCC
CTCTGCCCAAAAGAGCCTTTCTACTTCCTCCTCGCCATCATGAAGAAGCTGGAAAAAAAG
AATGATGGCACGCTCTATACCCCAAATGATTTTTCGGTGTGTCCTGCTGAGACGCTGGGC
TGCTTCCGGCTGGAGCTGTCTGTGATTGCATTTGAAGAGGGCCCCTCCGTGGCACTAGCA
GTGTTCCGCCTCCAGCGCCTGCTGGAATCTCTCGGGTCCCGGCTATGGGAGACTGACCAG
GGCCCTTGTTTGCCCTGTGAGGGACACCCCCAGAGACCTGTCCCACATTTTCTGGCCAAA
CTCTTGGAGTTATTACAGGGGGCATGTGCACACAACCTGCTTCTAGCATGA
>Microcebus_(lemur)_IL-15L
ATGTGGCCTCTCTGGACCATCCTCCTGCTCATGCAGCTCTGGGGGAGCCTAGGAGCCCCC
CTCTGCCGGAGGGAGCCTTTTTATTTCCTTGTGGCTATCATGAAGATGCTGGGAAACAAA
AATGACGGCACTCTCTACACCCCAGATGATCTCTCGGTGTGTCCTGCTGAGACTCTAGGC
TGCTTCAGGCTGGAGCTGTCTGTGATTGGGTTTGAGGAGGGCCCATCTGTGGGGACTGCT
GTGTTCCGGCTACAGCGCCTGCTGGATGCCCTGGGGTCCCGGCTGTGGGTGGCCAGCCAG
GGGCCTTGCCTGCCTTGTGAAGGACATCCCCAGAGACCTGTCCCCCGCTTCCTGGCCAAA
CTCTTGGAGTTATTACAGGGAGCTTGTGCTCGGCACCTTCGCACGGCATGA
>Oryctolagus_(rabbit)_IL-15L ORF
ATGTGGCCTCTCTGGACCATCCTCCTGCTTGGGGGACTCTTGGGGGGCCTAGGAACCCCT
CTCTGCCGAAGGGAACCCTTTTActtccttgtggccatcatgaagatacTGgGAGACAAA
AATGATGGCACTCTGTATACCCCAGATGATCTCTCGgTGTGTCCTGCGGAGACCCTAGGC
TGcttcaggctggagctggctGTGATTGGGTTTGAGGAGGGCCCATCTGTGGgaattgct
gtgttccggctacagcgcctgctggatGCCTTGGGGTCCCGGCTGTGGGTAGCCAGCCAG
GGGCCCTGCCTGCCCTGCGAAGGACATCCCCAGAGACCCGTCCCCCTCTTTCTGGCCAAA
CTGTTGGAATTGTTACAGGGGGCTTGTGCTCAGCACCTTTCCACAGCATAa
>Ochotona_(pika)_IL-15L
ATGTGGCCTCTCTGGACCATCCTCCTGCTTGGGGGGCTCTTGGGAGGTCTGGGAACCCCC
CTCTGCCGGAGAGAACCATTTTATTTCCTCTTGGCTATCATGAAGATGTTGGGAGACCAA
AGTGATGGCACCCTGTACACTCCAGGGGATCTCTCGGTGTGTCCTGTGGAGAGCCTGGGC
TGCTTCCGGCTGGAGCTGGCTGTGATCGGGTTTGAGGAGGGCCCATCTGTGGGAATGGCT
GTGTTCCGGCTGCAGCGCCTGCTAGATGCCTTGGGGGCCCGGTTGTGGGTGGCCAGCCAG
GGTCCCTGCCTGCCTTGCGAAGGACACCCCCAGAGACCGGTTCCCCTCTTTCTGGCCAAA
CTCTTGGAGTTGTTGCAGGGGGCATGTGCTCAACAAACATTCACAGCATAA
>Procavia_(hyrax)_ IL-15L
ATGTGGCCTCTCTGGATCATCCTTCTGCTGGTGCCACCCTTGTGGGGCCTGGGGCCTCCC
CTCTGCTCTCAGGAGCCTTTTAAATTTCTGGTTGCTATCAAGAAGATTCTGGGAAACAGA
AATGACGGCACGCTGTACACCCCAGATGATCTCTCAGTGTGTTCGGTTGAGACACTAGGC
TGCTTCAGGCTGGAATTGTCTGTGATTGGGTTTGAGGAAGGTCCATCTGTGGGGATTGTT
GTGTTGTGGCTGCAGCGACTGCTGGATGTTCTGGGTTCCCAGCTGTGGGTGGCCGGCCAG
GGCCCTTGTCCACCCTGTGAAAGACATCCCCAGAGACCTGTCCCCTTCTTTCTGGCCAAG
CTCTTGGAGTTATTACAATGGGCATGTACTGGACATTTGTCAAGAGCATGA

The investigated *IL-15* sequences:

Bos (cattle) *IL-15*: GenBank NM_174090
Ovis (sheep) *IL-15*: GenBank NM_001009734
Sus (pig) *IL-15*: GenBank DQ658181
Equus (horse) *IL-15*: GenBank AY682849
Ceratotherium (rhinoceros) *IL-15*: Ensembl "CerSimSim1"
Felis (cat) *IL-15*: GenBank NM_001009207
Mustela (ferret) *IL-15*: Ensmbl "MusPutFur1.0"
Sorex (shrew) *IL-15*: Ensembl "SorAra2.0"
Microcebus (lemur) *IL-15*: Ensembl "micMur1"
Oryctolagus (rabbit) *IL-15*: GenBank NM_001082216
Ochotona (pika) *IL-15*: Ensembl "OchPri3"
Procavia (hyrax) *IL-15*: Ensembl "proCap1"

```
>Bos_(cattle)_IL-15
ATGAGAATTTTGAAACCATATTTGAGAAGTACTTCCATCCAGTGCTACTTGTGTTTACTT
CTGAACAGTCATTTTTTAACAGAGGCTGGCATTCATGTCTTCATTTTGGGCTGTATCAGT
GCAAGTCTTCCCAAAACAGAAGCAAACTGGCAGTATGTAATAAATGATTTGAAAACAATT
GAGCATCTTATTCAATCTATACATATGGATGCCACTTTATATACTGAAAGTGATGCTCAT
CCCAATTGCAAAGTAACAGCGATGCAGTGCTTTCTCCTGGAGTTACGAGTTATTTTACAC
GAGTCCAAAAATGCCACCATTTATGAAATAATAGAAAATCTTACCATGCTAGCAAACAGC
AATTTATCTTCTATTGAGAATAAAACAGAATTGGGATGCAAAGAATGTGAGGAACTGGAG
GAAAAAAGTATCAAAGAATTTTTGAAGAGTTTTGTACATATTGTGCAAATGTTCATCAAC
ACTTCTTGA
>Ovis_(sheep)_IL-15
ATGAGAATTTTGAAACCATATTTGAGAAGTACTTCCATCCAATGCTACTTGTGTTTACTT
CTGAACAGTCATTTTTTAACAGAGGCTGGCATTCATGTCTTCATTTTGGGCTGTATCAGT
GCAGGTCTTCCCAAAACAGAAGCAAACTGGCAGTCTGTAATACATGATTTGAAAACAATT
GAGCATCTTATTCAATCTATGCATATGGATGCCACTTTATATACTGAAAGTGATGCTCAT
CCCAATTGCAAGGTAACAGCGTTGCAGTGCTTTCTCCTCGAGCTACGCGTTATTTTACAC
GAGTCCAAAAATGCCGCCATTTATGAAATAATAGAAAATCTTACCATGCTAGCAGACAGA
AATTTATCTTCTATTGAGAATAAAACAGAATTGGGATGCAAAGAATGTGAGGAACTGGAG
AAAAAAAGTATCAAAGAATTTTTGAAGAGTTTTGTACATATTGTGCAAATGTTCATCAAC
ACTTCTTGA
>Sus_(pig)_IL-15
ATGAGAATTTTGAAACCATGTTTGAGAAGTACTTGCATCCAGTGCTACTTGTGTTTACTT
CTGAACAGTCATTTTTTAACTGAGGATGGCATTCATGTCTTCATTTTGGGCTGTATCAGT
GCAGGTCTTCCTAAAACAGAAGCAACCTGGCAGCACGTAATAAGTGATTTGAAAAAAATT
GAAGATCTTATTCGATCTATACATATGGATGCCACATTGTATACTGAAAGTGATGCTCAT
CCCAATTGCAAAGTAACAGCGATGAAGTGCTTTCTCCTGGAGTTACGCGTCATTTTGCAA
GAGTCCAGAAATTCAGACATTAGTGATACAGTAGAAAACCTTATCATCCTTGCAAACAGC
AGTTTATCGTCCATTGAGTATAAAACTGAATCTGGATGCAAAGAATGTGAGGAGCTGGAG
GAAAAAAATATTAACGAATTTTTGAAGAGTTTTATACATATCGTGCAAATGTTCATCAAC
CCTTCTTGA
>Equus_(horse)_IL-15
ATGAGAATTTTGAAACCATATTTGAGAAGTACTTGCATCCAGTGCTACTTGTGTTTACTT
CTGAACAGTCATTTTTTAACTGAGGCTGGCATTCATGTCTTCATTTTGGGCTGTATCAGT
GCAGGTCTTCCTAAAACAGAGGCAAACTGGCAGGATGTAATAAGTGATTTGAAAAGAATT
GAAGATCTTATTCAATCTATACATGTTGATGCCACTTTATATACTGAAAGCGATGCTCAT
CCCAGTTGCAAAGTAGCAGCGATGAAGTGCTTTCTCCTGGAGTTACATGTTATTTTGCAT
GAGTCCAGAAACGAGGACATTAAGGAAACAGTAGAAAACCTTATCATCCTAGCAAACAGC
AGCTTATCTTCTAATGGGAATGTTACAGAATCTGGATGCAAAGAGTGTGAGGAACTGGAG
GAAAAAAATATTAAAGAATTTTTGCAGAGTTTTGTACATATCGTGCAAATGTTCATCAAC
CCTTCT
>Ceratotherium_(rhinoceros)_IL-15
ATGAGAATTTCGAAACCCTATTTGAGAAGTACTTCCATCCAGTGCTACTTGTGTTTACTT
CTGAACAGTCATTTTTTAACTGAGGCTGGAATTCATGTCTTCATTTTGAGCTGTATCAGT
GCGGGTCTTCCTAAAACAGAGGCAAACTGGCAGGATGTAATAAGTGATTTGAAAAGAATT
GAAGATCTTATTCAATCTATACATATTGATGCCACTTTATATACTGAAAGTGATGCTCAT
CCCAATTGCAAAGTAACAGCGATGAAGTGCTTTCTTCTGGAGTTACGTGTTATTTCGCAT
```

GAGTCCAGAAATATGGACATAAATGAAACAGTAGAAAACCTTATCATCCTGGCAAACAGC
AGTTTATCTTCTAATGGGAATATTATAGAATCTGGATGCAAAGAATGCGAGGAACTGGAG
GAAAAAAATATTAAAGAATTTTTGCGGAGTTTTGTACATATCGTGCAAATGTTCATCAAC
CTTTCTTGA
>Felis_(cat)_IL-15
ATGAGAATTTTGAAACCATATTTGAGAAGTACTTCCATCCAGTGCTACTTGTGTTTACTT
CTGAACAGCCATTTTTTAACTGAAGCTTGCATTCCTGTTTTCATTTTGAGCTGTATTAAT
GCAGGTCTTCCTAAAACAGAGGCAAACTGGCAGGATGTAATAAGTGATTTGAAAATAATT
GACAAGATTATTCAATCCTTACATATCGATGCCACTTTATATACTGAAAGTGATGTTCAT
CCCAATTGCAAAGTAACAGCGATGAAGTGCTTTCTCCTGGAGTTACATGTTATTTCGCTT
GAGTCCAAAAATGAGACCATTCATCAAACAGTAGAAAACATTATTATCCTGGCAAACAGT
GGTTTATCTTCTAACAGGAATATAACTGAAACAGGATGCAAAGAATGTGAGGAACTGGAG
GAAAAGAACATTAAAGAATTTCTGCAGAGTTTTGTACATATTGTACAAATGTTCATCAAC
ACTTCTTGA
>Mustela_(ferret)_IL-15
ATGAGAATTTTGAAAACGTATTTGAGAAGTACTTCCATCCAATGCTACTTGTGTTTACTT
TTACATTGTCATTTTTTAACCGAGGCTGGCATTCATGTCTTCATTTTGGGCTCTATCAGT
GCAGGTCTTCCTAAAACAGAGGCGAAATGGGAGTTTGTAATAAAGGATTTGGAAAAAATT
GACAAAATTATTCAATCTATACATATTGATACCACTTTATATACTGAAAGTGATGCTCAT
CCCGGCTGCAAAGTCACAGCGATGAACTGCTTTCTCCTGGAGTTACGTGTTGTTTTGCTT
GAATTCAGTCATGCTAACCTTCTTAATGAAACAATACATAACATTATCATCCAAGCAAAC
AGTAATTTATCTTCTAATGCGAACATAACTGAAACGGGATGCAAAGAATGTGAGGAACTG
GAGGAGAAAAATATTAAAGAATTTTTCAGAGTTTTTACATATTGTACAAATTTTCCAT
AATTCTTGA
>Sorex_(shrew)_IL-15
AAACCGGGTTGGAGAGGTTCTTGCTTCCCGTACTGGTTATGTTTACTCCTGAATATTCGC
TATTCAACCCAGGCTGGCATTCATGTCTTCATTTTGGGCTGTATCAGTGTGGTTCTCCCT
AAGACAGAAGCTCACTGGCCAGATGTACTACAAGATTTGAAGCATATTGAAAAACTTATT
CAATCTATACATATTGATGCTACCTTATATACTGAAAGTAATTTTGATCGGAACTGCTCA
GTAACAGCAATGAGATGTTTTCTCCTGGAGCTCCACGTTATTTTACAAGAGTGCAAAGAT
GAGAAAATTCAAGAAAAAGTAGAAAACATTATCATACTAGCAAACGACAGTTTGTCTTCA
AGAGGGAATGTAACGGAAGGCAAATGCAAACAGTGTGAGGAGCTGGAGGAAAAGAATATA
GAGGAATTTGTTGAGAATTCAATGCAGATGGTACAATGGTTCATTGCCGGCAAATGA
>Microcebus_(lemur)_IL-15
ATGAGAATTTCGAAACCATATTTGAGAAGTACTTCCATCCAGTGCTACTTGTGTTTACTT
CTAAACAGTCATTTTTTAACAGAGGCTGGCATTCATGTCTTCATTTTGGGATGTATCAGT
GCAAGTCTTCCTAAAACAGAGGCCAGCTGGCAGCATGTAATAAGTGATCTGAAAATAATC
GAAGATCTTATTCAATCTATACATATTGATGCTACTTTATATACTGAAAGTGATGTTCAT
CC-TAATGCAAAGTAACAGCAATGAAGTGCTTTCTCCTGGAGTTACAAGTTATTTTGCAC
GAGTCCAGAAATGTGACCCTTCAACAAACGGTAGAGAACCTTATCATCCTAGCGAACAGC
GGTTTGTCTTCTAATGAGAATGTGACAGAATCCGGATGCAAAGAATGTGAGGAACTGGAG
GAAAAAAATATTAAAGAATTTTTGCAGAGTTTCGTACATATTGTACAAATGTTCATCAAC
CCTTCTTGA
>Oryctolagus_(rabbit)_IL-15
ATGAGAATTTCGAAACCGTATCTGAGAAGTACTTCCATCCAGTGCTACCTGTGTTTACTC
CTAAACAGTCATTTTTTAGCTGAGGCTGGCATTCATGTCTTCATTTTTGGCTGTATCAGT
GCAGGTCTTCCTAAAACAGAAGCCAACTGGCATGATGTCATTAGTGATCTGAAAAGAATT
GAAGATCTTATAAAATCCATCCATATTGATGCTACTTTATATACTGAGAGTGATGCTCAT
CCCAATTGCAAAGTCACAGCGATGAAGTGCTTTCTCCTGGAGTTACGAGTTATTTCTCAT
GAGTCCAGAAACATGGACATTAATGAAACAGTACAAAACCTTATCATCCTAGCAAATACC
AGTTTATCTTCTAAAGGGAATGTAACGGAATCTGGTTGCAAAGAATGTGAGGAACTGGAG
GAAAAAAATATTACTGAATTTTTGCAGAGTTTTATACATATTGTACAAATGTTCATCAAT
TCTCCTTGA
>Ochotona_(pika)_IL-15
AAACCATATTTGAGAAGTACTTCCATCCAGTGCTGCTTGTGTTTATTGCTAAACAGTCAT
TTTTTACCCGAGGCTGGCATTCCTGTCTTCTTCTTCAGCTGTATCAGTGTAGGTCTTCCT
GAAACAGAAGCCAATTGGCATGATGTAATTAGTGATTTGAAAAGAATTGAAGATCTTATA
CAGTCTACCCATATTGATGCTACTTTATATACTGAGAGTGATGCTCATCCCAATTGCAAA
GTCACAGCGATGAAGTGCTTTCTCCTGGAGCTACGAGTTATTTCACATGAGTCCCGAGAC
ATGGACATTAACGAGACGGTACAAAACCTTATCATTCTAGCCAACAGCAGTTTATCTTCT
AAAGGGAACGTAACAGAATCTGGGTGCAAAGAATGTGAGGAACTGGAAGAAAAAAGTATT
TCTGAATTTTTGCAGAGTTTCATCCATATCGTGCAAATGTTCATCAACTCTCCTTGA
>Procavia_(hyrax)_IL-15

ATGAGAATTTTGAAACCGTATTTGAGAAGTACCTCTATCCAGTGCTATTTGTGTTTGCTT
CTAAACAGTCATTTTTTAACTGAGGCTGGAATTCAAGTCTTCATTTTGGGATGCTTTAGT
GCAAGTCTTCCTAAAACAGAGGCTAACTGGCAGTATGTAATAGATGATTTGAAAAGAATT
GAACATCTTATTCAATCTATACATGTTGATGCCACTTTGTATACTGAAAGTAATGCTTCT
CCTAATTGCAAAGTGACAGCGATGAAGTGCTTCCTCCTGGAGTTACATGTTATTTCACAT
GAATCCAGGAATATGGACCTTGTTGAAACAGTAGGAAACATTATCATCCTCGCAAACAAC
AGTTTAGCCTCTGATCGGACTATAACAGAGTCTGGATGCAAAGAATGTGAGGAACTGGAG
GAGAAGAATATTAAAGAGTTTTTGCAAAGTATTTTTACATATTGTACAGATGTTCATCAA
CTCTTGA

The investigated *IL-2* sequences:

Bos (cattle) *IL-2*: GenBank EU276068
Ovis (sheep) *IL-2*: GenBank NM_001009806
Sus (pig) *IL-2*: GenBank EU249805
Equus (horse) *IL-2*: GenBank JQ432544
Ceratotherium (rhinoceros) *IL-2*: Ensembl "CerSimSim1"
Felis (cat) *IL-2*: GenBank NM_001043337
Mustela (ferret) *IL-2*: GenBank EF368206
Erinaceus (hedgehog) *IL-2*: Ensembl "HEDGEHOG"
Sorex (shrew) *IL-2*: Ensembl "SorAra2.0"
Oryctolagus (rabbit) *IL-2*: GenBank EU639406
Ochotona (pika) *IL-2*: Ensembl "OchPri3"
Procavia (hyrax) *IL-2*: Ensembl "proCap1"

>Bos_(cattle)_IL-2
ATGTACAAGATACAACTCTTGTCTTGCATTGCACTAACTCTTGCACTCGTTGCAAACGGT
GCACCTACTTCAAGCTCTACGGGGAACACAATGAAAGAAGTGAAGTCATTGCTGCTGGAT
TTACAGTTGCTTTTGGAGAAAGTTAAAAATCCTGAGAACCTCAAGCTCTCCAGGATGCAT
ACATTTGACTTTTACGTGCCCAAGGTTAACGCTACAGAATTGAAACATCTTAAGTGTTTA
CTAGAAGAACTCAAACTTCTAGAGGAAGTGCTAAATTTAGCTCCAAGCAAAAACCTGAAC
CCCAGAGAGATCAAGGATTCAATGGACAATATCAAGAGAATCGTTTTGGAACTACAGGGA
TCTGAAACAAGATTCACATGTGAATATGATGATGCAACAGTAAACGCTGTAGAATTTCTG
AACAAATGGATTACCTTTTGTCAAAGCATCTACTCAACAATGACTTGA
>Ovis_(sheep)_IL-2
ATGTACAAGATACAACCCTTGTCTTGCATTGCACTAACTCTTGCACTCGTTGCAAACGGT
GCACCTACTTCAAGCTCTACGGGGAACACAATGAAAGAAGTGAAGTCATTGCTGCTAGAT
TTACAGTTGCTTTTGGAGAAAGTTAAAAATCCCGAGAACCTCAAGCTCTCCAGGATGCAT
ACATTTAACTTCTACATGCCCAAGGTTAACGCTACAGAATTGAAACATCTTAAGTGTTTA
CTAGAAGAACTCAAACTTCTAGAGGAAGTGCTAGATTTAGCTCCAAGCAAAAACCTGAAC
ACCAGAGAGATCAAGGATTCAATGGACAATATCAAGAGAATAGTTTTGGAACTACAGGGA
TCTGAAACAAGATTCACATGTGAATATGATGATGCGACAGTAAAGGCTGTAGAATTTCTG
AACAAATGGATTACCTTTTGTCAAAGCATCTACTCAACAATGACTTGA
>Sus_(pig)_IL-2
ATGTATAAGATGCAGCTCTTGTGTTGCATTGCACTAACCCTTGCACTCATGGCAAACGGT
GCACCTACTTCAAGCTCTACAAAGAACACAAAGAAACAACTGGAGCCATTGCTGCTGGAT
TTACAGTTGCTTTTGAAGGAAGTTAAGAATTACGAGAATGCTGATCTCTCCAGGATGCTC
ACATTTAAATTTTACATGCCCAAGCAGGCTACAGAATTGAAACACCTTCAGTGTTTAGTA
GAAGAACTCAAAGCTCTGGAGGGAGTGCTAAATTTAGGTCAAAGCAAAAACTCTGACTCA
GCAAATATCAAGGAATCAATGAACAATATCAACGTAACAGTTTTGGAACTAAAGGGATCT
GAAACAAGTTTCAAATGTGAATATGATGATGAGACAGTAACTGCTGTTGAATTTCTGAAC
AAATGGATTACCTTTTGTCAAAGCATCTACTCAACACTGACTTGA
>Equus_(horse)_IL-2
ATGTACAAGATGCAACTCTTGGCTTGCATCGCACTAACTCTTGCAGTCCTTGCAAACAGT
GCACCTACTTCAAGCTCTAAGAGGGAAACACAGCAACAACTGAAGCAATTACAGATGGAT
TTAAAGTTGCTTTTGGAAGGAGTTAATAATAACAAGAATCCCAAACTCTCCAAGATGCTC
ACATTTAAAATTAACATGCCCAAGAAGGCCACAGAATTGAAACATCTTCAGTGTCTAGAA
GAAGAACTCAAACCTCTGGAGGAAATGCTAAAAAACTTTCTCTCGAAAGATATCAAGGAA
TTAATGAGCAATATCAATGTAACAGTTCTGGGACTAAAGGGGTCTGAAACAAGATTCACA

TGTGAATATGATGATGAGACAGGAACAATTGTAGAATTTCTGAACAAATGGATTACCTTT
TGTCAAAGCATCTTCTCAACAATGACTTGA
>Ceratotherium_(rhinoceros)_IL-2
ATGTACAAGATGCAACTCTTGTCTTGCATTGTACTGACTCTTGCACTCCTTGCAAACAGT
GCACCTACCTCCAGCTCTAAGAGGGAAACACAGCAACAGCTGGAGCAATTACAGCTGGAC
TTACGGCTGCTTTTGAGTGGAGTTAATAATTACAAGAACACCAAACTCTCCAACAAGATG
CTCACATTTAAATTCAACATGCCCAAGAAGGCCACAGAATTGAAACATCTTCAGTGTCTA
GAAGAAGAACTCAAACCTCTGGAGGATGTGCTAAAAAACTTTCACTTGAAAGATATCAAG
GAATTAATGAGCAATATCAATGCAACAGTTCTGGAACTAAAGGGATCTGAAACAAGATTC
ACATGTGAATATGATGATGAGACAGCAACCATTGTAGAATTTCTGAACAAATGGATTACC
TTTTGTCAAAGCATCTTCTCAACACTGAGCTGA
>Felis_(cat)_IL-2
ATGTACAAAATTCAACTCTTGTCTTGCATTGCACTGACTCTTATACTCGTCACAAACAGT
GCACCTGCTTCAAGCTCTACAAAGGAAACACAGCAACAGCTGGAGCAATTACTGCTGGAT
TTACGGTTGCTTTTGAATGGAGTTAATAATCCTGAGAACCCCAAACTCTCCAGGATGCTC
ACATTTAAATTTTACGTGCCCAAGAAGGCCACAGAATTGACACATCTTCAGTGTCTAGTA
GAAGAACTCAAACCTCTGGAGGAAGTGCTATATTTAGCTCAAAGCAAAAACTTTCACTTG
AATCACATCAAGGAATTAATGAGCAATATCAATGTAACAGTTCTGAAACTAAAGGGATCT
GAAACAAGATTCACATGTAACTATGATGATGAGACAGCAACCATTGTAGAATTTCTGAAC
AAATGGATTACCTTTTGTCAAAGCATCTTCTCAACGCTGACTTGA
>Mustela_(ferret)_IL-2
ATGTACAAAATGCAACTCTTGTCTTGCATCGTACTGACTCTCGCACTCTTCGCAAACAGT
GCACCTACTACTTCAAGCTCTACGAAGGAAGCACAGCAACAGCTGGAGCAATTGCTGCTG
GACTTACAGTTGCTCTTGAATGGAGTTAAAAATTATGAGAGCCCCAGGATGCTCACGTTT
AAATTCTACATGCCCAAGAAGGCCACAGAATTGACTCATCTTCAATGTCTAGCAGAAGAA
CTCAAACTTTTGGAGGAAGTGCTATATTTAGCTCAAAGCAAAAACTTTCACTTGACAGAC
ATCAAGGAACTAATGAGCAATATCAATGTAACACTTCTGAAACTAAAGGGATCTGAAACA
AGTTTCAAATGTGAATATGATGACGAGACAGTAACCATTACAGAATTTCTGAACAAATGG
ATTACTTTTTGTCAAAGCATCTTCTCAACACTGACTTGA
>Erinaceus_(hedgehog)_IL-2
ATGTGCAAGGTACAGCTCCTGGTCTGCTCGGTGCTCGCGCTGGCCCTGCTGGCCTGCAGC
GTACCCACCGCCCGCCTGGCCCGGGACTCGGAGCAGCACCTGGAGCAGCTGCTGCTGGAC
CTGAAGAAGCTGAAGCTGGGCGTCGAGAATCACAAGGGCTCCACACTCGCCACGATGCTC
AGATTCCCCTTCTACCTGCCCAAGGAGGCCACAGAACTGAAGCACTTCCAGTGTCTGGTA
GGAGAACTCAAACCTCTGGGGGAAGTGCTGTCCCTGGCTGAGTGCAGACACATCAGCGAG
CTGATGAGCAACATCAACGCGACTGTCCTGGAGCTGAAGGGGTCTGGGCCCACACTCCCC
TGCGACTACGAGGAGGAGGCAGTGAGCGTCCTGCAGCTGCTGGCCAAGTGGATCAGCATC
TGTCAGAGCATCTACTCACGGCTGACCTGA
>Sorex_(shrew)_IL-2
ATGCACAGGCTGCAGCTCCTGGCCTGTGCCACGCTCACGCTCGCACTCCTCGCCACCAGT
GCACCCACCTCCAGGTCGACCCAGGCGGCACAGCAGCAGCTGGAGCACTTGCTGCTGGAC
TTACAGCTCCTTCAGAAAGGAGTGGAGAATTACAAGAACCTCAGAGTCCCCAGGATGTTT
AAATTTTACTTGCCCAAAAAGGCCACTGAACTGAAGCATCTTCAGTGTCTAGCAGAAGAA
CTCACATCTTTGGAGGAAGTACTAAATCTAGCTCAAGGCAAGAATGCAGTTATCAAGGAC
TTAATGAGCAACATCAATGTGACAGTCCTGGAACTAAAGGGTTCAGAAACAAGCTCCGCC
TGTGATTATGAGGATGAGCCAGTGAGCCTCACAGACTTTCTCACCAGATGGATCACCTCT
TGTCAAGGCATCTTCTCAACACTCGCTTGA
>Oryctolagus_(rabbit)_IL-2
ATGTACAAAGTACAACTCTTGTCTTGCATTGCACTAACTCCTGCACTCCTTACAAGCAGT
GCACCTACTCCAAGTTCTACAAAGGAAACACAGGAACAACTGGATCAGCTACTGCTGGAT
TTACAGGTGCTTTTGAAAGGAGTTAATGATTATAAGAATTCCAAACTTTCCAGGATGCTC
ACATTCAAATTTTATATGCCCAAGAAGGTCACAGAGTTGAAACATCTTCAGTGTCTAGAA
GAAGAACTCAAACCTCTGGAGGAAGTGCTTAACTTAGCTCAAGGCAAAAACTCTCATGGG
GGAAATACCAGAGAATCAATCAGCAATATTAATGTAACAGTTCTGAAACTAAAGGGATCT
GAAACATTCATGTGTGAATACGATGAGACAGTTACCATTGTAGAATTTCTGAACAGATGG
ATCACCTTTTGTCAAAGCATCATCTCAGCATCGAGTTCATAA
>Ochotona_(pika)_IL-2
ATGTTAAAGGTGCAACTTTTGTCTTGCCTTGCACTAACTCTTGTACTCCTCACAAACAGT
GCACCTACTTCAAGTTCTACAAAGGAAACACAGGAACAACTCGGTCAACTACTGCTGGAT
TTACAGGTGCTTTTGAAAGGAGTTAATGATTACAAGAATTCCAAACTTTCAAGGATGCTA
ACATTTAAATTTTATATGCCTAAAAAGGTCACAGAACTGGAACATCTTCAGTGTCTAGAA
GAAGAACTCAAACCTCTGGAAGAAGTGCTTAGCTTAGATCAAAGCAAAAACTCTCATTTG
AGAAATATTAGAGAATTAATCAGCAACATCAATGTGACAGTTCTGAAACTAAAGGATCT

GAAACGACATCCAGATTTGAGTGTGATGATGAGACAGTCACTGTGGTAGAACTCCTGAAC
AGATGGATCACTTTTTGTCAAAGTATCATCTCAACACTGGTTCATAATTAA
>Procavia_(hyrax)_IL-2
ATGTACAAGGTGCAACTCTTGTCTTGCATTGCACTAACTCTTGCACTCATCACAAACAGT
GCACCTACTTCAAACTCTACAAAGGCAACACAGCAACAACTGGAACAATTACTGCGGGAT
TTACAGATGCTTTTGACTAGAGTTAAAAATTATGAGACTCCCAGACTCTCCATGATGTTC
ACATTTAAATTCTACACACCTAAGACGGTCACAGAATTGAAACATCTTCAGTGTCTAGTA
GATGAACTCAAACCTCTGGAGTATGTGCTCAATGTAGCTCCCAGCAAAATAAGTACCAGG
GACTTAATCAGCAACATCAATGTAACGGCCCTGGAACTACAGGGATCTGAAACAACATTC
ATGTGTGAATACGATGAGAAGGCAGTGACCATTGAAGAATTCCTGGACAAATGGATTGTC
TTTTGTCAAAGCATCATCGCCAATACTGACTTGAGAGTGTTTCCCATTTAA
