# Supplementary material for: Identification of a gene for an ancient cytokine, interleukin 15-like, in mammals; interleukins 2 and 15 co-evolved with this third family member, all sharing binding motifs for IL-15Rα
Source: Immunogenetics. 2013 Nov 26;66(2):93–103. doi: 10.1007/s00251-013-0747-0 (PMC3894449; doi:10.1007/s00251-013-0747-0)

Supplementary Figure 1 (Fig. S1).
Genomic organization of *IL-15L*, *IL-15*, *IL-2*, *IL-15Rα* and *IL-2Rα* genes.
Phylogenetic tree with IL-15Rα and IL-2Rα.

Table of Contents:

**Legends to Figures S1*A*-to-*F***		Page 2
**Fig. S1*A***	Genomic locations of *IL-15L* and related cytokine genes in representative species	Page 8
**Fig. S1*B***	*IL-15L* intron-exon organization	Page 9
**Fig. S1*C***	Genomic locations of *IL-15Rα* and *IL-2Rα* in representative species	Page 10
**Fig. S1*D***	Intron-exon organization of *IL-15Rα* and *IL-2Rα* In representative species	Page 11
**Fig. S1*E***	Deduced amino acid sequences encoded by the genes shown in Fig. S1*D*	Page 12
**Fig. S1*F***	Phylogenetic tree inferred by NJ method for the IL-15Rα and IL-2Rα sushi domain sequences compared in main text Fig. 5	Page 13

Legends to Figures S1*A*-to-*F*

General: The Ensembl databases used to retrieve the genomic sequence information described in Figs. S1A-to-F were as follows (for full English and Latin names of species see main text Fig. 2): Pufferfish, TETRAODON8; stickleback, BROADS1; zebrafish, Zv9; gar, LepOcu1; frog, JGI_4.2; turtle, ChrPicBel3.0.1; lizard, AnoCar2.0; chicken, Galgal4; platypus, OANA5; opossum, BROADO5; cattle, UMB 3.1; lemur, micMur1; human, GRCh37.

Fig. S1*A* Genomic locations of *IL-15L* and related cytokine genes in representative species. The *IL-15L* locus (a) is not situated on the same chromosome as *IL-2*, *IL-21* (b) and *IL-15* (c), but all four loci are rather well conserved throughout various vertebrates. Cytokine genes are in red. Numbers behind genes indicate positions in megabase on the chromosome or scaffold indicated above the figures, according to the Ensembl database. Asterisks indicate that information for the complete gene is not available or that a full-length prediction is difficult to make. (a) *PLEKHG2*, *pleckstrin homology domain-containing family G member 2*; *SUPT5H*, *suppressor of Ty 5 homolog*; *TRIAP1*, *TP53 regulated inhibitor of apoptosis 1*; *CCDC61*, *coiled-coil domain containing 61*; *VASP, vasodilator-stimulated phosphoprotein*; *RPS16*, *ribosomal protein S16*. (b) For green pufferfish (Tetraodon) there is no intact sequence of the *TENR*-to-*BBS12* genomic region in the database and therefore the region sequence of another neoteleost fish, stickleback, is depicted. Although highly diverged from *IL-2*, the neoteleost specific *IL-2L* gene probably is a rather recent duplication of *IL-2* because both genes in teleost fish encode

the same unique cysteine pair (see Fig. S6). *TENR*, *Testis Nuclear RNA-Binding Protein*; *BBS12*, *Bardet-Biedl syndrome 12*. (c) *ZNF330*, *Zinc Finger Protein 330*; *INPP4B*, i*nositol polyphosphate-4-phosphatase*, *type II*.

Fig. S1*B* *IL-15L* intron-exon organization. The figures are based on analyses of Ensembl database genomic sequences (see also Fig. S2), and for zebrafish the depicted *IL-15L* intron-exon organization was confirmed by cDNA analysis [reference 1]. Fish *IL-15L* ORF comprises four exons, all in phase 0, which is common among most short-chain helical cytokines. In tetrapod evolution, before the split between the species lineages leading to extant reptiles and mammals, the intron between exons 3 and 4 was lost (see also Fig. S2). After the ancestors of humans and lemurs separated, within the human lineage the *IL-15L* ORF acquired a number of incapacitation motifs, here indicated by colored arrows and their descriptions (for details see Fig. S2). X, these exons do not encode canonical IL-15L domains and probably were incapacitated for coding of functional protein.

Reference in this figure legend:

[1] Gunimaladevi I, Savan R, Sato K, Yamaguchi R, Sakai M (2007) Characterization of an interleukin-15 like (IL-15L) gene from zebrafish (Danio rerio). *Fish Shellfish Immunol* 22(4):351-362.

Fig. S1*C* Genomic locations of *IL-15R*α and *IL-2R*α in representative species. The receptor chain genes are in red. Numbers behind genes indicate positions in megabase on the chromosome or scaffold indicated above the figures, according to the Ensembl

database. Double lines indicate presence of additional genes which are not shown. Our analysis agrees with that of Wen et al. [reference 1], on that bony fish only have a single *IL-2Rα/15Rα*-family gene. Based on Figs. S1*D* –to -F, this bony fish gene can be classified as *IL-15Rα*, which also agrees with the phylogenetic tree analysis by Fang et al. [2].

References in this figure legend:

[1] Wen Y, Fang W, Xiang L-X, Pan R-L, Shao J-Z (2011) Identification of Treg-like cells in Tetraodon: insight into the origin of regulatory T subsets during early vertebrate evolution. *Cell Mol Life Sci* 68(15):2615-2626.

[2] Fang W, Xiang L-X, Shao J-Z, Wen Y, Chen S-Y (2006) Identification and characterization of an interleukin-15 homologue from *Tetraodon nigroviridis*. *Comp Biochem Physiol B Biochem Mol Biol* 143(3):335-343.

Fig. S1*D* Intron-exon organization of *IL-15Rα* and *IL-2Rα* in representative species. Coding (parts of) exons are represented by boxes, with the exon number indicated within, the nucleotide length above, and the exon phase in between. Below the exon phase number the intron length is indicated. The domain regions shown at the top of the figure (TM for transmembrane region; CY for cytoplasmic tail) roughly correspond with the exons placed below them, except for the second sushi domain in IL-2Rα in chicken and human. All depicted intron-exon organizations are based on comparison of reported cDNA sequences with Ensembl database genomic sequences, except for frog (*Xenopus tropicalis*) *IL-15Rα* which is a prediction from Ensembl database genomic sequence only (*). For both *IL-15Rα* and *IL-2Rα* transcripts in mammals multiple splicoforms are

known, most impressively so for *IL-15Rα* [e.g. references 1, 2]. In contrast, our figure shows that within evolution the *IL-15Rα* intron-exon organization seems remarkably well conserved, whereas *IL-2Rα* intron-exon organization appears poorly conserved. The frog *IL-2Rα* gene only encodes a single sushi domain and also in the surrounding genomic region no extra sushi domain exon can be found. The last exons of frog *IL-2Rα* ORF are connected in phase 2 and 0 (purple circles), which is very different from family consensus. Whether frog *IL-2Rα* represents an ancestral *IL-2Rα* structure, or a degenerated form, or even a related gene different from *IL-15Rα* and *IL-2Rα*, cannot be determined at this stage. More information on *IL-2Rα* in primitive vertebrates, including information on possible splicoform variation, would ne needed to allow a proper discussion. Comparison of deduced amino acid sequences (see Fig. S1*E*) indicate relationships between the sushi domain exons of all *IL-15Rα* and *IL-2Rα* (red), and between the exon3 sequences of the IL-15Rα genes (blue). For the other exons it is very difficult to conclude relationship from comparison of encoded sequences (Fig. S1*E*). The elephant shark *IL-15Rα* sequence is not shown here because the short sequence scaffold in accession AAVX01047089 of the Eshark 1.4X assembly of the Elephant Shark Genome Project only contains the sushi domain exon. GenBank accession numbers of the cDNA sequences that were analyzed are: Tetraodon *IL-15Rα*, EF165732; Gallus *IL-15Rα*, AI980376 and BU370374; Homo *IL-15Rα*, NM_002189; Xenopus *IL-2Rα*, EL724547; Gallus *IL-2Rα*, AY772091; Homo *IL-2Rα*, X01057.

References in this figure legend:

[1] Müller J-R, Waldmann T-A, Kruhlak M-J, Dubois S (2012) Paracrine and transpresentation functions of IL-15 are mediated by diverse splice versions of IL-15Rα in human monocytes and dendritic cells. *J Biol Chem* 287(48):40328-40338.
[2] Horiuchi S, et al. (1997) Altered interleukin-2 receptor alpha-chain is expressed in human T-cell leukaemia virus type-I-infected T-cell lines and human peripheral blood mononuclear cells of adult T-cell leukaemia patients through an alternative splicing mechanism. *Immunology* 91(1):28-34.

Fig. S1*E* Deduced amino acid sequences encoded by the genes shown in Fig. S1*D*. Sequences are organized per corresponding exon. Conserved cysteines in the sushi domains are indicated in red font. The DP motifs encoded by IL-15Rα exon3 are in blue font.

Fig. S1*F* Phylogenetic tree inferred by Neighbor joining method [reference 1] for the sushi domain sequences of Il-15Rα (yellow) and IL-2Rα (orange) as aligned in main text Fig. 5. All the IL-15Rα sequences cluster together, although mostly with low bootstrap values. Conservation of the IL-2Rα sequences was poor and even another sushi domain sequence, the first sushi (CCP) domain of human CD55 (green), was found to map within the IL-2Rα cluster.

The optimal tree with the sum of branch length = 8.81357173 is shown. The tree is drawn to scale, with branch lengths in the same units as those of the evolutionary distances used to infer the phylogenetic tree. The evolutionary distances were computed using the Poisson correction method [2] and are in the units of the number of amino acid

substitutions per site. All ambiguous positions were removed for each sequence pair. There were a total of 74 positions in the final dataset. Evolutionary analyses were conducted in MEGA5 [3]. Bootstrap values lower than 50% are not shown.

GenBank accession numbers are: human IL-15Rα, GenBank AAP69528; mouse IL-15Rα, NP_001258430; cattle IL-15Rα, XP_871812; chicken IL-15Rα, XP_414982; pufferfish IL-15Rα, ABM46912; frog IL-2Rα, XP_002931900; cattle IL-2Rα, AAI33547; mouse IL-2Rα, AAA39282 ; human IL-2Rα, AAB59535; human CD55, EAW93490. Sequences retrieved from Ensembl databases are: opossum IL-15Rα, Opossum (BROAD05) Chr.8; lizard IL-15Rα, anole Lizard (AnoCar2.0) Chr.5; frog IL-15Rα, Xenopus (JGI_4.2) GL172647; gar IL-15Rα, Spotted gar (LepOcu1) Chr.8; lizard IL-2Rα, anole Lizard (AnoCar2.0) Chr.5; platypus IL-2Rα, Platypus (OANA5) Contig13; opossum IL-2Rα, Opossum (BROAD05) Chr.8. The elephant shark IL-15Rα sequence is encoded by accession AAVX01047089 in the Eshark 1.4X assembly of the Elephant Shark Genome Project. Human

References in this figure legend:

[1] Saitou N, Nei M (1987) The neighbor-joining method: A new method for reconstructing phylogenetic trees. *MolBiol Evol* 4(4):406-425.

[2] Zuckerkandl E, Pauling L (1965). Evolutionary divergence and convergence in proteins. Edited in *Evolving Genes and Proteins* by Bryson V and Vogel H-J, pp. 97-166. Academic Press, New York.

[3] Tamura K, et al. (2011) MEGA5: Molecular Evolutionary Genetics Analysis using Maximum Likelihood, Evolutionary Distance, and Maximum Parsimony Methods. *Mol Biol Evol* 28(10):2731-2739.

Fig. S1*A*
Genomic locations of *IL-15L* and related cytokine genes in representative species

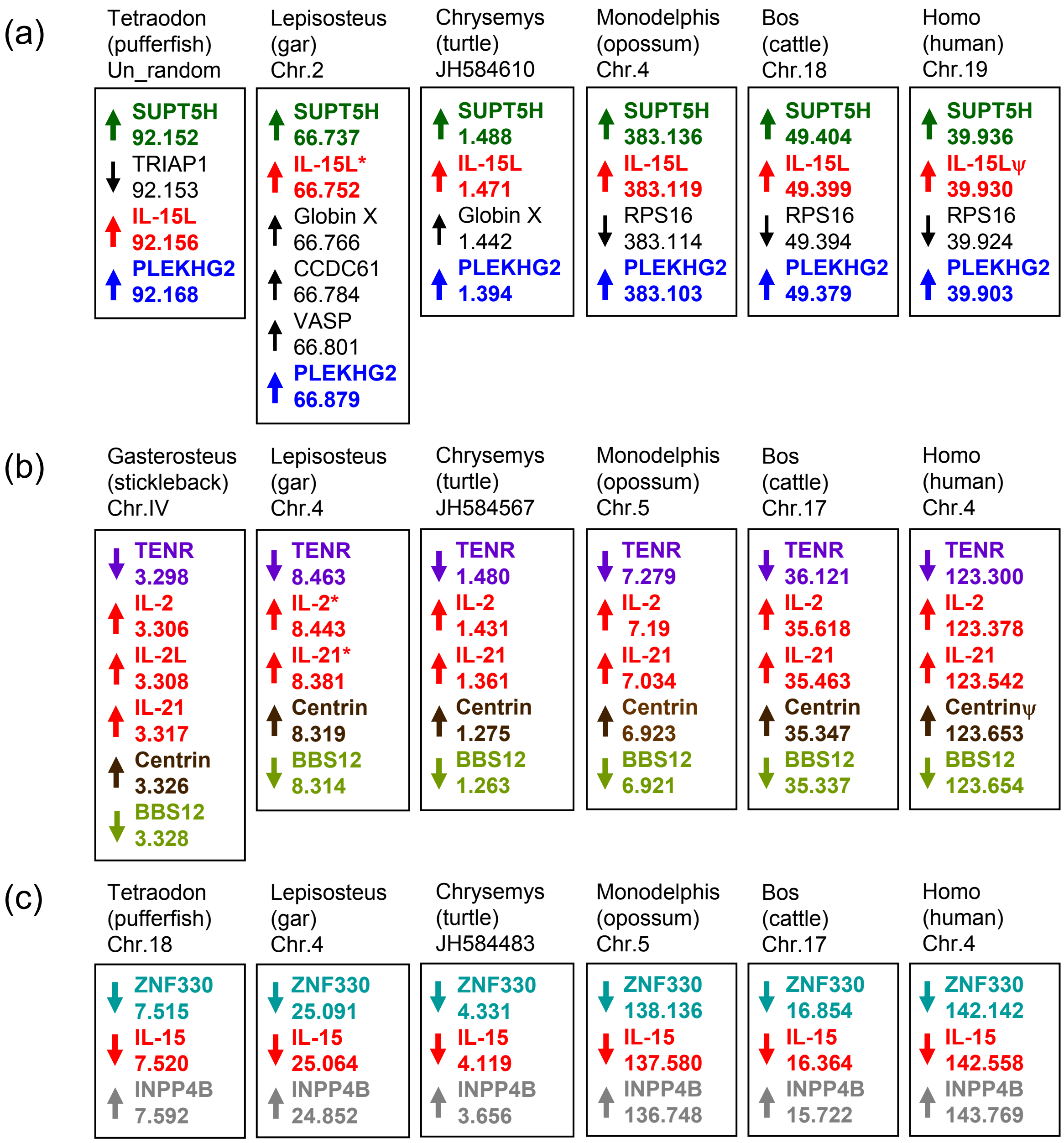

Fig. S1*B* *IL-15L* intron-exon organization

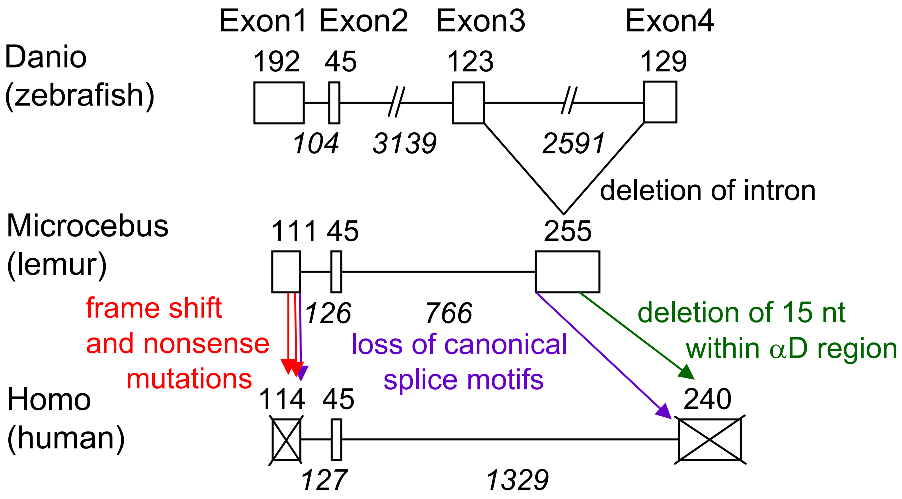

Fig. S1*C* Genomic locations of *IL-15Rα* and *IL-2Rα* in representative species

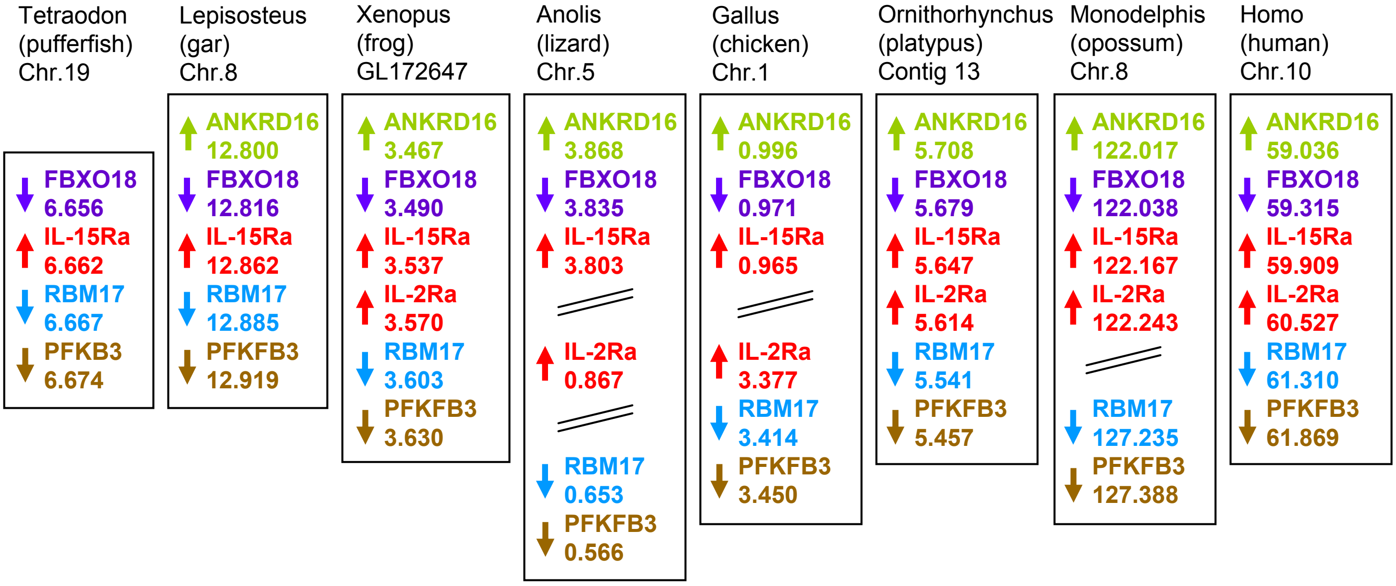

Fig. S1*D*
Intron-exon organization of *IL-15Rα* and *IL-2Rα* in representative species

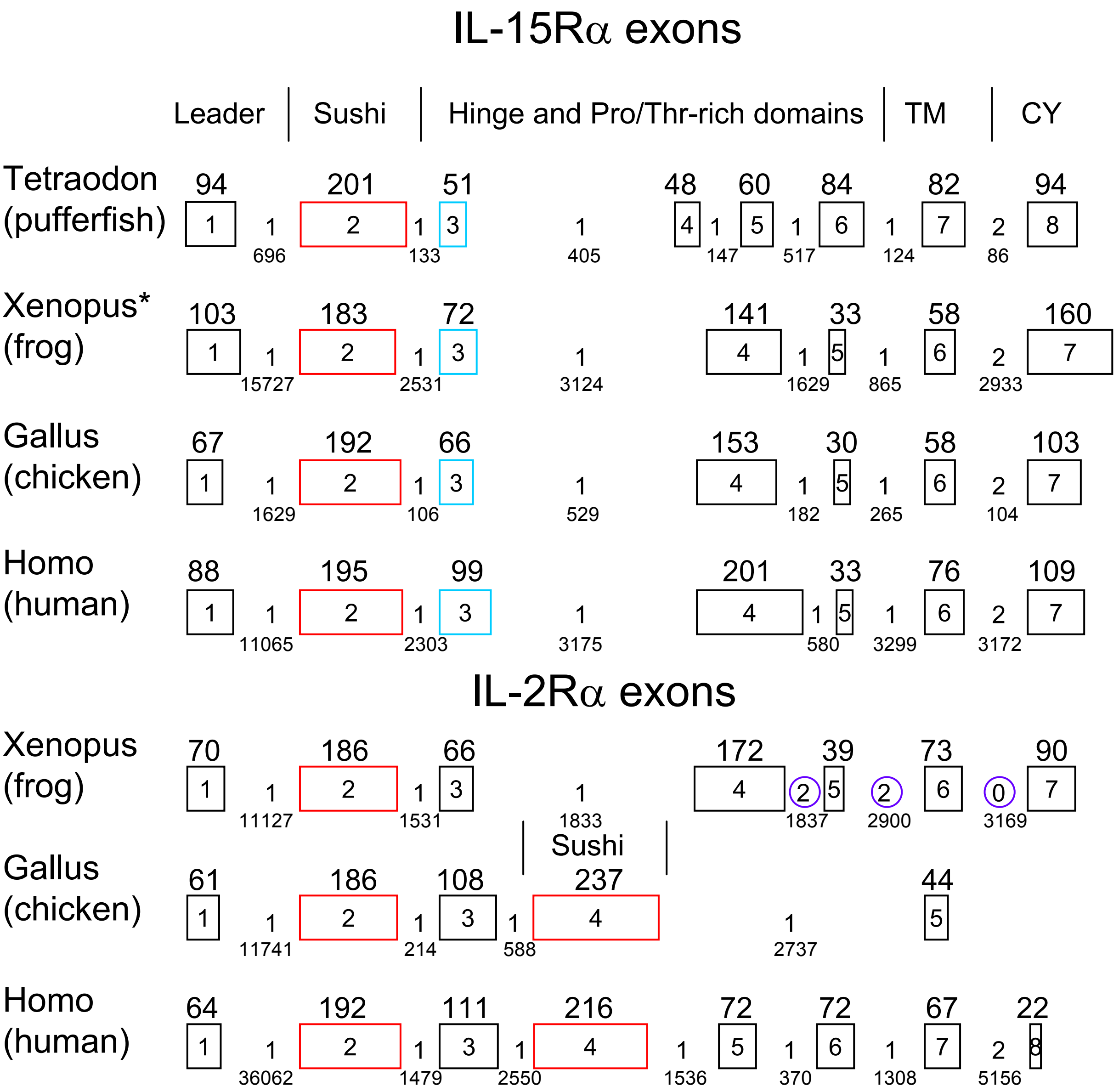

Fig. S1*E* Deduced amino acid sequences encoded by the genes shown in Fig. S1*D*

Tetraodon (pufferfish) IL-15Rα

Exon1: MVSCQMDRGPSLFSLCGLIYCVFQTVPFSIGD
Exon2: ESA**C**PCPKIPPVNLTEAPPTDCFQIDAKFRYK**C**KAGYVRKAGRSNLIT**C**ILKGHRAEWTQPDLL**C**IR
Exon3: **DP**KIGPTTKPTNPPLIS
Exon4: DHTEFLNVSITSTTVT
Exon5: KTASVDPTWTSPTTSDSLPA
Exon6: GTSGFAIGGTNAPQGGKNNPPDFASQPK
Exon7: YIVSLCVGLVCVVALVGGLIYIFFFKS
Exon8: RASPAEQPLSAEQIPLGKRPIGVDGSPRFFN

Xenopus (frog) IL-15Rα

Exon1: MACVLPSTCTTLYAAALMAVLLILQIQGHGTRSHV
Exon2: **C**STPKKVEHANDIEGEYKLDSVVRYK**C**QTDYKREAGTSNLAV**C**MLIDGKAQWSYGNIS**C**II
Exon3: **DP**KLLSTTSSSITELKPTAFSPSE
Exon4: PEALTFQKSTGTSAQIDTTKGMLPTTQPEQLVSKPPELTTGQTDQLE
Exon5: KKLLPGYISIG
Exon6: SVACISIIVISLILFLKYR
Exon7: HCAAYEPDLRQTAQSLPYDGISDEVCAEQLECLNMDSISLHPTFADNTEVTYL

Gallus (chicken) IL-15Rα

Exon1: MDRLLLLCAALALFLPYSASDSA
Exon2: VQ**C**GRPKDVANAIINADDTALVNTILRYT**C**KLGYKRKAGTSTLIQ**C**ILISDKPVWTHTELQ**C**IR
Exon3: **DP**ALPPQTPSPELPTVTTSQRG
Exon4: TNTPSATVPVPSPETSVPPAMPKPPEMPTPAEGTALGTTPLPTIPMDHVAV
Exon5: STQTLASSIG
Exon6: ISILFVVSVAGFCYWRMKT
Exon7: RQNYVVAVTAIPMVAPTTAAENDEMLPPGDIPTG

Homo (human) IL-15Rα

Exon1: MAPRRARGCRTLGLPALLLLLLLRPPATRG
Exon2: IT**C**PPPMSVEHADIWVKSYSLYSRERYI**C**NSGFKRKAGTSSLTE**C**VLNKATNVAHWTTPSLK**C**IR
Exon3: **DP**ALVHQRPAPPSTVTTAGVTPQPESLSPSGKE
Exon4: PAASSPSSNNTAATTAAIVPGSQLMPSKSPSTGTTEISSHESSHGTPSQTTAKNWELTASASHQPPG
Exon5: VYPQGHSDTTV
Exon6: AISTSTVLLCGLSAVSLLACYLKSR
Exon7: QTPPLASVEMEAMEALPVTWGTSSRDEDLENCSHHL

Xenopus (frog) IL-2Rα

Exon1: MEHCLLVFELAFFHLILRCSSQDN
Exon2: **C**LYHFPEDIIIIPEQQFFLGTIINLS**C**NKEYQRERNTHGMYT**C**VNNSGVLKWKTEKDLK**C**IK
Exon3: KDGTTTSDQSMELEEKQHNHTD
Exon4: ICAPLPHIPNARLTFVKVPVGQELHYMNKGTRANITQKCITENASGKYLGKESTTRE
Exon5: IINNFINISDYKS
Exon6: SCCWRHAWSCCLYSSFFSSWLGRM
Exon7: EKTKINQNSGVFRANAVPAYVQSYEYKKKI

Gallus (chicken) IL-2Rα

Exon1: MELKRLLMWLLLGSIMGTGAD
Exon2: K**C**PRLSTTEFADVAAETYPLKTKLRYE**C**DSGYRRRSGNTLTIR**C**QNVSGTASWVHDELV**C**ID
Exon3: EKFLFSRNHTAKLNPTQQPARQTQSPAPPKQANNSS
Exon4: F**C**GMPQTVPHASLSVHQIYSVGQVLHFK**C**PTGYNKQLPTTGTIT**C**KNVDGRIKWTPVDRP**C**TNDSGPINKQLSHLMEAV
Exon5: IFFILLLHSAVFL

Homo (human) IL-2Rα

Exon1: MDSYLLMWGLLTFIMVPGCQAE
Exon2: L**C**DDDPPEIPHATFKAMAYKEGTMLNCE**C**KRGFRRIKSGSLYML**C**TGNSSHSSWDNQCQ**C**TSSA
Exon3: TRNTTKQVTPQPEEQKERKTTEMQSPMQPVDQASLPG
Exon4: H**C**REPPPWENEATERIYHFVVGQMVYYQ**C**VQGYRALHRGPAESV**C**KMTHGKTRWTQPQLI**C**TGEMETSQFPG
Exon5: EEKPQASPEGRPESETSCLVTTTD
Exon6: FQIQTEMAATMETSIFTTEYQVAV
Exon7: AGCVFLLISVLLLSGLTWQRRQ
Exon8: RKSRRTI

Fig. S1*F*

Phylogenetic tree inferred by Neigbor Joining method for the IL-15Rα and IL-2Rα sushi domain sequences compared in main text Fig. 5

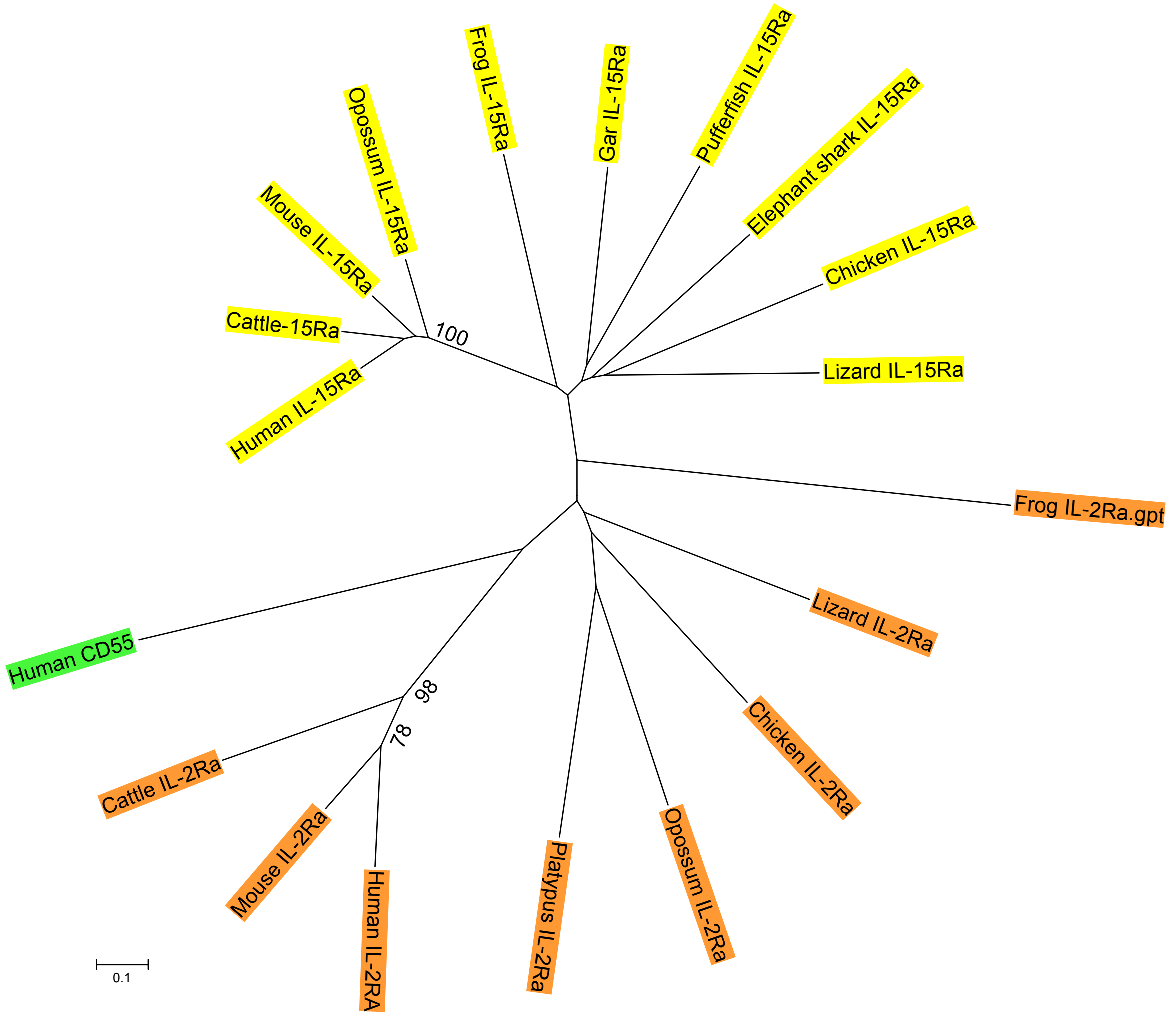

Supplement: Supplementary file 4 — (PDF 320 kb) [file 251_2013_747_MOESM4_ESM.pdf]
