# Supplementary material for: Identification of a gene for an ancient cytokine, interleukin 15-like, in mammals; interleukins 2 and 15 co-evolved with this third family member, all sharing binding motifs for IL-15Rα
Source: Immunogenetics. 2013 Nov 26;66(2):93–103. doi: 10.1007/s00251-013-0747-0 (PMC3894449; doi:10.1007/s00251-013-0747-0)
Supplement: Supplementary file 5 — (PDF 325 kb) [file 251_2013_747_MOESM5_ESM.pdf]

Supplementary Figure 2 (Fig. S2).

Alignment of *IL-15L* in tetrapod species (coding exons plus small parts of introns)

Table of Contents:

**Legend to Figure S2**		Page 2
**Fig. S2**	Alignment of *IL-15L* in tetrapod species (coding exons plus small parts of introns)	Page 6

Legend to Figure S2

Alignment of *IL-15L* in tetrapod species (coding exons plus small parts of introns). Species with names shaded green possess consensus intact *IL-15L* ORF. Sequences in species with names shaded gray may encode functional IL-15L protein, but are peculiar: (i) *Canus familiaris* (dog), *Ailuropoda melanoleuca* (giant panda), *Ursus americanus* (black bear), and *Odobenus rosmarus* (walrus) *IL-15L* have GTG as possible start codon, which in higher eukaryotes is rarely used [reference 1], or, if upstream ATGs as found in a reported bear cDNA sequence (EST GW294330) would be used for initiation of translation, do not encode likely leader peptides (Fig. S4); (ii) *Tursiops truncatus* (bottlenose dolphin) and *Python molurus* (Indian rock python) *IL-15L* encode unusually long C-termini. In species with names shaded red the consensus *IL-15L* ORF is incapacitated, with incapacitation motifs shaded red: X , frame-shift; *, stop codon; long red stretches indicate absence of *IL-15L* parts within continuous database sequences and probably represent true deletions. Partially blue-shaded species names refer to *IL-15L* sequences for which information is incomplete, with the blue stretches within the depicted sequence indicating absence of information for the respective region because of shortness of scaffolds or unknown ("NNNNN") parts within a scaffold. Many tetrapod *IL-15L* genes have their start codons in a poor context for translation according to Kozak rule [2]. Positive exceptions are *IL-15L* in *Bos Taurus* (domestic cow; cattle), *Ovis aries* (domestic sheep), *Equus caballus* (domestic horse), *Ceratotherium simum* (white rhinoceros), *Ornithorhynchus anatinus* (platypus), and *Chrysemis bellii* (painted turtle), as they all have a purine at position -3. Sequence databases in Ensembl for the rodents *Rattus norwegicus* (rat), *Cricetulus griseus* (Chinese hamster), and *Microtus ochrogaster* (prairie vole) suggest that traces of *IL-15L* were entirely deleted from their genomes (not

shown). The figure shows the *IL-15L* sequence of *Papio hamadryas* (Hamadryas baboon), but we did not include the Ensembl sequence of *Papio anubis* (olive baboon) since we felt it does not provide extra information.

Numbers refer to the length of the alignment. Amino acid sequences are indicated below the second nucleotide of codons. Hyphens indicate gaps in the alignment. Intron positions are boxed, with ellipses schematically representing the bulk of the intron. The sequences shown are from genome sequence reports unless mentioned otherwise and can be found at: *Bos Taurus*: Ensembl "UMB 3.1" Chr18 (identical with our cDNA sequence at GenBank JX271582); *Ovis aries*: our cDNA sequence at GenBank JX271585 plus intron sequences from Ensembl "oviAri1" Chr14 (which in the ORF is 2 nt different); *Tursiops truncates*: Ensembl "turTru1" scaffold 3071; *Sus scrofa*: GenBank FP236263 (fragment of Chr14; identical with our cDNA sequence at GenBank JX271584); *Vicugna pacos*: Ensembl "vicPac1" scaffold 27695; *Equus caballus*: Ensembl "EquCab2" Chr10 (identical with our cDNA sequence at GenBank KC914889); Ceratotherium simum; Ensembl "CerSimSim1" scaffold JH767830; *Myotis lucifugus*: Ensembl "Myoluc2.0" scaffold GL429952; *Pteropus vampirus*: Ensembl "pteVam1" scaffold 3305; *Felis catus*: Ensembl "Felis_catus-6.2" ChrE2; *Canis familiaris*: Ensembl "CanFam2.0" Chr1 (identical with our cDNA sequence at GenBank JX271586); *Ailuropoda melanoleuca*: Ensembl "ailMel1" scaffold GL193591; *Ursus americanus*: GenBank GW294330 (an EST sequence); *Odobenus rosmarus*: GenBank NW_004450317; *Mustela putorius furo*: GenBank AEYP01088720; *Erinaceus europaeus*: Ensembl "HEDGEHOG" scaffold 280547; *Sorex araneus*: Ensembl "SorAra2.0" scaffold JH798 250; *Microcebus murinus*: Ensembl "micMur1" scaffold 3863; *Daubentonia madagascariensis*: GenBank AGTM011747828; *Otolemur garnetti*:

Ensembl “OtoGar3” scaffold GL873752; *Tarsius syrichta*: GenBank accessions gnl|ti|1607110508 and gnl|ti|1612376660; *Callithrix jacchus*: Ensembl “C_jacchus3.2.1” Chr22; *Saimiri boliviensis*: Ensembl “SaiBol1.0” scaffold JH378281; *Chlorocebus sabaeus*: Ensembl “ChlSab1.0” Chr6; *Macaca mulatta*: Ensembl “MMUL_1” Chr19; Papio hamadryas: Ensembl “Pham_1.0” Contig426756 and Contig786684; *Nomascus leucogenys*: Ensembl “Nleu1.0” scaffold GL397330; *Gorilla gorilla*: Ensembl “gorGor3.1” Chr19; *Pongo abelii*: Ensembl “PPYG2” Chr19; *Pan troglodytes*: Ensembl “CHIMP2.1.4” Chr19; *Homo sapiens*: Ensembl “GRCh37” Chr19; *Tupaia belangeri*: GenBank accessions gnl|ti|1040766702 and gnl|ti|1042169077; *Oryctolagus cuniculus*: our cDNA sequence at GenBank JX271583 and Ensembl “oryCun2”Chr5; *Ochotona princeps*: Ensembl “OchPri3” scaffold JH802134; *Mus musculus*: Ensembl “NCBIM37” Chr7; *Heterocephalus gaber*: GenBank AFSB01139169; *Cavia porcellus*: Ensembl “cavPor3” scaffold 52; *Procavia capensis*: Ensembl “proCap1” scaffold 2974; *Trichechus manatus*: GenBank AHIN01131269; *Loxodonta africana*: Ensembl “loxAfr3” scaffold 99; *Orycteropus afer*: Ensembl “OryAfe1” scaffold JH864707; *Dasypus novemcinctus*: GenBank gnl|ti|2012596402, gnl|ti|1814056052 and gnl|ti|1858783515; *Monodelphis domestica*: Ensembl “BROADO5” Chr4; *Macropus eugenii*: GenBank gnl|ti|1654192767 and EX197763 (the latter sequence was reported as EST but may have derived from genomic DNA); *Sacrophilus harrisii*: GenBank AEFK01122608 (part of Chr3); *Ornithorhynchus anatinus*: Ensembl “OANA5” Contig23177 and GenBank gnl|ti|772263685; *Chrysemis picta bellii*: Ensembl “ChrPicBel3.0.1” scaffold JH584610; *Pelodiscus sinensis*: Ensembl “PelSin_1.0” scaffold JH211032; *Anolis carolinensis*: Ensembl “AnoCar2.0” scaffold GL343635; *Python molurus*: GenBank AEQU010066174.

References in this figure legend:

[1] Touriol C, et al. (2003) Generation of protein isoform diversity by alternative initiation of translation at non-AUG codons. *Biol Cell* 95(3-4): 169-178.

[2] Kozak M (1986) Point mutations define a sequence flanking the AUG initiator codon that modulates translation by eukaryotic ribosomes. *Cell* 44(2): 283-292.

Fig. S2 Alignment of *IL-15L* in tetrapod species (coding exons plus small parts of introns)

Page A1 (this part of the *IL-15L* alignment also includes pages B1 and C1; the 3' ends continue on page A2)

Exon1

```
                 1                                                                                                         99
Cetartiodactyla
Bos              AGTATGGGG GTGGGCAGGG TGTCCATGTG GCTTCTCTGG ACCACCCTCC TGCTGGTGCT GCCCTTGGGA GGCCTAGGAC CAC------T CCTCTGCCCA
(cattle)             M  G   V  G  R   V  S  M  W   L  L  W   T  T  L   L  L  V  L   P  L  G   G  L  G   P        L   L  C  P
Ovis                   GGG ATGGGCAAGG TGTCTATGTG GCTTCTCTGG ACCACCCTCC TGCTGGTGCT GCCCTTGGGA GGCCTAGGAC CAC------C CCTCTGCCCG
(sheep)                     M  G  K   V  S  M  W   L  L  W   T  T  L   L  L  V  L   P  L  G   G  L  G   P        P   L  C  P
Tursiops                                CCCATGTG GCCTCTCTGG ACCATCCTCC TGCTGGTGCT GCCCTTGGGA GGCCTAGGAC CAC------C CTTCTGCCCA
(dolphin)                                   M  W   P  L  W   T  I  L   L  L  V  L   P  L  G   G  L  G   P        P   F  C  P
Sus                                     CCCATGTG GCATCTCTGG ACCATCCTCC TGCTGGTGCC ACCCTTGGGG GGCCTAGGAC CAC------C CCTCTGCCCC
(pig)                                       M  W   H  L  W   T  I  L   L  L  V  P   P  L  G   G  L  G   P        P   L  C  P
Vicugna                                 CCCATGTG GCCTCTCCAA ACCATCTTC- TGCAGGTGCC ACACTTGGGG AGCCTGGGAC CAC------C GCTCTGCCCA
(alpaca)                                    M  W   P  L  Q   T  I  F X    Q  V  P   H  L  G   S  L  G   P        P   L  C  P
Perissodactyla
Equus                  GGG ATGGGCAGGG TGCCCATATG GCCTCTCTGG ACCATTGTCC TGCTGATGCG GCCTTTGGGG GGCCTAGGAC CAC------C CCTCTGCCCT
(horse)                     M  G  R   V  P  I  W   P  L  W   T  I  V   L  L  M  R   P  L  G   G  L  G   P        P   L  C  P
Ceratotherium          GGG ATGGGCAGGG TGTCCATATG GCCTCTCTGG ACCATCCTCC TGCTGATGTG GCCTTTGGGG GGCCTAGGAC CAC------C CCTCTGCCCT
(rhinoceros)                M  G  R   V  S  I  W   P  L  W   T  I  L   L  L  M  W   P  L  G   G  L  G   P        P   L  C  P
Chiroptera
Myotis                                  CCCATGCG GCCTCTCTGG ATCATTCTCC TGCTGGTAAG GCCTTCAGGG AGCCCAGGAC CAC------C CCTCTGTCCT
(brown bat)                                M  R   P  L  W   I  I  L   L  L  V  R   P  S  G   S  P  G   P        P   L  C  P
Pteropus                                CCTATGTG GACTCTTTGG CCCCCCCTCC TGCTGGTAAG GCCCTTGGGA GGCCTAGGAC CAC------C CTTCTGCCCT
(flying fox)                               M  W   T  L  W   P  P  L   L  L  V  R   P  L  G   G  L  G   P        P   F  C  P
Carnivora
Felis                                   CCCATGTG GCCTCTCTGG ACCATCCTTC TGCTGGTACG GCCCTTGGGA GGCCTAGGAT CAC------C CCTCTGCCCT
(cat)                                       M  W   P  L  W   T  I  L   L  L  V  R   P  L  G   G  L  G   S        P   L  C  P
Canis                                   CCCGTGTG GCCTCTCTGG ACCATCCTCC TGCTGGTACG GCCCTTGGGA AGCCTAGGAT CAC------C CCTCTGTCCT
(dog)                                       V  W   P  L  W   T  I  L   L  L  V  R   P  L  G   S  L  G   S        P   L  C  P
Ailuropoda                              CCCGTGTG GCCTCTCTGG ACCATCCTCC TGCTGGTACG GCCCTTGGGA GGCCTAGGAT CAC------C CCTCTGTCCT
(panda)                                     V  W   P  L  W   T  I  L   L  L  V  R   P  L  G   G  L  G   S        p   L  C  P
Ursus                                   CCCGTGTG GCCTCTCTGG ACCATCCTCC TGCTGGTACG GCCCTTGGGA GGCCTAGGAT CAC------C CCTCTGTCCT
(bear)                                      V  W   P  L  W   T  I  L   L  L  V  R   P  L  G   G  L  G   S        P   L  C  P
Odobenus                                CCTGTGTG GCCTCTCTGG ACCATCCTCC TCCTGGTACG GCCCTTGGGA GGCCTAGGAT CAC------C CCTCTGTCCT
(walrus)                                    V  W   P  L  W   T  I  L   L  L  V  R   P  L  G   G  L  G   S        P   L  C  P
Mustela                                 CCCATGTG GTCACTCTGG ACCATCCTCC TGCTGGTACG ACCCTTGGGA GGCCTAGGAT CAC------C CCTCTGTCCT
(ferret)                                    M  W   S  L  W   T  I  L   L  L  V  R   P  L  G   G  L  G   S        P   L  C  P
Lipotyphla
Erinaceus                               CCTATGTG GGCTCTCTGG GCCATTCTCC TGCTAGTGTG GCCTTCAGGG GGCCTAGGAT CCC------C TCTCTGCCCA
(hedgehog)                                  M  W   A  L  W   A  I  L   L  L  V  W   P  S  G   G  L  G   S        P   L  C  P
Sorex                                   CCCATGTG GGCTCTCTGG ACCATCCTCC TGCTGGTGTG GCCCTTGAGG GTTCTCGGAT CAT------C CCTCTGCCCA
(shrew)                                     M  W   A  L  W   T  I  L   L  L  V  W   P  L  R   V  L  G   S        S   L  C  P
```

Page B1 (this part of the *IL-15L* alignment also includes pages A1 and C1; the 3’ ends continue on page B2)

```
                        1                                                                                   99
Primata: Strepsirhini
Microcebus          CCCATGTG GCCTCTCTGG ACCATCCTCC TGCTCATGCA GCTCTGGGGG AGCCTAGGAG CCC------C CCTCTGCCGG
(mouse lemur)          M  W   P  L  W   T  I  L   L  L  M  Q   L  W  G   S  L  G   A         P   L  C  R
Daubentonia         CCCATGTG GCCTCTCTGG ACCATCCTCC TGTTCTTGCA GCTCTGGGGG TGCCTAGGAA -CC------C CCTCTGCCAG
(aye-aye)              M  W   P  L  W   T  I  L   L  F  L  Q   L  W  G   C  L  G   X         P  L  C  Q
Otolemur
(bush baby)
Primata: Haplorhini
Tarsius                                     TCA TGCTCGTGCA GCCCTGTGGG -GCTTAGGAA GCC------C CCTCTGCCGG
(tarsier)                                        M  L  V  Q   P  C  G  X   L  G   S         P   L  C  R
Callithrix          CCTATGTT GCCTCTGTGG ATCATCCTCC TGCTTGTGCA GTCCTGGGAG GGCCTAGGAA TGC------C CCTCTGCTGG
(marmoset)             M  L   P  L  W   I  I  L   L  L  V  Q   S  W  E   G  L  G   M         P   L  C  W
Saimiri             TCCATGTT GCCTCTGTGG ATCATCCTCC TGCTTGTGCA GCCCTGGGAG GGCCTAGGAA TGC------C CCTCTGCTGG
(squirrel monkey)      M  L   P  L  W   I  I  L   L  L  V  Q   P  W  E   G  L  G   M         P   L  C  W
Chlorocebus         CCGGTGTT GCTTCTGTGG ATCATCCTCC TGCTTGTACA GCCCTGGGGG GACCTAGGAA TGC------C CCTCTGCTGG
(vervet monkey)        V  L   L  L  W   I  I  L   L  L  V  Q   P  W  G   D  L  G   M         P  L  C  W
Macaca              CCGGTGTT GCTTCTGTGG ATCATCCTCC TGCTTGTACA GCCCTGGAGG GACCTAGGAA TGC------C CCTCTGCTGC
(macaque)              V  L   L  L  W   I  I  L   L  L  V  Q   P  W  R   D  L  G   M         P   L  C  C
Papio               CCGGTGTT GCTTCTGTGG ATCATCCTCC TGCTTGTACA GCCCTGGGGG GACCTAGGAA TGC------C CCTCTGCTGG
(baboon)               V  L   L  L  W   I  I  L   L  L  V  Q   P  W  G   D  L  G   M         P   L  C  W
Nomascus            CCCATGTT GCTTCTGTGG ATCATCCTCC TGCTTGTACA GCCCTGGGAG GGCCTAGGAA CGC------C CCTCTGCTGG
(gibbon)               M  L   L  L  W   I  I  L   L  L  V  Q   P  W  E   G  L  G   T         P   L  C  W
Gorilla             CCCATGTT GCTTCTGTGG ATCATCCTCC TGCTTGTACA GCCCTGGGAG GGCCTAGGAA CGC------C CCTCTGCTGG
(gorilla)              M  L   L  L  W   I  I  L   L  L  V  Q   P  W  E   G  L  G   T         P   L  C  W
Pongo               CCCATGTT GCTTCTGTGG ATCATCCTCC TGCTTGTACA GCCCTGGGAG GGCCTGGGAA CGC------C CCTCTGCTGG
(orangutan)            M  L   L  L  W   I  I  L   L  L  V  Q   P  W  E   G  L  G   T         P   L  C  W
Pan                 CCCATGTT GCTTCTGTGG ATCATCCTCC TGCTTGTATA GCCCTGGGAA GGCCTAGGAA CGC------C CTTCTGCTGG
(chimpanzee)           M  L   L  L  W   I  I  L   L  L  V  *   P  W  E   G  L  G   T         P   F  C  W
Homo                CCCATGTT GCTTCTGTGG ATCATCCTCC TGCTTGTACA GCCCTGGGAG GGCCTAGGAA CGC--CCGCC CCTCTGCTGG
(human)                M  L   L  L  W   I  I  L   L  L  V  Q   P  W  E   G  L  G   T      X  P   L  C  W
Scandentia
Tupaia              CCCATGCG GGTTCACTGG ACCATCCTTC TACTCATGCG GCCCTTGGGG GGCCTAGGAA CCC------C TCTCTGCCCA
(tree shrew)           M  R   V  H  W   T  I  L   L  L  M  R   P  L  G   G  L  G   T         P   L  C  P
Lagomorpha
Oryctolagus         CTCATGTG GCCTCTCTGG ACCATCCTCC TGCTTGGGGG ACTCTTGGGG GGCCTAGGAA CCC------C TCTCTGCCGA
(rabbit)               M  W   P  L  W   T  I  L   L  L  G  G   L  L  G   G  L  G   T         P   L  C  R
Ochotona            CCCATGTG GCCTCTCTGG ACCATCCTCC TGCTTGGGGG GCTCTTGGGA GGTCTGGGAA CCC------C CCTCTGCCGG
(pika)                 M  W   P  L  W   T  I  L   L  L  G  G   L  L  G   G  L  G   T         P   L  C  R
Rodentia
Mus
(mouse)
Heterocephalus
(mole rat)
Cavia               TTCATGTA GCCTCTCTGG ATCATTCTCC TACTGAAGGA AGT--TGGGG ACCCTAGGAC CCC------C TCTACCTCTT
(guinea pig)           M  *   P  L  W   I  I  L   L  L  K  E  X   G   T  L  G   P         P   L  P  L
```

Page C1 (this part of the *IL-15L* alignment also includes pages A1 and B1; the 3' ends continue on page C2)

```
                                         1                                                                                99
Afrotheria
Procavia (hyrax)                  CCTATGTG GCCTCTCTGG ATCATCCTTC TGCTGGTGCC ACCCTTGTGG GGCCTGGGGC CTC------C CCTCTGCTCT
                                     M  W   P  L  W   I  I  L   L  L  V  P   P  L  W   G  L  G   P         P   L  C  S
Trichechus (manatee)
Loxodonta (elephant)              CCCATGTG GCCTCTCTGG ACCATCCTCC TGCCAGTGCA ACCCTTGGGG GGCCTGGGGC CC-------C CCTCTGCTCT
                                     M  W   P  L  W   T  I  L   L  P  V  Q   P  L  G   G  L  G   P  X          L  C  S
Orycteropus (aardvark)                                                                                           TCTGTCCT
                                                                                                                   V  P
Xenarthra
Dasypus (armadillo)               TCAGTGCA GCCTCTCTGG ACCGTTCTCT TGCTGG---- -------GGG GACCTGGGAC CAC-- ---C CCTCTGCCCT
                                     V  Q   P  L  W   T  V  L   L  L  X             G   D  L  G   P         P   L  C  P
Marsupialia
Monodelphis (opossum)             CTCATGTG GCTCTTCTGG ATCTTCCTTG TGTTGGTTCA CCCCCTGGGG ACCCCCCCTC TGCGGCCCCC CAGCTGCCCT
                                     M  W   L  F  W   I  F  L   V  L  V  H   P  L  G   T  P  P   L  R  P  P   S  C  P
Macropus (wallaby)                CACATGTG GTTCTTATGG GCCTTCCTTG TGTTCGTTCG CCCCCTGGGG GGGCCCCCTT TGGGGCCCCC CAGCTGCCCT
                                     M  W   F  L  W   A  F  L   V  F  V  R   P  L  G   G  P  P   L  G  P  P   S  C  P
Sacrophilus (Tasmanian devil)
Monotremata
Ornithorhynchus (platypus)        AGCATGTG GTCCTTCAGG GTCTTGCTCC TGCTGGCACA GCTCCTGGTG TGTCTCTGC- --------CC ACCCTGCCCA
                                     M  W   S  F  R   V  L  L   L  L  A  Q   L  L  V   C  L  C         P   P  C  P
Reptilia: Chelonia
Chrysemys (painted turtle)        GGCATGAG GGGCCTCCAC GCACTCCTCT TGGTCTCTTTG CTGACGTGC TTCTCGGTGC GCGGGAGACG GGTCTGTGGA
                                     M  R   G  L  H   A  L  L   L  V  S  L   L  T  C   F  S  V   R  G  R  R   V  C  G
Pelodiscus (C. s. turtle)      GGCC AGCCTGCCTC CAGCCACGCG GGCCCTCCAC CCCCTCCTCT GGGTCTCTTT GCTGATGTGC CTCTCGGCAG GAGGGAGCCC GCTCTGCGGG
                                 A  S  L  P  P  A  T  R   A  L  H   P  L  L   W  V  S  L   L  M  C   L  S  A   G  G  S  P   L  C  G
Reptilia: Lepidosauria
Anolis (lizard)
Python (python)
```

Page A2 (this part of the *IL-15L* alignment also includes pages B2 and C2; the 3’ ends continue on page A3)

```
                Exon1                                                            intron          Exon2
             100                                                                                                                   189
Cetartiodactyla
          Bos  AGGGAGCCTT TCTACTTCCT CATTGCCATC ACGAAGATGC TGGTG>....>CAGGA AAACAAAAAT GATGG---CA GTCTGTACAC CCCAGATAAT
     (cattle)   R  E  P   F  Y  F  L   I  A  I   T  K  M   L             E   N  K  N   D  G      S  L  Y  T   P  D  N
         Ovis  AGGGAGCCTT TCTACTTCCT CGTTGCCATC ACGAAGATGC TGGTG>....>CAGGA AAACAAAAAT GATGG---CA GTCTCTACAC CCCAGATAAT
      (sheep)   R  E  P   F  Y  F  L   V  A  I   T  K  M   L             E   N  K  N   D  G      S  L  Y  T   P  D  N
     Tursiops  AGGGAGACTT TCTGCTTCCT CATTGCCATC ATGAAGTTGC TGGTG>....>CAGGG AAGCAAAAAT GATGG---TA CTCTTTACAC CGCAGATGAT
    (dolphin)   R  E  T   F  C  F  L   I  A  I   M  K  L   L             G   S  K  N   D  G      T  L  Y  T   A  D  D
          Sus  CGGGAGCCCT TCTACTTCCT CATTGCCATC ATGAAGATGC TGGTG>....>TAGGG CAACAAAAAT GATGG---CA CCCTCTACAC CCCAGATGAT
        (pig)   R  E  P   F  Y  F  L   I  A  I   M  K  M   L             G   N  K  N   D  G      T  L  Y  T   P  D  D
      Vicugna  CGGGAGCGTT TCTACTTCCT CATTGCCATC ATGAAGATCC TGGTG>....>TAGGG AAACAAAAAT GATGG---CA CTCTCTACAC CCCAGATGAT
     (alpaca)   R  E  R   F  Y  F  L   I  A  I   M  K  I   L             G   N  K  N   D  G      T  L  Y  T   P  D  D
Perissodactyla
        Equus  CGGGAGCCTT TCTACTTCCT TCTTGCCATC ATGAAGATGC TGGTG>....>TAGGG AAACAAAAAT GATGG---CA CTCTCTACAC CCCAGATGAT
      (horse)   R  E  P   F  Y  F  L   L  A  I   M  K  M   L             G   N  K  N   D  G      T  L  Y  T   P  D  D
 Ceratotherium CGGGAGCCTT TCTACTTCCT CGTTGCCCTC ATGAAGATGC TGGTG>....>TAGGG AAACAAAAAT GATGG---CA CTCTCTACAC CCCAGATGAT
  (rhinoceros)  R  E  P   F  Y  F  L   V  A  L   M  K  M   L             G   N  K  N   D  G      T  L  Y  T   P  D  D

Chiroptera
       Myotis  CAGGAGCCTT TCTACTTCCT GGTTGCCATC ATGAAGATGC TGGTG>....>TAGTG AAACAAAAAT GATGG---CA GTCTCTACAC CCCAGATGAT
  (brown bat)   Q  E  P   F  Y  F  L   V  A  I   M  K  M   L             *   N  K  N   D  G      S  L  Y  T   P  D  D
     Pteropus  CTGGAGCCTT TCTACCTCCT GGT-GCCCTC ATGAAGATGC TGGTG>....>TATGG AAACAAAAAT GATGG---CA TCCTCTACAC CCCAGATGAT
 (flying fox)   L  E  P   F  Y  L  L   X  A  L   M  K  M   L             G   N  K  N   D  G      I  L  Y  T   P  D  D
Carnivora
        Felis  CGGGAGCCTT TCTACTTCCT TGTTGCCATC ATGAAGATGC TGGTG>....>TAGGG AAACAAAAAT GATGG---CA CTCTCTACAC CCCTGATGAT
        (cat)   R  E  P   F  Y  F  L   V  A  I   M  K  M   L             G   N  K  N   D  G      T  L  Y  T   P  D  D
        Canis  CGGGAGCCTT TCTACTTCTT AATTGCCATC ATGAAGATGC TGGTG>....>TAGGG AAACAAAAAT GATGG---CA CTCTTTACAC CCCAGATGAC
        (dog)   R  E  P   F  Y  F  L   I  A  I   M  K  M   L             G   N  K  N   D  G      T  L  Y  T   P  D  D
   Ailuropoda  CGGGAGCCTT TCTACTTCCT TGTTGCCATC ATGAAGATGC TGGTG>....>TAGGG AAACAAAAAT GATGG---CA CTCTCTACAC CCCAGATGAT
      (panda)   R  E  P   F  Y  F  L   V  A  I   M  K  M   L             G   N  K  N   D  G      T  L  Y  T   P  D  D
        Ursus  CGGGAGCCTT TCTACTTCCT TGTTGCCATC ATGAAGATGC TG                GG AAACAAAAAT GATGG---CA CTCTCTACAC CCCAGATGAT
       (bear)   R  E  P   F  Y  F  L   V  A  I   M  K  M   L             G   N  K  N   D  G      T  L  Y  T   P  D  D
     Odobenus  CGGGAGCCTT TCTACTTCCT AGTTGCCATA ATGAAGATGC TGGTG>....>TAGGG AAACAAAAAT GATGG---CA CTCTCTACAC CCCAGATGAT
     (walrus)   R  E  P   F  Y  F  L   V  A  I   M  K  M   L             G   N  K  N   D  G      T  L  Y  T   P  D  D
      Mustela  CGGGAGCCTT TCTACTTCCT TGTTGCCATC ATGAAGATGC TGGTG>....>TAGGG AAACAAAAAT GATGG---CA CTCTCTACAC ACCAGATGAC
     (ferret)   R  E  P   F  Y  F  L   V  A  I   M  K  M   L             G   N  K  N   D  G      T  L  Y  T   P  D  D
Lipotyphla
    Erinaceus  AAGGAGCCTT TCTACTTCCT CGTGGCCCTC ATGAAGATGC TGGTG>....>TAGGG AAACAAAAAT TACGG---CA CTCTGTACAC CCCAGATGAC
   (hedgehog)   K  E  P   F  Y  F  L   V  A  L   M  K  M   L             G   N  K  N   Y  G      T  L  Y  T   P  D  D
        Sorex  AAAGAGCCTT TCTACTTCCT CCTCGCCATC ATGAAGAAGC TGGTG>....>CAGGA AAAAAAGAAT GATGG---CA CGCTCTATAC CCCAAATGAT
      (shrew)   K  E  P   F  Y  F  L   L  A  I   M  K  K   L             E   K  K  N   D  G      T  L  Y  T   P  N  D
```

Page B2 (this part of the *IL-15L* alignment also includes pages A2 and C2; the 3' ends continue on page B3)

```
                     100                                                                                              189
Primata: Strepsirhini
Microcebus           AGGGAGCCTT TTTATTTCCT TGTGGCTATC ATGAAGATGC TGGTG>....>TAGGG AAACAAAAAT GACGG---CA CTCTCTACAC CCCAGATGAT
(mouse lemur)         R  E  P   F  Y  F  L   V  A  I   M  K  M   L           G   N  K  N   D  G     T  L  Y  T   P  D  D
Daubentonia          AGGGAGCCTT TTCACTTCCT TGTTGCTGTC ATGAAGATGC TGGTG>....>TAGGG AAACAAGAAT GATAG---CA CTCTCTATAC CCCC-ATGAT
(aye-aye)             R  E  P   F  H  F  L   V  A  V   M  K  M   L           G   N  K  N   D  S     T  L  Y  T   P  X  D
Otolemur
(bush baby)
Primata: Haplorhini
Tarsius              AGGGAGCCTT TTTACTTTCT TGACGCCATC ATGAAGAGGC TGGTG>....>TAGGG AGACAGAAAT GATGG---CA CTCTCTACAC CCCAGATGAT
(tarsier)             R  E  P   F  Y  F  L   D  A  I   M  K  R   L           G   D  R  N   D  G     T  L  Y  T   P  D  D
Callithrix           AGGGAGCCTT TTTACTTCTT TGCGGCCCTC GTGAACATAC TAGTG>....>TAGGG AAACAAAAAT GAT------- ---------- ----------
(marmoset)            R  E  P   F  Y  F  F   A  A  L   V  N  I   L           G   N  K  N   D
Saimiri              AGGAAGCCTT TTTCCTTCTT TGTGGACCTC GTGAACATAC TAGTG>....>TAGGG AAACAGAAAC GATGG---CA CTTTCTACAC CACAAATGAT
(squirrel monkey)     R  K  P   F  S  F  F   V  D  L   V  N  I   L           G   N  R  N   D  G     T  F  Y  T   T  N  D
Chlorocebus          AGGGAGCCTT TT-ACTTCCT TGCAGCCCTC -TGAAGATAC TAGTG>....>TAGGG AAACAAAAAT GATGA---CA CCCTCTACAC CCCAGATGAT
(vervet monkey)       R  E  P   F  X  F  L   A  A  L   X  K  I   L           G   N  K  N   D  D     T  L  Y  T   P  D  D
Macaca               AGGGAGCCTT TTTACTTCCT TGCAGCCCTC -TGAAGATAC TAGTG>....>TAGGG AAACAAAAAT GATGA---CA CCCTCTACAC CCCAGATGAT
(macaque)             R  E  P   F  Y  F  L   A  A  L   X  K  I   L           G   N  K  N   D  D     T  L  Y  T   P  D  D
Papio                AGGGAGCCTT TTTACTTCCT TGCAGCCCTC -TGAAGATAC TAGTG>....>TAGGG AAACAAAAAT GATGA---CA CCCTCTACAC CCCAGATGAT
(baboon)              R  E  P   F  Y  F  L   A  A  L   X  K  I   L           G   N  K  N   D  D     T  L  Y  T   P  D  D
Nomascus             AGGGAGGCTT TTTACTTCCT TGTGGCCCTC -TGAAGATAC TAGTG>....>GAGGA AAACAAAAAT AATGA---CA CCCTCTACGC CCTAAATGAT
(gibbon)              R  E  A   F  Y  F  L   V  A  L   X  K  I   L           E   N  K  N   N  D     T  L  Y  A   L  N  D
Gorilla              AGAGAGCCTT TTTACTTCCT TGCAGCCCTC -TAAAGATAC CAGCG>....>TAGGA AAACAAAAAT GATGA---CA CCCTCTACAC CCCAGATCAT
(gorilla)             R  E  P   F  Y  F  L   A  A  L   X  K  I   P           E   N  K  N   D  D     T  L  Y  T   P  D  H
Pongo                AGGG-GCGTT TTTACTTTCT TGCAGCCCTC -TGAAGATAC TAGTG>....>TAGGA AAACAAAAAT GATGA---CA CCTTCTACAC CCCAGATGAT
(orangutan)           R  X  R   F  Y  F  L   A  A  L   X  K  I   L           E   N  K  N   D  D     T  F  Y  T   P  D  D
Pan                  AGGGAGGCTT TTTACTTCCT TGCAGCCCTC -TGAAGATAC CAGCG>....>TAGGA AAACAAAAAT GATGA---CA CCCTATACAC CCCAGATGAT
(chimpanzee)          R  E  A   F  Y  F  L   A  A  L   X  K  I   P           E   N  K  N   D  D     T  L  Y  T   P  D  D
Homo                 AGGGAGCCTT TTTACTTCCT TGCAGCCCTC -TGAAGATAC CAGCG>....>TAGGA AAACAAAAAT GATGA---CA CCCTCTACAC CCCAGATGAT
(human)               R  E  P   F  Y  F  L   A  A  L   X  K  I   P           E   N  K  N   D  D     T  L  Y  T   P  D  D
Scandentia
Tupaia               AGGGAGCCTT TTTACTTCCT CCTTGCTGTC ACGAAGATGT TGGTG>....>CAGGG AAACAAAAAT GATGG---CA CTCTCTACAC CCCTGATGAC
(tree shrew)          R  E  P   F  Y  F  L   L  A  V   T  K  M   L           G   N  K  N   D  G     T  L  Y  T   P  D  D
Lagomorpha
Oryctolagus          AGGGAACCCT TTTACTTCCT TGTGGCCATC ATGAAGATAC TGGTG>....>TAGGG AGACAAAAAT GATGG---CA CTCTGTATAC CCCAGATGAT
(rabbit)              R  E  P   F  Y  F  L   V  A  I   M  K  I   L           G   D  K  N   D  G     T  L  Y  T   P  D  D
Ochotona             AGAGAACCAT TTTATTTCCT CTTGGCTATC ATGAAGATGT TGGTG>....>TAGGG AGACCAAAGT GATGG---CA CCCTGTACAC TCCAGGGGATC
(pika)                R  E  P   F  Y  F  L   L  A  I   M  K  M   L           G   D  Q  S   D  G     T  L  Y  T   P  G  D
Rodentia
Mus
(mouse)
Heterocephalus                                    ACC ATGAAGATAC TGGGA
(mole rat)                                          T   M  K  I   L
Cavia                CTGGAGGAAC TCTTTTTACT TCCAGCCAGC ATGAAGATAC TGGGA>....>CAGGG AAACAGAAAG GATAATAACA CCTTCTGTAC CCCAAAGGAG
(guinea pig)          L  E  E   L  F  L  L   P  A  S   M  K  I   L           G   N  R  K   D  N  N   T  F  C  T   P  K  E
```

Page C2 (this part of the *IL-15L* alignment also includes pages A2 and B2; the 3' ends continue on page C3)

```
                     100                                                                                              189
Afrotheria
         Procavia    CAGGAGCCTT TTAAATTTCT GGTTGCTATC AAGAAGATTC TGGTG>....>TAGGG AAACAGAAAT GACGG---CA CGCTGTACAC CCCAGATGAT
          (hyrax)     Q  E  P   F  K  F  L   V  A  I   K  K  I   L            G   N  R  N   D  G     T  L  Y  T   P  D  D
        Trichechus
        (manatee)
        Loxodonta    CTCAGATTTT T--------- -------ATC AAGACGATTC TGGTG>....>TAGGG AAACAGAAAT GATGG---CA CGCTCTACAC CCCAGATGAT
       (elephant)     L  R  F    X                 I   K  T  I   L            G   N  R  N   D  G     T  L  Y  T   P  D  D
      Orycteropus    TGGGAGCCTT TCAGATTTCT GGATGCTGTC AAGAAGATTG TGGTG>....>TAGGG AAACAGAAAT TATGG---CA TGCTTTTCCA CCCAGATGAT
        (aardvark)    W  E  P   F  R  F  L   D  A  V   K  K  I   V            G   N  R  N   Y  G     M  L  F  H   P  D  D
Xenarthra
          Dasypus    -GGAATCCTT TCTGATTCCT GTTTGCCATC AAGAAGCTGC CGGTG>....>TTGGA GAACAGAAAC GATGG---CA CTCTCTACAC CCCAGAT---
      (armadillo)    X  N  P   F  *  F  L   F  A  I   K  K  L   P            E   N  R  N   D  G     T  L  Y  T   P  D
Marsupialia
       Monodelphis   TTGGAGCCCT TCCAGGGACT TATCGCTCTC TTGGAGGCCC TTGTG>....>CAGGG TACTAGAAAT GATGG---GA CACTTTATAC TCCGGACAAT
         (opossum)    L  E  P   F  Q  G  L   I  A  L   L  E  A   L            G   T  R  N   D  G     T  L  Y  T   P  D  N
        Macropus     TGGGAGCCAT TCGAGGTCCT TATAGCTATC ATGGAGGCCC TAGTG>....>CAGGG AAATAGAAAT GAGGG---GA CCCTTTATAC TCCGGACAGT
         (wallaby)    W  E  P   F  E  V  L   I  A  I   M  E  A   L            G   N  R  N   E  G     T  L  Y  T   P  D  S
       Sacrophilus
(Tasmanian devil)
Monotremata
   Ornithorhynchus   CGGGAGCCCT TCGACGGCCT GGTCACCATC ACAGAGGCCC TGGTG>....>CAGGG TAACCACAAT GACGG---GA TTCTTTACAC TCCGGACAAC
        (platypus)    R  E  P   F  D  G  L   V  T  I   T  E  A   L            G   N  H  N   D  G     I  L  Y  T   P  D  N
Reptilia: Chelonia
         Chrysemys   AAGGAGGCCA TCAGGGTCCT GCAGAACGTC ACCAAGTTGC TGGTG>....>CAGGG AGACGCCACC GACGG---GA CGCTCTACAC CCCGGAAGAT
   (painted turtle)   K  E  A   I  R  V  L   Q  N  V   T  K  L   L            G   D  A  T   D  G     T  L  Y  T   P  E  D
        Pelodiscus   ATGGAGACCA TCAGGCTCCT GCAGAACATC ACCAAGCTGC TGGTG>....>CAGGG AGACACCACG GATGG---GA TGCTCTACAC CCCAGAAGAC
     (C. s. turtle)   M  E  T   I  R  L  L   Q  N  I   T  K  L   L            G   D  T  T   D  G     M  L  Y  T   P  E  D
Reptilia: Lepidosauria
            Anolis
          (lizard)
            Python
          (python)
```

Page A3 (this part of the *IL-15L* alignment also includes pages B3 and C3; the 3’ ends continue on page A4)

Exon2 | **intron** | **Exon3/4**

```
                 190                                                                                          277
Cetartiodactyla
            Bos CTATTGGTG>....>CAGGTGTGT CCTGCTGAGA CTCTCCGATG CTTCCGG-CT GGAGTTGTCT GTGATCGGGT TTGAGGAG-- -------GGC
       (cattle)  L  L              V  C   P  A  E   T  L  R  C   F  R  L   E  L  S   V  I  G  F   E  E             G
           Ovis CTATTGGTG>....>CAGGTGTGT CCTGCTGAGA CTCTCCGTTG CTTCCGG-CT GGAGCTGTCT GTGATTGGGT TTGAGGAG--- ------GGC
        (sheep)  L  L              V  C   P  A  E   T  L  R  C   F  R  L   E  L  S   V  I  G  F   E  E             G
       Tursiops CTTTTGGTG>....>CAGGTGTGT CCTGCTGAGG CTCTAGGCTA CTTTTGG-CT GGAGCTGTCT GTGATTGGGT TTGAGGAG--- ------GGC
      (dolphin)  L  L              V  C   P  A  E   A  L  G  Y   F  W  L   E  L  S   V  I  G  F   E  E             G
            Sus CTTTCGGTG>....>CAGGTGTGT CCTGCCGAGA CTCTAGGCTG CTTCCGG-CT GGAGCTGTCT GTGATTGGGT TCGAGGAG--- ------GGC
          (pig)  L  S              V  C   P  A  E   T  L  G  C   F  R  L   E  L  S   V  I  G  F   E  E             G
        Vicugna CTCTCGGTG>....>TAGGTGTGT CCTGCTGAGA CTCTAGGCTG CTTCCAG-CT GGAGCTGTCT GTTTTTGGGT TTGAAGAG--- ------GGC
       (alpaca)  L  S              V  C   P  A  E   T  L  G  C   F  Q  L   E  L  S   V  F  G  F   E  E             G
Perissodactyla
          Equus TTCTCGGTG>....>CAGGTGTGT CCTGCGGAGA CGCTAGGCTG CTTTCGG-CT GGAGCTGTCT GTGATAGGGT TCGAAGAG--- ------GGC
          (pig)  F  S              V  C   P  A  E   T  L  G  C   F  R  L   E  L  S   V  I  G  F   E  E             G
   Ceratotherium TTCTCGGTG>....>TAGGTGTGT CCTGCGGAGA CTCTAGGCTG CTTCCGG-CT GGAGCTGTCT GTGATAGGGT TGGAGGAG--- ------GGC
    (rhinoceros)  F  S              V  C   P  A  E   T  L  G  C   F  R  L   E  L  S   V  I  G  L   E  E             G
Chiroptera
         Myotis CTCTTGGTG>....>CAGGTGTGT CCTGCTGAGA CTCCAAGCTG CTTCTGG-CT GGAGCTATGT -----TGGGT TTGAGGAG--- ------GGC
     (brown bat)  L  L              V  C   P  A  E   T  P  S  C   F  W  L   E  L  C   X     G  F   E  E             G
       Pteropus CTCTTGGTG>....>CAGGTGTGT GCTGCTGAGA CTCTAGGCTG CTTCTGG-CT GGAGCTGACT GTGATTGGGT TTGAGGAG--- ------GGC
    (flying fox)  L  L              V  C   A  A  E   T  L  G  C   F  W  L   E  L  T   V  I  G  F   E  E             G
Carnivora
          Felis CTCTCGGTG>....>CAGGTTTGT CCTGCTGAGA CTCTGGGATG CTTCCGG-CT GGAGCTTTCT GTGATCCAGT TCGAAGAG--- ------GGC
          (cat)  L  S              V  C   P  A  E   T  L  G  C   F  R  L   E  L  S   V  I  Q  F   E  E             G
          Canis CTTTCGGTG>....>CAGGTGTGT CCTGCTGAGA CTCTGGGGTG CTTCCGG-CT GGAGCTGTCT GTGATCCAGT TCGAAGAG--- ------GGC
          (dog)  L  S              V  C   P  A  E   T  L  G  C   F  R  L   E  L  S   V  I  Q  F   E  E             G
     Ailuropoda CTCTCGGTG>....>CAGGTGTGT CCTGCTGAGA CTCTAGGGTG CTTCCGG-CT GGAGCTGTCT GTGATCCAGT TCGAAGAG--- ------GGC
        (panda)  L  S              V  C   P  A  E   T  L  G  C   F  R  L   E  L  S   V  I  Q  F   E  E             G
          Ursus CTCTCG            GTGTGT CCTGCTGAGA CTCTAGGGTG CTTCCGG-CT GGAGCTGTCT GTG
         (bear)  L  S              V  C   P  A  E   T  L  G  C   F  R  L   E  L  S   V
       Odobenus CTCTCGGTG>....>CAGGTGTGT CCTGCTGAGA CTCTAGGGTG CTTCCGG-CT GGAGCTGTCC GTGATCCAGT TTG
       (walrus)  L  S              V  C   P  A  E   T  L  G  C   F  R  L   E  L  S   V  I  Q  F   E  E             G
        Mustela CTCTCGGTG>....>CAGGTGTGT CCTGCTGAGA CTCTAGGGTG CTTCCGG-CT GGAGCTGTCT GTGATCCAGT TCGAAGAG--- ------GGC
       (ferret)  L  S              V  C   P  A  E   T  L  G  C   F  R  L   E  L  S   V  I  Q  F   E  E             G
Lipotyphla
     Erinaceus CTCTCGGTG>....>CAGGTGTGT CCTGCAGAGA CGCTGGGTTG CTTCCAG-CT GGAGCTGTCT GTAATTGTGT TTGAGGAG--- ------GAC
     (hedgehog)  L  S              V  C   P  A  E   T  L  G  C   F  Q  L   E  L  S   V  I  V  F   E  E             D
          Sorex TTTTCGGTA>....>CAGGTGTGT CCTGCTGAGA CGCTGGGCTG CTTCCGG-CT GGAGCTGTCT GTGATTGCAT TTGAAGAG--- ------GGC
        (shrew)  F  S              V  C   P  A  E   T  L  G  C   F  R  L   E  L  S   V  I  A  F   E  E             G
```

Page B3 (this part of the *IL-15L* alignment also includes pages A3 and C3; the 3' ends continue on page B4)

```
                     190                                                                                      277
Primata: Strepsirhini
      Microcebus  CTCTCGGTG>....>CAGGTGTGT CCTGCTGAGA CTCTAGGCTG CTTCAGG-CT GGAGCTGTCT GTGATTGGGT TTGAGGAG-- -------GGC
   (mouse lemur)   L  S              V  C   P  A  E   T  L  G  C   F  R  L   E  L  S   V  I  G   F  E  E           G
     Daubentonia  CTCTCGGTG>....>CAGGTGTGT CCTGCTGAGA ATCTAGGCTG CTTCAGG-CT GGAGCTGT-- --GATTGGGT TTGAGGAG-- -------GGC
       (aye-aye)   L  S              V  C   P  A  E   N  L  G  C   F  R  L   E  L  X      *  I  G   F  E  E           G
       Otolemur                  >CAGGTATGT TCTGCTGAGA --CTAGGTTG CTTCAGG-CT GGAGCTGTCT GTAATTGGGT TTGAGGAG-- -------GGC
     (bush baby)                     V  C   S  A  E   X  L  G  C   F  R  L   E  L  S   V  I  G   F  E  E           G
Primata: Haplorhini
         Tarsius  CTCTCTGTG>....>CAGGTATGT CCTGCGGAGA CTCTAGGCTG CTTCAGG-TT GGAGCTATCT GTCATTGGGT TCGAGGAG-- -------GGC
       (tarsier)   L  S              V  C   P  A  E   T  L  G  C   F  R  L   E  L  S   V  I  G   F  E  E           G
      Callithrix  CTCTTGGTG>                                                                   GGAC CATCTGTG-- -------GGA
      (marmoset)   L  L                                                                           G   P  S  V           G
         Saimiri  CTCTTGGTG>....>CT-GTGTGT CCTGCTGAGA TTCTAGGCTG CTTCAGG-CT GGAGCTGTCT GTGATTGGGT TTGGGGAG-- -------GGA
(squirrel monkey)  L  L              V  C   P  A  E   I  L  G  C   F  R  L   E  L  S   V  I  G   F  G  E           G
     Chlorocebus  CTCCCGGTG>....>CT-GTGTGT CCTGCTGAGA CTCTAGGCTG CTTCAGG-TT GGAGCTGTCT GTGATTGGGT TTGAGGAG-- -------GGA
  (vervet monkey)  L  P              V  C   P  A  E   T  L  G  C   F  R  L   E  L  S   V  I  G   F  E  E           G
          Macaca  CTC-CGGTG>....>CT-GTGTGT CCTGCTGAGA CTCTAGGCTG CTTCAGG-TT GGAGCTATCT GTGATTGGAT CTGAGCAG-- -------GGA
       (macaque)   L X               V  C   P  A  E   T  L  G  C   F  R  L   E  L  S   V  I  G   S  E  Q           G
           Papio  CTC-CGGTG>....>CT-GTGTGT CCTGCTGAGA CTCTAGGCTG CTTCAGG-TT GGAGCTATCT GTGATTGGGT CTGAGTAG-- -------GGA
        (baboon)   L X               V  C   P  A  E   T  L  G  C   F  R  L   E  L  S   V  I  G   S  E  *           G
        Nomascus  CTCGCAATG>....>CT-GTGTGT CCTGCTGAGA CTCTAGGCTG CTTCAGG-TT GGAGCTGTCT GTGATTGGGT TTGAGGAG-- -------GGA
        (gibbon)   L  A              V  C   P  A  E   T  L  G  C   F  R  L   E  L  S   V  I  G   F  E  E           G
         Gorilla  CTCTCAGTG>....>CT-GTGTGT CCTGCTGAGA CTCTAGGCTG CTCCAGG-TT GGAGCTGTCT GTGATTGGGT TTGAGGAG-- -------GGA
       (gorilla)   L  S              V  C   P  A  E   T  L  G  C   S  R  L   E  L  S   V  I  G   F  E  E           G
           Pongo  CTCTCAGTG>....>CT-GTGTGT CCTGCTGAGA CTCTAGGCTG CTTCAGG-TT GGAGCTGTCT GTGATTGGGT TTGAGGAG-- -------GGA
     (orangutan)   L  S              V  C   P  A  E   T  L  G  C   F  R  L   E  L  S   V  I  G   F  E  E           G
             Pan  CTCTCAGTG>....>CT-GTGTGT CCTGCTGAGA CTCTAGGCTG CTCCAGT-TT GGAGCTGTCT GTGATTGGGT TTGAGGAG-- -------GGA
    (chimpanzee)   L  S              V  C   P  A  E   T  L  G  C   S  S  L   E  L  S   V  I  G   F  E  E           G
            Homo  CTCTCAGTG>....>CT-GTGTGT CCTGCTGAGA CTCTAGGCTG CTCCAGG-TT GGAGCTGTCT GTGATTGGGT TTGAGGAG-- -------GGA
         (human)   L  S              V  C   P  A  E   T  L  G  C   S  R  L   E  L  S   V  I  G   F  E  E           G
Scandentia
          Tupaia  CTCTCGGTG>....>CAGGTGTGT CCTGCTGAGA CCCTAGGCTG CTTCAGGGCT GGAGCTGTCT GTGATTGGGT TTGAGGAG-- -------GGC
     (tree shrew)  L  S              V  C   P  A  E   T  L  G  C   F  R X L   E  L  S   V  I  G   F  E  E           G
Lagomorpha
     Oryctolagus  CTCTCGGTG>....>CAGGTGTGT CCTGCGGAGA CCCTAGGCTG CTTCAGG-CT GGAGCTGGCT GTGATTGGGT TTGAGGAG-- -------GGC
        (rabbit)   L  S              V  C   P  A  E   T  L  G  C   F  R  L   E  L  A   V  I  G   F  E  E           G
        Ochotona  CTCTCGGTG>....>CAGGTGTGT CCTGTGGAGA GCCTGGGCTG CTTCCGG-CT GGAGCTGGCT GTGATCGGGT TTGAGGAG-- -------GGC
          (pika)   L  S              V  C   P  V  E   S  L  G  C   F  R  L   E  L  A   V  I  G   F  E  E           G
Rodentia
             Mus                 >CAAGTCTGG TCTGCTAAGA ATGTCAGCCA CATCAGG-AT GG-------- ---------T TTGAGGAA-- -------AGC
         (mouse)                     V  W   S  A  K   N  V  S  H   I  R  M   X                       F  E  E           S
 Heterocephalus                  >GAAATGTGT CCTGCTGAGT CTCTAGGCTG CTTCAGG-CT GGAGCTGTCC ATGATTGAGT CTGAGGAT-- -------GGC
      (mole rat)                     M  C   P  A  E   S  L  G  C   F  R  L   E  L  S   M  I  E   S  E  D           G
           Cavia  CTTCCTATG>....>
    (guinea pig)   L  P
```

Page C3 (this part of the *IL-15L* alignment also includes pages A3 and B3; the 3' ends continue on page C4)

```
                  190                                                                                                   277
Afrotheria
         Procavia CTCTCAGTG>....>CAGGTGTGT TCGGTTGAGA CACTAGGCTG CTTCAGG-CT GGAATTGTCT GTGATTGGGT TTGAGGAA-- -------GGT
          (hyrax)  L  S               V  C  S  V  E  T  L  G  C  F  R  L  E  L  S  V  I  G  F  E  E            G
       Trichechus               >CAGGTGTGT CCTGCTGAGA CTCTAGGCTG CTTCAGG-CT GGAGCTGTCT GTGATTGGGT TTGAGGAG-- -------GGT
        (manatee)                     V  C  P  A  E  T  L  G  C  F  R  L  E  L  S  V  I  G  F  E  E            G
        Loxodonta CTCTCAGTG>....>CAGGTGTGT CCTGCTGAGA CTCCAGGCTG CTTCAGG-CT GGAGCTGTCT GTGGTTAGGT TCGAGGAG-- -------AGT
       (elephant)  L  S               V  C  P  A  E  T  P  G  C  F  R  L  E  L  S  V  V  R  F  E  E            S
      Orycteropus CTCTCAGGA>....>CAGGTGTGT CTTGCTGAGA CTCTAGGCTG CTTCAGG-CT GGAGCTGTCT GTGATTGGGG TCAAAGAG-- -------GGA
       (aardvark)  L  S               V  C  L  A  E  T  L  G  C  F  R  L  E  L  S  V  I  G  V  K  E            G
Xenarthra
          Dasypus CTCTGGGGA>....>CAAGTGAGT CCTGCCGAGG CTTGAAGTTG CTTCAGG-CT AGAACTCTCT GTGACTGGGT TCGAGGAG-- --------AGC
      (armadillo)  L  C               V  S  P  A  E  A  *  S  C  F  R  L  E  L  S  V  T  G  F  E  E            S
Marsupialia
      Monodelphis CTCTCTGTG>....>CAGGTGTGC CCCATGGAGA TTCTGCACTG CTTTGGG-GT GGAGCTGTCT GTGATTGGGC ATGAGGAG-- -------GGC
        (opossum)  L  S               V  C  P  M  E  I  L  H  C  F  G  V  E  L  S  V  I  G  F  E  E            G
         Macropus CTCTCTGTG>....>CAGGTGTGC CCGATGGAGA CTCTGCACTG CTTTGGG-GT GGAGCTGTCT GTGATTGGGT ATGAGGAG-- -------GGG
        (wallaby)  L  S               V  C  P  M  E  T  L  H  C  F  G  V  E  L  S  V  I  G  Y  E  E            G
      Sacrophilus               >CAGGTGTGC CCCAAGGAGG TTCTGCACTG CTTTGTC-CT GGAACTGTCT GTGATTGGCT ATGAGGAG-- -------GGT
 (Tasmanian devil)                    V  C  P  K  E  V  L  H  C  F  V  L  E  L  S  V  I  G  Y  E  E            G
Monotremata
  Ornithorhynchus CTCTCTGTG>....>CAGGTGTGC CCGGCCGAGA CGCTGGGCTG CTTCGGG-GC AGAGCTGGGG GTGATCGGCT TGGAGCAC-- ----------
       (platypus)  L  S               V  C  P  A  E  T  L  G  C  F  G  A  E  L  G  V  I  G  L  E  H
Reptilia: Chelonia
        Chrysemys ATCACAGTA>....>CAGGTGTGC ATGGCGGAGA ACCTGAACTG CTTCCAC-AC CGAGCTGCGG GTGATCCAGT GGGAGCAC-- ----------
 (painted turtle)  I  T               V  C  M  A  E  N  L  N  C  F  H  T  E  L  R  V  I  Q  W  E  H
       Pelodiscus ATCACAGTA>....>CAGGTGTGC ACGGTGGAGA ACTTGAACTG CTTCCAC-AC CGAGCTGCAG GTGATCCGGT GGGAGCAC-- ----------
    (C. s. turtle)  I  T               V  C  T  V  E  N  L  N  C  F  H  T  E  L  Q  V  I  R  W  E  H
Reptilia: Lepidosauria
           Anolis               >CAGGTCTGT TATGTTGAAA ACCTGGACTG CTTTGTC-GA GGAATTGGAA GTGGTCCAAG AGGAGGAAGAA GAACATGCG
          (lizard)                    V  C  Y  V  E  N  L  D  C  F  V  E  E  L  E  V  V  Q  E  E  E  E   E  H  A
           Python               >TAGGTCTGC TACGCCGACA ACCTGGACTG CTTCTAC-AT GGAGCTCCGG GTCATCCAGG AGGAGCAGGAA GAACACACC
          (python)                    V  C  Y  A  D  N  L  D  C  F  Y  M  E  L  R  V  I  Q  E  E  Q  E   E  H  T
```

Page A4 (this part of the *IL-15L* alignment also includes pages B4 and C4; the 3’ ends continue on page A5)

Exon3/4

```
                278                                                                                  367
Cetartiodactyla
          Bos   CCATCCGTGG GGATCGTTGT GTTCCGCCTA CAGCGCCTAC TGGATGCCCT GGGGTCCCAG CTGT------ --------GG -GTGATT---
     (cattle)    P  S  V   G  I  V  V   F  R  L   Q  R  L   L  D  A  L   G  S  Q   L                  W   V  I
         Ovis   CCATCCGTGG GGATTGTTGT GTTCCGCCTA CAGCGCCTAC TGGATACCCT GGGGTCCCGG CTGT------ --------GG -GTGACT---
      (sheep)    P  S  V   G  I  V  V   F  R  L   Q  R  L   L  D  T  L   G  S  R   L                  W   V  T
     Tursiops   CCATCCATGG AGACTGCTGT GTTCCGGCTA CAACGCCTAT TGGATGCCCT CAGGTCCTGG CTGT------ --------GG -GTGACT---
    (dolphin)    P  S  M   E  T  A  V   F  R  L   Q  R  L   L  D  A  L   R  S  W   L                  W   V  T
          Sus   CCATTGGTGG GGACTGCTGT GTTCCGGCTG CAGCGCTTAC TGGATGCCCT GGGGTCCCGG CTGT------ --------GG -GTGACT---
        (pig)    P  L  V   G  T  A  V   F  R  L   Q  R  L   L  D  A  L   G  S  R   L                  W   V  T
      Vicugna   CCATCCGTGG GGACTGCTGT GTTCCGGCTA CAGCGCCTAC TGGATGCCCT GGGGTCCCGG CTGT------ --------GG -GTAACT---
     (alpaca)    P  S  V   G  T  A  V   F  R  L   Q  R  L   L  D  A  L   G  S  R   L                  W   V  T
Perissodactyla
        Equus   CCATCTGTGG AAATTGCTGT GTTCCGACTA CAGCGTCTGT TGGATGCCCT GGGGTCCCGG CTGT------ --------GG -GGGACT---
      (horse)    P  S  V   E  I  A  V   F  R  L   Q  R  L   L  D  A  L   G  S  R   L                  W   G  T
 Ceratotherium  CCATCTGTGG GGACTGCTGT GTTCCGGCTA CAGCGTCTGC TGGATGCCCT GGGGTCCCGG CTGT------ --------GG -GGGACT---
  (rhinoceros)   P  S  V   G  T  A  V   F  R  L   Q  R  L   L  D  A  L   G  S  R   L                  W   G  T
Chiroptera
       Myotis   CCATCCGTGG GGACTGCTGT GTTCAGGCTA CAGCGGCAGC TGGATGCCCT GGGGTCCCGG CTGT------ --------GG -GTGACA---
   (brown bat)   P  S  V   G  T  A  V   F  R  L   Q  R  Q   L  D  A  L   G  S  R   L                  W   V  T
     Pteropus   CCATCTGTGG GGACTGCTGT GTTCTGGCTA CAGCGCTTGC TGGATGCTCT GGGGTCCCCG CTGT------ --------GG -GTCACA---
  (flying fox)   P  S  V   G  T  A  V   F  W  L   Q  R  L   L  D  A  L   G  S  P   L                  W   V  T
Carnivora
        Felis   CGATCCATGG GGATTGCTGT GTTCCGGCTA CAGCGTTTGC TGGATGCATT GGGGTCCCGG CTGT------ --------GG -GTGACC---
        (cat)    R  S  M   G  I  A  V   F  R  L   Q  R  L   L  D  A  L   G  S  R   L                  W   V  T
        Canis   CCATCCTTGG GGATTGCGGT GTTCCGGCTA CAGCGTCTGC TGGATGCCCT GGGGTCTCGG CTGT------ --------GG -GTGACT---
        (dog)    P  S  L   G  I  A  V   F  R  L   Q  R  L   L  D  A  L   G  S  R   L                  W   V  T
   Ailuropoda   CCATCCTTGG GGATTGCCGT GTTCCGGCTA CAGCGTCTGC TGGATGCACT GGGGTCCCGG CTGT------ --------GG -GTGACT---
      (panda)    P  S  L   G  I  A  V   F  R  L   Q  R  L   L  D  A  L   G  S  R   L                  W   V  T
        Ursus
       (bear)
     Odobenus   CCATCCTTGG GGATTGCCGT GTTCCGGCTG CAGCGTCTGC TGGATGCACT GGGGTCCCGG CTGT------ --------GG -GTGACT---
     (walrus)    P  S  L   G  I  A  V   F  R  L   Q  R  L   L  D  A  L   G  S  R   L                  W   V  T
      Mustela   CCATCCCTGG GGATTGCCGT GTTCCGGCTA CAGCGTCTGC TGGATGCACT GGGGTCCCGG CTGT------ --------GG -GTGACT---
     (ferret)    P  S  L   G  I  A  V   F  R  L   Q  R  L   L  D  A  L   G  S  R   L                  W   V  T
Lipotyphla
    Erinaceus   CCGACTGTGG TGATTGATGT GTTCCGGTTG CAACGCCTAC TGGGTGCCCT GGGCTCTCGG CTGT------ --------GG -GCCACT---
   (hedgehog)    P  T  V   V  I  D  V   F  R  L   Q  R  L   L  G  A  L   G  S  R   L                  W   A  T
        Sorex   CCCTCCGTGG CACTAGCAGT GTTCCGCCTC CAGCGCCTGC TGGAATCTCT CGGGTCCCGG CTAT------ --------GG -GAGACT---
      (shrew)    P  S  V   A  L  A  V   F  R  L   Q  R  L   L  E  S  L   G  S  R   L                  W   E  T
```

Page B4 (this part of the *IL-15L* alignment also includes pages A4 and C4; the 3' ends continue on page B5)

```
                 278                                                                                          367
Primata: Strepsirhini
     Microcebus  CCATCTGTGG GGACTGCTGT GTTCCGGCTA CAGCGCCTGC TGGATGCCCT GGGGTCCCGG CTGT------ --------GG -GTGGCC---
  (mouse lemur)   P  S  V   G  T  A  V   F  R  L   Q  R  L   L  D  A  L   G  S  R   L                 W    V  A
    Daubentonia  CCCTCTGTGG GGACTGCTGT GTTCCGGCTA CAGCGCTTGC TGGATGCCCT GGGGTCCCGG CTGT------ --------GG -GTGGCC---
      (aye-aye)   P  S  V   G  T  A  V   F  R  L   Q  R  L   L  D  A  L   G  S  R   L                 W    V  A
      Otolemur   CCATCTGTGA GGATTGCTAT GTTCCATCTA CAATGCCTGC TGGATGCC-T GG-------- --GT------ --------GA -GTGGCC---
    (bush baby)   P  S  V   R  I  A  M   F  H  L   Q  C  L   L  D  A  X    X                                *    V  A
Primata: Haplorhini
       Tarsius   CCATCTGTGG GGATTACTGT GTTCCAGCTA CAGCGCCTGC TGGATGTCCT GGGGTCCCGA CTGT------ --------GG -GTGGCC---
      (tarsier)   P  S  V   G  I  T  V   F  Q  L   Q  R  L   L  D  V  L   G  S  R   L                 W    V  A
    Callithrix   CCATCTGTGG GAACTGCTGT GTTCCAGCTA CAGCACCTGC TAGATGCTCT GGGGCTCCAG CACCTGCTAG ATGCTCTGGG -GTGGCC---
    (marmoset)    P  S  V   G  T  A  V   F  Q  L   Q  H  L   L  D  A  L   G  L  Q   H  X              G    V  A
       Saimiri   CCATCTGTGG GAACTGCTGT GTTCCAGCTA CAGCACCTGC TAGATGCTCT GGCCT-CCAG CTGT------ --------GG -GTGGCC---
(squirrel monkey) P  S  V   G  T  A  V   F  Q  L   Q  H  L   L  D  A  L   A  X  Q   L                 W    V  A
   Chlorocebus   CCATCTGTGG GAACTGCTGT GTTGCAGCTA CAGTACCTGC TGGATCCTCT GGGGTCCCAG CTGT------ --------GG -GTGGCC---
(vervet monkey)   P  S  V   G  T  A  V   L  Q  L   Q  Y  L   L  D  P  L   G  S  Q   L                 W    V  A
        Macaca   CCATCTGTGG GAACTACTGT GTTGCAGCTA CAGTACCTGC TGGATCCTCT GGGGTCCCAG CTGT------ --------GA -GTGGCC---
      (macaque)   P  S  V   G  T  T  V   L  Q  L   Q  Y  L   L  D  P  L   G  S  Q   L                 *    V  A
         Papio   CCATCTGTGG GAACTGCTGT GTTGCAGCTA CAGTACCTGC TGGATCCTCT GGGGTCCCAG CTGT---------------GA -GTGGCC---
       (baboon)   P  S  V   G  T  A  V   L  Q  L   Q  Y  L   L  D  P  L   G  S  Q   L                 *    V  A
      Nomascus   CCATCTATGG GAACTGCTGT GTTCCAGCTA CAGTACCTGC TGGATCCCCT GGGGTCCCGG CTGT------ --------GG -GTGGCC---
       (gibbon)   P  S  M   G  T  A  V   F  Q  L   Q  Y  L   L  D  P  L   G  S  R   L                 W    V  A
       Gorilla   CCATCTATGG GAACTGCTGT GTTCCAGCTA CAGTACCTGC TGGATCCCCT GGGGTCCCGG CTGT------ --------GG -GTGGCC---
      (gorilla)   P  S  M   G  T  A  V   F  Q  L   Q  Y  L   L  D  P  L   G  S  R   L                 W    V  A
         Pongo   CCATCTATGG GAACTGCTGT GTTCCAGCTA CAGTGCCTGC TGGATCCCCT GGGGTCCCGG CTGT------ --------GG -GTGGCC---
    (orangutan)   P  S  M   G  T  A  V   F  Q  L   Q  C  L   L  D  P  L   G  S  R   L                 W    V  A
           Pan   CCATCTATGG GAACTGCTGT GTTCCAGCTA CAGTACCTGC TGGATCCCCT GGGGTCCCGG CTGT------ --------GG -GTGGCC---
   (chimpanzee)   P  S  M   G  T  A  V   F  Q  L   Q  Y  L   L  D  P  L   G  S  R   L                 W    V  A
          Homo   CCATCTATGG GAACTGCTGT GTTCCAGCTA CAGTACCTGC TGGATCTCCT GGGGTCCCGG CTGT------ --------GG -GTGGCC---
        (human)   P  S  M   G  T  A  V   F  Q  L   Q  Y  L   L  D  L  L   G  S  R   L                 W    V  A
Scandentia
        Tupaia   CCAACTGTAG -AACTGCTGT GGTCCGGCTA CAGCGCCTGC TGAATGCCCT GGG-TCTCGG CTGT------ --------GG -ATAGCC---
   (tree shrew)   P  T  V   X  T  A  V   V  R  L   Q  R  L   L  N  A  L   GX S  R   L                 W    I  A
Lagomorpha
   Oryctolagus   CCATCTGTGG GAATTGCTGT GTTCCGGCTA CAGCGCCTGC TGGATGCCTT GGGGTCCCGG CTGT------ --------GG -GTAGCC---
       (rabbit)   P  S  V   G  I  A  V   F  R  L   Q  R  L   L  D  A  L   G  S  R   L                 W    V  A
      Ochotona   CCATCTGTGG GAATGGCTGT GTTCCGGCTG CAGCGCCTGC TAGATGCCTT GGGGGCCCGG TTGT------ --------GG -GTGGCC---
         (pika)   P  S  V   G  M  A  V   F  R  L   Q  R  L   L  D  A  L   G  A  R   L                 W    V  A
Rodentia
           Mus   CAGTGGGTAG GGACTGCTGT GTTTCTGTGA CAGTGCTCAC T-GAGGCTCT GAGGTCCTGC TTGC------ --------CA -GTGGCC---
        (mouse)   Q  W  V   G  T  A  V   F  L  *   Q  C  S   LX E  A  L   R  S  C   L                 P    V  A
Heterocephalus   CCATCTGCAG GGGCTTCTGT GTTCTGGCTA CAGTGCCTGT TGGATGCCCT GGCATTCTC- ---------- ---------- ----------
     (mole rat)   P  S  A   G  A  S  V   F  W  L   Q  C  L   L  D  A  L   A  F  S
         Cavia                                                                     CTGT------ --------GC TCTAACC---
   (guinea pig)                                                                     L                 C    X L  T
```

Page C4 (this part of the *IL-15L* alignment also includes pages A4 and B4; the 3' ends continue on page C5)

```
                  278                                                                                      367
Afrotheria
         Procavia CCATCTGTGG GGATTGTTGT GTTGTGGCTG CAGCGACTGC TGGATGTTCT GGGTTCCCAG CTGT------ --------GG -GTGGCC---
          (hyrax)  P  S  V   G  I  V  V   L  W  L   Q  R  L   L  D  V  L   G  S  Q   L                   W    V  A
        Trichechus GCAACTGTGG GGATTATTGT GTTCCGGCTG CAGCGCCTGC TGGATGCTCT GGGGTCCCGG CTGT------ --------GG -GTGGCC---
        (manatee)  A  T  V   G  I  I  V   F  R  L   Q  R  L   L  D  A  L   G  S  R   L                   W    V  A
        Loxodonta CCATCCGTGG GGATTGTTGT GTTCCGGCTG CAGCGCCTGC TGGATGCTCT CGGGTCCCAG CTGT------ --------GG -GTGGCC---
       (elephant)  P  S  V   G  I  V  V   F  R  L   Q  R  L   L  D  A  L   G  S  Q   L                   W    V  A
      Orycteropus CCATCTGTGG GGATTGTTGT GTTGCAGCTG CAGA-CCTGC TAGACGCTCT GGGGTCCCAG CTAT------ --------GG -GTGGCC---
       (aardvark)  P  S  V   G  I  V  V   L  Q  L   Q  X  L   L  D  A  L   G  S  Q   L                   W    V  A
Xenarthra
          Dasypus CTATCCT--G GAGTTTTTGT GTTCTGGTTG CAGCACCTGC TGGATGCCC- GGGGTCCTGG CTGT------ --------GG -GTGGTT---
      (armadillo)  L  S  X   G  V  F  V   F  W  L   Q  H  L   L  D  A  X   G  S  W   L                   W    V  V
Marsupialia
      Monodelphis CTACCAACAG TGAGGGCTGT GAACCGGCTG CAGCGCTCAC TGGGGGCCCT GGGCCCCCAT CTCT------ --------GG -GGGGCCAGG
        (opossum)  L  P  T   V  R  A  V   N  R  L   Q  R  S   L  G  A  L   G  P  H   L                   W    G  A  R
         Macropus CCGGCAGTAG TGAGGGCTGT GATCCGGCTG CAGCGCTCAT TGACAGCCCT GGGGTCCCAC CTGT------ --------GG -GGAGCCAGG
        (wallaby)  P  A  V   V  R  A  V   I  R  L   Q  R  S   L  T  A  L   G  S  H   L                   W    G  A  R
      Sacrophilus CCGCTGACAG GGAGGACTGT GAACCGGCTG AAGCGCTCCT TGGAACAGCT GGCGACCCAC CTGT------ --------GG -GGGCCCCGG
(Tasmanian devil)  P  L  T   G  R  T  V   N  R  L   K  R  S   L  E  Q  L   A  T  H   L                   W    G  P  R
Monotremata
   Ornithorhynchus AGAGAGCAGG CCGTGGAGGT GGCCCGGCTG CAGTGTCACC TGGAGATCCT GGGGTTCCTG CTGC------ --------CT -CCCAGG---
       (platypus)  R  E  Q   A  V  E  V   A  R  L   Q  C  H   L  E  I  L   G  F  L   L                   P    P  R
Reptilia: Chelonia
        Chrysemys AGGGAGCACA CGGAGAGCCT CTCCTTGCTG ATCAGGCATC TGAGCCAACT GGAGAAGCTC ---------- ---------- -------AGG
  (painted turtle)  R  E  H   T  E  S  L   S  L  L   I  R  H   L  S  Q  L   E  K  L                                      R
        Pelodiscus AGGGAGCACA CCAAGAGTCT CTCCTTGCTG ATCAGGAACC TGAGCCAGAT GGAGCAGCTC AAAC------ --------TG -AAGGCCAGG
     (C. s. turtle)  R  E  H   T  K  S  L   S  L  L   I  R  N   L  S  Q  M   E  Q  L   K                   L    K  A  R
Reptilia: Lepidosauria
           Anolis GAGATCCTGT CCCGGCTCCT TCTGCGCCTG CAACAGATGA AGCTGAAATG GCAACGGGAG AATG------ --------GG -ACCAAGCCC
          (lizard)  E  I  L   S  R  L  L   L  R  L   Q  Q  M   K  L  K  W   Q  R  E   N                   G    T  K  P
           Python GAGATCCTTT CCCGGCTCAT TCTGCGCCTG GACCAGCTGA GGAGAAAGCT GAAGGGGACC AGCC------ --------GG -ATCAGC---
          (python)  E  I  L   S  R  L  I   L  R  L   D  Q  L   R  R  K  L   K  G  T   S                   R    I  S
```

Page A5 (this part of the *IL-15L* alignment also includes pages B5 and C5; the 3’ ends continue on page A6)

Exon3/4

```
                 368                                                                                                           459
Cetartiodactyla
          Bos    ---------- --------GA TCAGGGCCCT TGTCCACCCT GCGAAGGACA CCCTCAGAGA CCAGTCCCTC TTTTTCTGGC CAAACTCTTG -
     (cattle)                         D   Q   G  P    C   P  P    C  E   G  H    P  Q   R    P  V   P    L  F   L  A    K  L   L
         Ovis    ---------- --------GG TCAGGGCCCT TGTCCACCCT GTGAAGGACA CCCTCAGAGA CCAGTCCCTC TTTTTCTGGC CAAACTCTTG -
      (sheep)                         G   Q   G  P    C   P  P    C  E   G  H    P  Q   R    P  V   P    L  F   L  A    K  L   L
     Tursiops    ---------- --------GG CTGGGGCCCT TGTCCACCCT GTGGAGGACA CCCTCAGAGA CCCGTCCCTC TTTTTCTGGC CAAACTCTTG -
    (dolphin)                         G   W   G  P    C   P  P    C  G   G  H    P  Q   R    P  V   H    L  F   L  A    K  L   L
          Sus    ---------- --------GG CCAGGGCCCT TGTCCACCCT GTGAAGGACA CCCCCAGAGA CCTGTCCCTC TCTTTCTGGC CAAACTCTTG -
        (pig)                         G   Q   G  P    C   P  P    C  E   G  H    P  Q   R    P  V   P    L  F   L  A    K  L   L
      Vicugna    ---------- --------GG CCAGGGCCAC TGTCCACCCT GTGAAGGACA CCTGCGGAGA CCTGTCCCTC TCTTTTTGGC CAAACTCTTG -
     (alpaca)                         G   Q   G  H    C   P  P    C  E   G  H    L  R   R    P  V   P    L  F   L  A    K  L   L
Perissodactyla
        Equus    ---------- --------GG CCCGGGCCCT TGTCCACCCT GTGAAGGACA TCCCCAGAGA CCTGTCCCTC TCTTTCTGGC CAAACTCTTG -
      (horse)                         G   P   G  P    C   P  P    C  E   G  H    P  Q   R    P  V   P    L  F   L  A    K  L   L
Ceratotherium    ---------- --------GG CCAGGGCCCT TGCCCACCCT GCGAAGGACA TCCCCAGAGA CCTGTCCCTC TCTTTCTGGC CAAACTCTTG -
 (rhinoceros)                         G   Q   G  P    C   P  P    C  E   G  H    P  Q   R    P  V   P    L  F   L  A    K  L   L
Chiroptera
       Myotis    ---------- --------GC CCACGGCCCT TTTCCACCTT GTGAAGAATA TCCTCAGAGA CCAGTCCCTC TTTTCCTGGC CAAACTCTTG -
  (brown bat)                         A   H   G  P    F   P  P    C  E   E  Y    P  Q   R    P  V   P    L  F   L  A    K  L   L
     Pteropus    ---------- --------GC CCAGGGCCTT TGTTTACCCT GTGAAGAATA TCCCCAGAGA CCTGTCTCTC TCTTCCTGGC TAAACTATTA -
 (flying fox)                         A   Q   G  L    C   L  P    C  E   E  Y    P  Q   R    P  V   S    L  F   L  A    K  L   L
Carnivora
        Felis    ---------- --------GG CCAGGGTCCT TGTCCACCCT GTGAAGGACA TCCCCAGAGA CCCGTGCCCC TCTTTCTGGC CAAGCTCTTG -
        (cat)                         G   Q   G  P    C   P  P    C  E   G  H    P  Q   R    P  V   P    L  F   L  A    K  L   L
        Canis    ---------- --------GG CCAGGGCCCT TGTCCACCCT GCGAAGGACA TCCTCAGAGA CCTGTCCCTC TCTTTCTGTC CAAACTCTTA -
        (dog)                         G   Q   G  P    C   P  P    C  E   G  H    P  Q   R    P  V   P    L  F   L  S    K  L   L
   Ailuropoda    ---------- --------GG CCAGGGCCCT TGTCCACCCT GCGAAGGACA TCCTCAGAGA CCTATCCCAC TCTTTCTGGC CAAACTCTTG -
      (panda)                         G   Q   G  P    C   P  P    C  E   G  H    P  Q   R    P  I   P    L  F   L  A    K  L   L
        Ursus
       (bear)
     Odobenus    ---------- --------GG CCAGGGCCCT TGTCCACCCT GCGAAGGACA TCCCCAGAGA CCTGTCCCTC TCTTTCTGGC CAAACTCTTA -
     (walrus)                         G   Q   G  P    C   P  P    C  E   G  H    P  Q   R    P  V   P    L  F   L  A    K  L   L
      Mustela    ---------- --------GG CCAGGGCCCT TGTCCACCCT GCGAAGGACA TCCCCAGAGA CCTGTCCCTC TCTTTCTGGC CAAGCTCTTG -
     (ferret)                         G   Q   G  P    C   P  P    C  E   G  H    P  Q   R    P  V   P    L  F   L  A    K  L   L
Lipotyphla
    Erinaceus    ---------- --------GA CCAGGGCCCT TGTCCACCCT GCGAAGGACA TACCCAAAGG CCTGTCCATC ACTTTCTGAG CAAACTCTTG -
   (hedgehog)                         D   Q   G  P    C   P  P    C  E   G  H    T  Q   R    P  V   H    H  F   L  S    K  L   L
        Sorex    ---------- --------GA CCAGGGCCCT TGTTTGCCCT GTGAGGGACA CCCCCAGAGA CCTGTCCCAC ATTTTCTGGC CAAACTCTTG -
      (shrew)                         D   Q   G  P    C   L  P    C  E   G  H    P  Q   R    P  V   P    H  F   L  A    K  L   L
```

Page B5 (this part of the *IL-15L* alignment also includes pages A5 and C5; the 3' ends continue on page B6)

```
                   368                                                                                459
```

Primata: Strepsirhini

```
        Microcebus ---------- --------AG CCAGGGGCCT TGCCTGCCTT GTGAAGGACA TCCCCAGAGA CCTGTCCCCC GCTTCCTGGC CAAACTCTTG -
     (mouse lemur)                     S   Q  G  P   C  L  P   C  E  G  H   P  Q  R   P  V  P   R  F  L  A   K  L  L
       Daubentonia ---------- --------AG CCAGGGCCCT TGCCTGTCCT GTGAAGGACA TCCCCAGAGA CCTGTCCCCC TGTTCCTGGC CAAACTCTTG -
         (aye-aye)                     S   Q  G  P   C  L  S   C  E  G  H   P  Q  R   P  V  P   L  F  L  A   K  L  L
          Otolemur ---------- --------AT C------CCT TGCCTACCCT GTGAAGGATA TTCCCAGAAA CCTGTCTCCC TCTTCCTGGC CAAAATCTTG -
       (bush baby)                     I         P   C  L  P   C  E  G  Y   S  Q  K   P  V  S   L  F  L  A   K  I  L
```

Primata: Haplorhini

```
           Tarsius ---------- --------AG CCAGGGCCCT TGCCTGCCCT GTGAAGGACA TCCCCAGAGA TCTGTCCCCC TCTTTCTGGC CAAACTCCTG -
         (tarsier)                     S   Q  G  P   C  L  P   C  E  G  H   P  Q  R   S  V  P   L  F  L  A   K  L  L
        Callithrix ---------- --------AG CCAGGGCCTT TGCTTGCCCT GTAAACGACA TCTCCAGA-- -CTGTCCCCC TCTTTCTGGG CAAATTCTTG -
        (marmoset)                     S   Q  G  L   C  L  P   C  K  R  H   L  Q      T  V  P   L  F  L  G   K  F  L
           Saimiri ---------- --------AG CCAGGGCCTT TGCTTGTCCT TTAAACAACA TCCCCAGA-- -CTGTCCCCC TCTTTCTGGG CAAATTCTTG -
 (squirrel monkey)                     S   Q  G  L   C  L  S   F  K  Q  H   P  Q      T  V  P   L  F  L  G   K  F  L
       Chlorocebus ---------- --------AG CAAGGGCCGT TGCCGGCCCT GTGAAGGACA TCCCCAGAGA CCTGTCACCC TCTTGCTGGC CAAACTCCTG -
   (vervet monkey)                     S   K  G  R   C  R  P   C  E  G  H   P  Q  R   P  V  T   L  L  L  A   K  L  L
            Macaca ---------- --------AG CCAGGGCCGT TGCCGGCCCT ATGAAGGACA TCCCCAGAGA CCTGTCCCCC TCTTTCTGGC CAAACTCCTG -
         (macaque)                     S   Q  G  R   C  R  P   Y  E  G  H   P  Q  R   P  V  P   L  F  L  A   K  L  L
             Papio ---------- --------AG CCAGGGCTGT TGCCAGCCCT ATGAAGGACA TCCCCAGAGA CCTGTCCCCC TCTTTCTGGC CAAACTCCTG -
          (baboon)                     S   Q  G  C   C  Q  P   Y  E  G  H   P  Q  R   P  V  P   L  F  L  A   K  L  L
          Nomascus ---------- --------AG CCAGGGCTCT TGCCTGCCCT GTGAAGGACA TCCCCAGAGA CCTGTCCCCC TCTT------ ---------G -
          (gibbon)                     S   Q  G  S   C  L  P   C  E  G  H   P  Q  R   P  V  P   L  L
           Gorilla ---------- --------AG CCAGGGCCTT TGCCTGTCCT GTGAAGGACA TCCCCAGAGA CCTGTCCCCC TCTT------ ---------G -
         (gorilla)                     S   Q  G  L   C  L  S   C  E  G  H   P  Q  R   P  V  P   L  L
             Pongo ---------- --------AG CCAGGGCCCT TGCCTGCCCT GTGAAGGACA TCCCCAGAGA CCTGTCCCCC TCTT------ ---------G -
       (orangutan)                     S   Q  G  P   C  L  P   C  E  G  H   P  Q  R   P  V  P   L  L
               Pan ---------- --------AG CCAGGGCCCT TGCCTGTCCT GTGAAGGACA TCCCCAGAGA CCTGTCCTCC TCTT------ ---------G -
      (chimpanzee)                     S   Q  G  P   C  L  S   C  E  G  H   P  Q  R   P  V  L   L  T
              Homo ---------- --------AG CCAGGGCCCT TGCCTGTCCT GTGAAGGACA TCCCCAGAGA CCTGTCCCCC TCTT------ ---------G -
           (human)                     S   Q  G  P   C  L  S   C  E  G  H   P  Q  R   P  V  P   L  L
```

Scandentia

```
            Tupaia ---------- --------AG CCCGGGGCC- TGGCTGCCCT GCGA-GGACA CCCCCAGAGA CCTGGTCCCA TCTTTCTGTC CAG---CTCT -
      (tree shrew)                     S   P  G  PX  W  L  P   C   X G  H   P  Q  R   P  G  P   I  F  L  S   S     S
```

Lagomorpha

```
       Oryctolagus ---------- --------AG CCAGGGGCCC TGCCTGCCCT GCGAAGGACA TCCCCAGAGA CCCGTCCCCC TCTTTCTGGC CAAACTGTTG -
          (rabbit)                     S   Q  G  P   C  L  P   C  E  G  H   P  Q  R   P  V  P   L  F  L  A   K  L  L
          Ochotona ---------- --------AG CCAGGGTCCC TGCCTGCCTT GCGAAGGACA CCCCCAGAGA CCGGTTCCCC TCTTTCTGGC CAAACTCTTG -
            (pika)                     S   Q  G  P   C  L  P   C  E  G  H   P  Q  R   P  V  P   L  F  L  A   K  L  L
```

Rodentia

```
               Mus ---------- --------A- CCAAGGGCCC TGCCTGCTC- AATAAATACA TCCTCA-ATA AATA-CAGCC T-GTTCTGGC CAAACCCTTA
           (mouse)                     X   Q  G  P   C  L  L X    *  I  H   P  QX        X       X V  L  A   K  P  L
    Heterocephalus ---------- ---------- ---------- ---------- ---------- TCCCCAGAGA CCTGTGGCCC --TCTCTGGC CAAATTCTTG
        (mole rat)                                                          P  Q  R   P  V  A   X  S  L  A   K  F  L
             Cavia ---------- --------A- CAGTGCTTGT TGGCTGCCCC AGAATCCAAC CCAGCGGCGG TTGGTCACCC TCACTCTGGC CAAACTCTTG
      (guinea pig)                     X   S  A  C   W  L  P   Q  N  P  T   Q  R  R   L  V  T   L  T  L  A   K  L  L
```

Page C5 (this part of the *IL-15L* alignment also includes alignment with pages A5 and B5; the 3' ends continue on page C6)

```
                      368                                                                                                  459
Afrotheria
           Procavia   ---------- --------GG CCAGGGCCCT TGTCCACCCT GTGAAAGACA TCCCCAGAGA CCTGTCCCCT TCTTTCTGGC CAAGCTCTTG -
            (hyrax)                     G    Q  G  P    C  P  P    C  E  R  H    P  Q  R    P  V  P    F  F  L  A    K  L  L
         Trichechus   ---------- --------GG CCAGGGCCCT TGTCCACCCT GCGAGAGACA TCCCCAGAGA CCTGTCCCCC TCTTTCTGGC CAAGCTTTTG -
          (manatee)                     G    Q  G  P    C  P  P    C  E  R  H    P  Q  R    P  V  P    L  F  L  A    K  L  L
          Loxodonta   ---------- --------AG CCAGGGCCCT TGCCCACCCT GTGAGAGACA CCCCCAGAGA CCTATCCCCC TCTTTCTGGC CAAGCTCTTG -
         (elephant)                     S    Q  G  P    C  P  P    C  E  R  H    P  Q  R    P  I  P    L  F  L  A    K  L  L
        Orycteropus   ---------- --------AC CCAGGGCTCT GGCCCACCCT GCGAGAAACA TCCCAAAGAAGTCTGTCCCCT TCTTTCTGGC CAAGCTCTTA A
         (aardvark)                     T    Q  G  S    G  P  P    C  E  K  H    P  K  E  X S  V  P    F  F  L  A    K  L  L  X
Xenarthra
            Dasypus   ---------- ---------- ----GGCCCG ------CCCT GTGAGCGACA TCCCCAGACA CCTATCCTCC TCTTCCTGGC TACACTCTTG -
        (armadillo)                                G  P         P    C  E  R  H    P  Q  T    P  I  L    L  F  L  A    T  L  L
Marsupialia
        Monodelphis   GTGGGAGCT- --------GC TCCAGGGCTC TGCCCCCCCT GCGAGGGATA CCCTGAGAGG CCTGTCCCCC AATTCTTGGC CAAACTTCTG -
          (opossum)    V  G  A          A    P  G  L    C  P  P    C  E  G  Y    P  E  R    P  V  P    Q  F  L  A    K  L  L
           Macropus   TGGGGAGCT- --------GC CTCAGGGCTC TGCCCCCGCT GTGAGGAATA CCCCGAGAGG CCTGTCCTCC AGTTCTTGAC CAAACTTCTG -
          (wallaby)    W  G  A          A    S  G  L    C  P  R    C  E  E  Y    P  E  R    P  V  L    Q  F  L  T    K  L  L
        Sacrophilus   GGGGGCGCT- --------GC CTCAGGACCC TGCCCCCTCT GCGAGGGACA CCCCGAGAGG CCCGTGCCCC AGTTCTTGGC CAAACTCCTG -
(Tasmanian devil)      G  G  A          A    S  G  P    C  P  L    C  E  G  H    P  E  R    P  V  P    Q  F  L  A    K  L  L
Monotremata
     Ornithorhynchus  ---------- --------CC ACAGGAGGGC TGCCCGCCCT GCGAGGGACA CCCCGAGCAA CCCACCCCCC GCTTCCTGGC CAAGTTGCTG -
          (platypus)                    P    Q  E  G    C  P  P    C  E  G  H    P  E  Q    P  T  P    R  F  L  A    K  L  L
Reptilia: Chelonia
          Chrysemys   ACCTGCAAG- --------AC GGACCGGCAA TGCCACCCCT GCGAGGGTCA CCGGGAGCAG CCCAAGCCTC AATTCCTGAG CAAGCTCTTG -
   (painted turtle)    T  C  K          T    D  R  Q    C  H  P    C  E  G  H    R  E  Q    P  K  P    Q  F  L  S    K  L  L
         Pelodiscus   ACCTGCAAG- --------AC GGACCGGCCA TGTCATCCCT GCGAGGGCCA CCAGGAGCAG CCTGTGCCTC AATTCTTGAA CAAGCTCCTG -
      (C. s. turtle)   T  C  K          T    D  R  P    C  H  P    C  E  G  H    Q  E  Q    P  V  P    Q  F  L  N    K  L  L
Reptilia
             Anolis   TCCGCCAATG GGGCCAACCC TCCCAACACT TGCCCGCCTT GCCATTCCCA TCCCGAAAGG CCCGTGAAGG ATTTCTTGCA GAAACTCCAG -
           (lizard)    S  A  N   G  A  N  P    P  N  T    C  P  P    C  H  S  H    P  E  R    P  V  K    D  F  L  Q    K  L  Q
             Python   ---------- --------CG CTGTGTTGCC TGCCCGCCTT GCCTGTCTCA CCCAGAAAAG CCCGTGATGA CTTTCCTAAG GAGACTCCTG -
           (python)                     R    C  V  A    C  P  P    C  L  S  H    P  E  K    P  V  M    T  F  L  R    R  L  L
```

Page A6 (this part of the *IL-15L* alignment also includes pages B6 and C6; the 3' end of Tursiops *IL-15L* continues on page A7)

Exon3/4

	460 … 549
**Cetartiodactyla**	
**Bos**	GAGTTATTAC AGGGGACTTG TGCTCGGGAC C----TGCCC TCAGCATAA
**(cattle)**	**E L L Q G T C A R D L P S A ***
**Ovis**	GAGTTATTAC AGGGGACTTG TGCTCAGGAC C----TGCCC TCAGCATAA
**(sheep)**	**E L L Q G T C A Q D L P S A ***
**Tursiops**	GAGTTATTAC AGGGTGCTTG TGCTCAGCAT C----TGCCC TCAGCACGAA CCTGGGAGGT TCGTGGGAAG GATACAGACT CCCTGGTACT
**(dolphin)**	**E L L Q G A C A Q H L P S A R T W E V R G K D T D S L V L**
**Sus**	GAGTTATTAC AGGGGGCTTG TGCTCGGCAC C----TGGCC TCGGCTTGA
**(pig)**	**E L L Q G A C A R H L A S A ***
**Vicugna**	GAGTTATTAC AGGGGGCTTG TGCTCGGCAT C----TGCCC TCAGCATGA
**(alpaca)**	**E L L Q G A C A R H L P S A ***
**Perissodactyla**	
**Equus**	GAGTTATTAC AGGGGGCTTG TGCTCGGCAC C----TGCCC TCAGCATGA
**(horse)**	**E L L Q G A C A R H L P S A ***
**Ceratotherium**	GAGTTATTAC AGGGGGCTTG TGCTCGGCGC C----TGCCC TCAACATGA
**(rhinoceros)**	**E L L Q G A C A R R L P S T ***
**Chiroptera**	
**Myotis**	GAGTTATTAC AGGAAGCTTG TGCTCAGGAC G----TGCCC TCAG-ATAA
**(brown bat)**	**E L L Q E A C A Q D V P S X ***
**Pteropus**	AAGTTATTAC AGGGGGCTTG TGCTTGGCAC C----GGCCC TCAGCATGA
**(flying fox)**	**K L L Q G A C A W H R P S A ***
**Carnivora**	
**Felis**	GAGTTATTAC AGGGGGCTTG TGCTAGGCAC C----TGCCC TCAGCATGA
**(cat)**	**E L L Q G A C A R H L P S A ***
**Canis**	GAGTTATTAC AGGGGGCTTG TGCTCAGCAC C----TGCCC TCAGCATGA
**(dog)**	**E L L Q G A C A Q H L P S A ***
**Ailuropoda**	GAGTTGTTAC AGGGGGCTTG TGCTCGGCAC C----GGCCC TCAGCATGA
**(panda)**	**E L L Q G A C A R H R P S A ***
**Ursus**	
**(bear)**	
**Odobenus**	GAGTTATTAC AGGGGGCCTG TGCTCGGCAC C----GGCCC TCAGCATGA
**(walrus)**	**E L L Q G A C A R H R P S A ***
**Mustela**	GAGTTATTAC AGGGGGCATG TGCTCGGCAC C----GGCCC TCAGCATGA
**(ferret)**	**E L L Q G A C A R H R P S A ***
**Lipotyphla**	
**Erinaceus**	GAGCTGTTAC AGAGGGCTTG TATGCGGCAC C----TATTT TCACCATGA
**(hedgehog)**	**E L L Q R A C M R H L F S P ***
**Sorex**	GAGTTATTAC AGGGGGCATG TGCACACAAC C----TGCTT CTAGCATGA
**(shrew)**	**E L L Q G A C A H N L L L A ***

Page B6 (this part of the *IL-15L* alignment also includes pages A6 and C6)

```
                   460                                                              549
Primata: Strepsirhini
      Microcebus   GAGTTATTAC AGGGAGCTTG TGCTCGGCAC C----TTCGC ACGGCATGA
    (mouse lemur)   E  L  L   Q  G  A  C   A  R  H      L  R   T  A  *
      Daubentonia  GAGTTATTAC AGGGAGCTTG TGCTTGGCAC C----TTCCC ACAGCATGA
        (aye-aye)   E  L  L   Q  G  A  C   A  W  H      L  P   T  A  *
         Otolemur  GAATTATTAT GGGGAGGTTG TGCTCAGC
     (bush baby)    E  L  L   W  G  G  C   A  Q
Primata: Haplorhini
          Tarsius  GAGTTACTCC AAGGAGCCTG TGCTCAGCAC C----TTCCC ACAGCATGA
        (tarsier)   E  L  L   Q  G  A  C   A  Q  H      L  P   T  A  *
      Callithrix   GAGTTACTAC AGGGAGCTTG TGATTGGCAT C----CTACC ACAGCATGA
       (marmoset)   E  L  L   Q  G  A  C   D  W  H      P  T   T  A  *
          Saimiri  GAGTTACTAC AGGGAGCTTG TGATTGGCAC C----CTACC ACAGCATGA
(squirrel monkey)   E  L  L   Q  G  A  C   D  W  H      P  T   T  A  *
      Chlorocebus  GAGTTACTAC AGGGAGCTTG TGCTCAGCAC C----CTA-- -CAGCCTGA
  (vervet monkey)   E  L  L   Q  G  A  C   A  Q  H      P      T  A  *
           Macaca  GAGTTACTAC AGGGAGCTTG TGCTCAGCAC C----CTACC ACAGCCTGA
        (macaque)   E  L  L   Q  G  A  C   A  Q  H      P  T   T  A  *
            Papio  GAGTTACTAC AGGGAGCTTG TGCTCAGCAC C----CTACC ACAGCCTGA
         (baboon)   E  L  L   Q  G  A  C   A  Q  H      P  T   T  A  *
         Nomascus  GAGTTACTAC AGGGAGCTTG TGCTCGGCAC C----CTACC ACAGCCTGA
         (gibbon)   E  L  L   Q  G  A  C   A  R  H      P  T   T  A  *
          Gorilla  GAGTTACTAC AGGGAGCTTG TGCTCGGCAC C----CTACC ATAGCCTGA
        (gorilla)   E  L  L   Q  G  A  C   A  R  H      P  T   I  A  *
            Pongo  GAGTTACTAC AGGGAGCTTG TGCTCGGCAT C----CTACC ACAGCCTGA
      (orangutan)   E  L  L   Q  G  A  C   A  R  H      P  T   T  A  *
              Pan  GAGTTACTAC AGGGAGCTTG TGCTCGGCAC C----CTACC AGAGCCTGA
     (chimpanzee)   E  L  L   Q  G  A  C   A  R  H      P  T   R  A  *
             Homo  GAGTTACTAC AGGGAGCTTG TGCTTGGCAC C----CTACC ACAGCCTGA
          (human)   E  L  L   Q  G  A  C   A  W  H      P  T   T  A  *
Scandentia
           Tupaia  GGAGTAATAC AGGG-GCTTG -GCTCAGCAC TACTGCACCA AGAGCTTGA
      (tree shrew)  G  V  I   Q  GX        X A  Q  H   X    S  P   R  A  *
Lagomorpha
      Oryctolagus  GAATTGTTAC AGGGGGCTTG TGCTCAGCAC C----TTTCC ACAGCATAA
         (rabbit)   E  L  L   Q  G  A  C   A  Q  H      L  S   T  A  *
         Ochotona  GAGTTGTTGC AGGGGGCATG TGCTCAACAA A----CATTC ACAGCATAA
           (pika)   E  L  L   Q  G  A  C   A  Q  Q      T  F   T  A  *
Rodentia
              Mus
          (mouse)
   Heterocephalus  GAGTTATTAC AGGGG-CTTGT GATTGGCAC C----TTCCT GCAGCATGG
       (mole rat)   E  L  L   Q  G X    C   D  W  H      L  P   A  A  W
            Cavia  G-GTTATTAC AGGGTG-TTG TGATTGGCAT C----TTCCT GCAGCATGG
      (guinea pig)  X  L  L   Q  G  X  C   D  W  H      L  P   A  A  W
```

Page C6 (this part of the *IL-15L* alignment also includes pages A6 and B6; the 3’ end of Python *IL-15L* continues on page A7)

```
                   460                                                                                    549
Afrotheria
         Procavia  GAGTTATTAC AATGGGCATG TACTGGACAT T----TGTCA AGAGCATGA
          (hyrax)   E  L  L   Q  W  A  C   T  G  H      L  S   R  A  *
       Trichechus  GAGTTGTTAC AGAGGGCTTG TGCCGGGCAC C----TGCTA GCAGCATGA
        (manatee)   E  L  L   Q  R  A  C   A  G  H      L  L   A  A  *
        Loxodonta  GAGTTATTAC AAGGGGCTTG TGCCCGACAC C----TGCCA GCAGCATGA
       (elephant)   E  L  L   Q  G  A  C   A  R  H      L  P   A  A  *
      Orycteropus  GAGTTATTAC AGGGGAC-TG TGCCAGGTAC C----TGCCA GCAGCATGA
       (aardvark)   E  L  L   Q  G  X C   A  R  Y      L  P  A  A  *
Xenarthra
          Dasypus  GGGTTCCTGC AGGGATCTTG TGCTGGGCAT C----TGTCA GCAGCATGA
      (armadillo)  G  F  L   Q  G  S  C   A  G  H      L  S   A  A  *
Marsupialia
      Monodelphis  GAGCTAATGC AGGTAGCCTG TGCTGGGGCC C----ATCAG GTTGGGTAA
        (opossum)   E  L  M   Q  V  A  C   A  G  A      H  Q   V  G  *
         Macropus  GAGATACTGC ACTTAGCCTG TGCTGGAGCC A----GTACG GCTGGGTGA
        (wallaby)   E  I  L   H  L  A  C   A  G  A      S  T   A  G  *
      Sacrophilus  GAGCTACTGC AGGTTGCCTG TGCTGGAGCC C----ATCGG GCTGGGTGA
(Tasmanian devil)   E  L  L   Q  V  A  C   A  G  A      H  R   A  G  *
Monotremata
  Ornithorhynchus  GAACTGCTGC AGGTCCTCTG TGCTCCCGCC C----ACTGG GGACCCTGA
       (platypus)   E  L  L   Q  V  L  C   A  P  A      H  W   G  P  *
Reptilia: Chelonia
        Chrysemys  GAGCAGCTGC AGTGGGACTG CTGGGTTCAG G----GGAGC AACATGCATT CCTGTCCCTC CTGGCCCGGG TGA
 (painted turtle)   E  Q  L   Q  W  D  C   W  V  Q      G  S   N  M  H   S  C  P  S   W  P  G   *
       Pelodiscus  GAGCTGCTGC AGAGGGAGTG CTGGCTTCAG G----GGAGC AATGTGCATT CCTGTTCATC CTGA
    (C. s. turtle)  E  L  L   Q  R  E  C   W  L  Q      G  S   N  V  H   S  C  S  S   *
Reptilia
           Anolis  CAGTTGTTTC AGGCACAATG CAGGATTGTG G----CCCAA CAGAGAAGAA CCTATGACCT GGTCCCGGTG CCGTGA
          (lizard)  Q  L  F   Q  A  Q  C   R  I  V      A  Q   Q  R  R   T  Y  D  L   V  P  V   P  *
           Python  GAGCTGATGC AGTTCCACTG CAGTATGGGG G----CCCAC CAGCAGAAAG CCTTCTGCCC AACCCCAGTG CCTGAAAACG CCACCCTCCG
          (python)  E  L  M   Q  F  H  C   S  M  G      A  H   Q  Q  K   A  F  C  P   T  P  V   P  E  N   A  T  L  R
```

Page A7 (Tursiops and Python *IL-15L* do not have the family-consensus stop codon)

```
                550                                                                                        639

Cetartiodactyla
     Tursiops  CCTGGAGAGG CTGCGTGGAG AGAGGGAGAG GAGATTCGGT CTTCAGCCCC AGTTCCCAAA CCCCAAGCCT TCTCCTTCTA GGCCAGAGGA
    (dolphin)    L  E  R    L  R  G    E  R  E  R    R  F  G    L  Q  P    Q  F  P  N    P  K  P    S  P  S    R  P  E  D
Reptilia: Lepidosauria
       Python  CACCCAAACG ACTGCTTCTA GTGCCGCGTC TGCCATGGAG GACCAGCAAG TCTAG
     (python)    T  Q  T    T  A  S    S  A  A  S    A  M  E    D  Q  Q    V  *


                640                                   691
Cetartiodactyla
     Tursiops  TCTCACCCAG AGCCCCAGAA GCCAGGGGAA GAGGAGAGAA AGTAAAATAT AA
    (dolphin)    L  T  Q    S  P  R    S  Q  G  K    R  R  E    S  K  I    *
```
