# Supplementary material for: Identification of a gene for an ancient cytokine, interleukin 15-like, in mammals; interleukins 2 and 15 co-evolved with this third family member, all sharing binding motifs for IL-15Rα
Source: Immunogenetics. 2013 Nov 26;66(2):93–103. doi: 10.1007/s00251-013-0747-0 (PMC3894449; doi:10.1007/s00251-013-0747-0)

Supplementary Figure 3 (Fig. S3).
Organization of *IL-15L* transcripts in cattle, rabbit, horse, pig and sheep

Table of Contents:

**Legends to Figures S3*A*-to-*H***		Page 2
**Fig. S3*A***	Schematic presentation of *Bos taurus* (cattle) *IL-15L* RT-PCR products.	Page 6
**Fig. S3*B***	*Bos taurus* (cattle) *IL-15L* genomic sequence and cDNA details	Page 7
**Fig. S3*C***	Schematic presentation of *Oryctolagus cuniculus* (rabbit) *IL-15L* RT-PCR products.	Page 9
**Fig. S3*D***	*Oryctolagus cuniculus* (rabbit) *IL-15L* genomic sequence and cDNA details	Page 10
**Fig. S3*E***	Examples of translation products of *Oryctolagus cuniculus* (rabbit) *IL-15L* splicoforms without exon3/4	Page 11
**Fig. S3*F***	Schematic representation of *Equus caballus* (horse) *IL-15L* RT-PCR products	Page 12
**Fig. S3*G***	Schematic representation of *Sus scrofa* (pig) *IL-15L* RT-PCR products	Page 12
**Fig. S3*H***	Schematic representation of *Ovis aries* (sheep) *IL-15L* RT-PCR products	Page 12

Legends to Figures S3*A*-to-*H*

Organization of *IL-15L* transcripts in cattle, rabbit, horse, pig and sheep.

General note: It took several trial and error experiments (not shown) before we could establish RT-PCR conditions for amplification of *IL-15L* cDNA. This suggests that expression of *IL-15L* is generally low, which agrees with virtual absence in the NCBI EST database. For example, whereas bovine *IL-15* and *IL-2* are represented by five and eight EST reports, respectively, for bovine *IL-15L* there are none.

Fig. S3*A* Schematic presentation of *Bos taurus* (cattle) *IL-15L* RT-PCR products.

(a) Boxes indicate exons with white and gray for coding and non-coding regions, respectively, and numbers above indicate exon lengths. Numbers below represent intron lengths according to genome sequence database information. Boxes with dashed lines represent gene parts that were found in some but not all cDNA clones. Asterisks indicate ATG motifs found in cDNA upstream of the canonical *IL-15L* start codon, with in-frame ATGs that may be used for translation into IL-15L protein above the figure and the other ATGs below the figure.

(b) The various cDNA sequences that were found. The cDNA fragments were amplified by using gene-specific forward plus reverse primers ('PCR'; some of these experiments are shown in Fig. S5), by 3'RACE or by 5'RACE. The boxes correspond with exons indicated above in section (a), and hooked lines indicate a direct connection between those exons. AAAAA, site of oligo-dT primer binding in 3'-RACE experiments. Numbers in front of the depicted cDNA sequences relate to the following primer pairs that were used to amplify them (for primer sequences see Table S1):

1) Cow-IL-15L-5'UTR-F + Cow-IL-15L-3'UTR-R1
2) Cow-IL-15L-ex1-F2 + Cow-IL-15L-ex3-R2
3) Cow-IL-15L-ex1-F2 + Cow-IL-15L-3'UTR-R3
4) Cow-IL-15L-ex1-F1 + Cow-IL-15L-3'UTR-R2
5) Nested PCR first using Cow-IL-15L-5'UTR-3R-F + UPM, then Cow-IL-15L-ex1-F2 + NUP
6) Nested PCR first using Cow-IL-15L-3'UTR-5R-R + UPM, then Cow-IL-15L-ex3-R1 + NUP
7) Nested PCR first using Cow-IL-15L-3'UTR-5R-R + UPM, then Cow-IL-15L-ex1-R + NUP

Fig. S3*B* *Bos taurus* (cattle) *IL-15L* genomic sequence and cDNA details.

The depicted genomic sequence was derived from Ensembl "UMB 3.1" Chr18 positions 49395932-49402591, forward strand. The *IL-15L* ORF sequence agrees with many of our *IL-15L* cDNA clones, and this sequence was used for recombinant expression studies. The sequence has a few nucleotide exchanges compared to NCBI reference NW00149613, which presumably represents another allele of the same locus, and for which we also found matching cDNA clones (not shown). *IL-15L* ORF nucleotides and encoded amino acids which represent the allelic motif of NW001493613 are indicated in purple. Nucleotides found in cDNA clones are shaded blue and green, with green for those that encode protein. The intron borders are in red font. For organization of the individual cDNA clones compare with Fig. S3*A*. The *IL-15L* consensus start codon and ATG motifs in the 5'UTR are shaded yellow. The position to where the 3' end of the poly-T primer connected in 3'RACE experiments is shaded pink, and the possible poly-adenylation motif is shaded orange; it is unclear whether this represents a genuine poly-

adenylation site or that the poly-T primer bound to an immediate downstream transcript-internal A-rich stretch which is present in the genomic DNA sequence (see the figure).

Fig. S3*C* Schematic presentation of *Oryctolagus cuniculus* (rabbit) *IL-15L* RT-PCR products.

Symbols as in Fig. S3*A*. Primer pairs used were:

1) Rab-IL-15L-5'UTR-F + Rab-IL-15L-3'UTR-R1
2) Rab-IL-15L-ex1-F + Rab-IL-15L-ex3-R2
3) Rab-IL-15L-5'UTR-F + Rab-IL-15L-3'UTR-R2
4) Nested PCR first using Rab-IL-15L-5'UTR-3R-F + UPM,
   then Rab-IL-15L-ex3-F + Rab-IL-15L-3'UTR-R2
5) Nested PCR first using Rab-IL-15L-5'UTR-3R-F + UPM, then Rab-IL-15L-ex3-F + NUP
6) Nested PCR first using Rab-IL-15L-5'UTR-3R-F + UPM, then Rab-IL-15L-ex1-F + NUP
7) Nested PCR first using Rab-IL-15L-ex3-5R-R + UPM, then Rab-IL-15L-ex3-R1 + NUP

Fig. S3*D* *Oryctolagus cuniculus* (rabbit) *IL-15L* genomic sequence and cDNA details. The genomic sequence shown was derived from Ensembl "OryCun2" Chr5 positions 532031-537610, complementary strand. Symbols as in Fig. S3*B*. Two different poly-adenylation sites were found. Underlined is a deletion in the 3'UTR that was found in independent cDNA amplifications (Fig. S3*C*) and seems not to agree with GT-AG rule for intron splicing.

Fig. S3*E* Examples of translation products of *Oryctolagus cuniculus* (rabbit) *IL-15L* splicoforms without exon3/4.

Most rabbit *IL-15L* transcripts do not include exon3/4 (Figs. S3*C* and S5*B* middle picture). Two examples of the possible alternative translation products are shown with consensus IL-15L sequence in black font. These alternative *IL-15L* sequences are poorly conserved in *IL-15L* of other species, and we deem it unlikely that they encode functional protein.

Fig. S3*F* Schematic presentation of *Equus caballus* (horse) *IL-15L* RT-PCR products. For horse a nested PCR reaction was needed for *IL-15L* detection, first using primers Gen-IL-15L-ex1-F1 + Gen-IL-15L-ex3-R1, then Horse-IL-15L-ex1-F + Gen-IL-15L-ex3-R2.

Fig. S3*G* Schematic presentation of *Sus scrofa* (pig) *IL-15L* RT-PCR products.

The primer pair used was:

1) Gen-IL-15L-ex1-F1 + Gen-IL-15L-3'UTR-R

Fig. S3*H* Schematic presentation of *Ovis aries* (sheep) *IL-15L* RT-PCR products.

The primer pairs used for separate reactions were:

1) Gen-IL-15L-ex1-F2 + Gen-IL-15L-ex3-R2
2) Cow-IL-15L-5'UTR-F + Sheep-IL-15L-ex3-R

Fig. S3*A* Schematic representation of *Bos taurus* (cattle) *IL-15L* RT-PCR products

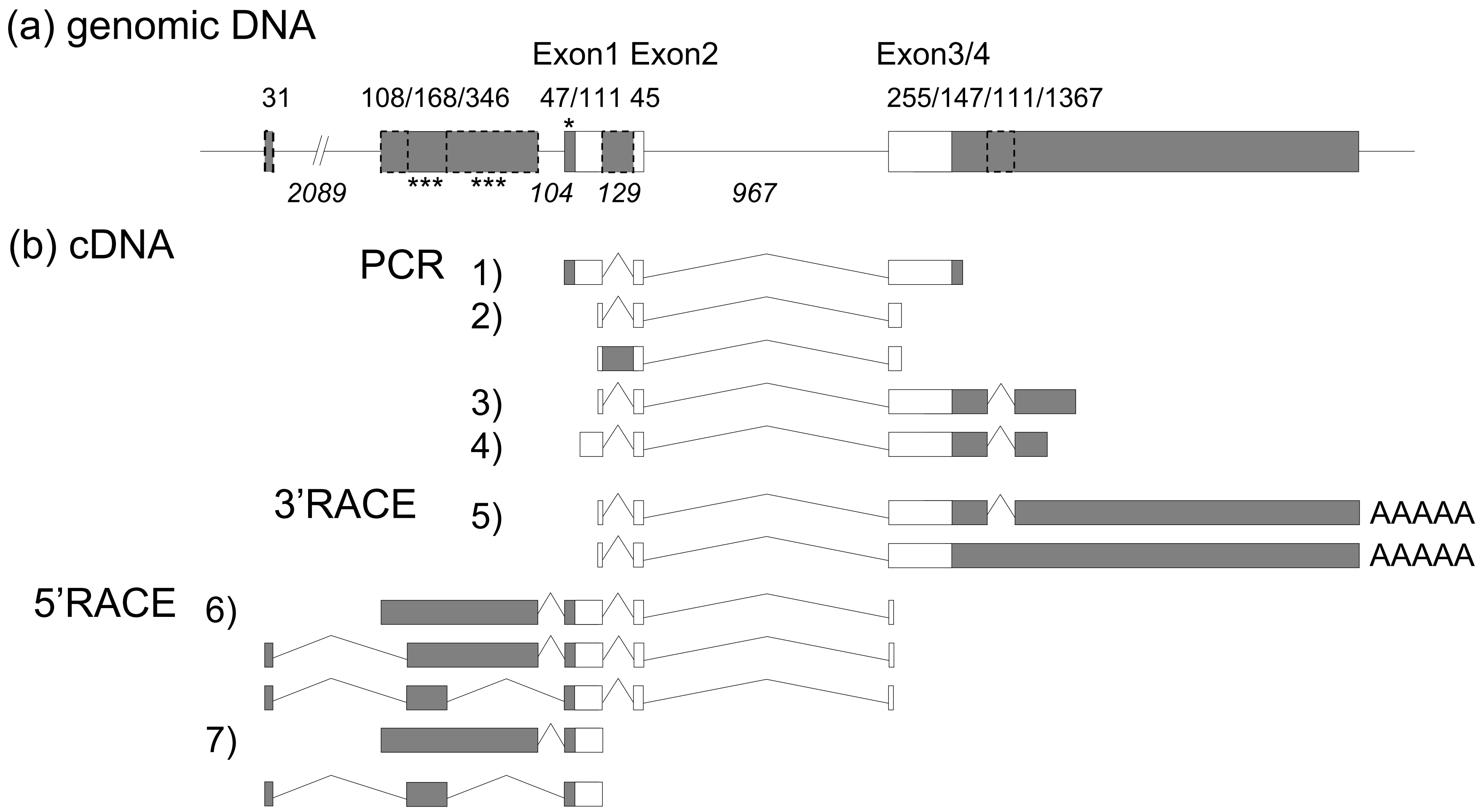

Fig. S3*B* *Bos taurus* (cattle) *IL-15L* genomic sequence and cDNA details

```
   1  TGCGCCACGGCTGTGGCCGTCTTCTGTGGAAAGCGAGGGGGAGACAGCGGAGCCACGTGAGCTCTGGGCCTCGGCTTCCAAGTAGACCCAGATTCCTGCCCACAGACTTCTCTCCGACAC
 121  GGGCCTCCCCTCGTCCCTACCATCGGGCGCCCGTCTCACCTTACGTCCGAAGACTTGCACCGACTGCAGAGGGCCCTTGGACGGCATGGCTCAAGGCGCAGAGACGAGATCCTCACTGCG
 241  CGGCGCCCCAACCGGAAAAGGAAAACGCGGAGCCAACCTGGCCGCTTTTCAGGGTCTGCGCAGGCGCGCTGTGCTCAGCGTTGCGACGGCAGAGAGCTTTACGGCTTGTGGGCGGGACTT
 361  TGGATTGGCGGTAGGAAGGTGGGCCGGGGGAGCGGAGAGGACGGGACCTGGGAGTGGAAGGGACTGACTTCCCTGAACGGTGTCGGGGGCCGGCCCGAGAGGGCGAGTTGTTCAGTCACT
 481  CAGTCGTGTCCGACTCTTTGGGACTCCGTGGACTGCAGCCCCCCAGGCTTCCATGTCTTTCACTATCTCCCGGAGTTTGCTTAAACTCATGTTCATTGAGGCAGTGATCCCATCCAACCA
 601  TCTCATCCTCTGTCGCACCCTTCTCCTGCCTTCAATCTTTCCCAGTATCGGGGTCTTCTCTAATGACCTGGCTCTTCGCATCAGGTGGCCAAAGTATTGGACCTTCAGCTACAGCATCAG
 721  TCTTTCCAATGAATCTTCAGGCTTGATTTCCTTTAGGATTGACTGATTTGATCTTGCTGTTCAAGGGACTCTCAAGAGTCTTCTCCAGTACCACAGTTCCAACGGATCAATTCTTCAATG
 841  CTCAGCCTTCTTTATAGTTGAACTCTCACATCCCTACATGACCAGTGGAAAAACTAGCTTTGACTATACGGACCTTTGTCGTCAAAGTGATGTCTCTGCTTCTTAATACGCTGTCTAGGC
 961  TTGTCATCGGAGAAGGCAATGGCACCCCACTCCAGTACTCCTGCCTGGAAAATCCCATGGGGTCGCTAAGGGTCGGATAGGACTGAGCGACTTCACTTTCACTTTCATGCATTGGAGAAG
1081  GAAATGGCAACCCACTCCAGTGTTCTTGCCTGGAGAATCCCAGGGACGGGGGGAGCCTGGTGGGCTGCACAGAGTCACATAGAGTCGGACACGACTGAAGTGACTTAGCAGCAGCAGCAG
1201  CAGCAGCAGGCTTGTCATAGGTTTTCTTCCAAGGAGAAAGGAGGGTGAAGACAGGCCATAATTCACATCCTCAGTTCTCATTGGCCACCTCCAGCTCTCATTTGCTTTTGGACTCTTCAT
1321  TTATATCCCTTTACTCCCATCCTCAATAGATTCTCATACATACACACTCACCCCTCCCCCCGCACCCCCCTGCTTTCCCACCACCAGGAGATTGCTTCAGAGTTGGCAGTCTTCCCGTCT
1441  TCTAGAATTTCTCCTCAAGAATCCTGGTAGGATTCAGAATCCCCCTCCTCCTAGAACCCATCTCCTTAAAGCATCCTCCCCAGAATCCTTTTCCTCTTAGAGCCCTTTCCCCAGAACCTT
1561  TCACCAAAACTCCTTTCATTTAGGGACTTCCCTGGTGGTCCAGTGGCCAAGACTCTGAGCTCCCAATGCAGGGGCCCAGTTTCTATCCCTGATCAAGGAACTAGATCCCATTTGCCCCA
1681  ATTAAGACCTGGTGCAGTCAAATAAGTAAATAAAAAAATATTAAAGGACTTTCTTCCTTTCAGAGTCTCCTCCCAAAATTCTCTCAGAGCCCTCAACTTAGATTCCCTTTTTGCCTAAAT
1801  CTCCCTCCACTCAGGGTACCCTTACCCCTCTTTCCCCCCAGAATCTTCCTTTCCTCAGAATCCACAGTGCCTAGAATCCCCCTCCTTCAGAAGCTTTCTCTCCCATAATCCACTTTTCCC
1921  TGAACTGCTGCCCCCTCCGAACCTGTTTTCACAAAATCTTCATCCCGGGACTTCCCTGGTGGCTAAGACTCCTTGCTCCCCATGCAGGGGGCCCAGGTTCCATCCCTGGTCTTGAAACTA
2041  GAACTCACATACTGCAATTCATGCACAAAGAAGATCAAAGATCTCCATGTAACTAAGACCTGGCACAGCCAAATAAATTATTTTAAAACAAAAAACCAAACTCTTCATCCTTCTCAGAGC
2161  CCTCTCTCAAAATCTACCTTTCCCAAGAATCCCCAGTCCTCTCACCCCCATCTGAGGAACCTCAAGATAGGGTAGCCCCTTGCTCCCCACTGCTCATGATGCTTGCTCCCTTCCCAGCCA
2281  TGAGTTTCCTTTCAGGTGCAGGCGGCAGTCGGCTGCCTCAGTTTCTCTGGCAGCACTGGGTCTGGGATCCAAGGTTCAAAGGGGTGTGGTGGCTCATGGATGCCTGGAGGGCGGAACCAG
2401  AGTGAAGATTGGTGGGCGGGTCTGGGGCCAAAGGGAGGAACCTGCAGCTCAGAGTCCAGCTCCTAAAGGGTGACAGAGGGAGAGGGCGAGGGGCCGGGGAGACAGGCAGCAAGGACCCAG
2521  AGGCTGACCTGGAGGGACCCACAGACCTACAGGCACACAGCACCAGAGAGAGAGGTGCACATGGAGGAAAAGGCAAAGACTGGACCAACGGAGGAGAGAAAAACGAAGAGAGACATGGGG
2641  TCGATCAGAGATGGAGACAGAAGTCACAAGTGACAGAGGCAGAGGCCAACGCTCAGAGAAAGAAGTTAGCAGGTACAGGCAGGCGTGAAGATTCCAAGACACTTGTGGCAGAAATAGGAG
2761  AAAGGACAGCAAAGACTCCCAAATAAGCACCTGCAGGATTCAAGCTAACTGGTGTTTTCCAGTGGGCAGACAAACAGGCCACAGAATGCACAGGCTGGACACACTCTAGACTGCCTCATG
2881  GGGCTGTTTGGGGGACAGGGGACGCTTCTGAACTCTCCGGTCTCTCCAGGATGATCCTAATAACCTTGCTGAATCTGGTATCTTGATTGGGGGAAAGGAAGGGAAGAGAGTTCTCTGAAC
3001  CACAACCTCCCTACTCTAGGGTCACCATCTCCCCCAGCCTCTGGGAGTACACTTCCAGGTATCTATGGATTGAGTAGAGATGGAAAGGCAGGGACGGGGCATTTCCTCTCCCCCATTCCT
3121  GTTTTCCAACTTGCTCCCTCTCTCTGTACTTCTTGTTTCCAGGAACCCAGGCTGGAACCTGAGAAAGTATGGGGGTGGGCAGGGTGTCCATGTGGCTTCTCTGGACCACCCTCCTGCTGG
                                                                                                 M  G  V  G  R  V  S  M  W  L  L  W  T  T  L  L  L

3241  TGCTGCCCTTGGGAGGCCTAGGACCACTCCTCTGCCCAAGGGAGCCTTTCTACTTCCTCATTGCCATCACGAAGATGCTGGTGAGGAGGGAGGGAAGAGCCAGCAGACAGGGGCAGGCTA
      V  L  P  L  G  G  L  G  P  L  L  C  P  R  E  P  F  Y  F  L  I  A  I  T  K  M  L

3361  GTTAAGGAGTGGGGTGGGTGACATGTTTGCGGAAGGGTCAGTCTCTTCCTCTCCAAGATCTGATTTTCCTCCCTACCCCACAATTACAGGAAAACAAAAATGATGGCAGTCTGTACACCC
                                                                                                                          E  N  K  N  D  G  S  L  Y  T

3481  CAGATAATCTATTGGTGAGTATCATGTTCCCTCTTTACTGGGGGTGGACATTTAGGGAGAATCCACTAGCTCAGGACACACTGGAGGCCAGGAGGCAGGCAGGCTGGTATTCTGGCTGTC
      P  D  N  L  L

3601  CAAGTCTTGACAAATGTATTTTGTTCACAGCTGTCTGTAACACCTAGTACAGTGATTGGCATATAATAGCAACAAAGGATAATAATGACTACAGCTATTATTTTAAATATATATATATTT
3721  ATTTGTTTGGCTGCATTGGGTCTTAGTTATGGCACCTGGAATCTTTCTTTGTGGCACATGGGCTCAGTAGTTGCTTCTCAGCGTGTGGGATCTTATTTCTCAGACCAGGGATCAAAGCCC
3841  CATCCTCTACACAGGAAGGTGGGTACTTAACCACTGGACGGCCAGTGAAGTCCCAATAACAGCTATTCTTACATAATTTCTTACTTGGTGACGGGTGCTGGCCTAAATGCTTTAAGTGTA
3961  TTGGCCTTTTTGGAGGGGGCACGATCTTAGTTCCCTGACCAGTGATTGAACCCTGGGCCCCCAGCAGTGAAAGTGCAAGCGCGGAGTCCTAACCCCTGGACCACCAGGGAAGTCCTAAGC
4081  ACATTAGCTTAATTACCTTCACACTGACCCTCCAAGGTAAACAGGGCTGAGACGTACGGATGTGAAGGTTGTACACTGCACAAGGGTCCCACACCTACAAGGACAGCATTCTCATTCTGG
4201  ATGTTTGTCAGTTTCCAGCACATGGCAGTAAAGTATCTTGTTCTAACAAAATCAGACCGTGGAAGCATTAAGGAAGGAAGATCTTTTTCTAACTTGCACTTCTAGTAAAGGTAGGGATTA
4321  TTCAGAACTCCATTTACAGATGAGGAAACTGAGGCTTAGATTGGTGAAGGGGCTTGTTCCAGGCTACAAGTCAGAAACTGTGGGGGTGAGGACTTGGGATCAGCCCTTGCCCAGAGACAC
```

```
4441  CAGATCACTCTGTCTCCACAGGTGTGTCCTGCTGAGACTCTCCGATGCTTCCGGCTGGAGTTGTCTGTGATCGGGTTTGAGGAGGGCCCATCCGTGGGGATCGTTGTGTTCCGCCTACAG
                            V  C  P  A  E  T  L  R  C  F  R  L  E  L  S  V  I  G  F  E  E  G  P  S  V  G  I  V  V  F  R  L  Q

        T                      G                                         C
4561  CGCCTACTGGATGCCCTGGGGTCCCAGCTGTGGGTGATTGATCAGGGCCCTTGTCCACCCTGCGAAGGACACCCTCAGAGACCAGTCCCTCTTTTTCTGGCCAAACTCTTGGAGTTATTA
       R  L  L  D  A  L  G  S  Q  L  W  V  I  D  Q  G  P  C  P  P  C  E  G  H  P  Q  R  P  V  P  L  F  L  A  K  L  L  E  L  L
                               R                                         A

4681  CAGGGGACTTGTGCTCGGGACCTGCCCTCAGCATAAACTTCAGAGGAAGTCTCCCTGGTACTCCCAAAGAGGCCAGGTGGGGAAGGAGAGGAAACTTGATCTCCAGCCCCAGTTCCTAAA
       Q  G  T  C  A  R  D  L  P  S  A  *

4801  CCCTAAGCCTTCTCCTTCTAGGCCAGAAGACCTTACCTGCAGCCCCAAGAGCGAGGGGAAGAGGTAAGAAGGAAGAAAATAAGGCGAGATGTGAAGCCTGAAATCTCTGACCTACTGGGG
4921  CAGGAAGTTGGATAAATTACCCCACCTTACCCCATTGTTCTCCCTCTCTTTCAGATACTTCCTCCTCTCTCTGCTTCCAGGAAGGGACAATGCACGCTTGTCCTCTAACCTCATCCCCAT
5041  CCCTTGAGGAGAGGAAGTCTTGTGGCTCATATCCCTGGCTACAAATAATCACTGCTTGGAGAACTTTTAAATACTGCTGGTGGCTAGGTCCTAGTCTGGAGAACTTGACAACTGGTCTCT
5161  AGTGAATCCTGAACATCTGTATTTTAAAAGTCAACTTTGTTGAGGAATAATTTACATACAGTAAAAGGAGAAGGAAAGGCAGAGAGAAGGAAATGGCAGCCCACTCCAGTACTCTTGCCT
5281  GGAAAAATCCCATAGATGGAGGAGCCTGGTAGGCTACCGTCTCTGGGGTCACAAAGAGTCGGACACGACTGAGTGAGTTCACTTTCATTTTCTTTCACTTTCAAAAGGTACCCGTTTTTT
5401  AAAAATTATTTATTTCATCGCGCTGTGTCTTAGTTGTGGCACGTGGGATCTAGTTCCCTGACCAGGAACCGTTGCTCCCTGCATTTGGAGCATGGAGTCTCAGCCACTGGACTACTGGAC
5521  TACCAGGGAAACCCCAAAGGTACCCATTTTAAGTGTACGTTTCAAAGGGTTCTGACATATATATATATATATATAGAGAGAGAGAGAGAGAGAGAGAGAGCGCACCACATTTCAATGACCCC
5641  AAAGCAATTCTTTTATGCCTCTTCCCTATCAGTCTCCACCTCTAGTCCTAGAGACTGACTAATCTGATTTATTTGTATAGATTGGTTTTACCTGCTCTAGAATCTCCTATGAATGGAATA
5761  ATACTGTACATACCCTAGTATCTGGCTTCTTTCACTCAGTGGGTGTTTTTGATATTCATTCATGTGCGTTATCAGCGGATTGTTATCATTCCAGTGCATGTATGTGCTGCGACTTGTTCA
5881  TTCCTTCTCCTCATGATAGACATTGGGTTGTTTCCTGTTTGGGGCTACTATGCATATCTCTTATCTTCTCAACTGTTCTCATGTGCTGCCGGATGGAGAGTTGAGAACCACTGATACACC
6001  AAGAAAAAGTTTTCCACAGGGAGAAAATGATGGTTCCCTGTCCTGGAGTAAATGTCTTCGTGCAGAAGTGTCAATAGGCATTTTTATGTGGAATCCCATTCTAAAGAACTGCTGTTTAAA
6121  GGAACCAAGCTTAGGGACTTCGACGGTGGTCCAGTGGCTAAGACTCAGCGTTCCTAGTGCAGGGGGCCTGGGCTCCCTCCCTGGTCAGGGAACTAGGTCCCACAAGCCTCAGCTAAGAGT
6241  TCACAGGCCAGGACTAAATATCCCACATGCTACAACAAAGATTAAGACCCCAAGTGCAGCAACTAAGAACCCCGAGGCCAAATAAGTAAATAAATACATATTTAAAGAAAAACAAAAAAA
6361  CAAAAAAATCGGAAGCAAGCTTATAGAAAAAGAAACTTCTTTGTGTGTGCCACTGTCAGCCCATCAGTAACTCATTCCCCACGTAACTAAATAGGTATATATTAAATTTCTGTAAATTGA
6481  ATCCAGATTTCTCATCGCTTATGTGGGAATTTTAGCAATGTGTTCTAAAAAGCTCCCGATTTTTTTTTTCCCCTCCATCAGGTTAATTTTTATCTCTCCCTGATGCCTGTTCTCTTTGCC
```

Fig. S3*C* Schematic representation of *Oryctolagus cuniculus* (rabbit) *IL-15L* RT-PCR products

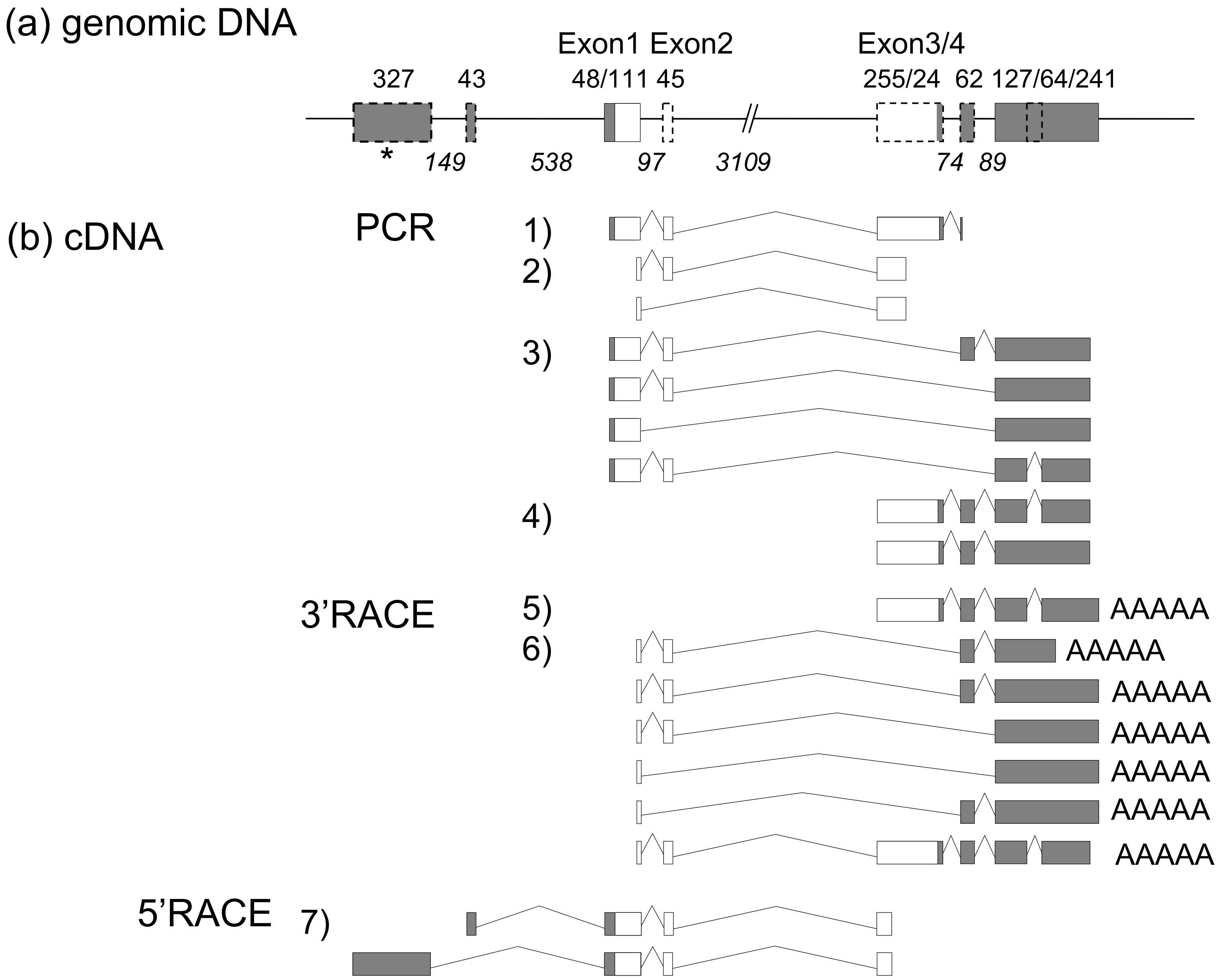

Fig. S3*D* *Oryctolagus cuniculus* (rabbit) *IL-15L* genomic sequence and cDNA details

```
   1  AGCTAGGGGTGGGGCCTCCGAAGGCGGCGGGAAGGCCTCCGGGAGCGCAGGGGGCGGGGTCTGAGCCGCGCCGCGAGCCCGGGAGAAGCATGAGGGCAAGGCGACTCTTCGTAGTTTGCA
 121  TTCTGGTTCCCGCCGCCCCCTTCCATGAGCGATTTTCACTCCCTCCCACCCCCACAGTAATCTTTGTCCCTTCTGGACTGCCTTCTAGAACCCCCGTCAGTCAGAATCTTCGCTCCAGAA
 241  TCCACTGCAAGACTTGAGGCTGGATTCCCAGATCCAGGGTCCCAGACTCCTCCCCTTTCCGAGGCCTCTCCCTCCGAATCTCCCGTTCGCTGGAATTCCTCTCCCGTCCCGGTGCCGCCT
 361  CCCCTTAGGATGCGCCTTTTCTTAGCGACCCTCACCCCGGAACCCCCCTCATCACGGTCCCTACACTCCAGTCTCCGGTTCCCCAGATTCCCCCAAGGCATCTCTACTCTCGCCTCCAAA
 481  TCACTGCACCCTAGGCTCACAGACTCCTGGCCCCGCCTGGAGAACCCTAGAACTGGGTTGCCCTAGATTCCCCTACAGGTGTGCTTCTTCCCCCCCCCCCCCCCGCCCCCCAAGATTCTCT
 601  TCGACCAAGAAGCTCCTCTTTAGGGCCGTAGTCTGCGTTTCTCTGGGGTGCAAGGTTCAGGGGGCGTGGTGGTGACCGGGTCAAGATTGGAGGGCAGGTCTGGGGCCAGAGGGAGGAACG
 721  ATCCTCCAGCCCCGAGTCAGCTCGGAAAGGGTGAGAGAGGGAGAAGGGGTGCGGGAGGGAGGAAGGCAGTGCCAGAGGCAGACCTGCAGGGCCAAACACCCGGAGAAAGAGGAAGAAGGA
 841  AGGGCCGGGGGAGGAGAGGAGGAGAAAAGAGAGGGGGGTCAGCCGGAGGCGGGCTCAGTGCGGAGATTCAGAGGCAGGGAGGCCAGCCCTCAGACGAAAAGGACAGACAGGCTTGCAGGCA
 961  CGACGATTCTGGAGTGCCTGATATAGAAATTAAAAAAAAAAAAAAAAAGCCTCCCAAATATGCACCTGCAGACACAAACCAGGCCTGTGCAGGTATCTTTTAGCAAGACGGACAGGAATGC
1081  TTGCCCTTGCGGAAGTTTGCACCCTCCAGCCCCAACAGGCGGGCCGATATTGCTAAGTCTCTGGGGCCACCATCTCCCCCAACCCTTGGAACAGGTTCTTTGAATATCTATGGCTTGAGT
1201  AGAGCTGGTGGGGGAGTACACAGGGGGCGGCTCCCCTCACCCACTGAAGTCTTTCAACCTGTTCCCTTTCTTTACCTCTTTCTTCCAGAACCACAGGCCGGGGACCAGAGAAAGCCTCGG
1321  AGTGGGCAAGGTGCTCATGTGGCCTCTCTGGACCATCCTCCTGCTTGGGGGACTCTTGGGGGGCCTAGGAACCCCTCTCTGCCGAAGGGAACCCTTTTACTTCCTTGTGGCCATCATGAA
                       M  W  P  L  W  T  I  L  L  L  G  G  L  L  G  G  L  G  T  P  L  C  R  R  E  P  F  Y  F  L  V  A  I  M  K

1441  GATACTGGTGAGGGGAGGCAGGAAGTTGAGGGGGTGAGGCAGTGGGTGTGTTTGGGGGTGGGTCAGACTGAGATCTGATTTTCTTTCTTTCCCCAAAATCGTAGGGAGACAAAAATGATG
        I  L                                                                                                   G  D  K  N  D

1561  GCACTCTGTATACCCCAGATGATCTCTCGGTGAGTGTCTGGTCAACTCCTTCCTGAAGTTGGAAGTTCGGACCTACTGGGCTCCTCATGCATCTGTGGGCGTGTTCTGGAGTGCAGGCAG
      G  T  L  Y  T  P  D  D  L  S

1681  TCGGCAGATGTGGCTGACCCACCCCCACCCTGGACACATAGATTTGTTCCCTCAGAACAGTGACTGGCATAAGATAGCAAGCGATGCTAGTGATGGTTACAGTCACACTTACACAGGGCC
1801  TGCTCCTGCCAGGTGGGAGTCTAAGGAGTGCTTTAGGTAAATTAGCTTATCTACTGGTTTTAAAAAATGGATTTATTATATTTGTTTGAAAAACAGTTACAGAGACAGAGAGGGGTGAAA
1921  CAGAGAGAGAGAGGTCTTCCATCCATTGGTTCACCCCGAGATGACCACAACGGCCAAGGCTGGGCCAGGCCAAAACCAGGGGCCAGGAGCTTCTTCCAGGTCTCCCACACGTGTGGCAGG
2041  GACCCAAGCAGTCGGGCCATTAGCAGGGAGCTGGATTGGAAATGAAGCAGCCAGGGTACGAGCCGGCACCCATGTGGGATGCTGGCATGGCAGGCAGTGGCTTTACCCACTATGCTACAG
2161  TGCCAATTCCTATATGCTGTTTTTAAAATTAGTATTTGTTTTCATTTTGTTTGATTGGCAGAGAGAGAGCTCCTATCTGCTCATTCACTTCCCGAATGCTGTAACAGTCAGGGCTGGGCC
2281  AGGTCAAAGCCCAGCTCCATGCAGATCTCCCACATGGGAGCAGAGACTCAATGACTTGAGCTTTTCCTGCTGCCTCCCAGGGTGCACACCAGCAGGAAGAGGGCACCAGAAGCAGAGCTG
2401  GACGGGGGCCCAGGCTCTCCAGTGTGGGATATGGGTGTCCCAAGCTGTGCCTTAACTGCTGCCCACCATGGCTTACTCACCCTTCATAGCAACCCCACTAGGTTACAGGGCGCCATGTGC
2521  AGCAGCGAAAGATGTGCACTGCACAAGGACCACACACCTACAATGACAGCATTTACAGCTTGGACACTGTAGATTTGCATATGTGTCAGTTTCTGACAATGGCAGCAGAATATCTTATTC
2641  TAATAAGAATCAGCATGTACAGCCGGCACTGTGGCTCACTAGGCTAATCCTCTGCCTGTGGTGCCAGCACCCAGGGTTCTAGTCCCAGTCGGGGCACCGGATTCTGTCCCAGTTGCTCCT
2761  CTTCCAGGCCAGCTCTCTGCTGTGGCCCGGGAGTGCAGTGGAGGATGGCACAAGTGCTTGGGCCCTGCACCCCATGGGAGACCAGGAAGAAGCACCAGGCTCCTGGCTTCGGATCAGCAC
2881  AGCGCGCCGGCCATGGCAGCCATTTGGGGGGTGAAAACGGAAGGAAGATCTTTCTCTCTGTTTTCCTCTAACTCTGCCTGTCAGAAAAAAAAAAAAAAAAAAAAAGAGAATCAACATGTAA
3001  AGGCAAGCCTCTGGCACAGGAGCTAAGGTCCTGTTTGGGATGCCCACATCCCACGCTGGAGTGTCCAAGTTTAAGCCCCTCTTGCCTCCAGCTTCCTGTTACTATGAACCCTGAGAGGCA
3121  GCAGGTGATGGTTCAAACACTTGGGTCCTTGCCATCCACATGGGAGACCTGGATTGAGTCCCAGACTCCTGGCTTTAGCCTTGAGCATTTTGAAGTGAACCAGCAGATGGAAGATCTCTG
3241  CCTGTCAAATAAAAAACAAAACAGCACTCGGACATATTTTTTTAAAAAGACTGATGTACTTATATGAAAGGCAGAATTAGGGAGAGATATTCCATCTGTTGATTCACTCCTCAATTGGCC
3361  ACAATGGCTGGAGCTTGGGGCTGGCACTGTGGCGCAGAGGGGTAAAGCCCCAGCCTGCAGCGCCAGCATCCCAGGTGGGTGCCGGTTCTAGTCCCAGCTGCTCCTCTTTGGATCCAGCTC
3481  TCTGCTATGGCCTGGGAAAGCAGTAGAAGATGACCCAAGTCCTTGGGACCCTGCACCCATGTGGGAGACCTGGTGCAGCTCTGGTCATTGCAACCATCTAGGGAGTGAACCAGCAGATGG
3601  AAAACCTCTCTCTGTGTCTCTGCCTCTCTCTGTAACTCTGTCTTTCAAGTAAATAAAATAAATCTTTAAAAAAAAAAAAAAAAAAAGGCTGGGGCTGGGCCAGGTAGAAGCCAGGAGCCGG
3721  CAGCCTCCAGGTTTCCCATGTGGGTACAGGGGCCCAAGCACCTGGGCCATTCTTCACTGCCTTCCGAGGCACATTAGCAGGGAGCTGGATCAGAAGTGGAACTACTGAGACTTGAACATA
3841  TCTTAAGGAAGGAGTAGGAGTTATTTTTTTCTTTTTTATTTTTTTTTCTAAGATGTATCTATGTTAAAGGCAGAGTTACACAGAGAGAAAGGTAGAGACCAGGAGAGAGAGAGAGCCATC
3961  CTCTGGTTCACTCCCTAAATGGCTACAACAGCTGGGGCTGGGCCAGACAGAAGCTAGGACCTTCATCTGGGTCTCCCATGAGGGTGGAAGGGGTCTGAGCACACGGGCCATCTTCTGCTG
4081  CCTTCCCAGGTGCATTAGCAGGGAGCTAGGTCAGAAGTGAAGCGAGTGGGACCGCCCATGTGGGATGCTGGTGTCACAGGCAGCGGCTTAACCTGCTGTGTGCCACGACACCGGCCCCTA
4201  GGAGAATCTTTATTTTTTTTTTTTAAGATTTATTTATGTTTTACTTGAGAGAGTTACACAGAGAGAGGAGAGGCAGAGAGAGAGAGAGAGAGGTCTTTCATCCGATGGTTCGCTAGGAGAATC
4321  TTTTTTTAACTTGCACACAGGTGTAGTAATAGGCTAGTGCCAGCCCTTGGGGAAGGGATTATTCAAAACCCCACTTTACGGATGAAGAAAGGGCTGTCCGGAGGCACACATCAAAAACCC
4441  TAGGAGGGAGGACTTGGGACCAGATCCTTGCCCAGAGATGCCAGGTCACTCTGTTCCCACAGGTGTGTCCTGCGGAGACCCTAGGCTGCTTCAGGCTGGAGCTGGCTGTGATTGGGTTTG
                                                                     V  C  P  A  E  T  L  G  C  F  R  L  E  L  A  V  I  G  F
4561  AGGAGGGCCCATCTGTGGGAATTGCTGTGTTCCGGCTACAGCGCCTGCTGGATGCCTTGGGGTCCCGGCTGTGGGTAGCCAGCCAGGGGCCCTGCCTGCCCTGCGAAGGACATCCCCAGA
      E  E  G  P  S  V  G  I  A  V  F  R  L  Q  R  L  L  D  A  L  G  S  R  L  W  V  A  S  Q  G  P  C  L  P  C  E  G  H  P  Q
```

```
4681  GACCCGTCCCCCTCTTTCTGGCCAAACTGTTGGAATTGTTACAGGGGGCTTGTGCTCAGCACCTTTCCACAGCATAAGCCTTGGAGGACGGAGACTCCCAGGTACTCCTGGACAGAGGGA
      R  P  V  P  L  F  L  A  K  L  L  E  L  L  Q  G  A  C  A  Q  H  L  S  T  A  *

4801  GGAAACTTGGTCCCCAACGCCAAGTCCCAAACCCTAAAGCCTCTGTTCCTTCTAGGCTAGAGAGCCCCGCCCACAGCGCCAGGAGCCCCGGGGAAGAGGAGAGAAGGGTACCGGGAGGTG
4921  AGCTCTGACGCTGAGGTCTCCGAACTCCTGGGGCACAGGGTCAGGTAGAAGGCTCCCCGCTTCCTCCATTTTCTCTTCTCTTCCAGATACTCCCTCTTCTCTCCTCTCCCGCGAAGGAAT
5041  GGACAGTCCATGGTGTCCTCTAACCTCATCCCCATCCCTGGAGAGCAGGGAGAGTCCTGGGGCCCGGGTCCCTGGCTGCAAATAGTTGCCACCTGGAGAACACTCAGATGACACTGATGG
5161  CCAGGCCCGGGCACAGAAGTTGACAAATGGTCATTGGTGGAGTCTGAGCCGCTGTCTGTTTGAAAATTAACTTTCCTGAGGTACGACTGACAGTAAAAGGCACCCACTGTACACGTGCAC
5281  GTCCGAGAGTTCTGACAGACGTGTGCAGACTTCGGCATCTGCGTCTCCCCCGAGCTCTCCCTCATGCCCTTCCCAGTCTCTGCCGCTAACCCGACACCCACTAATCTGCTGTCTGTCCGT
5401  GCGGATCTGGATTTCACATGAATGGAGTCATACGGTGCATGCCCGGTGTCTGGCTTCTTTGGCTTGGGGAGCGTTGGGGAGAGTCCTTCGTGTGAGCATCAGTTTGTGTCGGTTCCCTGG
```

Fig. S3*E* Examples of translation products of *Oryctolagus cuniculus* (rabbit) *IL-15L* splicoforms without exon3/4

(i) splicing variant without exon3/4 but including the subsequent small exon indicated in Fig S3B by arrow

```
  1   ATGTGGCCTCTCTGGACCATCCTCCTGCTTGGGGGACTCTTGGGGGGCCTAGGAACCCCTCTCTGCCGAAGGGAACCCTTTTACTTCCTTGTGGCCATCATGAAGATACTGGGAGACAAA
       M  W  P  L  W  T  I  L  L  L  G  G  L  L  G  G  L  G  T  P  L  C  R  R  E  P  F  Y  F  L  V  A  I  M  K  I  L  G  D  K

121   AATGATGGCACTCTGTATACCCCAGATGATCTCTCGGCTAGAGAGCCCCGCCCACAGCGCCAGGAGCCCCGGGGAAGAGGAGAGAAGGGTACCGGGAGATACTCCCTCTTCTCTCCTCTC
       N  D  G  T  L  Y  T  P  D  D  L  S  A  R  E  P  R  P  Q  R  Q  E  P  R  G  R  G  E  K  G  T  G  R  Y  S  L  F  S  P  L

241   CCGCGAAGGGATGGACAGTCCATGGTGTCCTCTAACCTCATCCCCATCCCTGGAGAGCAGGGAGAGTCCTGGGGCCCGGGTCCCTGGCTGCAAATAGTTGCCACCTGGAGAACACTCAGA
       P  R  R  D  G  Q  S  M  V  S  S  N  L  I  P  I  P  G  E  Q  G  E  S  W  G  P  G  P  W  L  Q  I  V  A  T  W  R  T  L  R

361   TGA
       *
```

(ii) splicing variant without exon3/4 and without the subsequent small exon indicated in Fig S3B by arrow

```
  1   ATGTGGCCTCTCTGGACCATCCTCCTGCTTGGGGGACTCTTGGGGGGCCTAGGAACCCCTCTCTGCCGAAGGGAACCCTTTTACTTCCTTGTGGCCATCATGAAGATACTGGGAGACAAA
       M  W  P  L  W  T  I  L  L  L  G  G  L  L  G  G  L  G  T  P  L  C  R  R  E  P  F  Y  F  L  V  A  I  M  K  I  L  G  D  K

121   AATGATGGCACTCTGTATACCCCAGATGATCTCTCGATACTCCCTCTTCTCTCCTCTCCCGCGAAGGGATGGACAGTCCATGGTGTCCTCTAA
       N  D  G  T  L  Y  T  P  D  D  L  S  I  L  P  L  L  S  S  P  A  K  G  W  T  V  H  G  V  L  *
```

Fig. S3*F*

Equus caballus (horse) *IL-15L*

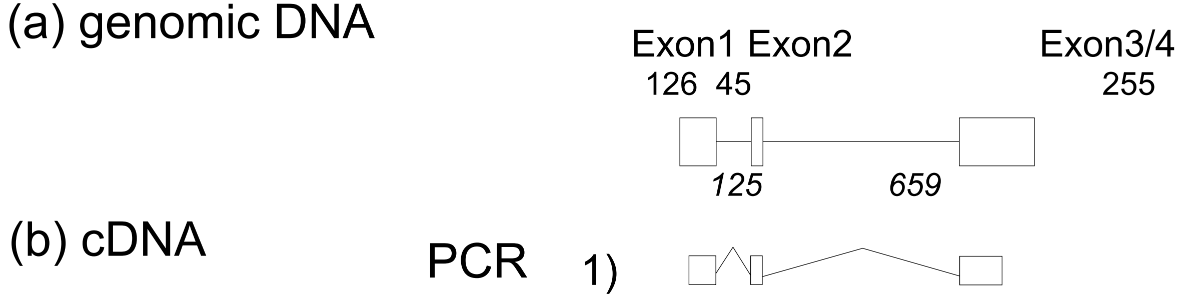

Fig. S3*G*

Sus scrofa (pig) *IL-15L*

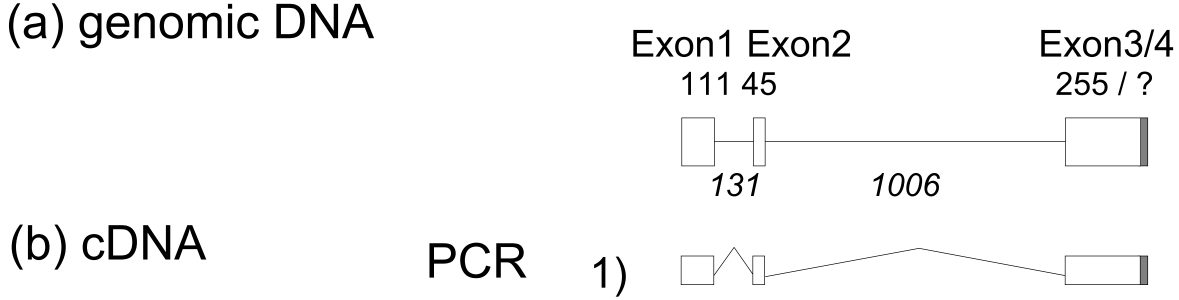

Fig. S3*H*

Ovis aries (sheep) *IL-15L*

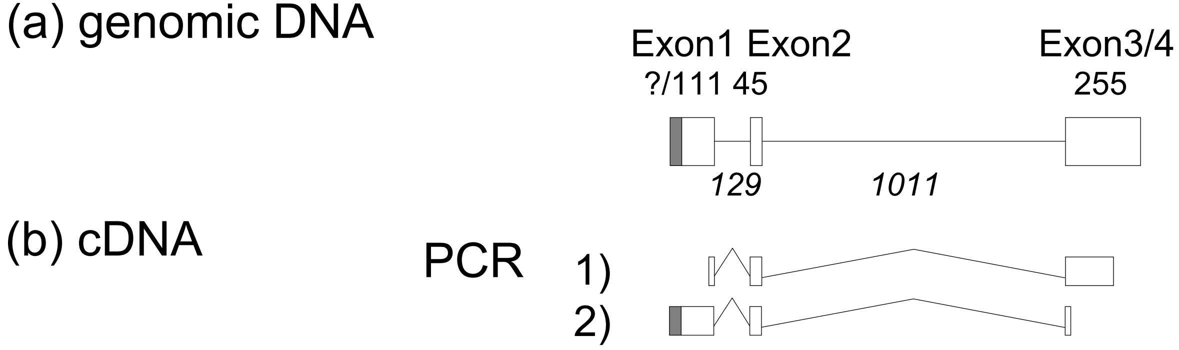

Supplement: Supplementary file 6 — (PDF 305 kb) [file 251_2013_747_MOESM6_ESM.pdf]
