# Supplementary material for: Identification of a gene for an ancient cytokine, interleukin 15-like, in mammals; interleukins 2 and 15 co-evolved with this third family member, all sharing binding motifs for IL-15Rα
Source: Immunogenetics. 2013 Nov 26;66(2):93–103. doi: 10.1007/s00251-013-0747-0 (PMC3894449; doi:10.1007/s00251-013-0747-0)
Supplement: Supplementary file 7 — (PDF 292 kb) [file 251_2013_747_MOESM7_ESM.pdf]

Supplementary figure 4 (Fig. S4).
Organization of *IL-15L* pseudogene transcripts in bear, dog and human

Table of Contents:

**Legends to Figure S4*A*-to-*E***		Page 2
**Fig. S4*A***	Alignment of *IL-15Lψ* genomic and cDNA sequences known for *Canis familiaris* (dog), *Ailuropoda melanoleuca* (giant panda bear) and *Ursus americanus* (American black bear)	Page 5
**Fig. S4*B***	Translated *Ursus americanus* (American black bear) *IL-15Lψ* cDNA (EST GW294330)	Page 8
**Fig. S4*C***	Translation of predicted "full-length" *Canis familiaris* (dog) *IL-15Lψ* cDNA	Page 9
**Fig. S4*D***	*Homo sapiens* (human) hybrid *IL-15Lψ-SUPT5H* cDNA sequence (EST DC400386) indicated in green font within the genomic region *RPS16*-to-*SUPT5H*	Page 11
**Fig. S4*E***	*Homo sapiens* (human) hybrid *IL-15Lψ-SUPT5H* sequence (EST DC400386) with translation products	Page 15

Legends to Figures S4*A*-to-*E*

Organization of *IL-15L* pseudogene transcripts in bear, dog and human.

Although it is not certain, bear and dog *IL-15L* most likely cannot express intact IL-15L protein and therefore are denoted in this figure as pseudogenes.

Fig. S4*A* Alignment of *IL-15L* genomic (g-) and cDNA (c-) sequences known for *Canis familiaris* (dog), *Ailuropoda melanoleuca* (giant panda bear) and *Ursus americanus* (American black bear).

Dog, giant panda bear and American black bear belong to the suborder Caniformia and have the *IL-15L* consensus start codon replaced by GTG. c-dog, our reported cDNA sequence at GenBank JX271586 which we amplified with the primer set Dog-IL-15L-5'UTR-F + Dog-IL-15L-3'UTR-R. g-dog and g-panda, dog and panda genomic sequences are from the Ensembl database (Fig. S2). c-bear, the EST at GenBank GW294330.

Puzzlingly, the sequence of *Mustela putorius furo* (domestic ferret), a species which is commonly thought to form a phylogenetic group with bears apart from canines [e.g. reference 1], does not have the ATG-to-GTG replacement (Fig. S2).

Reference in this figure legend:

[1] Meredith R-W, et al. (2011) Impacts of the Cretaceous Terrestrial Revolution and KPg extinction on mammal diversification. *Science* 334(6055):521-534.

Fig. S4*B* Translated *Ursus americanus* (American black bear) *IL-15L*ψ cDNA (EST GW294330).

Possible translation products of the black bear EST sequence.

Fig. S4*C* Translation of predicted “full-length” *Canis familiaris* (dog) *IL-15Lψ* cDNA. This figure shows a possible translation product of an assembly of known dog *IL-15L* cDNA sequence and upstream dog genomic sequences which match with the black bear *IL-15L* EST. The translation product of this “full-length” dog cDNA is similar to the automated, genomic sequence based prediction for panda IL-15L molecule at GenBank XP_002927443 (not shown). Unless the discussed GTG codon (here in green) is used for start codon, which is very unlikely [reference 1], the possible dog and bear IL-15L molecules are probably non-functional because they seem to lack proper leader peptides by either lacking the consensus leader region (if translation starts downstream) or adding a non-hydrophobic N-terminal region (if translation starts upstream).

Reference in this figure legend:

[1] Touriol C, et al. (2003)Generation of protein isoform diversity by alternative initiation of translation at non-AUG codons. *Biol Cell* **95:** 169-178.

Fig. S4*D* *Homo sapiens* (human) *IL-15Lψ-SUPT5H* cDNA sequence (EST DC400386) indicated in green font within the genomic region *RPS16*-to-*SUPT5H.*

The depicted genomic region corresponds to Ensembl “GRCh37” Chr19 positions 39925796-39942756, forward strand. The human *IL-15Lψ* exons (all incapacitated except for exon2, see Figs. S1*B* and S2) are shaded gray, while coding exons of *RPS16* and *SUPT5H* are shaded pink and yellow, respectively. The sequence corresponding with human EST DC400386 is shown in green. Intron borders are shown in red font. The DC400386 sequence ends close to the transcriptional start point of the upstream *RPS16* gene (GenBank NM_001020) and overlaps with exons of the downstream *SUPT5H* gene. The existence of *IL-15Lψ-SUPT5H* hybrid transcripts was confirmed by

our preliminary RT-PCR experiments (not shown), for which we used the the primer sets Human-IL-15L-F1 + Human-SUPT5H-R and Human-IL-15L-F2 + Human-SUPT5H-R (indicated by arrows). In addition to a sequence matching EST DC400386, we found some other splice variants, as explained in the figure. We deem it very unlikely that these transcripts encode functional IL-15L protein, as exemplified by the possible translation products for EST DC400386 shown in Fig. S4*E*. Our primary interest concerns the immune system, and because these human transcripts probably do not have immune functions, we did not analyze them in further detail. Despite various efforts (not shown), we could not confirm the existence of the human *IL-15L* "mRNA" sequence reported in GenBank DQ059386, and leave it to the authors of that GenBank accession to further discuss their report.

Fig. S4*E* *Homo sapiens* (human) *IL-15Lψ-SUPT5H* sequence of EST DC400386 with translation products.

It appears very unlikely that the *IL-15Lψ-SUPT5H* transcripts are used for production of IL-15L sequence containing protein.

Fig. S4*A* Alignment of *IL-15Lψ* genomic (g-) and cDNA (c-) sequences known for *Canis familiaris* (dog), *Ailuropoda melanoleuca* (giant panda bear) and *Ursus americanus* (American black bear)

```
c-dog      0:------------------------------------------------------------ 0
g-dog      1:TGGGTGGAGGTTAGTGGGCGGATCTGTGGCTGGAGGGAGAATCCTCCAGCCCAGAGTCCA 60
g-panda    1:TGGGTGGAGGTTTGTGGGCGGGTCTGTGGCAGGAGGGAGGATCCTCCAGCCCAGAGTCCA 60
c-bear     0:------------------------------------------------------------ 0

c-dog      0:------------------------------------------------------------ 0
g-dog     61:GCTCCAACAAGGTGAGAGAGAGGAGAGGTGGAGGGAGGGAGCACCCAGACGCTGACTTGG 120
g-panda   61:GCTCCAAAAGGGTGAGAGAGGGGAGAGGTGGAGGGAGGGAGCACCCAGAGGCTGACCTGG 120
c-bear     1:-------------------GGGGAGAGGTGGAGGGAGGGAGCACCCAGAGGCTGACCTGG 41

c-dog      0:------------------------------------------------------------ 0
g-dog    121:GGGGACAGACAGACA--TACAAAGCACAAGAGAGACAGACACACATGAAGGAGAAAGACA 178
g-panda  121:GGGGACACACAGACAGATACACAGCACAAGAAAGACAGACACACATGAAGGAGAAAGACA 180
c-bear    42:GGGGACACACAGACAGATACACAGCACAAGAGAGACAGACACACATGAAGGAGAAAGACA 101

                         out of frame for black bear, but would be in frame
                         for similarly spliced dog or panda transcripts

c-dog      0:------------------------------------------------------------ 0
g-dog    179:AAGAAGGGGCCAATGGAGAAGAGGAAAAAGAAGATA-GACACAGGGTCAATTACAGATGG 237
g-panda  181:AAGAAGGGGCCAATGGAGAAGAGGAGAAAGAAGATA-GACACAGGGGCAATTACAGATGG 239
c-bear   102:AAG---GGGCCAATGGAGAAGAGGAGAAAGAAGATAAGACACAGGGGCAATTACAGATGG 158

                  in frame for black bear                      out of frame
                          frame-shift dog and panda versus black bear

c-dog      0:------------------------------------------------------------ 0
g-dog    238:ACCCAGAAAACACAAGTGACGGCGATTCGGAGGCAGAGAGGCCAGCCCTCAGAGAACGAA 297
g-panda  240:ACCCAGAAAACACAAGTGACGGCGATTCGGAGGCAGAGAGGCCAGCCCTCAGAAAAAGAA 299
c-bear   159:ACCCAGAAAACACAAGTGACGGCGATTCGGAGACAGAGAGGCCAGCCCTCAGAGAAAGAA 218

c-dog      0:------------------------------------------------------------ 0
g-dog    298:GACAGACAGGTACAGGCAAGCATGGAGATTTCAAGATATTTGAAATATGGAGAAACTTGA 357
g-panda  300:GACAGACAGGTACAGGGAAGCATGGAGATTCCAAGTTATTTGAAATATGGAAATACTTGA 359
c-bear   219:GACAGACAG--------------------------------------------------- 227

c-dog      0:------------------------------------------------------------ 0
g-dog    358:TGTAGGAATAGAAGTAGAAGGGAGAGCAGATCCCC--AAATATGCACCTGCAAACACAGA 415
g-panda  360:TACAGAAATAGAAGTAGAAAGGACAGCAAAGCCCCCCAAATATGCACCTGCAAACACAGA 419
c-bear   227:------------------------------------------------------------ 227

c-dog      0:------------------------------------------------------------ 0
g-dog    416:TAAAGCCTAGCAGGGGTGTTTTCCAATGAGAAGACAGGCCATGGGATTCACGTACTCACA 475
g-panda  420:TAAAGCCTAGCAGGGGTGTTTTCCAACGAGAAGACAGGCCACGGAATTCATGCACTCTCA 479
c-bear   227:------------------------------------------------------------ 227

c-dog      0:------------------------------------------------------------ 0
g-dog    476:CACTCT-TATGCTGCCTGATGCACCTGTTGGGGGAGGAGGGTGGGATTAGACTTCCCAAA 534
g-panda  480:CACACTCTGTACTGCTTTATGGACCTCTTGGGGGAGG-GGGTGGGATGGGACTTCCCCAT 538
c-bear   227:------------------------------------------------------------ 227

c-dog      0:------------------------------------------------------------ 0
g-dog    535:ATCCTCTAATCTCTCTGGGCTAATCCTCATACGCTTGCTGAGTCTTTTATCTTCCTGTGT 594
g-panda  539:ACCCTCCAATCTCTCTGGACTGATCCTCCTACCCTTGCTGAGTCTTATATCTTCCTGTGG 598
c-bear   227:------------------------------------------------------------ 227

c-dog      0:------------------------------------------------------------ 0
g-dog    595:GTG-TGTGTGTGTGGGGGGGGTGTCAGGGAGCCCTCTGACCTGGACCCTCCCTACTGTAG 653
g-panda  599:GGGGTGGGGGTGGGGGAGAGGTGTCAGGAAACCCTCTGACCTGGACCCTCCCTACTGTAG 658
c-bear   227:------------------------------------------------------------ 227

c-dog      0:------------------------------------------------------------ 0
g-dog    654:CATGACCATCTTCCCTGGCCTCTTGAAGAACATTTTCTGAGTATCTGTGGGTTGAGTGGA 713
g-panda  659:GGTGACCATCTTCCCCGGCCTCTTGGAGAACATTCCCTGAGTATCTGTGGGTTGAGTGGA 718
c-bear   227:------------------------------------------------------------ 227
```

```
c-dog      0:------------------------------------------------------------ 0
g-dog    714:GCTGGACCAGAAGGGGTCAGGACACTTCAGTCTTCCAACTTGCTCTGTCTCTTTGCACTT 773
g-panda  719:GCTGGGCAGGAAGGGGTCAGGACACTTCAGTCTTCTAACTTGCTCTGTCTTTCTGTACTT 778
c-bear   227:------------------------------------------------------------ 227

c-dog      1:----------------------------------------------GGGCAGGGTGCCCG 14
g-dog    774:CTTGCTTCCAGGACCCTGGGCTGGGACCAGAGAAAGCCTGGGTGGTGGGCAGGGTGCCCG 833
g-panda  779:CTTGCTTCCAGGGCGCTGGGCTGGGACCAGAGAAAGCCTTGGGAGTGGGCAGGGTGCCCG 838
c-bear   228:-----------GACGCTGGGCTGGGACCGGAGAAAGCCTTGGGAGTGGGCAGGGTGCCCG 276

c-dog     15:TGTGGCCTCTCTGGACCATCCTCCTGCTGGTACGGCCCTTGGGAAGCCTAGGATCACCCC 74
g-dog    834:TGTGGCCTCTCTGGACCATCCTCCTGCTGGTACGGCCCTTGGGAAGCCTAGGATCACCCC 893
g-panda  839:TGTGGCCTCTCTGGACCATCCTCCTGCTGGTACGGCCCTTGGGAGGCCTAGGATCACCCC 898
c-bear   277:TGTGGCCTCTCTGGACCATCCTCCTGCTGGTACGGCCCTTGGGAGGCCTAGGATCACCCC 336

 IL-15L consensus ATG start codon modified into GTG

c-dog     75:TCTGTCCTCGGGAGCCTTTCTACTTCTTAATTGCCATCATGAAGATGCTG---------- 124
g-dog    894:TCTGTCCTCGGGAGCCTTTCTACTTCTTAATTGCCATCATGAAGATGCTGGTGAGGAGGA 953
g-panda  899:TCTGTCCTCGGGAGCCTTTCTACTTCCTTGTTGCCATCATGAAGATGCTGGTGAGGAGGA 958
c-bear   337:TCTGTCCTCGGGAGCCTTTCTACTTCCTTGTTGCCATCATGAAGATGCTG---------- 386

                              translation from these ATG codons would result in
                              IL-15L protein without leader peptide (see below)

c-dog    124:------------------------------------------------------------ 124
g-dog    954:GGGAGGGGA----GAGC-CAGCAGATAGGCAGGGAGGGGGTTGGGGGGGGCATGTTTGAA 1008
g-panda  959:GGGAGGGAA----GAGC-CAGCAGGCAGGCAGGGAGGGGGT---GGGGGGCATGTTTGAA 1010
c-bear   386:------------------------------------------------------------ 386

c-dog    125:--------------------------------------------------------GGAA 128
g-dog   1009:GAAGGGTCCGGCTTTCCCCTTCAAGATCTGATTCTTCTCCCTGCCTCAAATTATAGGGAA 1068
g-panda 1011:GAAGGGTCAGGCTCTCCCCTCCAAAATCTGATCCTTCTCCCTGACCCAAATTATAGGGAA 1070
c-bear   387:--------------------------------------------------------GGAA 390

c-dog    129:ACAAAAATGATGGCACTCTTTACACCCCAGATGACCTTTCG------------------- 169
g-dog   1069:ACAAAAATGATGGCACTCTTTACACCCCAGATGACCTTTCGGTGAGTATTTCATTAGCTC 1128
g-panda 1071:ACAAAAATGATGGCACTCTCTACACCCCAGATGATCTCTCGGTGAGTATTTCATTAGCTC 1130
c-bear   391:ACAAAAATGATGGCACTCTCTACACCCCAGATGATCTCTCG------------------- 431

c-dog    169:------------------------------------------------------------ 169
g-dog   1129:CT---TAC--CAGGCGTTGGAAATTTAGGGAGAATCCACTAG-CTCAGAATACATGTTGG 1182
g-panda 1131:CT---TAC--TGGACCTTGGAAGTTTAGGGAGAATCCACTAGGCTCAAAACACACGTTGG 1185
c-bear   431:------------------------------------------------------------ 431

c-dog    169:------------------------------------------------------------ 169
g-dog   1183:CGGCAGGAGGCAGTAGGGCTGGTGTT-TGGCTGTCCAGCTGCTGACCCAGCTCCCAGCCT 1241
g-panda 1186:GGGCAGGAGGCAGTGGGGCTGGTATTCTGGCTGTCCAGCTGCTGACCCAACTCCCAGCCT 1245
c-bear   431:------------------------------------------------------------ 431

c-dog    169:------------------------------------------------------------ 169
g-dog   1242:TCA--AATAGATTTTGTTTGCTGCTGTCTCCAATACCTATTTTTTAAAAAGATTTAT-TT 1298
g-panda 1246:TCACAAATACGTTTTGTTCACTGCTGTCTCCAACACCTGTTGTTGTTGTTTTTAAGACTG 1305
c-bear   431:------------------------------------------------------------ 431

c-dog    169:------------------------------------------------------------ 169
g-dog   1299:ATTTATTTGAGAGAGAGAGAGAGAGAGGGAGCACACTTGCAGATGCATGTGAGCAGTGGGGA 1358
g-panda 1306:ATTTATTTATTTATTTGAGAGAGAGAGAGAGCGCACACA--CGCACAGGAGCAGTGGGGA 1363
c-bear   431:------------------------------------------------------------ 431

c-dog    169:------------------------------------------------------------ 169
g-dog   1359:GGGACAGAGGGAGAGGGAGAGAATCCCAAGCAGACTCCCTCACTTAGTGGGGAGCCCGAT 1418
g-panda 1364:G------------AGGGAGAGAATCCCAAGCAGACTCCC-CACTGAGTGTGGAGCCTGAT 1410
c-bear   431:------------------------------------------------------------ 431

c-dog    169:------------------------------------------------------------ 169
g-dog   1419:GTAGAGCTTGATCCCATGAACCTGAGATCAGGACCCCAGCCAAAACCAAGAGTTGGACGC 1478
g-panda 1411:GCAAAGCTCAATCCCATGACCCTGAGATCATGACCCGAGCTGAAACCAAGAGTTGGA--- 1467
c-bear   431:------------------------------------------------------------ 431
```

\

```
c-dog     169:------------------------------------------------------------ 169
g-dog    1479:CTAACGGACTGAGCCACCCAGGTGCCCTGTGCCATCTCCCAACACCTAGAACATAAG-TG 1537
g-panda  1468:-TGGCTGAC--AGCCAGCCAGGCACCCCATGCTGTC-CCCAACACCTAGAACAGCGACTG 1523
c-bear    431:------------------------------------------------------------ 431

c-dog     169:------------------------------------------------------------ 169
g-dog    1538:GCACATAGCAACAAATGATGATGATGGTG------GTGATGG------TTAAATAGTGCT 1585
g-panda  1524:ACACATAGCAACAAAGGATGATGATGGTGTTGATGATGATAGCTACACTTAAATAGTGCT 1583
c-bear    431:------------------------------------------------------------ 431

c-dog     169:------------------------------------------------------------ 169
g-dog    1586:CCATCTGCCAGGCATTGGTCTAAATGGTTTAAGTATATGAGCTTGTTTACTCTTCACACT 1645
g-panda  1584:GCTTCCACCAGGTACTGGTCTAAATGCTTTAAGTATATTAGCTTATCTACTCTTCACGGT 1643
c-bear    431:------------------------------------------------------------ 431

c-dog     169:------------------------------------------------------------ 169
g-dog    1646:GACCAGGATCCCTGGGTGGCTCGGCAGTTTAGCGTCTGCCTTTGGCCCAGGGCGTGATCC 1705
g-panda  1644:GACC-------------------------------------------------------- 1647
c-bear    431:------------------------------------------------------------ 431

c-dog     169:------------------------------------------------------------ 169
g-dog    1706:TGGAGTCCCGGGATCGAGACCCATGTTGGGCTCCCAGCATGGAGCATGCTTCTCCCTCTG 1765
g-panda  1647:------------------------------------------------------------ 1647
c-bear    431:------------------------------------------------------------ 431

c-dog     169:------------------------------------------------------------ 169
g-dog    1766:CCTGTGTTTCTGCCTCTCTCTCTGTGTGTCTTTCATGAATAAATAAATAAAAATCTTAAA 1825
g-panda  1647:------------------------------------------------------------ 1647
c-bear    431:------------------------------------------------------------ 431

c-dog     169:------------------------------------------------------------ 169
g-dog    1826:AAAAAAAAACTCTTCACACTGACCCTATGAGGTAGAGAGGTCCACTGTATATGGATGTGA 1885
g-panda  1648:------------------------CTATGAGGTAGATAGGGCCACCGCGTATGGATGTGA 1683
c-bear    431:------------------------------------------------------------ 431

c-dog     169:------------------------------------------------------------ 169
g-dog    1886:AGAACATACACCACACAAGGATCCCACATCAACTAGGGCAGTATTCCCATCCAGGATATA 1945
g-panda  1684:AGAATGTACACTGCACAAGGATCCCACACCACCAAGGGCAGTATTCATACCCTGGATATA 1743
c-bear    431:------------------------------------------------------------ 431

c-dog     169:------------------------------------------------------------ 169
g-dog    1946:TTCATCAATTTCTGGTAGATAGCAGTAATGTATTTTGTTCTAACAAAATCAACACTTTAA 2005
g-panda  1744:TTTGTCAGTTTCTGGTAGATAGCAGTAACGTATCTTGTTCTAACAAGCACTTC-CAAGTA 1802
c-bear    431:------------------------------------------------------------ 431

c-dog     169:------------------------------------------------------------ 169
g-dog    2006:AGTAATTTAAGAAAGAAGTGTCATTTTCTAGGTATAGGTTTGCATTAGCCCTGAAGGTAG 2065
g-panda  1803:TTTTAAGAAAGAAAGAAGTGTCATTTTCTAGGTATAGGCTAGTATCAGCCCTGAAGGTAG 1862
c-bear    431:------------------------------------------------------------ 431

c-dog     169:------------------------------------------------------------ 169
g-dog    2066:GAATTATTCAGAACTCTGTC--ACAGATTAGAAAACTGAGGGCCAAAGAGGTGAAGGGGC 2123
g-panda  1863:AAATC-TTCAGAGTTCTGTTTTACAGATGAGGAAACTGAGGGCCAAAGAGGTGAAGGGGC 1921
c-bear    431:------------------------------------------------------------ 431

c-dog     169:------------------------------------------------------------ 169
g-dog    2124:TTATCCCAGGCCATAATTCAGAAACCCTGGGGTTGGGATTTTGGATCAGACCCTTGCCC 2183
g-panda  1922:TTATCTCAGGCCATGGTTCAGAAACCCTGGGGTTGGGGATTTGGATCAGATCCTTGCCC 1981
c-bear    431:------------------------------------------------------------ 431

c-dog     170:-----------------------------GTGTGTCCTGCTGAGACTCTGGGGTGCTTCC 200
g-dog    2184:AGAGATACCAAGTCACTCTGTCCCCACAGGTGTGTCCTGCTGAGACTCTGGGGTGCTTCC 2243
g-panda  1982:AGAGACACCAAGTCACTCTGTCCCCACAGGTGTGTCCTGCTGAGACTCTAGGGTGCTTCC 2041
c-bear    432:-----------------------------GTGTGTCCTGCTGAGACTCTAGGGTGCTTCC 462

c-dog     201:GGCTGGAGCTGTCTGTGATCCAGTTCGAAGAGGGCCCATCCTTGGGGATTGCGGTGTTCC 260
g-dog    2244:GGCTGGAGCTGTCTGTGATCCAGTTCGAAGAGGGCCCATCCTTGGGGATTGCGGTGTTCC 2303
g-panda  2042:GGCTGGAGCTGTCTGTGATCCAGTTCGAAGAGGGCCCATCCTTGGGGATTGCCGTGTTCC 2101
c-bear    463:GGCTGGAGCTGTCTGTG------------------------------------------- 479
```

```
c-dog     261:GGCTACAGCGTCTGCTGGATGCCCTGGGGTCTCGGCTGTGGGTGACTGGCCAGGGCCCTT 320
g-dog    2304:GGCTACAGCGTCTGCTGGATGCCCTGGGGTCTCGGCTGTGGGTGACTGGCCAGGGCCCTT 2363
g-panda  2102:GGCTACAGCGTCTGCTGGATGCACTGGGGTCCCGGCTGTGGGTGACTGGCCAGGGCCCTT 2161
c-bear    479:------------------------------------------------------------ 479

c-dog     321:GTCCACCCTGCGAAGGACATCCTCAGAGACCTGTCCCTCTCTTTCTGTCCAAACTCTTAG 380
g-dog    2364:GTCCACCCTGCGAAGGACATCCTCAGAGACCTGTCCCTCTCTTTCTGTCCAAACTCTTAG 2423
g-panda  2162:GTCCACCCTGCGAAGGACATCCTCAGAGACCTATCCCACTCTTTCTGGCCAAACTCTTGG 2221
c-bear    479:------------------------------------------------------------ 479

c-dog     381:AGTTATTACAGGGGGCTTGTGCTCAGCACCTGCCCTCAGCATGAGCCTGGGAAGACACAG 440
g-dog    2424:AGTTATTACAGGGGGCTTGTGCTCAGCACCTGCCCTCAGCATGAGCCTGGGAAGACACAG 2483
g-panda  2222:AGTTGTTACAGGGGGCTTGTGCTCGGCACCGGCCCTCAGCATGAGCCTGAGAGGACACGG 2281
c-bear    479:------------------------------------------------------------ 479

c-dog     441:ACTCC---------                                               445
g-dog    2484:ACTCCCTGGAACTG                                               2497
g-panda  2282:ACTCCCT-------                                               2288
c-bear    479:--------------                                               479
```

Fig. S4*B* Translated *Ursus americanus* (American black bear) *IL-15Lψ* cDNA (EST GW294330)

```
         10        20        30        40        50        60
ggggagaggtggagggagggagcacccagaggctgacctgggggacacacagacagata
 G  E  R  W  R  E  G  A  P  R  G  *  P  G  G  T  H  R  Q  I
  G  R  G  G  G  R  E  H  P  E  A  D  L  G  G  H  T  D  R  Y
   G  E  V  E  G  G  S  T  Q  R  L  T  W  G  D  T  Q  T  D

                  out of frame ATG          in frame ATG
         70        80        90       100       110       120
cacagcacaagagagacagacacacatgaaggagaaagacaaagggccaatggagaaga
 H  S  T  R  E  T  D  T  H  E  G  E  R  Q  R  G  Q  W  R  R
  T  A  Q  E  R  Q  T  H  M  K  E  K  D  K  G  A  N  G  E  E
T  Q  H  K  R  D  R  H  T  *  R  R  K  T  K  G  P  M  E  K

                            out of frame ATG
        130       140       150       160       170       180
ggagaaagaagataagacacaggggcaattacagatggacccagaaaacacaagtgacgg
 G  E  R  R  *  D  T  G  A  I  T  D  G  P  R  K  H  K  *  R
  E  K  E  D  K  T  Q  G  Q  L  Q  M  D  P  E  N  T  S  D  G
R  R  K  K  I  R  H  R  G  N  Y  R  W  T  Q  K  T  Q  V  T

     if the ATG at position 111 is used as start codon, the
     underlined stretch with many charged amino acids would be
     positioned N-terminal of the sequence which forms the
     leader peptide in canonical IL-15L molecules


        190       200       210       220       230       240
cgattcggagacagagaggccagccctcagagaaagaagacagacaggacgctgggctgg
 R  F  G  D  R  E  A  S  P  Q  R  K  K  T  D  R  T  L  G  W
  D  S  E  T  E  R  P  A  L  R  E  R  R  Q  T  G  R  W  A  G
A  I  R  R  Q  R  G  Q  P  S  E  K  E  D  R  Q  D  A  G  L
```

```
                       site of consensus IL-15L start codon
       250        260        270        280        290        300
gaccggagaaagccttgggagtgggcagggtgcccgtgtggcctctctggaccatcctcc
 D  R  R  K  P  W  E  W  A  G  C  P  C  G  L  S  G  P  S  S
  T  G  E  S  L  G  S  G  Q  G  A  R  V  A  S  L  D  H  P  P
G  P  E  K  A  L  G  V  G  R  V  P  V  W  P  L  W  T  I  L

       310        320        330        340        350        360
tgctggtacggcccttgggaggcctaggatcacccctctgtcctcgggagcctttctact
 C  W  Y  G  P  W  E  A  *  D  H  P  S  V  L  G  S  L  S  T
  A  G  T  A  L  G  R  P  R  I  T  P  L  S  S  G  A  F  L  L
L  L  V  R  P  L  G  G  L  G  S  P  L  C  P  R  E  P  F  Y  F

  translation from these ATGs would produce
  IL-15L without leader peptide and α-helix A
       370        380        390        400        410        420
tccttgttgccatcatgaagatgctgggaaacaaaaatgatggcactctctacaccccag
 S  L  L  P  S  *  R  C  W  E  T  K  M  M  A  L  S  T  P  Q
  P  C  C  H  H  E  D  A  G  K  Q  K  *  W  H  S  L  H  P  R
   L  V  A  I  M  K  M  L  G  N  K  N  D  G  T  L  Y  T  P

       430        440        450        460        470        480
atgatctctcggtgtgtcctgctgagactctagggtgcttccggctggagctgtctgtg
 M  I  S  R  C  V  L  L  R  L  *  G  A  S  G  W  S  C  L
  *  S  L  G  V  S  C  *  D  S  R  V  L  P  A  G  A  V  C
D  D  L  S  V  C  P  A  E  T  L  G  C  F  R  L  E  L  S  V
```

Fig. S4*C* Translation of predicted "full-length" *Canis familiaris* (dog) *IL-15Lψ* cDNA

```
  1   GAGGAGAGGTGGAGGGAGGGAGCACCCAGACGCTGACTTGGGGGGACAGACAGACATACA

                 upstream in frame ATG    upstream out of frame ATG
 61   AAGCACAAGAGAGACAGACACACATGAAGGAGAAAGACAAAGAAGGGGCCAATGGAGAAG
                              M  K  E  K  D  K  E  G  A  N  G  E

                              upstream out of frame ATG
121   AGGAAAAAGAAGATAGACACAGGGTCAATTACAGATGGACCCAGAAAACACAAGTGACGG
      E  E  K  E  D  R  H  R  V  N  Y  R  W  T  Q  K  T  Q  V  T

181   CGATTCGGAGGCAGAGAGGCCAGCCCTCAGAGAACGAAGACAGACAGGACCCTGGGCTGG
      A  I  R  R  Q  R  G  Q  P  S  E  N  E  D  R  Q  D  P  G  L

                             site of consensus IL-15L start codon

241    GACCAGAGAAAGCCTGGGTGGTGGGCAGGGTGCCCGTGTGGCCTCTCTGGACCATCCTCC
      G  P  E  K  A  W  V  V  G  R  V  P  V  W  P  L  W  T  I  L

301   TGCTGGTACGGCCCTTGGGAAGCCTAGGATCACCCCTCTGTCCTCGGGAGCCTTTCTACT
      L  L  V  R  P  L  G  S  L  G  S  P  L  C  P  R  E  P  F  Y
```

translation from these ATGs would produce
IL-15L without leader peptide and α-helix A

```
361  TCTTAATTGCCATCATGAAGATGCTGGGAAACAAAAATGATGGCACTCTTTACACCCCAG
     F  L  I  A  I  M  K  M  L  G  N  K  N  D  G  T  L  Y  T  P

421  ATGACCTTTCGGTGTGTCCTGCTGAGACTCTGGGGTGCTTCCGGCTGGAGCTGTCTGTGA
     D  D  L  S  V  C  P  A  E  T  L  G  C  F  R  L  E  L  S  V

481  TCCAGTTCGAAGAGGGCCCATCCTTGGGGATTGCGGTGTTCCGGCTACAGCGTCTGCTGG
     I  Q  F  E  E  G  P  S  L  G  I  A  V  F  R  L  Q  R  L  L

541  ATGCCCTGGGGTCTCGGCTGTGGGTGACTGGCCAGGGCCCTTGTCCACCCTGCGAAGGAC
     D  A  L  G  S  R  L  W  V  T  G  Q  G  P  C  P  P  C  E  G

601  ATCCTCAGAGACCTGTCCCTCTCTTTCTGTCCAAACTCTTAGAGTTATTACAGGGGGCTT
     H  P  Q  R  P  V  P  L  F  L  S  K  L  L  E  L  L  Q  G  A

661  GTGCTCAGCACCTGCCCTCAGCATGAGCCTGGGAAGACACAGACTCC  707
     C  A  Q  H  L  P  S  A  *
```

Fig. S4*D* *Homo sapiens* (human) hybrid *IL-15Lψ-SUPT5H* cDNA sequence (EST DC400386) indicated in green font within the genomic region *RPS16*-to-*SUPT5H*

```
   1  GGGCGTGGTGGTGCATGCCTGTAATCTCAGCTACTCAAGAGGCTGAGGCAGGAGAATCGC
  61  TTTAACCCGGGAGGCGAAGGTTGCAGTGATCCGAGATCGCGCCACTACACTCCAGCCTGG
 121  GCGACACAGCGAGACTCCCTCTCAAAACAAATAAATAAAATAAAAAATAGTCGGTGTCCC
 181  TTCACCCTTTGGGTCCCCCCGCCCCAGTGAGGTGAGCTTCCTAACATCCCAGTTCTCAAA
 241  GCAAACCCTTAATCTTCAACAACCCCATCTCAGCTTTTACATCTTCTGAACCCCAAGCCC
 301  AGCTCTATTGCCAAATACCCCACTGTTCTACACCCAACACAACTTCTAAACATCCCGTTG
 361  CTGATGCCAGCCCCCCCAGCTTTCAAGAGCTACAACATCTCTGTCTCCAAGAGTCCTCCA
 421  TCCACTCAACGCCGGTGCCGGATCCCAGCACCTTGTACTGTAGCGTGCGCGGCTCAATCA
 481  TCTCCAGGGGCCGCCCGTTCACCTTGATGAGACCATTGCCGCGTTTGCAGTGCGCCACAG
 541  CTGTCGCTGTCTTCTGTAAGATACAAGAGAAACAGGGGCCCCGTGAGCTCCGGCTCCAGC
 601  TCCCATGTTACCCCCTAGATTCCTGCCCACCGACCTCTCCCAGACCCTGGCCTTCTCCTT
 661  CCCCTCCCCTTCCCATCCGGCGTCTGGCTCACCTTGCGTCCGAAGACCTGCACAGACTGC
 721  AGCGGGCCCTTGGACGGCATGGCTCCGAGCGTGGACTAGACAACCTCACCGCGCGGCGCC
 781  GCAACCGGAAAAGGAAAGCTAGGGGCCACCCTGGCCGCTTTTCAGGGTCTGCGCAGGCGC
 841  CTTGAGCACTGCGTCGCGACGGGAGAGAGCTTTACGGCTTCTGTTGCGGCTAACGGGCGG
 901  GGCGTCTGGTCAGCGGCGGGAAGGCCCTTAGGCGCTCAGAGCGGGCGGGCCCTGTGAGGC
 961  GGAAGTGGCGGGCTCTGTGGGCGGAAGGGGCGGGGCCTGGGAGTGTCGTGTGCGGACTGA
1021  AGGGAAGGCTGCGGGAGTGTGCTTCGCGTCCTCTGTAGTCGACTACCGCAGCTCTTCTTT
1081  GCATTCTGGCTCTTTAATAATCTCCCTTCGCCCTTTCTTTAAATCAGTTCCCACCCTCCC
1141  AACACCTGTAATCTTTCTCCCCTCTGGACTCCCTCCTCCCCGAATCCCCCGCATCAGAAT
1201  CTTCCTTTGGAATCTACTGCAAGAAATCTGGCTGGATTCTCAGATTAAGGACTTCCAGAA
1261  TCCTATCCTCCCTAGAATCCCCCTCCTTGGGGATCCTCTCCCAGAATCCTTTTCCCCTCA
1321  GAGCCCCTTCCCCTCAGAATCTCCCTTTCACCAGAATGTCCTTCCTTTCAGTTTCCTCTT
1381  CCCAGAATGTTTCCCATCCTTCTCTCAGATGGCCAACCCCAAATGTCCCTCTTAAGAGTG
1441  CTCAACTTAGACCCCCCTTTTCCCTGAATCTCCCTCCATTCGGTCCTCGTCCCCTCAGTC
1501  ACCCTTCCCAGAATCTTTCTCTTGTCAGGACCCAGTACCCCTGGAATCTCCCTCCTCCAG
1561  AATCTCCCTTTCCCGGCAAATCCCCCTTTCCCGGCAAATCCCCCTTTCCCAGAACACCCC
1621  GCCCCGCAAATCCACAAAAACTTTTCTCTCAGAGACCCGTCCCTCAAAATCTACCTTTCT
1681  CCTGAGTTTATTTCCCTTCCGAGACTCCTCTTCCCAGCAACTGCCTTCCCTGGAGTTTTC
1741  CCTCCGTTAGAATCCCCCTCTCCTAAGAGACCCTCCCCCAAGAATTAACCTCTTCCCCGT
1801  CCCCACCCTAGAATCTCTCTCTTTTTTTTTGAGACGGAGTTTCGCTCGTTGCCCTGGCTAG
1861  AGTGCAGTGGTGCGATCTCGTCTCACTGCAACCTCCGCCTCCCGGGTTCAAGAGATTCTC
1921  CTGCCTTAGGCTCCCGAGTAGCTGGAATTACAGGCGTCTACCACCACGCCCTGGTCGTTT
1981  TTTGTATTTTTAGTAGAAATGGGGTTTCACCACGTTGGCCAGGCTGGTCTCGAACTCCTG
2041  ACCTCAGGTGATGTGATCCGCCTCAGCCTCCCAGAGTCCTGGGATTACAGGCGTGAGCCA
2101  CCACGCCCGGCCGAATCTCTCTGTTTTTTGTTTTTTTTTTTTCTTTTTTTTGAGACAGAG
2161  TCCTGCTCTGTCGCCCAGGCTGGAGTGCAGTGGCATGATCTCTGCTCACTGCAACCTCTG
2221  CCTCCCCGGTTAAAGCGATTCTCCTGCCTCAGCCTCTTGAGTAGCTGAGATTACAGGCAT
2281  GCACCACCACGCCCGGCTAATTTTTGTATTTGTAGTAGAGACGGGGTTTCACCATATTGG
2341  CCAGACTGGTCTCGAACTCCTGACATCAAATGATCCACTCATCTCAGCCTCCCAAAGTGC
2401  TGGGATTACAGGCGTGAGCCACCGCGCCCAGCCCCTAGAATCTCTTTCTCCAGAATCTCC
2461  CAGTCATCTTTTTCCTCTCCCCCAAATCACCCTCCACTTAAGCCTCCTCCCCACAGAATT
2521  CTCCATCCCCAGCCCCCCAACCCCACCTGAGGAACCCCAGGATTGGGTTGTCCTCTGGTT
2581  CCCCGGCAGATGTCCCTGCGTCTCACCCCATCAAGAGTCTCCCCAGCGAAGAGGTTCCTC
2641  TCTCAGAGCTAGAGGGAGCCCACAGTTGCCTCAGTTTCTCTGGCAGTACAGGGTCTGGGA
2701  TCCAGGGTTCAAAAGGGCGTCGTGGTGCACCTTGCCAAAGAGGGTGGAGACAGGGAGACA
2761  CTGGAGGGCAGGTCTGGGGCCAGAGGGAAAAGCCAGCCCGGAGTCCAGCTCTAAAAGGGT
2821  GAGGGGGGAGGGGGCTGTTGGGAGGGAGGAAGGAAGTACCCAGAGGCTGACCGGGAGGGA
2881  CACAGACACAGCCCAAAGAAATGTAGGCACAAGTGGAGAAGAAGAGAAAGATGAAAGGCC
2941  AAGGGAGAAGAGGAGGAGAAAAGGGAGAGACCAGGACAGTCAAGAGATGGACCCCCAAAA
3001  CAAGCCCAGTGACAGATTTAGAGGCAGAGAGGCCAACCCTCAGACAAAAAAGAGATAAAG
3061  GCAGGCATGATGTTTCCAAAATACTTGAGACAGAGGCCAGGTGCAGTGGCTCACACCTAT
3121  AATCCCAGCACTTTGGGAGGCCAAGGCAGCAGGATCACTCGAGCCCAGGGTTCAAGACCA
3181  GCCTGGCCAACATGGTGAAACCCCGCCTTTACTAAAAATACAAAAATCAGCTGGGCATGG
3241  TGGCAGGTGCCTGTATCCCAGCCACAAGGGAGGCTGAGGCAGGAGAACCGCTTGAACCCG
3301  GGAGGCGGAGGTTGCAGTGAGCTGAGATGTCGCCACTGCACCCTAGGCTGGGCGCCAGAG
3361  CAAGACTCTGTCTCAAAAATAAATAAATAAATAAACTCTGTCTCAAAAATAAATAAATAA
3421  AATAAAATAAATAGAGCAGAGACCTCAAATATTCACTTGCAGACACAGATAAAGGCTAGC
3481  ACAGGTGTTTTCCAATGAAGACACAGGCAGATGAGAGGATGCACACATTCTACCTGTACT
3541  TTTTCTTTTTTTTTTTTTTAAATTGAGATAGAGTCTGTGTCACCCAGGCTGGAATGCAGTG
3601  ATGCAATCTGAGCTCACTGCAACCTCCACCTCCCCGGTTCAAGTGATTGTCCTGCTTCAG
3661  ACTCTGGAGTAGCTGAGATTACAGATTCTCCTGCTTCAGACTCCTGAGTAGCCCAGATCA
3721  CGGGCCTCCACCACTATGCTTGACTAATAATTTTTCTTTTTTTTTTTTGAGATGGAGTAT
3781  CGCTCTGTCGCCCAGACTGGAGGGCAGTGGCTTGATCTCAGCTCACTGCAACGTCAGCTT
3841  CCTGGGTTCAAGCAATTCTCCTGCATCAGCCTTCCAAGTAGCTGGGATTAAAGGCATGCA
3901  CCACCATGCCCAGCTAATTTTTGTATTTATTAGTAGAGACAGGGTTTCACTATGCTGGGC
```

Exons 1 and 2 of *RPS16*, complementary strand; coding regions are shaded pink and the 5'UTR is underlined

Start of EST DC400386, forward strand. Non-canonical splicing motif.

```
3961  AGACTAGTCTTGAACTCCTGACCTCAAGTGATCCACTTGCCTCGGCCTCCCAAAGTGCTG
4021  GGATTACAAGTGTGAGCCACTGTGCCTGGCCACTAATTTTTGTATTTTTTAGTAGAGACA
4081  GGGTTCCATCGTGTTGGCCAGGCTGGTCTTGAACTCCTTACCTCAAGTGATCCGCCCACC
4141  TCGGCCTCCCAAGGTTCTGGGATTACAGGTGTGAGCCACAGCGGTTGGCCTAGCCTGCAC
4201  TGTCTTAAGGGCCTGTTGGGCAGGGGAGTTCAGGTTCTCTCGTCTCTCCAGATTGATCTT
4261  CACACATTTGCTAAGTCTCGTATCGTCCCTGAGCTTGGGAGTCAGGGAGCGCTCTGACCT
4321  AAGCCCTCCTGACTTTGGGGTCACCATCTTTCCCCAGCCTCTATGGGCACACTCCCTGAA
4381  TTATCTGTGGCTTGAGTGGAGCAAGCAGGGGATGGGATGAGGGGTGGCGGGGGAGTTCCC
4441  CTCATCCACTTCAGCCTTCCAGTCTGCTCCCCCTTCCTGTACCTCTCTCTTCCAGAACCC
4501  CAGGCCAAGCACCAGAGAAGGCCTGGGAGTGAGCAAGGTGCCCATGTTGCTTCTGTGGAT
4561  CATCCTCCTGCTTGTACAGCCCTGGGAGGGCCTAGGAACGCCCGCCCCTCTGCTGGAGGG
4621  AGCCTTTTTACTTCCTTGCAGCCCTCTGAAGATACCAGCGAGTGGGGAGCAGAGCCAGCA
4681  GACGGGGTGGGCCAATTGATGGGGCGTGGTAGGGGGTGTGTTTGAGGAAGGGTCAGCCTC
4741  TTTCCCTCCAAGATCTGATTCTCCTCCTTACCCCTGAACTGTAGGAAAACAAAAATGATG
4801  ACACCCTCTACACCCCAGATGATCTCTCAGTGAGTATTGCATTCGCTTCTTTCTGCAGTT
4861  GCAGGTTTTGAGAGAATTCAGTGGTCTCCATGTGTGAGGGCTGCTGTGTGACCATATGAA
4921  TATGTTGCGGGGAGGAAGTAGTGGGACTATCTCCTGACCCAGCCCCTAGCCTTGACAGAT
4981  ATATTTTGCTCACTGCTGTCTCCAACACCTAGAATAGGGAGGTATATAATAACAAATGAT
5041  GATGATGACTACAGCTGCATGTAAATAGTGTTTACTCTGTGCCAGGTATTGGTTTAAATG
5101  CTTTAAGTATGTTAGCTTATTTACTCTGTACACCAATCCTATAAGATAGATAGGACCACC
5161  ATGTATGGATGTGAATGTTGTACACTGCACAAGGATCCCACATCTACAAAGACAGTACTA
5221  TATTCACAGGCTGGATATTGTAGATTTGCATATTTGCCAACTTGCGGCAGATGGCAGTAA
5281  AGTATCTTGTTCTAATAAAATCAAGACTTAGAAGGTAGAAGGAACTTAAGGAAGGTCTTT
5341  TTATTTTCTTTTTTTTGAGACAGGGTCTTGCTCTGTCCCCTACGCTGGAGTGCAGTGGCA
5401  CAATCACAGCTCACTGCAGTCTTGACCTTTTAGGCTCAAGCAATCCTCCCACCTCAGCCT
5461  CCAGAGTAGCTGGGACCACATACACGTGACACCACAAATAGCTAATGGCTAATTTTTTTT
5521  TTTTTTTTTGAGACACAGTGTTGCTCTGTTGCCAGCTGGAGTGCAGTGGCGTGATCTCT
5581  GCTCACTGCAACCTCTGCGTCCTGGGTTGAAGCAATTCTTCTGCCTCAGCCTCCCGAGTA
5641  GCTGGGACTACAGGCATGTGCCACCATGCCCAGCTAATTTTTGTATTTTTAGTAGAGACA
5701  GGGTTTCACCATGTTGGCCAGGATGTTCTCGATCTCTTGACCTCGTGATCCACCTGCCTC
5761  AACCTCCCAAAGTGCTGGGATTACAGGCGTGAGCCACCGTGCCCGACCTTTTTTTTTTTT
5821  TTTTTTTTTTTGTAGATATGGGGTGTCGCTGTGTTGCCCAGGCTGGTTTTGGACACCTGG
5881  GCTCAAGTGATCTTCCTGCCTTGGCCTCTCAAAGAGCTGGGATTACAGGCATGAGCCACT
5941  GTGCCCAGCTGAGTGTCTTTTTCTAGCTTGCACATAAGTACAGCATGGGATAGTGTCAGG
6001  CCTTACGGTTGGGATTATCCAGAGCCCTATTTACAGATGAGGAAACTGAGGCCCAGAGAA
6061  CTGAAGGGGTTTGTTCCAGGACACAGATAAGAAATCTTGGGAGTGGGGACTTGGACTCAT
6121  GCCCTTACCCATAGATACCAGGTCACTTTGTCCCTACTGTGTGTCCTGCTGAGACTCTAG
6181  GCTGCTCCAGGTTGGAGCTGTCTGTGATTGGGTTTGAGGAGGGACCATCTATGGGAACTG
6241  CTGTGTTCCAGCTACAGTACCTGCTGGATCTCCTGGGGTCCCGGCTGTGGGTGGCCAGCC
6301  AGGGCCCTTGCCTGTCCTGTGAAGGACATCCCCAGAGACCTGTCCCCCTCTTGGAGTTAC
6361  TACAGGGAGCTTGTGCTTGGCACCCTACCACAGCCTGAGCTTTGGAGGACATGGGCTCCC
6421  AGGTACACCCAGAGAGGCTGGGTAGAGAGAGGGAGGGAATAGTCTTCAGCTCCAACTGCC
6481  AAACCCTAAGCCTTTTCTTCCCTCTAGGCCAGAGGACCCCACCTGGAGCCCTAGTAGCCA
6541  AGGGGAAGAGTTAAGAAGGAGGAAAGTAAGGTGCGATCTGAAGTCTCTGACCTCTTGGGC
6601  AGGGAGTTGGATAAATTGCCCCACCTGCCCCCATTCTCACCCTCCTTTAGATACTCTTCT
6661  CTCTTCTTCCAGGAAGGGACAGAGAAGTGCACAGGTATTCTCTAACCTCATCCCTATACC
6721  TTGAGAACAGGGAGGGTCCTGTGGTTCACATCCCTACATCCCTGGCTACAAAAAATTACC
6781  ACTTGAAGAACTTTCTTTTCTTTTCTTTTCTGTTTTTGAGGCAGAGTCTCACTCTGTTGC
6841  CCAGGCTAGAGTGCAGTGGCATGGTCTCAGCTCGCTGCAACCTCCACCTCCTGGGTTCAA
6901  GTAATTCTCCTGCCTCAGCCTCCTGAGTAACTGGGATTACAGGCACGCGCCACTACACCT
6961  GACTAATTTTTGTATTTTTAGTAGAGATGGGGTTTCACAATGTTGGTCACGCTGGTCTCG
7021  AACGCCTGACCTTGTGATCTGCCCACCTCAGCCTCCCAAACTGCTGGGATTACAGGCGTG
7081  AGCCACCGTGCCCAGCCCACCACTTGGAGAACTTTCAAGTAATACTGATGGCCAGACCCC
7141  AGCCCAGATAATTTGATAATTGGTCTCTGGTGAAGCCTGAGCATCTATATTATTTTTATT
7201  ATTATTTAGAGACAGGGTTTCACTATGCTGCACAGGCTGTAACTTGGCATCTGCCCATAT
7261  ATTTTTTTGAGACAGGGTCTCACTCTGTCACCCAGGCTGGAGTGCAGTGGCATGATCAAA
7321  ACTCATTGCAGGCTCGATCTTTCAGGCTCAAGTGACTCTCCCACCTCAGTCCCCCAAGTA
7381  GCTAGCGCTATAGGCGCATGCCACCACACTCAGCTAATTTTTGTATTTTTTGTAGAGATG
7441  GGGTTACCCTTTATTGCCCAGGCTAATCTTGAACTCCTGGCTCAAGCGATACTCCCCCCT
7501  TGGCCTCCCAAAGTGCTGGGATTATAGGCATGAGCCACCACACCTGACATTTTTTTTTTT
7561  TTTTTGAGACAGAGTCTCCCTTTGTCACCCAGGCTGGAGTGCACCGGCTGGTGGACTGGA
7621  TGGCCACATTTCCATCACCCCAAAGCATTCCTTCTCTCTCTTCCCAGTCAATTTCCACCT
7681  CCCTCTCCAGCCCCAGACATCCACTAATCTGCATTGTCTGTATAGGTTGGTTTTGCTTGC
7741  TCTGGAGTTTCATATGAATGGAATCATGCTGGACATCTCCTGATGTCTGGCTTCTTTCAC
7801  TCAGCATAATGTTTTGGAGATTCAGTCATGGGTGTATTGGTAGTTGTTACTATTCTGTTG
7861  CATGGATGGATGTACCAAAATTTGTTTTTCTTTTGAGACGAGTCTCACTCTGTTGCCCAG
7921  GCTGGAGTGCAGTGGTGCGATCTCTGCTCACTGCAACCTTCACCTCCCTGGTTCAAGTGA
7981  TTCTCCTGCCTCAGCCTCCCAAGTAGCTGGGATTACAGGCGTGCGCCACCACACCTGGCT
8041  ATTTTTTTTTTTTTTTTTTTTGTATTTTTAGTAGAGATGGGGTTTCACCATATTGGCCAG
8101  GCTGGTCTTGAACTCCTGACTTCGTGATCCGCCTGCCTTGGCCTTCCAAAGTGCTGGGAT
8161  TACAGGCGTGAGTCACTGCGCCCGGCCCTAATTTTTGTGTTTCCAGTTTAGGGCTATTAT
8221  GCAACTCTTTCTTTCTAAAATATTCTCAAGTATTCTGATGTGCTACCAGAGTTGAGTTCA
```

EST DC400386 partly overlaps with canonical *IL-15L* exon1 region (gray shading), and exactly matches canonical *IL-15L* exon2.

Arrows underline sequences of forward primers that we successfully used in separate RT-PCR experiments, in conjunction with a reverse primer binding to the *SUPT5H* gene (see below), to confirm the existence of *IL-15L ψ-SUPT5H* hybrid transcripts

Canonical *IL-15L* exon3/4 region (gray shading), which is incapacitated for protein coding ability (Fig. S1) and was not found in any transcript.

The exon depicted with blue font was found in some but not all of the *IL-15L ψ-SUPT5H* hybrid sequences that we amplified

```
8281   GAACCACGGATCCACCAAGGAAGAAACTTCCATGGCTGGGGAAATGACTGTGCCCTCTTT
8341   TTTCTGCAGGGAATGGCTTTCTGCAGCAGTGTCTGTAGCGATTTAATATGTAGAATCCTA
8401   TTTTCTTGTTGTTTTTTTTTTTTGAGACGGAGTCTTGCTCTTTTGTCACAGCAACCTCCAC
8461   CTCTCAAGTTCAAGAGATTCTCCTGCCACAGCCTCCCGAGTAGCTGGGATTACGGGTGTG
8521   CGCCACTACGCCACCTGGCTAATTTTTGTATTTTTTAGTAGAGACAGGGTTTTACCATGT
8581   TGGCCAGGCTGGTCTTGAACTCCTGACCTCAGGTGATCCGCCTGCCTTGGCCTCCCAAAG
8641   TGCTGGGATTACAGGCGTGAGCCACCGCGCCCGGTGGAGAATCCTTTCTTTTTTTGAGAT
8701   GGAGTCTAGCTCTGTTGCCCAGGCTGGAGTGCAGTAGCACGATCTCGGCTCACTGCAAGC
8761   TCGGCCTCCCGGGTTCACGCCATTCTCCTGCCTCAGCCTTACGTGTAGCTGGGACTACAG
8821   GCGCCCGCCACCACGCCCAGCTAATTTTTTTTTGTATTTTTAGTAGAGATGGGGTTTCAC
8881   CGTGTTAGCCAGGATGGTCTCGGTCTCCTGACCTCGTGATCCACCCGCCTCGGCCTCCCA
8941   AAGTGCTGGGATTACAGACGTGAGCCACCGCGCCCGGCCCAGGTGTGTCATACTTATGTG
9001   AGAAATTTTACCAATGTGTCCTGAGGCTTCTTTTTTTTTTTTTTTTTTTGAGACTGAGT
9061   CTCACTCTCGCCCAGGCTGGAGTGCAGTGGCGCGATCTCGGCTCACTGCAAGCTCCGCCT
9121   CCTGGGTTCACGCCATTCTCCTGCCTCAGCCTCCCGAGTAGCTAGAATCACAGGCACATG
9181   CCACCACGCCCGGCTAATTTTTTTGTATTTTTAGTAGAGACGGGGTTTCACCATGTTGGC
9241   CAGACTGGTTTCGAACTCCTGACCTCAGGCAATCTGCCCGCCCCGGCCTCCCAATGTGTT
9301   GGGATTACAGGCATGAGCCACCGCGACTGACATAGACTTCTGATTTTTAAAATTTCCCCT
9361   ATCAGGTTAATTTTTCAATTTTTAAAAATTTAAGTTTTAATTTTTTTTTTTTTTTTTTT
9421   TAGAGACAGGGTCTCTATTACCCAGTCTGGAGTGCAGTGGTGCAATCATAGCTCACTGAA
9481   GCCTCAACATGGGTTCAAGTGATCCTCCCACCTTAGCCTTTGGAGTAGCAGGGACCACAG
9541   GCACACGCCACCACACCACATTAAAAAAAACACAAAAATTGGATACATGGGGTCTCAATT
9601   TGTTGCCCATGCTAGTCTCAAATACCTGGCCTCAGGTGATCCTCCCGCCTCAGCCTCCCA
9661   AAGCGCTGGGATTACAGGCGAAGGTTCATTTTTCATCTCTCCATTACACCTGTCCACTCT
9721   GCCATCACTCGTCAGCCGTCATTACTAATAGATAAATTCGCCTTTAAATGAACTTAGGAA
9781   GCCTGGAAAGAAAAATAGGGACTTGGGCCGGGCGCGGCAGGTTATGCCTGTGATCTCAGC
9841   ACTTTGGCAGGACGAGGCCGGAGGATGGCTTTTTAGTTTGAGTTAGAGGAGTTTGAGACC
9901   AGGCCGGGCAACAGCCCGACTCTACCAAAAAAAAAAAAAAAAGTACAAAAAGAAAGAAAAA
9961   GAAGGAAAAACCAGGGCTTCACGGTCACTCAGACTTGGCGAGGAGGCTTGGGTCTGCCGC
10021  TTTCCACTGAGTTTTTCTGAGCTTCAGCGTTCTCTCCAAAACGGGGATACGGATCTCCTG
10081  GGGATGCTTTAGAGGGCCTAGGAGATAAAGCCTTGTTTCTTTTATAATTTGCTATTCGCT
10141  GATTTCGCCGCCTCCTCGCAGCTTGGCCTACCGCGCTAGTGTTCCGGACTAACGAACAAA
10201  CGGCTCACTAAATTCAAACATGAGTTTCAGCCTGGTCGCCGAGCCGGCGGGTAGAAAAAA
10261  CCTTTCCCCTCCTAACCCTCCGCAGATGTCGGCCCCGCACTCCGCCCAGGCCAGCCCTGC
10321  CTCTCTCAAGATGGCGACCTGGAAGTGCGTCGCCCGTGCCAGCCCCGGAAATCTCGTGTT
10381  AGGCGAGGTCAACGGGTAGGTTCTCGCGAGAGGACCCGTCAGCCCCAGTCAGGCGTCGTG
10441  CGAACAGCAGCTGGTACCGAAGGCGGAGGTGGAGCCCGAGAGGTAAGTGCGTGTGCAGAG
10501  GTGGCAGTTCCGGGCGCCGGGGAGGTGTAGAGAACAGATTCGGAAACTGGGGAGGTCTAG
10561  CATGTGGCGTAGGAGGGGGTCCTCACTCCGCTTCGCGATTGCCAAAACGAGCCTGCCGGA
10621  AGCGCCCTAAGGGGTTTTCTTCTCCCAGGGAACCAGCGGGGAAACTGAGGCTCGGGGTGG
10681  AGCGCAGGATTGTGGGACGCGCCAAGGCTGCTGTCTTTCCCAGCAGCAGCGGAAGATGTC
10741  GGACAGCGAGGACAGCAACTTTTCCGAGGAGGAGGACAGCGAGCGCAGCAGTGACGGCGA
10801  GGAGGCCGAGGTCTGTGGCTGGGGCGCTGGGGGAGACATTGCGTCTGGGGACAGGACTCC
10861  GGGCAGAAAGGCCCCTGTGGGAGGCTCGAGGGGTTCACAGCGGGCTCTGGCTTCTGGGAA
10921  CTCCCAGATTGTCTCAAGAGATAGAGAATTAGGCAAGTCGAAGTCAAGCAAAGGGACAGG
10981  TTCTGGAGGCCAGAAATGGGTCAGGTCCAGTGAGATAACAGGTGATGTTTTGAAAGTTTA
11041  CTCTGTGTGCATATAGTAAGCACTTAGCATTAGTTTTTTTTTTTAGTTATGTCTAGTCAT
11101  TCCATAACTATATTTGTAGTGCCTACTATGTACCAGGCGCAATTCTTTGCTCTTAGGGTA
11161  CACCAGTAAACAAAAAGATTTTAAAAATGTCTGCCTTCGGGAATGTAAATAAATATAGCC
11221  AAGGAATAAACTATATAGTAAGCTGGAAGGTACTCTGGAGAAAAGAACAGAAAAGGGGAA
11281  TGGAGAGTCCTGGTTGCTCTTGAAATAGGGTCATCAGCATGAGTCTGAGGGGTGACATTT
11341  GAGCAAAAACTTGTTGGAGGTGAGGGAGTGAGTCGTGGTTAGCTGGAAGTGTAGTGTTCC
11401  AGTGAGGGGGACCAGCAGGTGCAGAGATGGGAGTGTGTCCGCCGTGTTCAGGGATTAACG
11461  AGGATTTGAGAGGCCAAGCTGGGAGGATCACTTGAGCCCAGAAGTTTGAGAGCAATCTCG
11521  GTAACACAACGGGACCCTGTCTCTACAAAAATATAAAAACCATTGGCTGAGGCCGGGCTT
11581  GGTGGCTCACACCTGTAATCTCAGCACTGTGGGAGGCCAAGACAGTGGATCACCTGAGGT
11641  CAGGAGTTCAAGACCAGCCTGGTCAACATGGTGAAACACCATTTCTACTAAAAATACAAA
11701  AAAATTAGCTTGGCATGGTGGCGCCCACCTGTAATCCCAGCTACTCGGGAGGCTGAAGCT
11761  GAGAATCGCTTGAACCAGGGAGGTGGAGACTGTGGTGAGCTGAGATCGCGCCACTGCACT
11821  GCAGCCTGGGCGACGAGCAAGACTCCGTCTTATAAAAATATTAGCTGAGCTTGGTGGCAC
11881  GCTTCTGTAGTCCCAGCTACTTGGGAGGCTGAGGTGGGAAGATTGCTTGAGCCAGAAGGT
11941  GAAGGTCTAGTGAGCTGTGATGGTGCCACTGCACTGCAGCCTGGGTGACAGAGTGAGACT
12001  TTGTCTCCCCACCACCCCCTAAAAAAAGGGGGTCCACTTTGGTTGGATCAGAGTGAGTGC
12061  AACAAAGAATTTATGAGAGCCAAGTCATATAGGATCTTGAAGGCTGTGGTAAGGAATTTG
12121  GTCTTAACTGAGTAAAATGAAACCACGAAAAAGTTTGAGCAAGTGGAAGATGTGATCTAA
12181  TGTTTTGAAAGGATCACTCTGGCTTTGGGTCTGTTGTACCTCTGTTCCAGGAACCAAAAA
12241  ACCAACATTTCCTGGGGCATTTCTGATTTCAAATGTTCTTTTATTTGCCATTATCTTTTT
12301  TTTTTTTTTTTTTGAGACAGCATCTCACTAGGTTGCACAGGTTGGAGTGCAGTGGCATGA
12361  TCTCAGCCCACTGCAACCTCAGCCTCCCGAGTAGCTGGGATTACAGGCATGCACCACCAC
12421  ATCAGGCTAATTTCTGTATTTTTAGTAGAGACGGGGTTTCACTGTGTTGGCCAGGCTGGT
12481  CTTGAACTCCTGACCTCAGGTGATCCACCCTCCTTGGCCTCCCAAAGTACTGGGATTACA
12541  GGCGTGAGCCACTGCACCAAGCCCATTATCCACCTTTTAAACTTATTTTTGTTTTATTTG
```

EST DC400386 exon; in some of the *IL-15L ψ-SUPT5H* hybrid sequences that we amplified splicing had occurred at the site indicated in blue

EST DC400386 matches the first coding exon of *SUPT5H* gene (SUPT5H coding sequences are shaded yellow)

The arrow underlines the position of the reverse primer that we used to confirm the existence of *IL-15L ψ-SUPT5H* hybrid transcripts

12601 TTTGTTGAACAAATCAGTGTTTGCATCTACTACTGTGTTCCACTTACTTCTCAGGTGTCT
12661 GGTAGGAAATCTTTGCATTTTTGGAGTTGACATTTAGTAGGTAGCAATCACAAACCAGTA
12721 AACAGATGACAGGGTGATTATAGATCCCTTGTTTGATGCACAGAAACTTAAATTGGCTGT
12781 TAAATTAGTTGCCAATGCTTGTGAGGGAGATTTAACATAAAGATTAAGATTTCTTTTTCT
12841 GGGGAAATGGAAGGAGCTGGCACCCTGGGACACAGAGTTGTTCTGCAGAGCAGCCGTTTG
12901 CTTGATGAGTGGCTGCCCTACTTATACAGCTGGGAATTTTCTCCAGTTCCCAGGATATGT
12961 TAGTTGCCATTTGTCCTCTTGCTTGCCTTGTGGCAGAGTGAAGAAAAGGAAAAGATTTCA
13021 TGTGGCTGTGTTCTGGAAAACGTTTGGTAGATGGAGAGTGCTGTGTGTTTCGGGTGACTT
13081 GTCCCAGTCACTTATGTTGCCTGCTTATCCTCTGTGGACATTTGATTGCCAATCTTTATT
13141 GTGACCTTTGGAGAGTGCCTGGCTTCCAGGCTTGCAGTGAAGAGTTGGAGAAATTGGGAT
13201 GCTGTGGTGGGAGAGTGACAGGGAGGGCCTTTTCTCTAAGCTGTAGCACTGGATTCTTGG
13261 TCTCTGATTCATGGCTTGGAGACACTGCTTGGTGGTTAAAGTCAGGTGGAAATGAGACCA
13321 AGATCCCCAGTTCCATTTCCTGTCTGGGACATTCTCGTTTGCACTGTTACTAGTCTCACT
13381 TCCCCAACACTACACCATACTTGACCCCCCACCCCCGCCGCTGGCTCCTCTTCAGTTACC
13441 AGGTGAGGCAAGAGGGCACTGTGAGGCCTGTCCCCACTGCCAGGGACAGGATTGGATGGC
13501 CTTTTCAGACAGCCAAATGATGACATGGTCCTTGAAGGACAGGGTTCTGTGGCTAGACAG
13561 GTAGGGATGGCAAATAATGAGCTACTTTTGAGGGGAGCTGCTGGTTCACCCATCATGGCT
13621 CCAGTAACCTGTTCATTTATTCATTCAACAAATGTTCTGGCTGGGTGTGGTGGCTCACGC
13681 CTGTGATCCCAGCACTTTGGGAGGCTGAGGTGGGTGGATCACGAGGTCAGGAGATTGAGA
13741 CCATCCTGGCTAACATGGTGAAACCCCGTCTCTACTAAAAATACAAAAAATTAGCGTGGT
13801 GGTGGGTGCCTGTAGTCCCTGCTACTTGGGAGGCTGAGGCAGGAGAATGGCGTGAACGCA
13861 GGAGACAGAGCTTGCAGTGAGCAGAGATCGCACCATTGCACTCCAGCCTGGGAGACAGAG
13921 CGAGACTGCATCTCAAAAAACAAAAAACAAAAAACAAATGTTCCTTGACTTGGCATGTTG
13981 GGGTCACAGTCTCCCTTACAGAGATCTCAGTCTCAAGGCAGAGGCAGTTTTATAGATACA
14041 CAGTTACTGCAGGGGATGTTGAGGACGGTGAGAGCACATCCAAGCCCTGAGGTGAGCAGA
14101 GAGTGGTGGGGGCATCCTCCACTGGGAGGGAGGAGCCAGGTTTGGAAAGAGGCATCTCTG
14161 GGACCTGTCAGAGGGGAACTGATGACAAGGTTCCTAGAAATCAGCCTGTGGTCCTGTCAT
14221 AGTTTGGGTTGTTAAAATGTGCTTCATCGTGGGCTAGTCGAGATTTGTGTTAAGCAAATA
14281 TTTCTGAAGTCTTCTGTGCTTGGCCTTGGGCTGGGTGATGTTGGGGGACCCAGCAGTGCC
14341 TGAGATGGACTGGGCCCTACTATTGGGAGGCTCACAGTGGTCAGGGCTGTGATGGAGGGA
14401 GCCCAGAGAATTGTGAGCATAGAGGATGTGCCTGACTCTGCCTGGGGGAGAGGGGGCGGG
14461 GATGGCTGCCTGGAGGAGGAAGGTTAGTTTGGTTTTTTTTTTTTTGTCTTGGTGTCTTGCT
14521 CTGTCACCCAGGCTGGAGTGCAGTGGCACGATCTCAGCTCACTGCAGCCTCCACCTCACG
14581 GTCAAGCAGTTCTCCTCTCTCAGCTTCCTGAGTAGCTGGGATTACAGCGGTGCGCCACCA
14641 CACCTGGCTGATTTTTATATTTTTGGTAGAGATGGGGTTTCACCATGTTGGCCAGGGTGG
14701 TCTCAGAACTCCTGACCTCAAGTGATCGCCTACCTCGGCCTCCCAAAGTGCTGGGATTAC
14761 AGGCATGAGCCACCACGCTAAGCCTTTTTTTTTTTTTTTTGAGACAGGGTCTCACTTTGT
14821 CACCCATGCTGGGGTGCAGTGGTGTGATCTCGACTCACTGCAGCCTCAGCCTCCTGAGGA
14881 TCAAGCAATCCTCCTGCTTCAGCCTCCCAATGAACTGGGACAACAAGATTTTGCCACCAT
14941 GCCTGGTTAAGTTTTGTATTTTTGGTAGAGACAGGGCTTTGTCAGATTGTCCAGGCTGGT
15001 GTCAAACTCCTGGGCTCAAGTGATCCTTCTACCTTGGCCTCCCAAAGTGTTGGGATTACA
15061 GGTGTGAGCCCCCATGCCTAGCTAAGGAGGGAATATTTTCGTTGAAGTGTGAATGGGAGG
15121 CACAGTTTTTTTTTTTTGGAGATGAAGTCTCACTCTGTCGCCCAGGCTGGAGTGTAGTGG
15181 CACAATCGCGGCTCACTGCAACCTCTTCCTTCTGGGTTTAAGTGATTCTCCTGCCTCAGC
15241 CTCCTGAATAGCTGGGATTACAGGCGCATGCCACTGCGCCCAGGCCCAGAGGGGACTTTT
15301 TATACAGAAAACCAGCATGGATGATGGCCAGCACTGAGAGAGTATGGTCCAAGTAGTTGA
15361 GTGCAGCTGAGGCCTAGTGTTGGGGAGGCAGTCCTGAATGGTGGGGAACGGGGAGAGGCA
15421 GCAGGCAGAGCATGTGCTTCTAGTTCAGGCTGGCTGAAGCTGAGCAGGGGAACAAATGGT
15481 CAGGTTCATGTTTTAGATTACAGTTTTCTAAGACTGTACCATATATTCTAGGTTGGGGAT
15541 GATGAAAGGTGCTTTTAGGTGGAATAAGGACAAGACCTTAAGCTACATTGAATCATTTAG
15601 TGAGAATATTTCCTTTTCAGTTGTGTGTCTTATCTTGATTGGGTCATGAGAAAGTTTCAT
15661 CTTAGTGCTACTGTGTCTTGAACGCCTCTGGGTGGGAGAACAGGCCTTAGGCTTAGAGCC
15721 TGCAGCAGGCAGCAGTATCTTGCTGGGTCTTAATAACATTGTTTTGCTTTTATCTTTATA
15781 GTTACCTTGTGTTTATGACAAACCTTCAAAAAGTTTTAAATTTTGAAACATTGCATAATA
15841 TAAAAATGTTTTTATTTAAGAAGGTGAGTAGATTTGAAGAAAATACACATTGAGTATTAT
15901 CTAAAATGCTTGGGACAAGAAGTGTTTCAGATTTTGGATTTTTTTTTTTTTTGATTTTGTA
15961 ATATTTGCTGTGGAGCGTTCCCTAATCTGAAAATCCAGAATGCTCCAGTGAACATTTACT
16021 TTGAGCATGACCTTTTTTTTTTTTTTTTTTTTGAGACAGAGTCTTGCTGTGTCGCCTAGGC
16081 TGGAGTGCAGTGGTGCGATCTTGGCTCACTGCAACCTGTGCCTCCCAGGTTCAAACAATC
16141 CTCCTGCCTCAGCCTCCCAAGTAGTTGGGACTACAGGCGTGCCACCATGCCTGGCTAGGT
16201 TTTTTTTTTTTTGTATTTTTAGTAGAGATGGGGTTTCACTATGTTGGCCAGGCTGGTCTT
16261 GAACTCCTGACCTCAGGTGATCCGCGCGAGCCACCGCGCCCAGCCTGAGCATGACCTTTG
16321 AGCATTATGTCAGTGCTGAAAAAGTTTCAGATTTTGAAGCCTTTCAGATTTCAGTTTTTT
16381 GTTTGTTTGTTTTTTTGACAGTGTCTTGGTCCATTGGCCAGGCTGGAGTGCAGTGGTACA
16441 ATCTCGGCTGAGTGCAACCTTCACCTCCCAGTTTCAAGCGATTCTCCTACCTCAGCCTCC
16501 TGAGTAGCTGGGAGTACAGGTGCCCACCACCGTGCTGGGCTAGCTTTTGTATTTTTAGTA
16561 AAGACGGGGTTTCACCACATTGGCTAGGCGGGTCTTGTACTCCTGATGTCAGGTGATCCA
16621 CCCGCTTTGGCCTCCTAAAGTGTTGGGATTACAGGCATGAGCCACTGCACCTGACCAGAT
16681 TTCAGATTTTTGGATTAGGGATGTTCAACCTGTATTAGGTAAATGGATTGTAGTAATGAC
16741 ATGGAAGAAGTCGTGAAGTTCATGAGTTAAAGACTGAAGTTTGGTATGCATCCATTAATG
16801 TTGTTTCTGGTATGGAAAGAGGGATTAGAGGCCAGGAGAGTAGGGAGGAGGCTGGGGTAA
16861 GGATCCAGGTGTTAGAGGGCAGTGTCCTGGCCTAGGCTCAGATATATGGGGCTGGGGGAA

```
16921  AAGGATGGGTTGGAGAAATGATGTTTACAGGCAGAAAGGATGAGGATTTGGTGCCTGTCG
16981  GTAATTGGAGGAGGCCATCACAGGTCTAGGGGAAGACACTGAGGTCAGTTTGGGACACAG
17041  TGAGTATGAAAGGGCTCAGAGGCCATCTAGGGGAGGCATCCAGGATGCTGCTGGTGCATG
17101  GAGGTCTTGAGTTCTGGAAAAAAGTGGGACTCTATAGACAGATGAGGACACGTGTCATCT
17161  CTAGAGACAGGAACTGAGGTCATGGAAAGTAAAATGGACAGGGAGAGAGGGCATGGAGGG
17221  AGAACAAAGTGGCCCTGGACTGAGCCCTGGTAATTGATGTCAGATTTGAAGGGGTTAACA
17281  GTAAGAGGAGCTTGAAGAGGAGCTGGTGGAGTTTTGAGAAAAACCAGGAGAGCATGATAC
17341  GGCAGAAGCCAAGAGAGGAGCATTTCTCCAGGGAGGGCATGGTTGGGGTGCCAGATGCTG
17401  CTGGGATGGTAGGGAATACAGAATGGCCACAGAATTAGCAACAGGGGTGGCCAGTGATCT
17461  TCGTGAGCAGTGTGGACTAGAAACAGAGATGGGAGTCACTGTCACAGAGATGTCCACCAC
17521  CGCCAAGGGAGAGGACGAGATGGCCTTGGGGAGTTGTAGAATGGGAAGAGATGGGCCAGG
17581  TGCTGTGGCTCACACCTGTAATCCCAGCATTTTCGGAGGCAGAGGCGGATGGATCACCTG
17641  AGGTCAAGAGTTCGAGACCAGCCTGGCCAACATGGTGAAGCCACGTGTCTACTAAAAAAA
17701  TACAAAAATTAGCCAGGTGTGGTGGCGCACACCTGTAATCTCAGCTACTCAGGAGGCTGA
17761  GGCAAGAGAATTGCTTGAACCCAGGAGGTGGAGGTTGCAGTGAGCCAAGATTCTGCCATG
17821  GCACTCTAGCCTGGGAGACAGAGTGGGACTCTGTCTCAAAAAAAAAAAAAAAAAAGAGAGA
17881  GAAAGAAATGAGGCCCAAGGACCAAGCTTCATCAAGCTATTATAGTTAAAGACTCGGTGG
17941  AAAGTGAGCAAGAGGTCTAGTGCAGGGAGAAGCCCGGGTCAACTCCCAGGGTTGCTTGGG
18001  TGGCTGGGGAGATGGCAGTGCTGTCGCTGAGATAGGATGGTAACCAGAGAGGCCAGGCTA
18061  GAGTGAAAGGGATATATGCAGAGCAAAGTGGGCTATGATTGGCCAGGCCAATCTGAGGTC
18121  AGATTTAGGGTGAGGAACTTGGTTTTTGGATTTTTGGGGTAGCAGAATTCTGGTGCTACA
18181  ACCCTGCCTGACCCCTGTAGGTAGACGAAGAGCGGCGGAGTGCAGCGGGCAGTGAGAAAG
18241  AAGAAGAGCCTGAGGACGAAGAGGAGGAGGAAGAGGAGGAGGAATACGATGAGGAAGAGG
18301  AGGAAGAAGATGATGACCGACCCCCCAAGAAACCCCGCCATGGAGGCTTCATTCTGGACG
18361  AGGCTGGTATGTCATAGTGCTTGGCCAGTGCCGAGCAGAGGCTGAGCTTCTACTTTTAGT
18421  TCCATTTCTTTTTCTTTTTTTGAGGCGGAGTCTCGCTCTGTCGCCCAGGCTGGAGTGCAG
18481  TAGCATGAACTTGGCTCACTGCAAGTTCATGCCATTCTCCTGCCTCAGCCTCCTGAATAG
18541  CTGGGACTACAGGCGCCCGCCACCACGCCCGGCTAATTTTTCTATTTTTTAGTGGAGAC
18601  GGGGTTTCACCTTGTTAGCCAGGGTGGTCTCAATCTCCTGACCTCGTGATCTGCCCGCCT
18661  TGGCCTCCCAAAATGCTGGGATTACAGGCGTGAAGGACCAAAGTTCTTAACTTTTCTCGT
18721  GCTACGGCCCCTTCTGTAGTCTGGTGAAACCCTGTTCCCTTCTCAGAATCATCTTCTGAA
18781  ATGCATAAAACAAAATACATAGGATTCCAAAGAGGACAATTTATTGAAGTACAGTTATCA
18841  GAATATTTAAAAAGAACATTTTGGTGCATATGTTTCTTTGTTAACATATTTAGGTAATAA
18901  GACTTTGTAGGTAGTCTGAGAATCTAATTTTCAAGTAGCAGTGAGTGTAAATGCTGCCTT
```

The 3'-end of EST DC40038 6maps within a *SUPT5H* coding exon (yellow shading); the 5' and 3' end points of the *IL-15L*ψ-*SUPT5H* hybrid transcripts were not properly determined yet.

Fig. S4*E* *Homo sapiens* (human) hybrid *IL-15L*ψ *-SUPT5H* sequence of EST DC400386 with translation products

```
  1  gagtgtcgtgtgcggactgaagccctctgaagataccagcgagtggggagcagagccagc
      E  C  R  V  R  T  E  A  L  *  R  Y  Q  R  V  G  S  R  A  S
       S  V  V  C  G  L  K  P  S  E  D  T  S  E  W  G  A  E  P  A
        V  S  C  A  D  *  S  P  L  K  I  P  A  S  G  E  Q  S  Q

 61  agacggggaaaacaaaaatgatgacaccctctacaccccagatgatctctcaatgtcggc
      R  R  G  K  Q  K  *  *  H  P  L  H  P  R  *  S  L  N  V  G
       D  G  E  N  K  N  D  D  T  L  Y  T  P  D  D  L  S  M  S  A
     Q  T  G  K  T  K  M  M  T  P  S  T  P  Q  M  I  S  Q  C  R

121  cccgcactccgcccaggccagccctgcctctctcaagatggcgacctggaagtgcgtcgc
      P  A  L  R  P  G  Q  P  C  L  S  Q  D  G  D  L  E  V  R  R
       P  H  S  A  Q  A  S  P  A  S  L  K  M  A  T  W  K  C  V  A
     P  R  T  P  P  R  P  A  L  P  L  S  R  W  R  P  G  S  A  S

181  ccgtgccagccccggaaatctcgtgttaggcgaggtcaacgggtaggttctcgcgagagg
      P  C  Q  P  R  K  S  R  V  R  R  G  Q  R  V  G  S  R  E  R
       R  A  S  P  G  N  L  V  L  G  E  V  N  G  *  V  L  A  R  G
     P  V  P  A  P  E  I  S  C  *  A  R  S  T  G  R  F  S  R  E

241  acccgtcagccccagtcaggcgtcgtgcgaacagcagctggtaccgaaggcggaggtgga
      T  R  Q  P  Q  S  G  V  V  R  T  A  A  G  T  E  G  G  G  G
       P  V  S  P  S  Q  A  S  C  E  Q  Q  L  V  P  K  A  E  V  E
     D  P  S  A  P  V  R  R  R  A  N  S  S  W  Y  R  R  R  R  W


301  gcccgagagggaaccagcggggaaactgaggctcggggtggagcgcaggattgtgggacg
```

ATG codons are in orange, yellow-shaded asterisks relate to stop codons, the IL-15L exon2 encoded sequence is in green, and the encoded SUPT5H sequence is in red.

```
      A  R  E  G  T  S  G  E  T  E  A  R  G  G  A  Q  D  C  G  T
       P  E  R  E  P  A  G  K  L  R  L  G  V  E  R  R  I  V  G  R
     S  P  R  G  N  Q  R  G  N  *  G  S  G  W  S  A  G  L  W  D

361  cgccaaggctgctgtctttcccagcagcagcggaagatgtcggacagcgaggacagcaac
      R  Q  G  C  C  L  S  Q  Q  Q  R  K  M  S  D  S  E  D  S  N
       A  K  A  A  V  F  P  S  S  S  G  R  C  R  T  A  R  T  A  T
     A  P  R  L  L  S  F  P  A  A  A  E  D  V  G  Q  R  G  Q  Q

421  ttttccgaggaggaggacagcgagcgcagcagtgacggcgaggaggccgaggtagacgaa
      F  S  E  E  E  D  S  E  R  S  S  D  G  E  E  A  E  V  D  E
       F  P  R  R  R  T  A  S  A  A  V  T  A  R  R  P  R  *  T  K
     L  F  R  G  G  G  Q  R  A  Q  Q  *  R  R  G  G  R  G  R  R

481  gagcggcggagtgcagcgggcagtgagaaagaagaagagcctgaggacgaagaggaggag
      E  R  R  S  A  A  G  S  E  K  E  E  E  P  E  D  E  E  E  E
       S  G  G  V  Q  R  A  V  R  K  K  K  S  L  R  T  K  R  R  R
     R  A  A  E  C  S  G  Q  *  E  R  R  R  A  *  G  R  R  G  G

541  gaagaggaggaggaatac  558
      E  E  E  E  E  Y
       K  R  R  R  N
     G  R  G  G  G  I
```
