# Supplementary material for: Identification of a gene for an ancient cytokine, interleukin 15-like, in mammals; interleukins 2 and 15 co-evolved with this third family member, all sharing binding motifs for IL-15Rα
Source: Immunogenetics. 2013 Nov 26;66(2):93–103. doi: 10.1007/s00251-013-0747-0 (PMC3894449; doi:10.1007/s00251-013-0747-0)

Supplementary Figure 5 (Fig. S5).
IL-15L specific RT-PCR using tissues of *Bos taurus* (cattle) and *Oryctolagus cuniculus* (rabbit), or permanent cell lines of these two species

Table of Contents:

**Legends to Figures S5*A*-to-*D***		Page 2
**Fig. S5*A***	Tissue distribution of bovine *IL-15L* transcripts	Page 5
**Fig. S5*B***	Tissue distribution of rabbit *IL-15L* transcripts	Page 5
**Fig. S5*C***	Detection of *IL-15L* transcripts in permanent bovine cell lines	Page 5
**Fig. S5*D***	Detection of *IL-15L* transcripts in permanent rabbit cell lines	Page 5

Legends to Figures S5A-to-D

IL-15L specific RT-PCR using tissues of *Bos taurus* (cattle) and *Oryctolagus cuniculus* (rabbit), or permanent cell lines of these two species.

General note: All the fragment types indicated by arrowheads were sequenced. For the lower pictures in each of the sections A-to-D the primers GAPDH-F and GAPDH-R were used in combination with 25 amplification cycles to check sample quality; the white triangle points to the expected 338 bp *GAPDH* fragment.

Fig. S5*A* Tissue distribution of bovine *IL-15L* transcripts. The cDNA template samples were derived from bovine thymus (1), lymph nodes (2), lung (3), skin (4), duodenum (5), colon (6), and testis (7). For the upper picture PCR was performed with primers Cow-IL-15L-ex1-F2 plus Cow-IL-15L-ex3-R2 using 40 cycles. White arrowhead, 127 bp exon1-exon2-exon3/4 sequence; black arrowhead, similar but including the 129 bp intron between exons 1 and 2; gray arrowhead, primer-dimer.

Fig. S5*B* Tissue distribution of rabbit *IL-15L* transcripts. The cDNA template samples were derived from rabbit spleen (1), lung (2), duodenum (3), colon (4), liver (5), kidney (6) and testis (7). For the upper picture PCR was performed with primers Rab-IL-15L-ex1-F plus Rab-IL-15L-ex3-R using 37 cycles; white arrowhead, 185 bp exon1-exon2-exon3/4 sequence; gray arrowhead, 140 bp exon1- exon3/4 sequence (thus without exon2). For the middle picture PCR was performed with the primers Rab-IL-15L-5'UTR-F plus Rab-IL-15L-3'UTR-R2 using 40 cycles; the reaction extension time of 1 min should have been sufficient to amplify the expected <1 kb sequences including exon

3/4, but only a variety of sequences without this exon was amplified (bracket; Fig S3*C* reaction No.3).

Fig. S5*C* Detection of *IL-15L* transcripts in permanent bovine cell lines. RT-PCR results were obtained using RNA samples derived from the following bovine permanent cell lines: (1) MDBK (CCLV cell bank number: 0261/batch 3435, established from: kidney, morphology: epitheloid), (2) MDBK (0261/batch 3436, kidney, epithelioid), (3) MDBK-K (0581, kidney, epithelioid), (4) PES (0154, kidney, epithelioid), (5) KOP-R (0244, oesopharynx, fibroblastoid), (6) KMU-R (0098, muscle, fibroblastoid), (7) KLU-R1 (0031, lung, epithelioid/fibroblastoid), (8) KTR-R1 (0049, trachea, fibroblastoid), (9) KHO-R (0041, testicle, fibroblastoid), (10) KSN-R (0050/batch 1332, nasal mucosa, epithelioid), (11) KSN-R (0050/batch 1333, nasal mucosa, epithelioid), (12) FE-R (0978, udder, fibroblastoid), (13) KE-R (0977, coronal band, epithelioid), and (14) KM-R (0061, spleen, fibroblastoid). For the upper picture RT-PCR was performed with the primers Cow-IL-15L-5'UTR-F plus Cow-IL-15L-3'UTR-R1 using 40 cycles; white triangle, 479 bp sequence with consensus *IL-15L* ORF.

Fig. S5*D* Detection of *IL-15L* transcripts in permanent rabbit cell lines. RT-PCR results were obtained using RNA samples derived from the following rabbit cell lines: (1) RL1-Z-R (0751, liver, epithelioid), (2) KMK (0901, bone marrow, fibroblastoid), (3) RLI-R (0750, liver, epithelioid), (4) PBK-R (0920, blood, fibroblastoid), (5) KaNie (not indexed, kidney, epitheloid/fibroblastoid), and (6) KFT-R (1014, thymus, fibroblastoid). For the

upper picture PCR was performed with the primers Rab-IL-15L-5'UTR-F plus Rab-IL-15L-3'UTR-R1; white triangle, 474 bp sequence with consensus *IL-15L* ORF.

Fig. S5

IL-15L specific RT-PCR analyses using tissues of *Bos taurus* (cattle) (A) or *Oryctolagus cuniculus* (rabbit) (B), or permanent cell lines of these two species (C: cattle, D: rabbit).

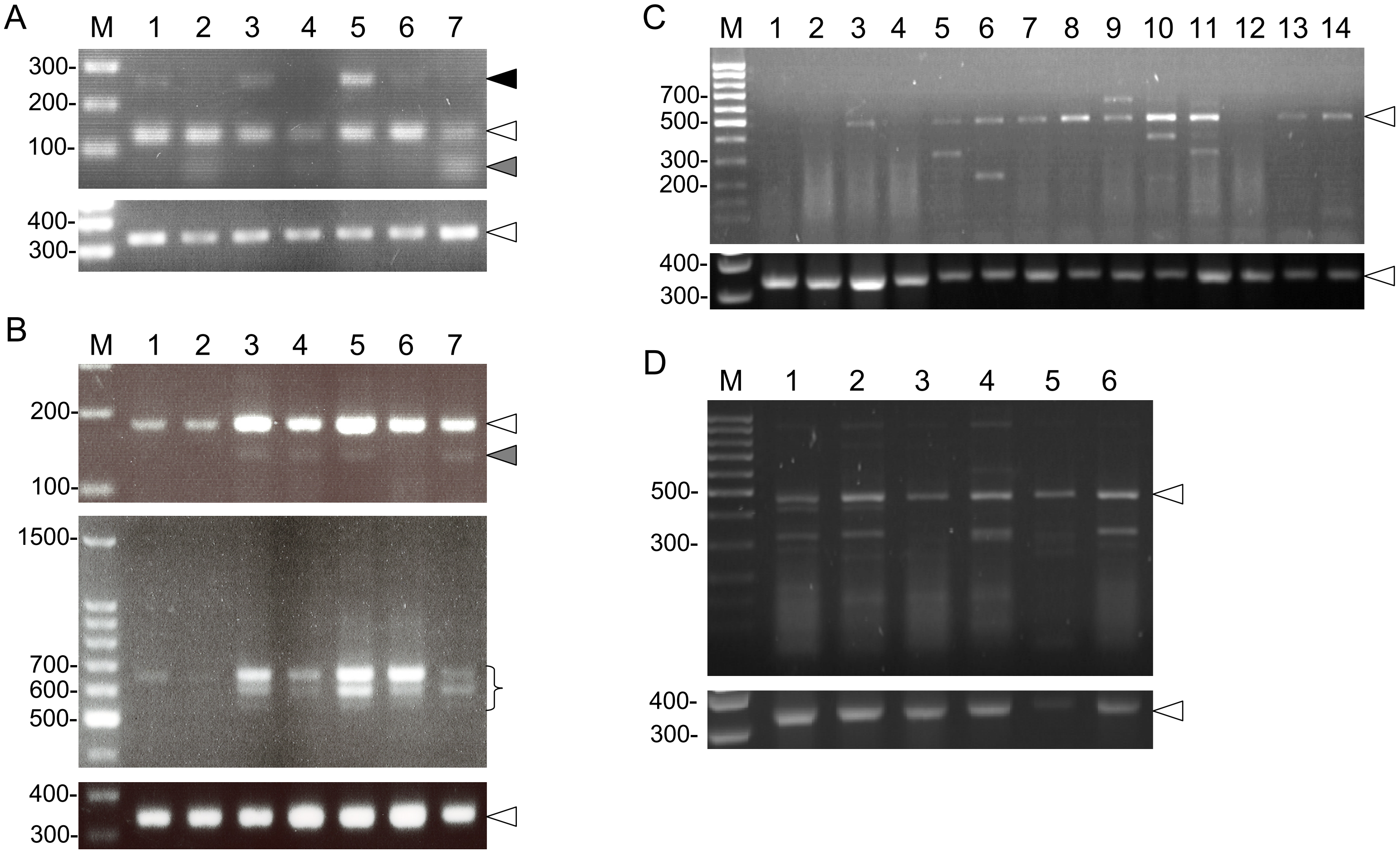

Supplement: Supplementary file 8 — (PDF 1208 kb) [file 251_2013_747_MOESM8_ESM.pdf]
