# Supplementary material for: Identification of a gene for an ancient cytokine, interleukin 15-like, in mammals; interleukins 2 and 15 co-evolved with this third family member, all sharing binding motifs for IL-15Rα
Source: Immunogenetics. 2013 Nov 26;66(2):93–103. doi: 10.1007/s00251-013-0747-0 (PMC3894449; doi:10.1007/s00251-013-0747-0)
Supplement: Supplementary file 9 — (PDF 306 kb) [file 251_2013_747_MOESM9_ESM.pdf]

Supplementary Figure 6 (Fig. S6).

Analyses of deduced IL-15L amino acid sequences and related cytokines

Table of Contents:

Legends to Figures S6*A*-to-*C* Page 2

**Fig. S6*A***	Alignment of deduced IL-15L amino acid sequences and related cytokines	Page 8
**Fig. S6*B***	Phylogenetic tree inferred by Neighbor joining method for relatively well conserved regions of IL-15L and related cytokines	Page 12
**Fig. S6*C***	Similarity levels (percentages of identical amino acids) between representative IL-15L molecules and related cytokines	Page 13

Legends to Figs. S6*A*-to-*C*

Fig. S6*A*

Alignment of deduced IL-15L amino acid sequences and related cytokines.

This figure is similar to main text Fig. 3, except that all deduced intact tetrapod IL-15L molecules, more teleost fish IL-15L molecules, the partial IL-15L molecules deduced for lizard, python, Chinese softshell turtle and spotted gar, and additional IL-2, IL-4, IL-15, and IL-21 molecules, are included.

Basic residues are indicated in red, acidic residues in blue, and green residues are more hydrophilic than the orange ones [reference 1]; cysteines are indicated in purple. From structural superimposition of multiple short-chain type I cytokines (IL-2, IL-4, IL-5, GM-CSF and M-CSF), Rozwarski and coworkers [2] distinguished a common inner core of which 30 residues were relevant for IL-2 and IL-4 (gray shading). Most of these buried residues lack hydrogen bonding capacity [2], which was helpful for making the alignment, which was done manually based on considerations regarding overall sequence similarity, molecule structure, functional motifs, degree of relatedness and also intron-exon organization of the gene. However, especially the leader peptides and parts of the loop regions are so divergent that some of the alignments are tentative only, and even some of the alignments of α-helices A and C remain highly questionable. Cysteine pairs found in some of the sequences are indicated above the alignment. The cysteine pair indicated by olive-green C's is characteristic for both teleost fish IL-2 and teleost fish IL-2L, suggesting that the corresponding genes originated from a rather recent duplication of a common ancestor. The downward triangles, diamonds, and numbers 1 and 2 above and

within the alignment indicate (putative) receptor binding residues as explained in the main text and as also depicted in main text Fig. 3. The blue lines under the alignment indicate rather well conserved regions around α-helices B and D which were used for the phylogenetic analysis described in Fig. S6*B*.

For lay-out reasons an amino acid stretch in the N-terminal region of mouse IL-2 situated between two glutamines was removed (indicated as AqqH instead of AQQQQQQQQQQQH) and also a glutamic acid of stickleback IL-2 was removed (indicated as KeC instead of KEEC).

For full names of species see main text Fig. 1, except for *Anas platyrhynchos* (wild duck, mallard), *Gallus gallus* (chicken), *Xenopus tropicalis* (Western clawed frog), and *Oncorhynchus mykiss* (rainbow trout). Mammalian and reptilian IL-15L were identified in the present study (Fig. S2). The other sequences are available as GenBank accessions or were predicted by us from Ensembl databases: gar IL-15L, Ensembl “LepOcu1”; zebrafish IL-15L, GenBank BAD69558; medaka IL-15L, Ensembl “MEDAKA1”; stickleback IL-15L, Ensembl “BROADS1”; fugu IL-15L, GenBank AAZ20137; pufferfish IL-15L, Ensembl “TETRAODON8” and described in reference [3]; cattle IL-15, AAA85130; human IL-15, AAA21551; mouse IL-15, AAA75377; opossum IL-15, Ensembl “BROADO5”; platypus IL-15, Ensembl “OANA5”; chicken IL-15, GenBank AAD38392; duck IL-15, GenBank ABF47546; painted turtle IL-15, Ensembl “ChrPicBel3.0.1”; lizard IL-15, Ensembl “AnoCar2.0”; frog IL-15, GenBank ABS44963; gar IL-15, Ensembl “LepOcu1”; zebrafish IL-15, GenBank AAZ43090; trout IL-15, GenBank AJ555868; stickleback IL-15, EF513158; pufferfish IL-15, GenBank AAZ23017; cattle IL-2, GenBank AAA30586; human IL-2, GenBank 0904306A; mouse

IL-2, GenBank 1102250A; opossum IL-2, GenBank XP_003341421; chicken IL-2, GenBank AAC96064; duck IL-2, GenBank AF294322; painted turtle IL-2, Ensembl "ChrPicBel3.0.1"; Chinese softshell turtle IL-2, Ensembl "PelSin_1.0"; lizard IL-2, Ensembl "AnoCar2.0"; frog IL-2, Ensembl "JGI_4.2" in combination with ESTs EL670866, EL670867, EL717500, and EL717501; gar IL-2, Ensembl "LepOcu1"; zebrafish IL-2, Ensembl "Zv9"; trout IL-2, GenBank NM_001164065; stickleback IL-2, GenBank NP_001254611; pufferfish IL-2, Ensembl "TETRAODON8"; stickleback IL-2L, GenBank NP_001254612; pufferfish IL-2L, Ensembl "TETRAODON9" and GenBANK EF513164; cattle IL-21, GenBank NP_942129; human IL-21, Genbank AAG29348; opossum IL-21, Ensembl "BROADO5"; chicken IL-21, GenBank AAX40651; painted turtle IL-21, Ensembl "ChrPicBel3.0.1"; lizard IL-21, Ensembl "AnoCar2.0"; frog IL-21, GenBank ABS50434; gar IL-21, Ensembl "LepOcu1"; zebrafish IL-21, GenBank ABM46913; pufferfish IL-21, ABB05043; human IL-4, GenBank AAA59149; chicken IL-4, GenBank NP_001007080; zebrafish IL-4/13B, NP_001164211.

References in this figure legend:

[1] Hopp T-P, Woods K-R (1981) Prediction of protein antigenic determinants from amino acid sequences. *Proc Natl Acad Sci USA* 78(6):3824-3828.

[2] Rozwarski D-A, et al. (1994) Structural comparisons among the short-chain helical cytokines. *Structure* 2(3):159-173.

[3] Fang W, Xiang L-X, Shao J-Z, Wen Y, Chen S-Y (2006) Identification and characterization of an interleukin-15 homologue from Tetraodon nigroviridis. *Comp Biochem Physiol B Biochem Mol Biol* 143(3):335-343.

Fig. S6*B* Phylogenetic tree inferred by Neighbor joining method [reference 1] for relatively well conserved regions of IL-15L and related cytokines.

Stretches around α-helices B and D of molecules compared in Fig. S6*A*, and in that figure indicated with a blue line under the alignment, were assembled and kept aligned as in Fig. S6*A* for phylogenetic analysis by computer software. Bootstrap values lower than 50% are not shown, and a few >50% values in cramped IL-15L regions in section (a) were deleted for lay-out reasons. Yellow background highlights the newly found tetrapod IL-15L. In section (b) a cramped part of the tree shown in section (a) is enlarged. Most cytokines cluster with their orthologues in other species which is depicted by shared background colors. However, resulting from the high sequence plasticity of this cytokine family, a few sequences "dance out of line" and their background was left white. The tree is inconclusive (low bootstrap values) on the relative position of the different cytokines, a problem which we could not solve by using other software programs, full-length alignments, and/or automated alignments (not shown).

The optimal tree with the sum of branch length = 26.12392397 is shown. The tree is drawn to scale, with branch lengths in the same units as those of the evolutionary distances used to infer the phylogenetic tree. The evolutionary distances were computed using the Poisson correction method [2] and are in the units of the number of amino acid substitutions per site. The analysis involved 74 amino acid sequences. All ambiguous positions were removed for each sequence pair. There were a total of 48 positions in the final dataset. Evolutionary analyses were conducted in MEGA5 [3].

References in this figure legend:

[1] Saitou N, Nei M (1987) The neighbor-joining method: A new method for reconstructing phylogenetic trees. *MolBiol Evol* 4(4):406-425.

[2] Zuckerkandl E, Pauling L (1965). Evolutionary divergence and convergence in proteins. Edited in *Evolving Genes and Proteins* by Bryson V and Vogel H-J, pp. 97-166. Academic Press, New York.

[3] Tamura K, et al. (2011) MEGA5: Molecular Evolutionary Genetics Analysis using Maximum Likelihood, Evolutionary Distance, and Maximum Parsimony Methods. *Mol Biol Evol* 28(10):2731-2739.

Fig. S6*C*

Similarity levels (percentages of identical amino acids) between representative IL-15L molecules and related cytokines. Molecules were aligned full-length as in Fig. S6*A*, and the number of identical aligned residues was divided by all aligned residues and then multiplied by 100%; residues without match in the compared molecule in the Fig. S6*A* alignment were neglected (insertion and deletions, alias “indels”, were neglected). Colored backgrounds help to estimate amino acid identity percentages at a glance:

-Blue: <15%

-Green: ≥15% and <20%

-Yellow: ≥20% and <25%

-Orange: ≥25% and <40%

-Red: ≥40%

The IL-15L, IL-15 and IL-2 sequences share similarity levels with each other that generally are slightly above 20% amino acid identity. That is, with the exception of turtle and mammalian IL-2 (the sequences separated by dashed line), which all lack a family consensus disulfide bridge (green C's above the alignment in Fig. S6*A*), and which share on average <20% amino acid identity with IL-15L and IL-15 molecules. Also IL-21 molecules tend to have a somewhat lower level of similarity with the IL-15L, IL-15 and IL-2 molecules, supporting that IL-21 forms the phylogenetic outgroup within the IL-2/-15/-15L/-21 family. The similarity levels cannot clarify which of the IL-15L, IL-15 and IL-2 molecules are related more closely to each other.

Supplementary figure 6*A* (Fig. S6*A*).
Alignment of deduced IL-15L amino acid sequences and related cytokines

```
                              Exon 1a                  Exon 1b
cattle IL-15                                   MRIL       KPYLRSTSIQCYLCLLLNSHFL----TEAGIHVFILG
human IL-15                                    MRIS       KPHLRSISIQCYLCLLLNSHFL----TEAGIHVFILG
mouse IL-15                                    MKIL       KPYMRNTSISCYLCFLLNSHFL----TEAGIHVFILG
opossum IL-15                    MLVMSRAPQKTTRAYSRPIRGR   KSYLRSSPIQCYLYLILNGHFFSFL-TEAGTHFFILS
platypus IL-15                   MLVISRAPRKSSGACSRSIGGR   KTCLGSTCMPCYLCLILHSHFFSLLNNKTGIHFFILS
chicken IL-15                    MLGMAQPTQNSAGARRR-PESQ   KTHVKSICLQYQLYLLLNSHFFCLLKNKTGLTIFFLC
duck IL-15                       MLGMAWPTQKSAGMYSR-LESQ   KTHLKSIGLQYHMYLLLNSHFFFLLKNKTGLTIFFLC
lizard IL-15                                                                 MSNYFWSSFINVSLFILC
frog IL-15                                                  MGQLLKLPYFWIQMIYLL------LAYNYTVLQM
gar IL-15 (partial)                                       KSQ-VSHV-YIYCCF--HLFLGCPLTSEAWLSLFFLS
zebrafish IL-15               MISVTLFLVFIAGLWINKPAKLKSM   RTG-RCAC--NTLCF--ENHMECHWNSEVWNSILILS
trout IL-15                        MTGFLTVLLFCIRLLERRTK   KSV-RWIC-LFWGFH-YYPH--QRLNIELWNCFIILS
stickleback IL-15                     MTDFMTVFPEILVKASQ   KGF-QLRS-TCHLFQ--ESH-----KPQVWLCFLVLS
pufferfish IL-15                    MSGFMTATAQPTCARGHRS   RGV-SFHS-TCCLCR--EF------KT--WFPFFLLC
```

(this figure continues on the next pages)

```
                   Exon 1 (1c)
                                                                                          αA
                                                                                  ·1       ·10
                                                                                C  C  ▼                C
cattle IL-15L                     MGVGRVSMWLLWTTLLLVLPLGGLG---------------------PLLCPREPFYFLIAITKML
sheep IL-15L                        MGKVSMWLLWTTLLLVLPLGGLG---------------------PPLCPREPFYFLVAITKML
pig IL-15L                                MWHLWTILLLVPPLGGLG---------------------PPLCPREPFYFLIAIMKML
horse IL-15L                        MGRVPIWPLWTIVLLMRPLGGLG---------------------PPLCPREPFYFLLAIMKML
rhinoceros IL-15L                   MGRVSIWPLWTILLLMWPLGGLG---------------------PPLCPREPFYFLVAIMKML
cat IL-15L                               MWPLWTILLLVRPLGGLG---------------------SPLCPREPFYFLVAIMKML
ferret IL-15L                            MWSLWTILLLVRPLGGLG---------------------SPLCPREPFYFLVAIMKML
hedgehog IL-15L                          MWALWAILLLVWPSGGLG---------------------SPLCPKEPFYFLVALMKML
shrew IL-15L                             MWALWTILLLVWPLRVLG---------------------SSLCPKEPFYFLLAIMKKL
human IL-15Lψ                            MLLLWIILLLVQPWEGLG--------------------TXPLCWREPFYFLAALxKIP
lemur IL-15L                             MWPLWTILLLMQLWGSLG---------------------APLCRREPFYFLVAIMKML
rabbit IL-15L                            MWPLWTILLLGGLLGGLG---------------------TPLCRREPFYFLVAIMKIL
pika IL-15L                              MWPLWTILLLGGLLGGLG---------------------TPLCRREPFYFLLAIMKML
hyrax IL-15L                             MWPLWIILLLVPPLWGLG---------------------PPLCSQEPFKFLVAIKKIL
opossum IL-15L                           MWLFWIFLVLVHPLGTPP-------------------LRPPSCPLEPFQGLIALLEAL
wallaby IL-15L                           MWFLWAFLVFVRPLGGPP-------------------LGPPSCPWEPFEVLIAIMEAL
platypus IL-15L                          MWSFRVLLLLAQLLVCLC----------------------PPCPREPFDGLVTITEAL
p. turtle IL-15L                         MRGLHALLLVSLLTCFSV-------------------RGRRVCGKEAIRVLQNVTKLL
C. turtle IL-15L (partial)          ASLPPATRALHPLLWVSLLMCLSA-------------------GGSPLCGMETIRLLQNITKLL
zebrafish IL-15L    MTNLLLRESW--------TFDMRTDLLYYSMLCTSLSLYLLIMLTRQIKS---------------QSVCSRESVEMVKQIAAKLNNV
medaka IL-15L                       MLRGGPAVLGVFLCFMFPPMLTSP-------------------SNQCPRTIITLVEQLRKLNCLQ
gar IL-15L (partial)                            LCCLVAMTPWAG-------------------GHRALCSREAVADLQSLQSNISG
stickleback IL-15L                  MLGGRSALASVFLCFVCLLGPTPPAAG--------------------ICTTDLPKQVKSMIKIAPRL
fugu IL-15L         MLNLDAARVWDHVLSSGDTLASRVRWRGGRMLCVIFLLTVALQLAAQ------------------KACSYDIISVQTLINSTSSL
pufferfish IL-15L    MTLEAARLRDHARSS---VCGSGAMRGGQTLCVLLLLSLTLQLAA------------------NNPCSRDIKQNVQSLMKSVTNQ
cattle IL-15                                     CISASLPKTEA------------------------NWQYVINDLKTIEHLIQ
human IL-15                                      CFSAGLPKTEA------------------------NWVNVISDLKKIEDLIQ
mouse IL-15                                      CVSVGLPKTEA------------------------NWIDVRYDLEKIESLIQ
opossum IL-15                                    CISAHLPKTEA-----------------------SNWEFVIRDLELLKDL
platypus IL-15                                   CISVCLTKTEG-------------------SRDNGTWQPVIDDLELIKNMTK
chicken IL-15                                       AYVPKTEA---------------------NHCKWSDVLKDLELIKTSE
duck IL-15                                          AYIPNTEG---------------------DHCKWTEVLKDLEQIKTSK
p. turtle IL-15 (partial)                          AYLPGIEA---------------------ERRRWEAVIDDLHEIKDTSD
lizard IL-15                                          SCFQEV-------------------QTRRFVSWEPVLKDLEQIIKIPTK
frog IL-15                                            VTTSEA------------------------VRVKAILDNINHIKK
gar IL-15 (partial)                              CVYANMPTTEA-------------------------LDQALREMQTCL--ESNLSLLK
zebrafish IL-15                                  CLSALLPLTEG---------------------QAGHPEQGLEDLLTIL--ENHKKLFT
trout IL-15                                      CLSATAHLPIA-----------------GAAETHGMTIDDVKELQSEL--KNLKSTIE
stickleback IL-15                                  LLSTSTCAA------------------------PGAAKLAHLQSCLGTEYLKKAIE
pufferfish IL-15                                   FLSLYTCHA------------------------AVVETSEVKTCI--TAIQPAIE
                                                                                          ◇
cattle IL-2                          MYKIQLLSCIALTLAL-VANG-------APTSSS-----TGNTMKEVKSLLLDLQLLLEKVK
human IL-2                           MYRMQLLSCIALSLAL-VTNS-------APTSSS-----TKKTQLQLEHLLLDLQMILNGIN
mouse IL-2                           MYSMQLASCVTLTLVL-LVNS-------APTSSS-TSSSTAEAqqHLEQLLMDLQELLSRME
opossum IL-2                         MSKVPLLLCVALTLAV-LAGG-------APTSPPP----TS----LLEYLLLDLQEAHEKLS
chicken IL-2                        MMCKVLIFGCISVAMLMTTAYG-------ASLSS-------AKR--KPLQTLIKDLEILENIKN
duck IL-2                            MCKVLIFSCLSVLMLMTTAYG-------APLS-------EKD--NTLKTLIKDLENLGTSMN
p. turtle IL-2                       MCKVLV-TCYIPLLLVIAANG-------KPFISPNGTPFTSPNEEYLKYAIDDVNELEKMTK
C. turtle IL-2                       MCKVLV-TCYILLLLAIAANG-------NTLCS-------LHK--KHLEHAIADVIELEKMNI
lizard IL-2                          MNKVSL--CFIAL-ILTTIGG-------MPIINP----------DYLEAMQIDIRLLLSILP
frog IL-2                            MCKILVL-CLLAVTLLVFSQG-------APTEN-------VAKQLELLRAMGNDLQRMKTSSK
zebrafish IL-2                    MMMRMSALHWICALCLTLLCSLSA-------QP----------VKR--EVTSEIEDNLQHLKKVITNYEC
trout IL-2                        MDRRYRISFLTLFLTGCL--QG--------------------------NPIPRLLAGIDYLEE---NITC
stickleback IL-2                  MFFFIQMAYWILL-SDCL--LA--------------------------RSFPLSDFRAITQS--HVEC
pufferfish IL-2                   METFNRIYFGIVIVCVCL--PANS-------NP------------------IRSDDSDIGVMKK---NVIC
stickleback IL-2L                      MEHSLRTALWVFCLFGFLQA--------------------TPP--CYGQGDLGFCFLQQ---HVKC
pufferfish IL-2L                        MTWIAIALWLVPLIGQVQV-------------RPVSNDTEPLGPLNMDDLGLRFLDK---RL-C
cattle IL-21                     MRW-PGNMERIVI-CLMVIFSGTVAHKSSS--------------QGQDRLFIRLRQLIDIVDQLKNYVNDL
human IL-21                      MRSSPGNMERIVI-CLMVIFLGTLVHKSSS--------------QGQDRHMIRMRQLIDIVDQLKNYVNDL
opossum IL-21                            MEGIVIYCLVVIFSGMVASKPS----------------YPCRLQSRMIQLVETVEQLKSCVNDT
chicken IL-21                            MERMIIFCMLFFCFSMVLT-------------------ATSPKAMKYKQLSKTIDHLKDVVKDK
p. turtle IL-21                          MERMIIFCLFFFCSSMLLS-------------------AAAPTKLRYKELVKTVIELKKIVKVK
lizard IL-21                             MEKV--CCLLFVCSIVLVQG----------------------ERTMVFEELLPSVTSLMEM
frog IL-21                               MANVLLWCLLACSCCILIDA------------------KLPSIERCREIKKGIEEWIIKQANKT
zebrafish IL-21                           MKACV-CFLLMCVLAAQA------------------EESPKILTLSKVMNELKKIQQGL
trout IL-21                               MKLLV-CYLLAITCVLVDA------------------DKGERILKLTEVIKELKALNRTVT
stickleback IL-21                         MKLVVFCFLAAWCGSLVGASTS-----------RPTTRPPMFRRKLEEVIGHLHRVKESQQ
pufferfish IL-21                          MKQLVFCLFAVCCWWLADA------------------SSAECSERKLEEVRRELEGVNNTLQ
human IL-4                          MGLTSQLLPPLFFLLACAGNFVHG-------------------HKCDITLQEIIKTLNSLTEQK
chicken IL-4                           MSSSLPTLLALLVLLAGPGAV----------------PTLCLQLSVPLMESIRIVNDIQ-GE
zebrafish IL-4/13B                          MRTFLLLVLTLVPVSES------------------KLKIDEILMEIIQSVNGILNGKGEKM

                Exon 1d

Frog IL-15   IFVEFNHANYE
```

```
                Exon2                           Exon 3 (3/4, 3a)
                                                                   αB                                     αC
                          ·20      ·30                             ·40      ·50                             ·60      ·70      ·80
                           C                                   C    CC                 C        C                      ▼
cattle IL-15L            ENKNDGSLYTP--DNLL          VCPAETLRCFRLELSVIGFEE----------------GPSVGIVVFRLQRLLDALGSQLWVI-----
sheep IL-15L             ENKNDGSLYTP--DNLL          VCPAETLRCFRLELSVIGFEE----------------GPSVGIVVFRLQRLLDTLGSRLWVT-----
pig IL-15L               GNKNDGTLYTP--DDLS          VCPAETLGCFRLELSVIGFEE----------------GPLVGTAVFRLQRLLDALGSRLWVT-----
horse IL-15L             GNKNDGTLYTP--DDFS          VCPAETLGCFRLELSVIGFEE----------------GPSVEIAVFRLQRLLDALGSRLWGT-----
rhinoceros IL-15L        GNKNDGTLYTP--DDFS          VCPAETLGCFRLELSVIGLEE----------------GPSVGTAVFRLQRLLDALGSRLWGT-----
cat IL-15L               GNKNDGTLYTP--DDLS          VCPAETLGCFRLELSVIQFEE----------------GRSMGIAVFRLQRLLDALGSRLWVT-----
ferret IL-15L            GNKNDGTLYTP--DDLS          VCPAETLGCFRLELSVIQFEE----------------GPSLGIAVFRLQRLLDALGSRLWVT-----
hedgehog IL-15L          GNKNYGTLYTP--DDLS          VCPAETLGCFQLELSVIVFEE----------------DPTVVIDVFRLQRLLGALGSRLWAT-----
shrew IL-15L             EKKNDGTLYTP--NDFS          VCPAETLGCFRLELSVIAFEE----------------GPSVALAVFRLQRLLESLGSRLWET-----
human IL-15Lψ            ENKNDDTLYTP--DDLS          VCPAETLGCSRLELSVIGFEE----------------GPSMGTAVFQLQYLLDLLGSRLWVA-----
lemur IL-15L             GNKNDGTLYTP--DDLS          VCPAETLGCFRLELSVIGFEE----------------GPSVGTAVFRLQRLLDALGSRLWVA-----
rabbit IL-15L            GDKNDGTLYTP--DDLS          VCPAETLGCFRLELAVIGFEE----------------GPSVGIAVFRLQRLLDALGSRLWVA-----
pika IL-15L              GDQSDGTLYTP--GDLS          VCPVESLGCFRLELAVIGFEE----------------GPSVGMAVFRLQRLLDALGARLWVA-----
hyrax IL-15L             GNRNDGTLYTP--DDLS          VCSVETLGCFRLELSVIGFEE----------------GPSVGIVVLWLQRLLDVLGSQLWVA-----
opossum IL-15L           GTRNDGTLYTP--DNLS          VCPMEILHCFGVELSVIGHEE----------------GLPTVRAVNRLQRSLGALGPHLWGARVGA-
wallaby IL-15L           GNRNEGTLYTP--DSLS          VCPMETLHCFGVELSVIGYEE----------------GPAVVRAVIRLQRSLTALGSHLWGARWGA-
platypus IL-15L          GNHNDGILYTP--DNLS          VCPAETLGCFGAELGVIGLEH-----------------REQAVEVARLQCHLEILGFLLPPR-----
p. turtle IL-15L         GDATDGTLYTP--EDIT          VCMAENLNCFHTELRVIQWEH-----------------REHTESLSLLIRHLSQLEKL----RTCK-
C. turtle IL-15L (part.) GDTTDGMLYTP--EDIT          VCTVENLNCFHTELQVIRWEH-----------------REHTKSLSLLIRNLSQMEQLKLKARTCK-
lizard IL-15L (partial)                             VCYVENLDCFVEELEVVQEEE-----------------EEHAEILSRLLLRLQQMKLKWQRENGTK-
python IL-15L (partial)                             VCYADNLDCFYMELRVIQEEQ-----------------EEHTEILSRLILRLDQLRRKLKGTSRIS-
gar IL-15L (partial)      NRDNCTLYTANVSDYK          ECPNSTLSCFIEELGVLILEF---------------GHTRTNVSSKLLRRLHRLNTK
zebrafish IL-15L           ENDCMMYTPTQANYK          NCSKSTITCFAFEVSVLSVEI-----------------QVESLAFRQLQRILNRVTKR
medaka IL-15L             DCPDSTLYTPDVQDFK          RCPSTTLRCFVAELQVLRAEL--------------THDGCIAVSNRTARRLEVWSNRYK
stickleback IL-15L        NHLGSRLYTQTATDFQ          NCIVSTLKCFSAEMDVLINEW----------KLANVKIPQKKTLVSGLKRLADRFSLAKNT
fugu IL-15L               TGLDSRLYTPTINDYD          KCPESTLMCFAEEVKVLKYES----------------EIEAFSLIRKLRRIAGSFKK
pufferfish IL-15L           DSMLYTPTTTDYR          KCPKSTMRCFTEEAKVLEREW----------------KNMTVRLVKKLKVITDDLNQ
cattle IL-15             SIHMDATLYTE--SDAH         PNCKVTAMQCFLLELRVILHESKN---------------ATIYEIIENLTMLANSNLSSIE
human IL-15              SMHIDATLYTE--SDVH         PSCKVTAMKCFLLELQVISLESGD---------------ASIHDTVENLIILANNSLSSNG
mouse IL-15              SIHIDTTLYTD--SDFH         PSCKVTAMNCFLLELQVILHEYSN---------------MTLNETVRNVLYLANSTLSSNK
                             1   2                           2  22  2  11
opossum IL-15          TNKTDIS-SLYTE--SDAH         PSCKITMLSCFLQELHVISYESNN---------------DEINQTVENIIILASSTLSSDE
platypus IL-15           FIDIDAMLYTD---KGQ         PNCKLTVMRCFLLELHVILHESDD---------------KTLNRTVWNVIITANSTLSSKT
chicken IL-15              DIDVSLYTAN-TYED         IECQEPVMRCFFLEMKVILHECDI-------------KKCSRKHDVRNIWKNGNARFATYQ
duck IL-15                 DIDVSLYTAN-TDED         KECQEPIMRCFFLEMNVILHECNI-------------KNCSKTQDVYNILKNGNASF-KNE
p. turtle IL-15 (partial)  SIDACLYTAY-TDKP         DECEVQVMECFLLELKVISHECEI------------RKGCKIKQHVRNVLANVKANLPKNTSLLNSP
lizard IL-15                VDASLYTA--TYS         NHCKISVVRCFHLEMKVLLYEANI----------------GGDSDIYNSTRRVLRSVGRFLDDEQ
frog IL-15                 PSSLTLYTAQENDIR         DACYNVILHCYFLEMRTVVEELTI-------------LKAEDTGELKLLHLLENLNISPT
gar IL-15 (partial)        SAGCVYYTP--EDYD         TDCTNTLFECYILEMEVIIFELDQ-----------------DYESVHSLQTFLEHAKKK
zebrafish IL-15            SSTAHLYTPKIDDIH          NCTFRFFDCFLLEMKVVLYEEGS---------------GEDSPGVVMVERTLQHYSQN
trout IL-15                KSDACLYAPTNDDIYN        DHCIFKFMHCYLLELEVVLFEDMS------------VTDNYHDEIKTSIYHRKKHLEEHERQY
stickleback IL-15          KSDAMLYAPSANEVK         ENCKMMSLKCYILELRMVIVEEVG-------------SENPKTHCVMDFNERLPDLDNGASAYP
pufferfish IL-15           KSDAMLYTPSANYVGWSSQGK   KNCKNKSLKCYMLELLMVIDEEAE--------------TGMYPNCINDFNARLLPDNS
                                                                                                    ◇
cattle IL-2         NPENLKLSRMHTFDFYVP----KVN        ATELKHLKCLLEEELKLLEEVLNLA-------PSKNLNPREIKDSMDNIKRIVLELQ
human IL-2          NYKNPKLTRMLTFKFYMP----KK         ATELKHLQCLEEELKPLEEVLNLA-------QSKNFHLR-PRDLISNINVIVLELK
                        11 11  11222                                 22  2  11  1
mouse IL-2          NYRNLKLPRMLTFKFYLP----KQ         ATELKDLQCLEDELGPLRHVLDLT-------QSKSFQLEDAENFISNIRVTVVKLK
opossum IL-2            GVSERMKRYELYVP----SR         ARSIADLQCFTKELHPVADALKY----------ESREARYIQDHIRNINVTVNRLM
chicken IL-2               KIHLELYTP--TETQ         ECTQQTLQCYLGEVVTLKKETED----------DTEIKEEFVTAIQNIEKNLKSLT
duck IL-2                  GIDLELYTP--NDTK         ECSWQTLQCYLKEIVTLEEEIED----------EDEIEDEKVSSVRNIKMNLQKLM
p. turtle IL-2            DIEYDGFYTP--EDFSN        EERRMEALGVYMAEMNTLKDESEN--------------NVMVNNIIDRIMKYLGAFG
C. turtle IL-2            SIENGGFYTP--EDFSN        EKRRKEALDVYMTEMNVLKEEIED--------------NPDANNTIALIKRNLQCM
lizard IL-2                NREGTLYTP--NITG         VKCARPAMECFMREIDILEEENEN---------------RNITNTIQNIKRNLRAGW
frog IL-2                  IIPEEFYTI--TDTGNGD      MKCRISALCCFVHELYTVEKYL-------------GTLNEKAGKQANNTITNALELL
gar IL-2 (partial)        GNKDGLYYTP--ADYK          SCKAKALKCFLLELDVVQMEY------------AKETDEEIDEAVAGLKDLGKK
zebrafish IL-2             ISDLTLYTP--TDIAP         GCLHMAGNCTLQELKVLKNDCFD----------LKEDDDDDKESLNNVLDLLEAE
trout IL-2                  PDSVFYTP--TDVE         DSCIVAALACSIKELDTVKVECLD------NAVHLESMQHHISMTATDLQKTIDKEN
stickleback IL-2           RSDSRFYAP--SDVT         EACITTALDCVMRELNGTVKeCDD----------SEQDILDAVESLNHVINRRTTAGH
pufferfish IL-2            EQDSKFYTP--TNIK         PECLTAALQCFKDELQTVKHECKD----------PQNYINRTKGFLERVISTMKNE
stickleback IL-2L           VNVTFTYP--INVQ         AKCSRDALQVFVQGLNNATTDCQD-----------------DQEIIPDTLESLAWKFPT
pufferfish IL-2L          GDGMNFTSP--TNVM         AKCHSAALGLFIQEFKKVYTHCGE------------------NSPVKQTIEVLERAHNK
cattle IL-21                DPEFLPAP--EDVK         RHCERSAFSCFQKVQLKSANN--------------GDNEKIINILTKQLKRKLPATNTGRRQKHEV
human IL-21                 VPEFLPAP--EDVE         TNCEWSAFSCFQKAQLKSANT--------------GNNERIINVSIKKLKRKPPSTNAGRRQKHRL
opossum IL-21               DPALLPTP--ENNE         KHCEEAAFKCFQEAQLKPSDH--------------QEKKMRFDTLIKQLRRRLPRKEANKTKP
chicken IL-21               DVELLHTP--ENPG         DGCLLTAVTCFQNGILKLQPK-------------NSQVNATFAKTVKILRRPFLPVSEE
p. turtle IL-21             DVEHPNTP--EDFE         SKCLSSAFNCFQNASLHLEPA-------------NSQSSRNFEVMITRLRRPIIIDTITDM
lizard IL-21                NATRLHSPP-IDTN          ECLLSTFTCFVNQSALLMPA-------------DRHRAKDFNISLARIKISPRFVELSNTNL
frog IL-21                  HPSFLHTP--VDVT         AECWESALNCFRYETSHLKEA-------------NGQKEEDFKRNIRKVYLKRMHTPENK
gar IL-21 (partial)                                DCCIPSALRCFKEMLPGLT-------------------TTDEQKSQKVKKMCYKMSNKTI
zebrafish IL-21          TNSNKTWFNSPATNDLK         DCCVASALECFRSKVLHLS--------------------VSDEKLKKSQRSVHHELRKSFI
trout IL-21                HNSVMSNAPS-MDTE         ECCSQSALKCFRAMVPHLK-----------------------AKNKTLQRKVIKNLNNRMI
stickleback IL-21          HNEKTLNIPP-QSFK         DRCCVSTLKYFLENLE----------------------KQFNASQDKLYRSLKQRKT
pufferfish IL-21           NRELLLTTPP-KNIE         EGCCLSALRCFRDSIQENI-------------------KSTVRLQRRLYKSLNNSHT
human IL-4               TLCTELTVTDI-FAASK         NTTEKETFCRAATVLRQFYSHHEKDTRCLGATAQQFHRHKQLIRFLKRLDRNLWGLAGL
chicken IL-4             VSCVKMNVTDI-FADNK         TNNKTELLCKASTIVWES-------QHC----------HKNL-QGLFLNMRQLLNASSTSLK
zebrafish IL-4/13B         DLDQFIPDI-YETGHYS            KKTLCQAGMAL-----------------------KGKIPSRSHLQRQLNAYAAYTG
```

```
                 Exon 3b           Exon 4 (3/4, 4a)                                       Exon 4b
                                                                 αD
                                              C     ·90        ·100      ·110
                                              C   C                    ▽  C  CCC
cattle IL-15L-----------------------------DQGPCP-PCEGHPQ-RPVPLFLAKLLELLQGTCARDLPSA
sheep IL-15L------------------------------GQGPCP-PCEGHPQ-RPVPLFLAKLLELLQGTCAQDLPSA
pig IL-15L--------------------------------GQGPCP-PCEGHPQ-RPVPLFLAKLLELLQGACARHLASA
horse IL-15L------------------------------GPGPCP-PCEGHPQ-RPVPLFLAKLLELLQGACARHLPSA
rhinoceros IL-15L-------------------------GQGPCP-PCEGHPQ-RPVPLFLAKLLELLQGACARRLPST
cat IL-15L--------------------------------GQGPCP-PCEGHPQ-RPVPLFLAKLLELLQGACARHLPSA
ferret IL-15L-----------------------------GQGPCP-PCEGHPQ-RPVPLFLAKLLELLQGACARHRPSA
hedgehog IL-15L---------------------------DQGPCP-PCEGHTQ-RPVHHFLSKLLELLQRACMRHLFSP
shrew IL-15L------------------------------DQGPCL-PCEGHPQ-RPVPHFLAKLLELLQGACAHNLLLA
human IL-15Lψ-----------------------------SQGPCL-SCEGHPQ-RPVP-----LLELLQGACAWHPTTA
lemur IL-15L------------------------------SQGPCL-PCEGHPQ-RPVPRFLAKLLELLQGACARHLRTA
rabbit IL-15L-----------------------------SQGPCL-PCEGHPQ-RPVPLFLAKLLELLQGACAQHLSTA
pika IL-15L-------------------------------SQGPCL-PCEGHPQ-RPVPLFLAKLLELLQGACAQQTFTA
hyrax IL-15L------------------------------GQGPCP-PCERHPQ-RPVPFFLAKLLELLQWACTGHLSRA
opossum IL-15L----------------------------APGLCP-PCEGYPE-RPVPQFLAKLLELMQVACAGAHQVG
wallaby IL-15L----------------------------ASGLCP-RCEEYPE-RPVLQFLTKLLEILHLACAGASTAG
platypus IL-15L---------------------------PQEGCP-PCEGHPE-QPTPRFLAKLLELLQVLCAPAHWGP
p. turtle IL-15L--------------------------TDRQCH-PCEGHRE-QPKPQFLSKLLEQLQWDCWVQGSNMHSCPSWPG
C. turtle IL-15L (partial)----------------TDRPCH-PCEGHQE-QPVPQFLNKLLELLQRECWLQGSNVHSCSS
lizard IL-15L (partial)------------PSANGANPPNTCP-PCHSHPE-RPVKDFLQKLQQLFQAQCRIVAQQRRTYDLVPVP
python IL-15L (partial)-------------------RCVACP-PCLSHPE-KPVMTFLRRLLELMQFHC
gar IL-15L (partial)                    KDQKADCP-QCEAHEE-QPPWEFLTALLSVLQSIN-DSC
zebrafish IL-15L                      LQDEMEMKCP-VCELYKE-ESTKTFLNTLQHILEQMNAERICAP
medaka IL-15L                             TEQDCL-QCELLEE-TSVDNFLQSLVSVLQNICSESAS
stickleback IL-15L                        TTSECR-QCERLQQ-KDAEEFLRELEGTLEKINSAPCPPEDSS
fugu IL-15L                                 PDCL-QCELSAE-RATKQFLRDLLTVLQHMNALNC
pufferfish IL-15L                         PQLDCP-ECELHRE-NAVLKFLENLLGVLQAIYVDNCPSRPPAGG
cattle IL-15                            NKTELGCK-ECEELEE-KSIKEFLKSFVHIVQMFINTS
human IL-15                             NVTESGCK-ECEELEE-KNIKEFLQSFVHIVQMFINTS
mouse IL-15                             NVAESGCK-ECEELEE-KTFTEFLQSFIRIVQMFINTS
                                                 2222  2
opossum IL-15                           NKAESGCK-ECEELEE-KNVGEFLKSFTHMVQSFIGTA
platypus IL-15                          STKELGCK-KCEEFEE-KNFAEFLDDFMSTVRSLNHPSNTT
chicken IL-15                         LNSTTAKKCK-ECEEYEE-KNFTEFIQSFVKVIQRECKKYAN
duck IL-15                            LSSTTSKKCK-ECEEYEE-KSFTEFIQNFVKVIQKECK
p. turtle IL-15                         NRNKITCK-QCEEYEE-RNFIKFIETFEEFAKHKLKFLPHVPK
lizard IL-15                            DKETSQCQ-ECETFEE-KQYTEFIENFQDIARRLHREEKWEK
frog IL-15                              VTQWGDCK-RCEEFQE-KDLPVFIEAFIEFIQMKYSDGP
gar IL-15 (partial)                       IFLHCK-RCEEYEE-KTAEAFLSKFQTFLQHLHSIGYNMTS
zebrafish IL-15                           NCSARH-PCELQEL-TNSTLFFDRMRNFLQKLIDNCGKKNNATCD
trout IL-15                               NSSRCS-PCEAQRV-ANSTIFLYNLERLLERIGQTVS
stickleback IL-15                           VDCP-PCEAYSL-KNITVFMERLNSLLQELNSMQT
pufferfish IL-15                            VGCP-PCEVYPQ-NNITIFLRELQNLLEEINVINRT
cattle IL-2                                GSETRFTCEYDDATVNAVEFLNKWITFCQSIYSTMT
human IL-2                                 GSETTFMCEYADETATIVEFLNRWITFCQSIISTLT
                                                  22222
mouse IL-2                                 GSDNTFECQFDDESATVVDFLRRWIAFCQSIISTSPQ
opossum IL-2                               GTATT-HCQYAVK-IKIRGFFGEWITFCQRLIHLTR
chicken IL-2                          GLNHTGSECK-ICEANNK-KKFPDFLHELTNFVRYLQK
duck IL-2                             DLIPPGTGCN-ICEANA--NNFPEFRQELTNFLRSMLK
p. turtle IL-2                            KNTTRK--YEGSQQ-KNTQEFLEGLRKLLQSMYSSMVPVKSH
C. turtle IL-2                            KIQERK-VCEGCQQ-KNTQEFLEGLRQLLQAMYSE
lizard IL-2                       KQVAGDAKEDEVCP-LCESYEE-QNYVDFLNRLVALVQFVLRTGFEKH
frog IL-2       KLLDASYSKTEPSQYLE        KQCEQCK-QHKAHTTTKNLEGFIEDFEALLKNLATNL
gar IL-2 (partial)                   LQFQPSSETCS-PCESYPR-NNSEQFFDEFTKFIKTSLITSS
zebrafish IL-2                      IKPCPNRKCECR-TCEEYEA-TDVMTFLKNVKNLDERMEAERK
trout IL-2                             STTDTSEC--ICEDKRLEKSFKDFIQNIRHLTQAHAAKRLSS
stickleback IL-2                       ARTDSNEC--TCERWPL-ASYAVFKKNTLNLLQMTNTMG
pufferfish IL-2                         EVNSNAC--SCESYSE-EPFPQFLNAMETLVQRFNSKARQNQQR
stickleback IL-2L                        TDSTNC--KLQTKE--SQFEDFVKDLERLVQLINASGDK
pufferfish IL-2L                         TQAEEC--TLTMTEH-KNFEDLIEAMKHFAQRINSAYYH
cattle IL-21                                 TCP-SCDSYEK-KPPKEYLERLKSLIQK                 MIHQHLS
human IL-21                                  TCP-SCDSYEK-KPPKEFLERFKSLLQK                 MIHQHLSSRTHGSEDS
opossum IL-21                                KCP-PCDSFKG-KPPHEFLNSLRSFLQK                 MIFRHQNRHCCYGHCT
chicken IL-21                                HCESSCESYER-KKPQEFLNSFSKLMQK                 LFKNSTAERYGKSTI
p. turtle IL-21                              NCS-PCESYAK-EAPRQFLDSFLSLLQE                 AK
lizard IL-21                                 KCP-PCGSYKK-VSEQDFLRSFETLLQR                 LNRMSWTKCKLHNSCQ
frog IL-21                                   GCP-ICTKYPT-QKPGDFLESMLLLLQQ                 IIADYPTQTSG
gar IL-21 (partial)                        KASCR-ACDSYEM-KDSKDFLNAMEKLLQK
zebrafish IL-21   VDSVSCTPQETQ             KAQCK-SCEAYTM-VNSRTFVDNFKTLLQK                 IYARQA
trout IL-21      LGSLDSCSQEERE             KTVCQ-GCDSYP--KDSQKCVQQLESLLQK                 AISRLA
stickleback IL-21  ERALCPSGDIQ              ANCQ-TYNSHP--GTVQVFFERLQSFIEE                 GITRLSMK
pufferfish IL-21 AACLNFCHSEN                ATCQ-TCNSHPQ-EKVGEFFSRLDSFIQK                 AISKLRSSAAN
human IL-4                                  NSCP--VKEANQ-STLENFLERLKTIMREKYSKCSS
chicken IL-4                                APCP--TAAGNT-TSMEKFLADLRTFFHQLAKNK
zebrafish IL-4/13B                         TGNCS--VSTSGE-CTMKEFLEKTKACCHYLYSAQRT
```

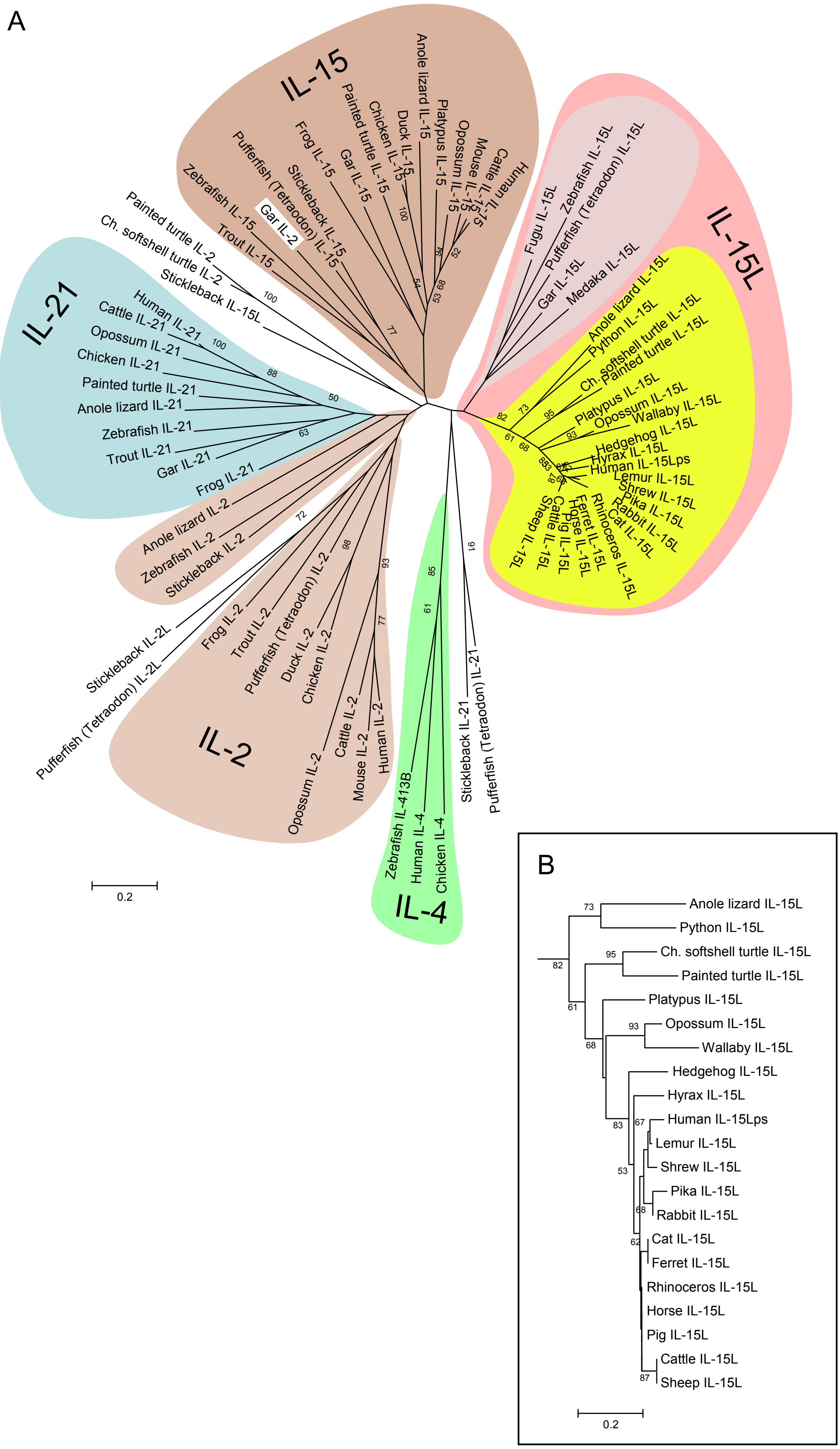
A
IL-15
IL-15L
IL-21
IL-2
IL-4
Anole lizard IL-15
Painted turtle IL-15
Chicken IL-15
Duck IL-15
Platypus IL-15
Opossum IL-15
Mouse IL-15
Cattle IL-15
Human IL-15
Frog IL-15
Gar IL-15
Stickleback IL-15
Pufferfish (Tetraodon) IL-15
Gar IL-2
Zebrafish IL-15
Trout IL-15
Painted turtle IL-2
Ch. softshell turtle IL-2
Stickleback IL-15L
Human IL-21
Cattle IL-21
Opossum IL-21
Chicken IL-21
Painted turtle IL-21
Anole lizard IL-21
Zebrafish IL-21
Trout IL-21
Gar IL-21
Frog IL-21
Anole lizard IL-2
Zebrafish IL-2
Stickleback IL-2
Stickleback IL-2L
Pufferfish (Tetraodon) IL-2L
Frog IL-2
Trout IL-2
Pufferfish (Tetraodon) IL-2
Duck IL-2
Chicken IL-2
Cattle IL-2
Opossum IL-2
Mouse IL-2
Human IL-2
Zebrafish IL-413B
Human IL-4
Chicken IL-4
Stickleback IL-21
Pufferfish (Tetraodon) IL-21
Fugu IL-15L
Zebrafish IL-15L
Pufferfish (Tetraodon) IL-15L
Gar IL-15L
Medaka IL-15L
Anole lizard IL-15L
Python IL-15L
Ch. softshell turtle IL-15L
Painted turtle IL-15L
Platypus IL-15L
Opossum IL-15L
Wallaby IL-15L
Hedgehog IL-15L
Hyrax IL-15L
Human IL-15Lps
Lemur IL-15L
Shrew IL-15L
Pika IL-15L
Rabbit IL-15L
Cat IL-15L
Rhinoceros IL-15L
Ferret IL-15L
Pig IL-15L
Horse IL-15L
Cattle IL-15L
Sheep IL-15L
0.2
B
73
Anole lizard IL-15L
Python IL-15L
95
Ch. softshell turtle IL-15L
82
Painted turtle IL-15L
Platypus IL-15L
61
93
Opossum IL-15L
68
Wallaby IL-15L
Hedgehog IL-15L
Hyrax IL-15L
83
67
Human IL-15Lps
Lemur IL-15L
53
Shrew IL-15L
Pika IL-15L
68
Rabbit IL-15L
62
Cat IL-15L
Ferret IL-15L
Rhinoceros IL-15L
Horse IL-15L
Pig IL-15L
Cattle IL-15L
87
Sheep IL-15L
0.2

Fig. S6*C*

Similarity levels (percentages of identical amino acids) between representative IL-15L molecules and related cytokines

		IL-15L					IL-15							IL-2							IL-21						
		Pufferfish IL-15L	Turtle IL-15L	Opossum IL-15L	Cattle IL-15L	Rabbit IL-15L	Zebrafish IL-15	Pufferfish IL-15	Chicken IL-15	Turtle IL-15	Opossum IL-15	Cattle IL-15	Human IL-15	Zebrafish IL-2	Pufferfish IL-2	Chicken IL-2	Turtle IL-2	Opossum IL-2	Cattle IL-2	Human IL-2	Zebrafish IL-21	Pufferfish IL-21	Chicken IL-21	Turtle IL-21	Opossum IL-21	Cattle IL-21	Human IL-21
IL-15L	Zebrafish IL-15L	27	19	24	24	25	21	25	16	17	17	24	23	22	20	17	13	12	13	15	21	17	16	21	24	19	17
	Pufferfish IL-15L		26	28	32	30	20	24	24	19	22	22	22	22	20	19	18	13	18	19	16	20	14	21	20	21	23
	Turtle IL-15L			34	37	41	22	19	17	18	23	25	24	25	24	20	18	15	19	17	18	20	16	22	19	15	18
	Opossum IL-15L				58	57	2	23	20	22	25	20	20	23	25	20	19	13	15	14	18	22	18	18	19	16	15
	Cattle IL-15L					79	24	25	21	20	20	20	22	22	21	20	20	16	16	16	22	23	15	19	17	16	15
	Rabbit IL-15L						24	25	19	22	21	23	20	24	20	20	22	14	17	17	22	21	15	21	15	16	14
IL-15	Zebrafish IL-15							22	25	26	29	28	28	21	18	22	15	21	20	20	20	17	17	15	20	14	17
	Pufferfish IL-15								16	18	19	24	25	20	19	20	21	16	15	17	17	16	16	19	22	17	18
	Chicken IL-15									43	37	37	40	22	22	24	17	15	15	15	14	20	20	14	17	13	14
	Turtle IL-15										37	37	40	27	23	24	16	17	17	17	22	16	15	13	14	15	16
	Opossum IL-15											61	63	24	21	26	19	17	23	22	17	18	21	15	17	18	21
	Cattle IL-15												74	24	22	23	18	17	16	21	18	16	20	16	17	17	20
	Human IL-15													23	25	22	15	15	19	21	19	20	23	15	17	17	18
IL-2	Zebrafish IL-2														27	22	19	11	20	15	21	16	20	20	15	18	18
	Pufferfish IL-2															21	19	18	19	20	19	22	20	18	19	16	17
	Chicken IL-2																28	24	25	24	13	20	16	17	21	18	18
	Turtle IL-2																	20	24	18	16	15	18	18	16	19	18
	Opossum IL-2																		38	40	14	19	11	14	13	12	14
	Cattle IL-2																			66	16	13	17	15	16	14	16
	Human IL-2																				18	16	13	13	15	13	13
IL-21	Zebrafish IL-21																					20	20	23	14	17	18
	Pufferfish IL-21																						19	22	21	23	20
	Chicken IL-21																							47	31	31	30
	Turtle IL-21																								34	29	34
	Opossum IL-21																									49	44
	Cattle IL-21																										82
