# Supplementary material for: Identification of a gene for an ancient cytokine, interleukin 15-like, in mammals; interleukins 2 and 15 co-evolved with this third family member, all sharing binding motifs for IL-15Rα
Source: Immunogenetics. 2013 Nov 26;66(2):93–103. doi: 10.1007/s00251-013-0747-0 (PMC3894449; doi:10.1007/s00251-013-0747-0)
Supplement: Supplementary file 10 — (PDF 490 kb) [file 251_2013_747_MOESM10_ESM.pdf]

# **Supplementary Figure 7 (Fig. S7).**
DNA plasmid expression vectors and Western blot analysis

Table of Contents:


| | | |
|---|---|---|
| **Legends to Figures S7*A* and S7*B*** | | Page 2 |
| **Fig. S7*A*** | DNA plasmid vectors for eukaryotic expression of bovine IL-15L, IL-15Rα and IL-2Rα | Page 4 |
| **Fig. S7*B*** | Interaction between bovine IL-15L-FLAG or IL-15L and soluble IL-15Rα (as shown by Western blot analysis of supernatants of transfected cells) | Page 6 |

**Legends to Figures S7*A* and S7*B***

**Fig. S7*A***

DNA plasmid vectors for eukaryotic expression of bovine IL-15L, IL-15Rα and IL-2Rα. Bovine *IL-15L*, *IL-15Rα* and *IL-2Rα* genes were cloned into commercial plasmid vectors as indicated in Text S1.1. The figure shows the relevant sequence fragments of the resulting expression vectors with encoded amino acids below the second nucleotides of codons. IL-15L, IL-15Rα, and IL-2Rα amino acid sequences are in black font. FLAG-tag, Myc-tag, and poly-His-tag are in red, purple, and green, respectively. Non-tag residues encoded by the vector are in gray.

**Fig. S7*B***

Interaction between bovine IL-15L-FLAG or IL-15L and soluble IL-15Rα (as shown by Western blot analysis of supernatants of transfected cells).

Two similar sets of cell transfection experiments were done, one using expression vector *pRcCMV2-Bos-IL-15L-FLAG* [left panel; blots (a) and (b)] and the other using *pRcCMV2-Bos-IL-15L* [right panel; blots (c) and (d)]. The difference between the two experiments is the presence or absence of a FLAG-tag added to IL-15L. Cells were transfected with these constructs alone (lanes 1, 4 and 7), with these constructs plus *pcDNA3.1-Bos-sIL-15Rα-Myc-His* (lanes 2, 5 and 8) for expression of soluble IL-15Rα (sIL-15Rα), or not transfected for use as negative controls (lanes 3, 6 and 9). For Western blot analysis cell lysates (lanes 1-3), concentrated supernatants (lanes 4-6), and concentrated supernatants after nickel affinity chromatography (lanes 7-9), were run in

SDS-12% polyacrylamide gel systems, and transferred to nylon membranes which were probed with anti-Myc mAb [(a) and (c)], anti-FLAG mAb (b), or anti-Bos-IL-15L polyclonal antibodies (d).

Results of both sets of experiments were identical, namely that co-expression with soluble IL-15Rα was needed to detect IL-15L in the cell supernatant [compare lanes 4 and lanes 5 in blots (b) and (d)]. The atypical running behavior in SDS-page gel of the IL-15L protein seen in the supernatant samples [lanes 5 in blots (b) and (d)] was most likely caused by detergent in the expression medium that had been concentrated during sample preparation (see supplementary Materials and methods in Text S1.1), because after purification using nickel affinity chromatography the IL-15L protein regained its running behavior in agreement with the expected molecular weight of 13 kDa [lanes 8 in blots (b) and (d)]. For unknown reason the isolation of poly-His-tagged sIL-15Rα by nickel affinity chromatography was not very efficient, and rather the results of lanes 4-6 than of lanes 7-9 in both sets of experiments allow conclusions on interaction between IL-15L and sIL-15Rα.

Note that sIL-15Rα is efficiently secreted and not clearly detectable in the cell lysates, in contrast to IL-15L. Some bands of the approximate size of sIL-15Rα are found in the cell lysates, but those are probably non-related background bands because they are also found in non-transfected cell lysates.

M, visible size markers are from “MagicMark” (Invitrogen) while the 3, 10 and 15 kDa indications derive from another size marker. Sample loads in lanes 4-6, as well as those in lanes 7-9, roughly corresponded to the supernatants of 4x the amount of cells loaded in lanes 1-3.

# Fig. S7*A*

DNA plasmid vectors for eukaryotic expression of bovine IL-15L, IL-15Rα and IL-2Rα

## *pRcCMV2-Bos-IL-15L*

TAGGGAGACCCAAGCTTACCATGTGGCTTCTCTGGACCACCCTCCTGCTGGTGCTGCCCTTGGGAGGCCTAGGACCACTCCTCTGCCCAAGGGAGCCTTTCTACTTCCTCATTGCCATCACGAAGATGCTGG
M W L L W T T L L L V L P L G G L G P L L C P R E P F Y F L I A I T K M L

AAAACAAAAATGATGGCAGTCTGTACACCCCAGATAATCTATTGGTGTGTCCTGCTGAGACTCTCCGATGCTTCCGGCTGGAGTTGTCTGTGATCGGGTTTGAGGAGGGCCCATCCGTGGGGATCGTTGTGT
E N K N D G S L Y T P D N L L V C P A E T L R C F R L E L S V I G F E E G P S V G I V V

TCCGCCTACAGCGCCTACTGGATGCCCTGGGGTCCCAGCTGTGGGTGATTGATCAGGGCCCTTGTCCACCCTGCGAAGGACACCCTCAGAGACCAGTCCCTCTTTTTCTGGCCAAACTCTTGGAGTTATTAC
F R L Q R L L D A L G S Q L W V I D Q G P C P P C E G H P Q R P V P L F L A K L L E L L

AGGGGACTTGTGCTCGGGACCTGCCCTCAGCATAATCTAGAGGGCCCGTTTAAAC
Q G T C A R D L P S A *

## *pRcCMV2-Bos-IL-15L-FLAG*

TAGGGAGACCCAAGCTTACCATGTGGCTTCTCTGGACCACCCTCCTGCTGGTGCTGCCCTTGGGAGGCCTAGGACCACTCCTCTGCCCAAGGGAGCCTTTCTACTTCCTCATTGCCATCACGAAGATGCTGG
M W L L W T T L L L V L P L G G L G P L L C P R E P F Y F L I A I T K M L

AAAACAAAAATGATGGCAGTCTGTACACCCCAGATAATCTATTGGTGTGTCCTGCTGAGACTCTCCGATGCTTCCGGCTGGAGTTGTCTGTGATCGGGTTTGAGGAGGGCCCATCCGTGGGGATCGTTGTGT
E N K N D G S L Y T P D N L L V C P A E T L R C F R L E L S V I G F E E G P S V G I V V

TCCGCCTACAGCGCCTACTGGATGCCCTGGGGTCCCAGCTGTGGGTGATTGATCAGGGCCCTTGTCCACCCTGCGAAGGACACCCTCAGAGACCAGTCCCTCTTTTTCTGGCCAAACTCTTGGAGTTATTAC
F R L Q R L L D A L G S Q L W V I D Q G P C P P C E G H P Q R P V P L F L A K L L E L L

AGGGGACTTGTGCTCGGGACCTGCCCTCAGCAGATTACAAGGATGACGACGATAAGTAATCTAGAGGGCCCGTTTAAAC
Q G T C A R D L P S A D Y K D D D D K *

## *pcDNA3.1-Bos-sIL-15Rα-Myc-His*

GCTGGCTAGTTAAGCTTACCATGTCCGGGCGGCTCCGGGGCCGCGGGGCCGGCGCCCTCCCCGCGCTGGGGCTGCTGCTGCTACTACTGCTGCTCGGATCTTCGGCCACGCCGGGCATCACCTGCCCGACTC
M S G R L R G R G A G A L P A L G L L L L L L L G S S A T P G I T C P T

CCACATCCGTGGAGCATGCAGACATCCAGGTCAAGAGTTACAGCATCAACTCCAGGGAGCGGTATGTTTGTAATTCTGGCTTCAAGCGTAAAGCTGGGACTTCCAGCTTGACCCAGTGTGTGTTTAACGAGA
P T S V E H A D I Q V K S Y S I N S R E R Y V C N S G F K R K A G T S S L T Q C V F N E

CCGCGAAAGTCGCCCACTGGACCACTCCCAACCTCAAGTGCATCAGAGACCCCTCCCTGAGTCACCAAAGGCCACCCTCCACAGCAGCGCCTACAGGGTTGACCCCAGAGCCAGAGAGCCCCACCCCCTCCG
T A K V A H W T T P N L K C I R D P S L S H Q R P P S T A A P T G L T P E P E S P T P S

GAAAAGAGCCAGATCTTACTTCCAAGTCAGACACCAAAGTGGCCACAAGGCCAGCTACTGGACCAGGCTCCAGGCTGCCATCCACAGCTCCTCCTGTGGGAACCACAGGGGTAGTCAGTAAGGAGACCACCT
G K E P D L T S K S D T K V A T R P A T G P G S R L P S T A P P V G T T G V V S K E T T

ACGTCCCAGCTCAGACAGCAGCCAAGGCTCCGGAACACACATACCCGGCCTTGCAGGACACGCCCGGTGCATATCAGAACAATCCCAGCTCGAGTCTAGAGGGCCCGCGGTTCGAACAAAAACTCATCTCAG
Y V P A Q T A A K A P E H T Y P A L Q D T P G A Y Q N N P S S S L E G P R F E Q K L I S

AAGAGGATCTGAATATGCATACCGGTCATCATCACCATCACCATTGAGTTTAAACCCGCTGATCAGC
E E D L N M H T G H H H H H H *

## *pcDNA3.1-Bos-IL-15R$\alpha$-Myc-His*

```
GCTGGCTAGTTAAGCTTACCATGTCCGGGCGGCTCCGGGGCCGCGGGGCCGGCGCCCTCCCCGCGCTGGGGCTGCTGCTGCTACTACTGCTGCTCGGATCTTCGGCCACGCCGGGCATCACCTGCCCGACTC
                      M  S  G  R  L  R  G  R  G  A  G  A  L  P  A  L  G  L  L  L  L  L  L  L  L  G  S  S  A  T  P  G  I  T  C  P  T
CCACATCCGTGGAGCATGCAGACATCCAGGTCAAGAGTTACAGCATCAACTCCAGGGAGCGGTATGTTTGTAATTCTGGCTTCAAGCGTAAAGCTGGGACTTCCAGCTTGACCCAGTGTGTGTTTAACGAGA
P  T  S  V  E  H  A  D  I  Q  V  K  S  Y  S  I  N  S  R  E  R  Y  V  C  N  S  G  F  K  R  K  A  G  T  S  S  L  T  Q  C  V  F  N  E
CCGCGAAAGTCGCCCACTGGACCACTCCCAACCTCAAGTGCATCAGAGACCCCTCCCTGAGTCACCAAAGGCCACCCTCCACAGCAGCGCCTACAGGGTTGACCCCAGAGCCAGAGAGCCCCACCCCCTCCG
T  A  K  V  A  H  W  T  T  P  N  L  K  C  I  R  D  P  S  L  S  H  Q  R  P  P  S  T  A  A  P  T  G  L  T  P  E  P  E  S  P  T  P  S
GAAAAGAGCCAGATCTTACTTCCAAGTCAGACACCAAAGTGGCCACAAGGCCAGCTACTGGACCAGGCTCCAGGCTGCCATCCACAGCTCCTCCTGTGGGAACCACAGGGGTAGTCAGTAAGGAGACCACCT
G  K  E  P  D  L  T  S  K  S  D  T  K  V  A  T  R  P  A  T  G  P  G  S  R  L  P  S  T  A  P  P  V  G  T  T  G  V  V  S  K  E  T  T
ACGTCCCAGCTCAGACAGCAGCCAAGGCTCCGGAACACACATACCCGGCCTTGCAGGACACGCCCGGTGCATATCAGAACAATCCCAGAGTTGTGACCGCCGTCTCAACTGTCACTGTGCTCTTTGTAGTAT
Y  V  P  A  Q  T  A  A  K  A  P  E  H  T  Y  P  A  L  Q  D  T  P  G  A  Y  Q  N  N  P  R  V  V  T  A  V  S  T  V  T  V  L  F  V  V
GCCTGGTGTTTCTTCTGGGACGTTGCCTGTGGTCAAGGCGAGCCCACCAGACACCCGGTGTTGAGATGGAGAGCATGGAGAGTGTGCCAATGACCACGGGGGCCGATGCCAGAGGGGAGGACACAGAAATCC
C  L  V  F  L  L  G  R  C  L  W  S  R  R  A  H  Q  T  P  G  V  E  M  E  S  M  E  S  V  P  M  T  T  G  A  D  A  R  G  E  D  T  E  I
ACCCGCATGGCCTAGGAGGCTCCGGGGACGCTGAGGCCAGCAGCGGCCGCAGTGAAGGCCCAGCTCTTCCCCAGTCAGAGAGGACCTCGAGTCTAGAGGGCCCGCGGTTCGAACAAAAACTCATCTCAGAAG
H  P  H  G  L  G  G  S  G  D  A  E  A  S  S  G  R  S  E  G  P  A  L  P  Q  S  E  R  T  S  S  L  E  G  P  R  F  E  Q  K  L  I  S  E
AGGATCTGAATATGCATACCGGTCATCATCACCATCACCATTGAGTTTAAACCCGCTGATCAGC
E  D  L  N  M  H  T  G  H  H  H  H  H  H  *
```

## *pcDNA3.1-Bos-IL-2R$\alpha$-Myc-His*

```
GCTGGCTAGTTAAGCTTACCATGGAGCCCAGCTTGCTGATGTGGAGGTTCTTCGTATTCATCGTGGTACCTGGCTGCGTGACAGAGGCTTGTCATGATGACCCTCCGAGTCTCAGAAACGCCATGTTCAAGG
                      M  E  P  S  L  L  M  W  R  F  F  V  F  I  V  V  P  G  C  V  T  E  A  C  H  D  D  P  P  S  L  R  N  A  M  F  K
TCTTCAGGTACGAGGTGGGCACCATGATAAACTGCGACTGCAAGACAGGCTTCCGCAGAGTGTCGGCCGTCATGCGCTGCGTGGGGGACTCCAGCCACTCTGCCTGGGAAAACAGATGCTTCTGCAACAGCA
V  F  R  Y  E  V  G  T  M  I  N  C  D  C  K  T  G  F  R  R  V  S  A  V  M  R  C  V  G  D  S  S  H  S  A  W  E  N  R  C  F  C  N  S
CCTCCCCTGCTAAGAACCCAGTAAAACAAGTCACTCCTGCACCCGAAGAACAGAGGGAGAAAAAACCCACAGATGCGCAGAACCAAACGCAGCCTCCGGAGGAAGCTGACCTTCCAGGTCACTGTGAGGAAC
T  S  P  A  K  N  P  V  K  Q  V  T  P  A  P  E  E  Q  R  E  K  K  P  T  D  A  Q  N  Q  T  Q  P  P  E  E  A  D  L  P  G  H  C  E  E
CGCCACCATGGGAACACGAACGTGAACCTTTAAAGAGAGTCTACCATTTCACGCTGGGGCAGACGGTTCATTACCAGTGCGCCCAGGGATTCAGGGCCCTACAGACCAGTCCTGCTGAAAGCACCTGCATGA
P  P  P  W  E  H  E  R  E  P  L  K  R  V  Y  H  F  T  L  G  Q  T  V  H  Y  Q  C  A  Q  G  F  R  A  L  Q  T  S  P  A  E  S  T  C  M
TGATCAACGGGGAGCTGAGGTGGACCAGGCCCAGGCTCAAGTGCATACGTGAAGGGGAGCACGGTCAGGCTTCAGATGACGCAGAGCCTCAGGAGAGCACGGAAGCTCCCCCTGGGAGTGGAACTTTCTTAC
M  I  N  G  E  L  R  W  T  R  P  R  L  K  C  I  R  E  G  E  H  G  Q  A  S  D  D  A  E  P  Q  E  S  T  E  A  P  P  G  S  G  T  F  L
CAACCAGGATGGCAGGGACCACAGATTTCCAGAAGCCCACAGATGAGATTGCAACGCTGGATACGTTCATATTTACCACTGAGTACCAGATTGCAGTGGCCGGCTGCACCCTCCTGCTCGCCAGCATCCTCC
P  T  R  M  A  G  T  T  D  F  Q  K  P  T  D  E  I  A  T  L  D  T  F  I  F  T  T  E  Y  Q  I  A  V  A  G  C  T  L  L  L  A  S  I  L
TCCTGAGCTGCCTCACCTGGCAGCGGAAATGGAAGAAGAACAGAAGGACAATCTCGAGTCTAGAGGGCCCGCGGTTCGAACAAAAACTCATCTCAGAAGAGGATCTGAATATGCATACCGGTCATCATCACC
L  L  S  C  L  T  W  Q  R  K  W  K  K  N  R  R  T  I  S  S  L  E  G  P  R  F  E  Q  K  L  I  S  E  E  D  L  N  M  H  T  G  H  H  H
ATCACCATTGAGTTTAAACCCGCTGATCAGCCTCGA
H  H  H  *
```

**Fig. S7*B***
Interaction between bovine IL-15L-FLAG (left panel) or IL-15L (right panel) and soluble IL-15Rα

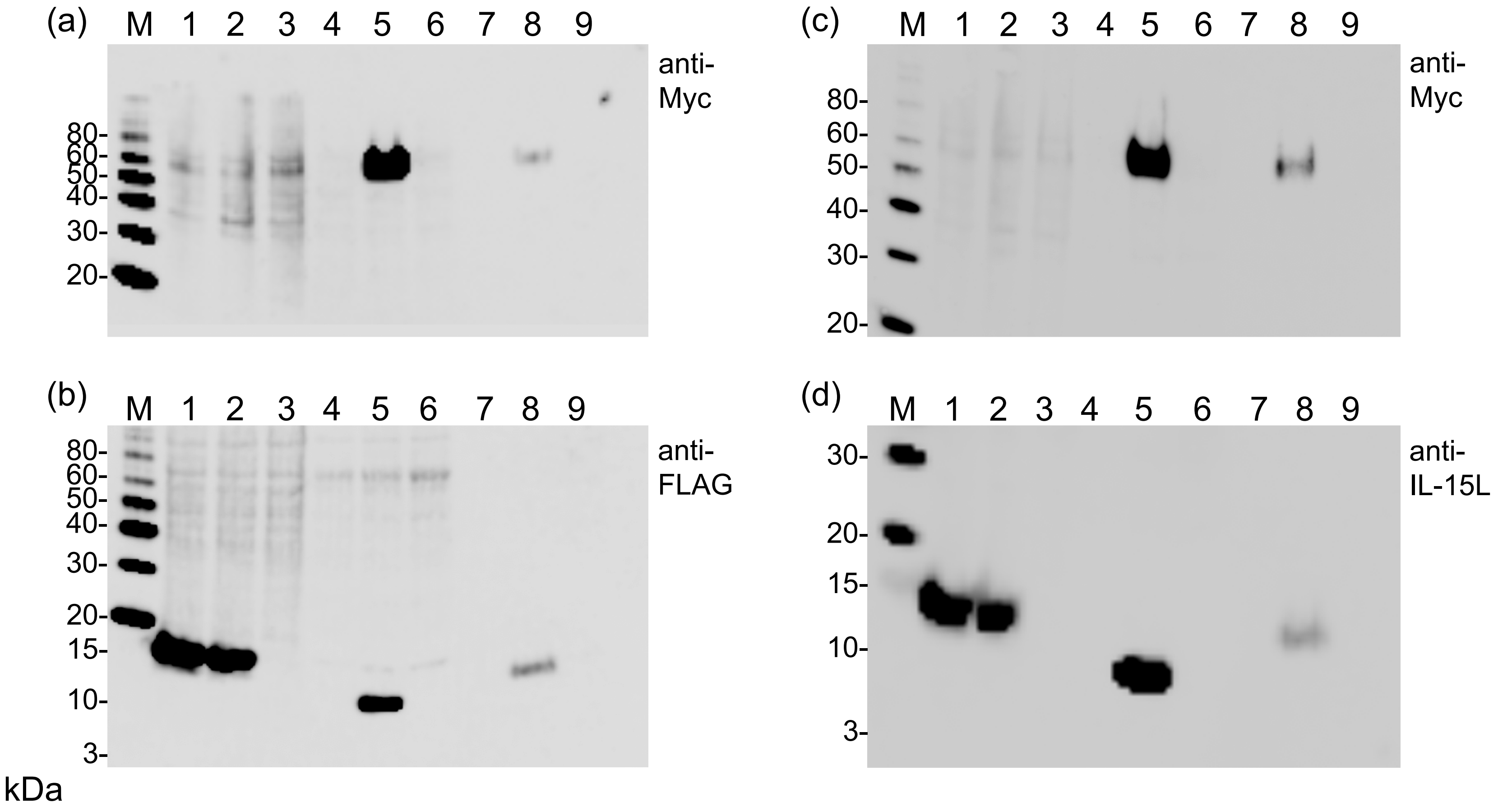


1, 4, 7: cells transfected for IL-15L-FLAG or IL-15L
2, 5, 8: cells transfected for IL-15L-FLAG or IL-15L, plus sIL-15Ra
3, 6, 9: non-transfected control cells
1-3: cell lysates
4-6: concentrated supernatants
7-9: concentrated supernatants after purification by Ni-column
